# Geographic Variation in Personality is Associated With Fertility Across the United States

**DOI:** 10.5964/ps.7275

**Published:** 2021-12-16

**Authors:** Eleanor J. Junkins, Joseph E. Potter, Peter J. Rentfrow, Samuel D. Gosling, Jeff Potter, K. Paige Harden, Elliot M. Tucker-Drob, Jaime Derringer, Daniel A. Briley

**Affiliations:** [1] Department of Psychology, University of Illinois at Urbana-Champaign, Champaign, IL, USA.; [2] Department of Sociology, University of Texas at Austin, Austin, TX, USA.; [3] Population Research Center, University of Texas at Austin, Austin, TX, USA.; [4] Department of Psychology, University of Cambridge, Cambridge, United Kingdom.; [5] Department of Psychology, University of Texas at Austin, Austin, TX, USA.; [6] School of Psychological Sciences, University of Melbourne, Melbourne, Australia.; [7] Atof, Cambridge, USA.

**Keywords:** personality, fertility, demography, geographic variation, Big Five

## Abstract

Levels of fertility and the shape of the age-specific fertility schedule vary substantially across U.S. regions with some states having peak fertility relatively early and others relatively late. Structural institutions or economic factors partly explain these heterogeneous patterns, but regional differences in personality might also contribute to regional differences in fertility. Here, we evaluated whether variation in extraversion, agreeableness, conscientiousness, neuroticism, and openness to experience measured at the U.S. state-level was associated with the level, timing, and context of fertility across states above and beyond sociodemographics, voting behavior, and religiosity. Generally, states with higher levels of agreeableness and conscientiousness had more traditional fertility patterns, and states with higher levels of neuroticism and openness had more nontraditional fertility patterns, even after controlling for established correlates of fertility (r ~ |.50|). Personality is an overlooked correlate that can be leveraged to understand the existence and persistence of fertility differentials.

Geographical regions across the United States differ widely in their fertility norms—how many children individuals have, when they have children, what actions they take to control their fertility, and the family contexts in which fertility takes place. Fertility has a tremendous impact on the well-being of society through effects on national growth, economic stability, and population aging (Bloom et al., 2010; Demeny, 2003; Harper, 2014; Morgan, 2003). Moreover, reproductive health policies are hotly debated in science, politics, and culture (Mills et al., 2011). Conventional accounts of geographic variation in fertility center on interactions among regional institutional, political, economic, and religious forces and individual behavior (D’Addio & d’Ercole, 2005; Glass & Levchak, 2014; Lesthaeghe, 2014; Lesthaeghe & Neidert, 2006; Morgan, 1996).

A nascent body of research indicates that personality—contextually and developmentally stable patterns of thinking, feeling, and behaving (John et al., 2008)—is predictive of fertility outcomes at the individual-level in both human (Berg et al., 2013; Briley et al., 2017; Courtiol et al., 2012; Hutteman et al., 2013; Jokela, 2012; Jokela et al., 2009; Rajan et al., 2017) and non-human populations (Aplin et al., 2013; Réale et al., 2009; Seyfarth et al., 2012). Moreover, geographic variability in personality is associated with many conventional geographic predictors of fertility (Rentfrow et al., 2013). However, no research has yet considered geographic variation in personality as an incremental predictor of regional fertility.

Using a sample of 890,253 U.S. residents, we show here that, even beyond a wide array of previously established correlates, states with relatively high average levels of extraversion, agreeableness, and conscientiousness and lower levels of neuroticism and openness tend to display more traditional fertility (i.e., higher fertility, earlier fertility, and more structured practices). These findings point to an entirely new kind of correlate of reproductive rates with important implications for population projections and cultural divides surrounding fertility practices.

## Psychosocial Geographic Variation in the United States

In the United States, some regions have individuals who are, on average, more extraverted (e.g., outgoing vs. timid), agreeable (e.g., warm vs. confrontational), conscientious (e.g., disciplined vs. accidental), neurotic (e.g., anxious/depressed vs. emotionally stable), and open (e.g., creative/intellectual vs. conventional) than other regions (Rentfrow et al., 2008). Regional variation in personality constructs could emerge from several causal processes, such as the environmental context socializing personality development or selective migration of individuals with certain personality traits (Rentfrow et al., 2008), or from methodological artifacts, such as reference group effects (Heine et al., 2002, 2008). However, there are several known properties of regional personality estimates that indicate construct validity (Mõttus et al., 2010), including that regional personality has been shown to correlate with personal spending (Ebert et al., 2021) and individual life satisfaction (Stavrova, 2015) over and above individual personality. Regional personality differences are stable across time (Elleman et al., 2018). Geographic variation in personality is correlated with such diverse outcomes as presidential voting patterns (Rentfrow et al., 2009), entrepreneurial activity (Obschonka et al., 2013), and several other economic (e.g., wealth and human capital), sociological (e.g., social capital, social tolerance, and residential mobility) and health (e.g., well-being and lifestyle choices) indicators (Rentfrow et al., 2013).

The associations between such phenomena and regional concentrations of personality may result from both top-down influences of social institutions on psychological development (e.g., living in an active artistic community affects levels of openness) and bottom-up influences of personality on the creation of social structures and outcomes (e.g., concentrations of highly open individuals generate artistic social settings). Additionally, individuals with certain personality traits may systematically migrate to some regions and away from others (Rentfrow et al., 2008). Links between regional personality and fertility may emerge from shared ecological-level influences (e.g., influential religious institutions may increase levels of both fertility and agreeableness) and from the aggregation of individual-level personality effects on fertility (e.g., agreeable individuals may tend to form certain family types, and this effect sums across many individuals living in a region).

To account for persistent regional differences in fertility, previous explanations have largely centered on regional differences in political, economic, or religious characteristics (Lesthaeghe & Neidert, 2006). However, these explanations may be limited to the extent that the individuals that generate the fertility schedule differ across regions. Several converging lines of evidence indicate that personality (i.e., consistent patterns of behavior that vary across individuals) may be a complementary explanatory variable to political, religious, or economic influences. First, personality is an enduring feature of an individual’s psychology (Conley, 1984). Second, individual differences are measurable very early in development (Measelle et al., 2005), and these initial differences are highly predictive of adult personality (Caspi et al., 2003). Third, geographic variability in personality is associated with many conventional geographic predictors of fertility (Rentfrow et al., 2013). Fourth, time-ordered relations have been found between personality and the formation of select sociodemographics, such as political preferences (Sibley & Duckitt, 2010) and religious beliefs (Wink et al., 2007). Finally, personality is predictive of individual-level fertility outcomes (e.g., Berg et al., 2013; Briley et al., 2017; Jokela et al., 2011; Miller, 1992).

These pieces of information point to personality as an enduring individual differences variable that may play a role in persistent state-level variation in fertility, above and beyond the influence of political orientation, religiosity, or economic constraints. To date, no study has examined such links.

## The Current Study

We constructed measures of state-level extraversion, agreeableness, conscientiousness, neuroticism, and openness to experience as our primary independent variables. As our dependent variable, we primarily focus on the total fertility rate (i.e., the average number of children that would be born to a woman if she experienced the age-specific fertility rates that prevailed in a given period through her lifetime). This rate has the most direct impact on the global population through cohort replacement. Subreplacement fertility (i.e., total fertility rates below 2.1) could restructure the age distribution of the population causing economic instability (Bloom et al., 2010; Demeny, 2003; Harper, 2014; Morgan, 2003). In areas with low fertility, cohorts are not replaced, which causes population aging along with economic burden associated with health care and loss of productivity.

**Figure 1:**
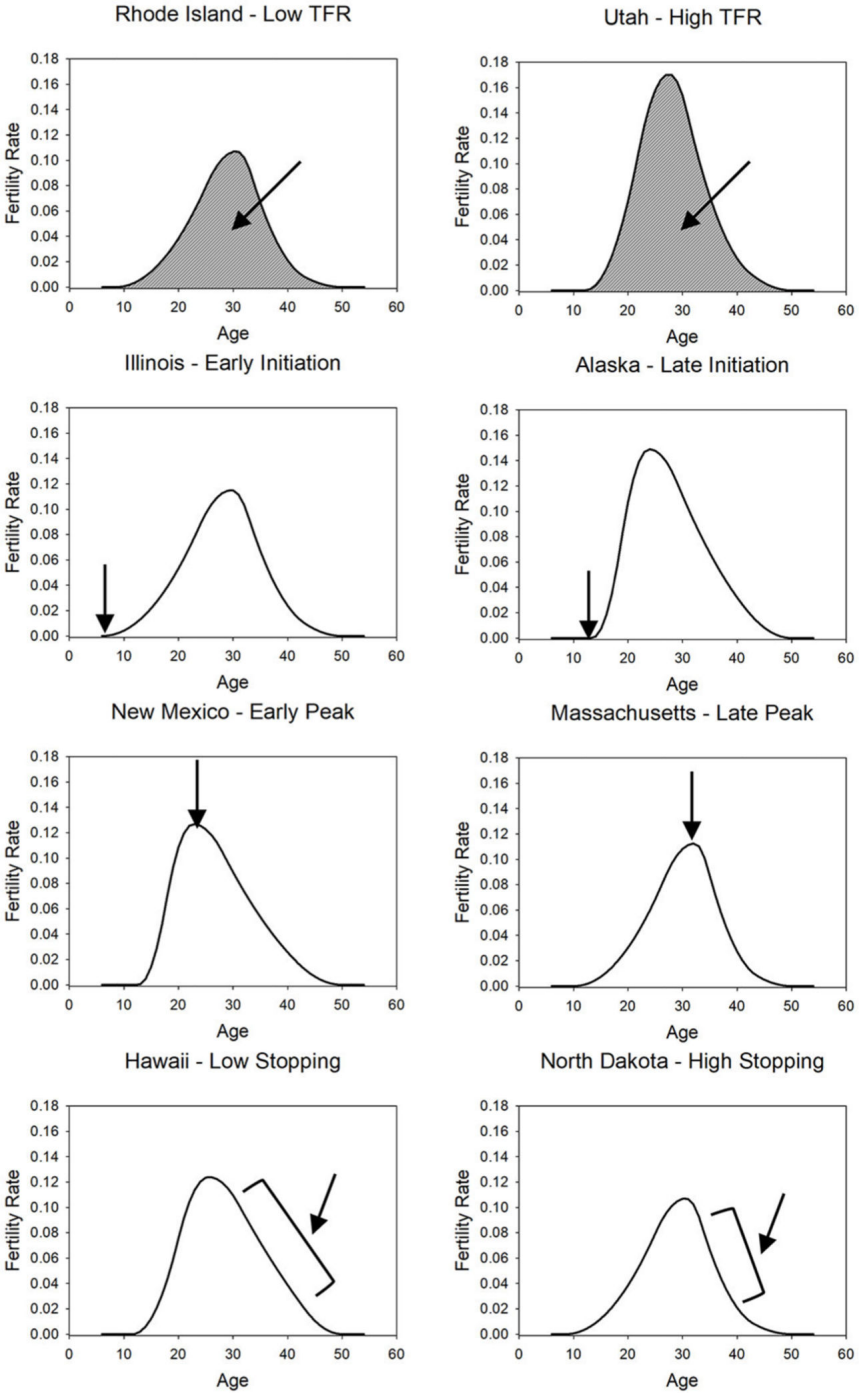
Example Fertility Schedules for Eight States With the Lowest and Highest Values for Total Fertility, Initiation, Peak, and Stopping *Note*. TFR = Total Fertility Rate. TFR represents the area under the curve. Initiation represents the earliest age with a non-zero fertility rate. Peak represents the age that fertility is highest. Stopping represents the force of limiting fertility after peak fertility, which is conceptually analogous to the slope of the curve after the peak.

We also included other features of the fertility schedule (highlighted in Figure 1; Schmertmann, 2003) as outcomes, such as the initiation age (i.e., the age at which fertility begins), peak fertility (i.e., the age at which fertility is highest), as well as stopping (i.e., the rate at which fertility falls following the peak), and markers of fertility-relevant behaviors identified by previous research as central to heterogeneous regional fertility (Lesthaeghe & Neidert, 2006). These variables included age at first birth, age at first marriage, percent never married, percent of marriages that ended in divorce, the percentage of cohabiting households, non-marital fertility rate, percent unintended pregnancy, abortion rate, and family planning expenditures per woman in need of contraceptives.

We selected a comprehensive set of established sociocultural correlates to include as control variables in our analyses. Regional differences in fertility are associated with demographic, political, and religious characteristics (Lesthaeghe & Neidert, 2006). Therefore, we included a number of state-level predictors: median household income, percent African American, percent Hispanic, percent female, percent that has obtained a college degree, the percent that lives in an urban area, the percent reporting that religion is very important to them, and the percent that voted for Obama in the 2008 election, which was the election most temporally consistent with data collection.

We performed exploratory analyses to test whether state-level aggregates of the Big Five were associated with the fertility outcomes. We did not have strong *a priori* hypotheses, and we focused primarily on effect size estimation.

## Method

### Regional Estimates of Personality

We obtained regional estimates of personality from a large-scale, online study (Gosling et al., 2004). Self-reports on the Big Five Inventory (John et al., 2008) were obtained from 1999–2005 for 890,253 individuals in the United States. No statistical method was used to determine sample size, as larger samples provide more stable estimates of state-level personality. The data collection was declared exempt from informed consent by the approval of the Institutional Review Board at the University of Texas at Austin because there were no significant risks to participants (IRB number: 2004–10–0073). Responses were classified based on self-reported state of residence. Numerous measures have been taken to test the validity, representativeness, and reliability of the data. These procedures are described in several publications (Rentfrow et al., 2008; Rentfrow et al., 2009; Rentfrow et al., 2013). We controlled for the influence of response sets, such as acquiescence (i.e., yea-saying) and extreme responding (i.e., preferential use of polar response options; John et al., 2008). Additionally, we controlled for the individual-level influence of age, age^2^, gender, and an age-×-gender interaction so that demographic differences of the sample would not confound our analyses. From this individual-level data, we calculated state-level aggregates for the Big Five.

In addition, we created separate measures based on segments of each state’s population. Regional personality levels of males and females may have differential associations with fertility because of gendered divisions of labor and childrearing (McDonald, 2000). For example, regional female conscientiousness may have associations with fertility independent of male conscientiousness due to mechanisms linked to gender roles concerning childcare. At the individual-level, associations between personality and fertility differ across gender (Jokela, 2012).

Similarly, fertility rates follow a strong age-related pattern (see Figure 1). Regional personality levels of younger and older individuals may have differential associations with fertility because the older population typically has greater control over policy and institutions (Ingraham, 2014), but the younger population is responsible for most births (Martin et al., 2012). For example, regional openness of the younger population may have associations with fertility independent of the older population due to mechanisms linked to reproductive behavior.

We explored these potential driving mechanisms of personality-fertility associations. Specifically, we created state-level personality measures for male and female individuals, the younger population (age < 30) and the older population (age ≥ 30), and the difference between gendered (male personality - female personality) and aged personality (younger personality - older personality). Age 30 was selected as the cutoff to allow for roughly equal sample sizes across the groups. Thus, we calculated a total of 7 (data conditions) × 5 (Big Five) estimates of personality for each state.

### Fertility Schedule

We obtained 5-year age-specific fertility rates for each of the 50 states for 2010 (Martin et al., 2012). We transformed the 5-year age-specific fertility rates into 1-year age-specific fertility rates using the method designed by Schmertmann (2012). This method uses historical consistencies in fertility schedules to estimate the most likely 1-year age-specific fertility rates. From this, we fit Schmertmann’s (2003) calibrated spline model to the fertility schedules to provide intuitively meaningful parameters. This model uses very few parameters to construct a continuous fertility function. We focus on four aspects of the fertility schedule. First, the total fertility rate represents the average number of children that would be born to a woman if she experienced the age-specific fertility rates that prevailed in the year 2010 through her lifetime. This rate reflects the overall level of fertility in each state. Second, initiation reflects the earliest age at which fertility begins. Third, peak fertility refers to the age at which fertility is highest. Fourth, stopping refers to the force of individuals controlling maximum fertility (i.e., individuals choosing not to have additional children after a certain number). Following Schmertmann’s (2003) recommendation, stopping is calculated as the difference between the age at which fertility would linearly fall to half from peak fertility to age 50 and the actual age at which fertility reaches half of the peak. Larger stopping values indicate a steeper decline in fertility following the peak and presumably more control of fertility. The initiation, peak, and stopping parameters describe differences in the shape of the fertility schedule. Figure 1 displays eight example distributions highlighting each parameter.

### Fertility-Relevant Behaviors

We included several markers of behaviors previously found to be indicative of regional variation in fertility (Lesthaeghe & Neidert, 2006). These variables include age at first birth (Matthews & Hamilton, 2009), age at first marriage (American Community Survey [ACS]^1^), percent of the population never married (ACS), percent of marriages that ended in divorce in the last year (in reference to the total married population; ACS), the percentage of cohabiting households (Lofquist et al., 2012), non-marital fertility rate (ACS), percent unintended pregnancies (Finer & Kost, 2011), abortion rate (i.e., number of abortions per 1,000 women aged 15–44; Jones & Kooistra, 2011), and family planning expenditures per woman in need of contraceptives (Sonfield & Gold, 2012). Most indicators were obtained for the year 2010 and are based on 2010 U.S. Census estimates (Lofquist et al., 2012), the American Community Survey, the National Vital Statistics System (Matthews & Hamilton, 2009), the Pregnancy Risk Assessment Monitoring System project at the Centers for Disease Control and Prevention (Finer & Kost, 2011), an extensive census of abortion providers (Jones & Kooistra, 2011), and a survey of social service providers at the state-level (Sonfield & Gold, 2012).

### Established Correlates

We included sociodemographic variables that are established correlates of state-level fertility. These included state differences in median household income, percent African American population, percent Hispanic population, percent female population, percent of the population that has obtained a college degree, and the percent of the population that lives in an urban area based on estimates from the 2010 U.S. Census (Lofquist et al., 2012). Based on previous evidence that regional variation in fertility is associated with values (Lesthaeghe & Neidert, 2006), we additionally included the percent that voted for Obama in the 2008 election^2^ and the percent that reported that religion is very important to them in the Gallup State of the States poll^3^.

### Data Preparation

Table S1 (see in the Supplementary Materials) presents descriptive statistics for all study variables, including measures of spatial autocorrelation (i.e., geographic neighbors are more similar than expected by chance) using Moran’s I (Moran, 1950). Interpreting and addressing spatial autocorrelation is important because it indicates that empirical observations are not independent of one another and may lead to faulty statistical inferences due to violations of statistical assumptions (e.g., independent and identically distributed errors; Anselin, 1988).

We were primarily interested in the association between state-level personality and fertility, holding known correlates constant. Therefore, we computed residuals from linear models in which each of the primary study variables (i.e., personality and fertility) were regressed on the established correlates.

The online supplement and analytic report (see in the Supplementary Materials) provide full details on how we ensured that spatial autocorrelation does not bias our results. To summarize, most study variables were spatially autocorrelated across the United States and shared variance with sociodemographic characteristics and control variables. We used ordinary least squares regression to regress each study variable on the controls and saved the residuals for analysis. If the residuals still displayed spatial autocorrelation, we used spatial regression models to account for the spatial structure of the data. This was the case for six study variables. Ordinary least squares regression produced non-spatially autocorrelated residuals for the other variables. Use of standard correlation and regression techniques for the primary analyses is valid because, after residualization, no variable displayed spatial autocorrelation.

### Analytic Approach

Following the above procedure, we calculated the correlation between the aggregate personality variables and the fertility outcomes. These correlations provide a general impression of whether state-level differences in personality are associated with fertility. The state-level estimates are based on aggregates of thousands of individuals, so the mean estimates are very precise and typically produce robust associations (Rosnow et al., 2000).

The total fertility rate can be seen as the ultimate outcome of many intermediary fertility behaviors (e.g., age at first birth). Therefore, to better understand any correlations between personality and the total fertility rate, we fit mediation models. Specifically, we selected any instance in which a personality dimension correlated > |.3| with the total fertility rate and one of the other fertility outcomes. Then, we regressed the total fertility rate on the personality dimension and the other fertility outcome, and we regressed the other fertility outcome on the personality dimension. We used the lavaan package (Rosseel, 2012) to specify the model and calculated the indirect effect from personality to total fertility through the intermediary and the percent that the bivariate correlation was reduced. Standard errors were calculated through 500 bootstrap draws. Importantly, the current data do not allow for strong mediational interpretation (e.g., Cole & Maxwell, 2003), and therefore these analyses should be interpreted descriptively.

To probe whether personality factors differentially matter for fertility based on gender, we used personality aggregates derived from males and females separately. We used multiple regression to regress each fertility outcome on the estimates of male and female personality. This procedure provides an index of whether male or female personality matters more or in a different direction than personality for both sexes combined. We performed a similar approach with the two age ranges of personality, again, including both variables in a single regression. These estimates of personality tended to be correlated across gender (average *r* = .71) and age categories (average *r* = .62). Such large correlations introduce the problem of multicollinearity, which tends to inflate standard errors and can sometimes obfuscate interpretation of the regression parameters (Cohen et al., 2003).

Therefore, to complement the standard regression analysis, we also performed a commonality analysis (Nimon et al., 2008). Commonality analysis partitions variance accounted for (*R*^2^) among predictor variables into that which is unique to that predictor and that which is shared with the other predictors. This is accomplished by comparing the amount of variance accounted for in the outcome variable by all possible regression subsets. For our analysis based on subgroups, this entailed a comparison of three separate models predicting fertility. For example, the commonality analysis for gendered personality entailed estimating variance explained by male personality, by female personality, and by the multiple regression of male and female personality. This approach allows the overlapping variance to be identified and partitioned. Rather than treating multicollinearity as a problem to be fixed, this approach takes multicollinearity into account and provides reasonable estimates of an independent variable’s association at multiple levels.

Although our gender and age estimates of personality were moderately strongly correlated, they were very strongly correlated with the estimates of personality based on the full sample. The average correlation between male and female estimates of personality and the full sample estimate was .80. For estimates based on age categories, the average correlation was .73. Therefore, we interpret common effects on fertility shared across the gender or age variables to be primarily indicative of the general association found with the full sample estimates of personality. The unique predictive power of the gender or age category variables, then, represents potential personality associations with fertility that are obscured when the full data estimates of personality are used.

To test whether the influence of subgroup personality is relative to the personality of another subgroup, we calculated difference scores. For gendered personality, we calculated the difference between male and female personality with higher scores indicating that males tend to score higher on average in the region. For aged personality, we calculated the difference between the younger (< 30 years) and older (≥ 30 years) personality levels with higher scores, indicating that the younger population tends to score higher on average in the region. We used these difference scores to correlate with the fertility outcomes.

Finally, the omnibus, aggregate regional personality estimates were moderately inter-correlated (average absolute value *r* = .67). As a sensitivity analysis, we evaluated whether associations between personality traits and fertility were due to unique or common effects using commonality analysis. To accomplish this goal, we evaluated all possible regression subsets for the five predictor variables (i.e., univariate associations with fertility and every pairwise through n-wise combination of personality traits, including a multiple regression with all five traits simultaneously predicting the outcome).

### Transparency, Openness, and Reproducibility

The current study was not pre-registered. We focus on effect size estimation, rather than hypothesis testing. All analyses should be considered exploratory. Sample size was determined based on the accumulated sample at the time of analysis. No available data were excluded. The individual-level sample matches the demographics of the states well (see Rentfrow et al., 2008, p. 348). All code and output used to compile this report, as well as, data and code necessary to reproduce the analyses are included in the Supplementary Materials.

## Results

As a preliminary step, we compared the predictive power of the personality variables to that of the established sociocultural correlates before performing any residualization. We found that the five personality variables statistically account for 52% of the between-state variation in total fertility. Personality has never been implicated in geographical variation in fertility, so it is particularly striking that this percentage is nearly as large as that explained by established sociocultural predictors (*R*^2^ = .57). Moreover, a regression that includes both personality and established correlates statistically accounts for 74% of the between-state variation, indicating that both personality and established correlates account for variation in fertility uniquely of one another.

In the remainder, we report incremental associations between personality and fertility outcomes. As described in the Methods, we accomplished this by residualizing the main study variables for all established correlates. Thus, the effect sizes reported below can be considered conservative because regional personality may also exert indirect effects on fertility through values or policy (i.e., the political climate could mediate regional personality and fertility outcomes; Rentfrow et al., 2009).

### State-Level Personality-Fertility Associations

Table 1 reports correlations between personality and fertility outcomes, adjusted for all established correlates. Agreeableness, conscientiousness, neuroticism, and openness to experience substantially correlated with total fertility with large effect sizes (*r* ~ |.50|). Put differently, these effects indicate that each 1 *SD* unit difference in regional personality translates to a difference of approximately .07 children per woman in a state. Total state-average fertility across the United States ranged from 1.63 to 2.45 (*SD* = .17) in 2010, meaning that the difference associated with 1 *SD* change in personality amounts to 9% of the observed range. States with high total fertility were marked by high agreeableness and conscientiousness and low neuroticism and openness.

**Figure 2:**
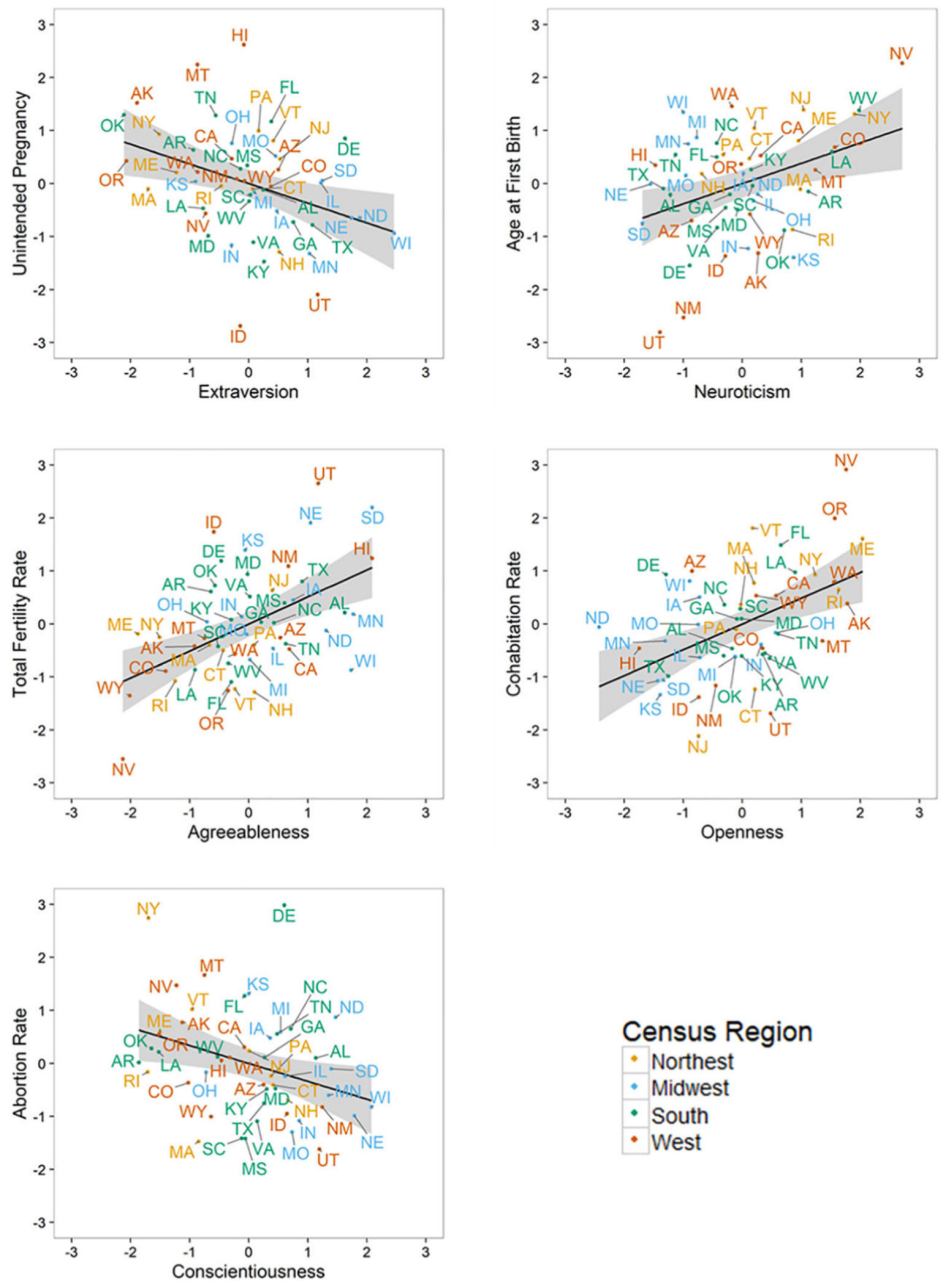
Scatterplots of Fertility Outcomes by Personality *Note*. *N* = 50 states. State-level personality was obtained from geocoded self-reports of 890,253 participants. The solid line represents the linear trend with the shaded section representing the 95% confidence interval. See Table 1 for point estimates and 95% confidence intervals. All variables are adjusted for sociodemographic characteristics and value controls and plotted as standardized residuals.

Turning toward fertility-relevant behaviors, higher state-level neuroticism was associated with later age at first birth and marriage and higher rates of cohabitation and abortion. States with higher openness tended to have higher rates of cohabitation. These moderate to large associations (*r* > .30) indicate that state-level neuroticism and openness tend to be associated with markers of nontraditional fertility, particularly in reference to delayed family formation. States with higher extraversion tended to display greater stopping behavior and lower rates of unintended pregnancy (*r* ~ |.35|). States with higher levels of agreeableness tended to have lower rates of cohabitation (*r* = −.43). States with higher levels of conscientiousness tended to display greater stopping behavior and lower rates of cohabitation, unintended pregnancy, and abortion (*r* ~ |.35|). These moderate to large associations point toward higher state-average extraversion, agreeableness, and conscientiousness as markers of traditional fertility, particularly in reference to family structures where fertility occurs. Figure 2 presents scatterplots for each personality trait and a major correlate.

### Mediation Models

The final column of Table 1 reports correlations between the total fertility rate and the other fertility outcomes. Several fertility outcomes, such as age at first birth and marriage and cohabitation, were strongly correlated with total fertility and may statistically account for the personality-total fertility association.

To explicitly test this possibility, we ran ten mediation models for sets of variables in which personality was correlated > |.3| with the fertility variables. A full description of these results can be found in the Supplementary Materials, Analytic report, Section 6.2. When age at first birth or marriage or cohabitation acted as the mediator, the personality-total fertility direct association was reduced by approximately 50% and a significant indirect effect was found. Other potential mediators (stopping, unintended pregnancy, abortion rate) minimally reduced the personality-total fertility association.

### Subgroup Analyses Based on Gender

In contrast to our aggregated personality results, subgroup analyses did not indicate a simple pattern of results. In part, the inconsistent results may stem from the relatively high collinearity of subgroup assessments of personality. For this reason, we describe general patterns of whether certain subgroups tended to have stronger associations with fertility outcomes. More information on the specific associations for each trait can be found in the Supplementary Materials.

Results of subgroup analyses based on gender are presented in Table S2 (see in the Supplementary Materials). There were relatively few unique associations between male or female personality and fertility. In total, 10 parameters associated with male personality were statistically significant (*p* < .05), compared to only 5 for female personality. Male conscientiousness appears to uniquely drive the association with cohabitation and abortion rates, with a similar result for male neuroticism and age at first marriage and the abortion rate. Female openness, rather than male openness, was associated with total fertility rate, age at first marriage, and cohabitation. Typically, the majority of variance was explained by common effects, consistent with general personality, not gender-based assessments, primarily driving associations.

### Subgroup Analyses Based on Age

Table S2 (see in the Supplementary Materials) reports subgroup analyses based on age. Several coefficients are significant, indicating that regional fertility outcomes are sensitive to levels of personality among the younger (< 30 years old) and older (≥ 30 years old) population. A total of 15 coefficients were significant for younger personality, and 17 were significant for older personality. Significant coefficients among the younger population were concentrated in extraversion, conscientiousness, and neuroticism. For example, younger, but not older, conscientiousness was associated with total fertility, cohabitation, and abortion. Openness, in contrast, was entirely driven by the older population, with significant associations for 7 of 13 outcomes. In general, states with higher openness among the older population tended to have lower fertility, delayed fertility, and more nontraditional fertility.

### Analyses Based on Relative Subgroup Differences

Results of analyses correlating age and gender differences in personality with fertility are presented in Table S2 (see in the Supplementary Materials). Gender differences were generally uncorrelated with fertility outcomes, but age differences tended to be more strongly correlated. This pattern was particularly the case for extraversion and openness, and in reference to fertility outcomes related to family formation. For example, states with higher openness among the younger population relative to the older population tended to have earlier ages at first marriage and birth, fewer never-married individuals, less cohabitation, and less non-marital fertility.

### Sensitivity Analysis: Trait Covariation

Table S2 (see in the Supplementary Materials) reports regression coefficients from regressing the fertility outcomes on the Big Five. Common variance explained relatively large portions of the association for total fertility rate and non-marital fertility rate, but other outcomes frequently displayed unique associations with personality. This result indicates that considering each of the Big Five is important for modeling fertility outcomes.

## Discussion

State-level aggregates of personality are substantially correlated with a wide range of fertility outcomes in the United States (*r* ~ |.50|). Fertility tends to be higher, earlier, and more traditional, particularly with respect to rates of cohabitation and abortion, in regions with high levels of agreeableness and conscientiousness and low levels of neuroticism and openness. Several possible mechanisms could drive such associations.

Our results are inconsistent with the hypothesis that regional-level personality-fertility associations simply and exclusively represent the aggregation of individual-level effects. For instance, individual-level studies find that conscientiousness is associated with *lower* fertility (Jokela, 2012). We find the opposite result at the region-level; conscientiousness tends to correlate with higher total fertility rates. Further, individual-level studies find that extraversion is associated with an increased likelihood of unintended pregnancies (Berg et al., 2013). We find state-level extraversion is associated with lower unintended pregnancies. Of course, mechanisms leading to individual-level and region-level phenomena do not necessarily depend on one another. Assuming that these levels of analysis must correspond is the ecological fallacy (Robinson, 1950). Some results are consistent across individuals and regions, such as the negative relation between openness and fertility (Jokela, 2012) and the negative association between conscientiousness and unintended pregnancy (Rajan et al., 2017).

Related to the ecological fallacy, the modifiable areal unit problem (Openshaw & Taylor, 1979) refers to the necessarily arbitrary selection of geographic units of aggregation to test hypotheses. For instance, in our study we drew the lines of aggregation at state borders. As in the ecological fallacy, we could find different results at both higher levels (regions, nations) and lower levels (cities, counties) of aggregation. However, using states is not completely arbitrary as many fertility-relevant policies are enacted at the state-level.

Our results are more consistent with the hypothesis that regional differences in personality influence regional policies and social norms that in turn affect individual-level fertility outcomes. In other words, states differ in terms of the social climate of fertility beliefs, public policy, and other sociodemographic predictors of fertility (Bloom et al., 2010; Demeny, 2003; Harper, 2014; Morgan, 2003). Individuals tend to create these institutions and general social contexts partially on the basis of individual differences in personality (Rentfrow et al., 2008). These societal institutions may exert top-down influences on individual-level fertility outcomes.

Fertility differentials may also influence personality concentrations. In addition to personality predicting subsequent fertility, the experience of parenthood may result in personality change (Jokela et al., 2009, but see also van Scheppingen et al., 2016 for null results and Bleidorn et al., 2018 for review). Thus, having a child or even living in a region that emphasizes childbearing may change personality levels, and therefore create a link between fertility and regional personality (Bleidorn et al., 2013).

Finally, it is of note that personality and fertility outcomes are partially heritable (Briley & Tucker-Drob, 2014; Harden, 2014), and therefore regional concentrations of these phenotypes might emerge from differential patterns of migration that persist across generations (i.e., founder effects). Historically, the spread of sociocultural influences relevant to fertility also followed migration flows (Woodard, 2011). If levels of personality in the population influence the creation of norms, genetic and sociocultural transmission could combine to produce regionally distinct fertility practices linked to personality. This logic implies that selection pressures may vary across geographical space in response to culturally created ecological niches in modern societies (e.g., Tropf et al., 2017; Whitehead et al., 2019), a phenomenon known as gene-culture coevolution. This effect may operate independently or jointly with variation associated with environmental pressures or resources. For example, regional personality differs across regions with variable ambient temperature (Wei et al., 2017), physical topography (i.e., mountainousness; Götz et al., 2020), and historical proximity to the coal industry (Obschonka et al., 2018), all of which may shape personality development or perhaps more interactively shape behavioral responses as a function of personality.

### Strengths, Limitations, and Future Directions

The current study reports novel associations between state-level personality and consequential fertility outcomes. These results have the potential to expand theoretical models of population growth and change by linking demography with personality science. As highlighted in the discussion, the causal mechanisms linking region-level personality and fertility are ambiguous. Given the complexity of influences on fertility outcomes, it is impossible to say from the current data whether or how changes in state-level personality would change fertility rates or behaviors in the future. Consistent with recent recommendations (Grosz et al., 2020), we proposed several non-mutually exclusive mechanisms that may drive the observed associations. Our view is that most of these processes likely play out to some extent. Importantly, one should not commit the ecological fallacy of assuming that individual-level and region-level associations must be consistent.

We analyzed fertility differences at one level of geographic aggregation, state-level within the United States. The modifiable areal unit problem (Openshaw & Taylor, 1979) refers to an inherent problem in geographical sciences – one’s unit of analysis can always be changed to higher (e.g., nations, rather than states) or lower (e.g., counties, rather than states) levels. Often, changing the unit of analysis changes the pattern of results. Thus, just as one should not assume that region-level associations hold at the individual level, results at the state-level may not hold at lower or higher levels of geographic aggregation.

Future work should evaluate whether the current results hold at different levels of geographic aggregation. Collecting consistently reported fertility data at lower levels of geographic aggregation can be challenging, particularly given that individuals often must travel long distances to give birth (Gjesfjeld & Jung, 2011) or access abortion services (Thompson et al., 2021), potentially distorting the geographic resolution of the data. Our results also represent an association at one point in time. Future work could examine whether similar associations are found for other years or for the trajectory of fertility patterns across years. Since fertility trends show heterogenous patterns of change (Smock & Schwartz, 2020), this approach may be particularly fruitful. Cohort fertility indices could also be used to complement the total fertility rate, which can be distorted by period influences (e.g., economic recessions).

## Conclusion

Reproductive behavior shapes the future of society. Economic and public policy decisions often rely on demographic forecasts of population growth, development, and aging based on known predictors of fertility. For the first time, we add regional personality as a strong and independent correlate of fertility. Our implementation of an extensive set of known correlates of fertility ensured that the detected personality-fertility associations were novel. Future work will be necessary to disentangle the specific mechanisms driving these new links with fertility. Theoretically, the present results highlight the dynamic interplay between socially constructed ecological niches and fertility behavior. Our findings open new avenues for research on the mechanisms of persistent geographical heterogeneity in fertility and for modeling population growth and geographical dispersion.

## Supplementary Material

Pers and Fert data

Pers and Fertility Script

Analytic report

Personality and Fertility Supplement

US Map 1

US Map 2

US Map 3

US Map 4

## Figures and Tables

**Table 1:** Correlations Between Personality and Fertility Outcomes

Fertility outcome	E [95% CI]	A [95% CI]	C [95% CI]	N [95% CI]	O [95% CI]	TFR [95% CI]
Total fertility rate	.24 [−.04, .49]	.51 [.27, .69]	.42 [.17, .63]	−.49 [−.68, −.25]	−.53 [−.70, −.30]	−
Initiation age	−.17 [−.42, .12]	.07 [−.21, .34]	−.05 [−.32, .23]	−.25 [−.49, .04]	−.04 [−.32, .24]	.25 [−.04, .49]
Peak fertility age	.20 [−.08, .46]	.19 [−.09, .45]	.21 [−.07, .46]	.01 [−.27, .29]	−.04 [−.32, .24]	−.02 [−.30, .26]
Stopping after peak fertility	.36 [.09, .58]	.09 [−.20, .36]	.36 [.09, .58]	.01 [−.27, .29]	−.22 [−.47, .06]	.19 [−.10, .44]
Age at first birth	−.03 [−.30, .25]	−.14 [−.41, .14]	−.22 [−.47, .06]	.38 [.12, .60]	.22 [−.07, .47]	−.62 [−.77, −.42]
Age at first marriage	−.16 [−.42, .12]	−.15 [−.41, .13]	−.26 [−.51, .02]	.37 [.11, .59]	.28 [.00, .52]	−.66 [−.79, −.46]
Percent never married	.04 [−.24, .32]	.20 [−.08, .45]	.00 [−.28, .28]	.04 [−.24, .32]	.04 [−.24, .32]	−.11 [−.38, .17]
Percent divorce	−.06 [−.42, .12]	−.16 [−.42, .12]	.00 [−.28, .27]	−.05 [−.33, .23]	.12 [−.16, .39]	.15 [−.14, .41]
Percent cohabit	−.27 [−.51, .01]	−.43 [−.63, −.17]	−.43 [−.63, −.17]	.31 [.03, .54]	.49 [.24, .67]	−.70 [−.82, −.52]
Non-marital fertility rate	−.10 [−.37, .19]	−.08 [−.35, .20]	−.20 [−.45, .08]	.00 [−.28, .28]	.07 [−.21, .34]	−.39 [−.60, −.12]
Percent unintended pregnancy	−.37 [−.59, −.10]	−.11 [−.38, .17]	−.44 [−.64, −.19]	.12 [−.17, .38]	.18 [−.11, .43]	−.18 [−.44, .10]
Abortion rate	−.20 [−.46, .08]	−.26 [−.50, .02]	−.34 [−.56, −.07]	.36 [.09, .58]	.19 [−.09, .45]	−.20 [−.45, .08]
Family planning expenditures	−.26 [−.50, .02]	.07 [−.21, .34]	−.18 [−.44, .10]	−.04 [−.32, .24]	.14 [−.14, .41]	−.15 [−.41, .13]

*Note*. E = Extraversion; A = Agreeableness; C = Conscientiousness; N = Neuroticism; O = Openness to experience; TFR = Total Fertility Rate. All variables adjusted for sociodemographic characteristics and value controls.

## Data Availability

The data for this article are freely available (see the Supplementary Materials section).

## Non-Technical Summary

### What is the study’s background?

People living in different regions of the U.S. make different choices about having children. In some regions, people have babies early and often, whereas in other regions, people have babies late or not at all.

### Why was this study done?

This study was done to test whether regional differences in personality were related to regional differences in fertility. Researchers in the field of demography, which studies how populations differ in birth and death rates, have typically not considered that people living in different regions may have different personalities—meaning different patterns of thoughts, feelings, and behaviors that are stable across time and context.

### What did the researchers do and find?

Researchers in this study used data on how each U.S. state differs in its average level of five basic personality traits, known as the “Big Five”. Residents of states like Wisconsin are higher on agreeableness (warmth and consideration of other people) and conscientiousness (dutifulness and planning ahead), and they show more traditional patterns of fertility. The regional differences in personality were predictive of fertility patterns even after considering differences in religiousness and politics.

### What do these findings mean?

People’s fertility behaviors are related to where they live. Part of what makes areas of the U.S. different from one another is the personality traits of the people who live there. These results have the potential to expand theoretical models of population growth and change by linking demography with personality science.

## Relevance Statement

Regional personality is associated with heterogenous fertility rates, timing, and practices across the U.S., a finding that adds a novel input to models of population growth and change.

## Key Insights

- State-level personality was associated with fertility behaviors beyond established correlates.
- Higher state-level agreeableness and conscientiousness correlated with traditional fertility behaviors.
- Higher state-level neuroticism and openness correlated with nontraditional fertility behaviors.

## Footnotes

1) Retrieved from www.census.gov/acs

2) Retrieved from http://www.fec.gov/pubrec/fe2008/federalelections2008.shtml

3) Retrieved from http://www.gallup.com/poll/125066/state-states.aspx
