# Supplementary material for: Geographic Variation in Personality is Associated With Fertility Across the United States
Source: Personal Sci. Author manuscript; Available in PMC 2024 Aug 30. (PMC11364352; doi:10.5964/ps.7275)
Supplement: Analytic report [file NIHMS1912217-supplement-Analytic_report.html]

Analytic Report for Geographic Variation in Personality is Associated with Fertility across the United States


# Analytic Report for Geographic Variation in Personality is Associated with Fertility across the United States

#### Daniel Briley

#### 10/13/2021

In this analytic report, we provide more in-depth description of our data preparation and empirical results. For all files necessary to conduct the analyses, please see our OSF page.

# 1 Dataset description

First, we load the libraries necessary to conduct the analyses. These include packages for spatial analysis (spdep and rgdal), psychometrics (psych), commonality analysis (yhat), structural equation modeling (lavaan), compiling the report (knitr), and plotting (ggplot2, ggExtra, ggrepel).

```
library(spdep)
```

```
## Loading required package: sp
```

```
## Loading required package: spData
```

```
## To access larger datasets in this package, install the spDataLarge
## package with: `install.packages('spDataLarge',
## repos='https://nowosad.github.io/drat/', type='source')`
```

```
## Loading required package: sf
```

```
## Linking to GEOS 3.8.0, GDAL 3.0.4, PROJ 6.3.1
```

```
library(rgdal)
```

```
## rgdal: version: 1.5-12, (SVN revision 1018)
## Geospatial Data Abstraction Library extensions to R successfully loaded
## Loaded GDAL runtime: GDAL 3.0.4, released 2020/01/28
## Path to GDAL shared files: C:/Users/Daniel/Documents/R/win-library/4.0/rgdal/gdal
## GDAL binary built with GEOS: TRUE 
## Loaded PROJ runtime: Rel. 6.3.1, February 10th, 2020, [PJ_VERSION: 631]
## Path to PROJ shared files: C:/Users/Daniel/Documents/R/win-library/4.0/rgdal/proj
## Linking to sp version:1.4-2
## To mute warnings of possible GDAL/OSR exportToProj4() degradation,
## use options("rgdal_show_exportToProj4_warnings"="none") before loading rgdal.
```

```
library(psych)
library(yhat)
library(lavaan)
```

```
## This is lavaan 0.6-6
```

```
## lavaan is BETA software! Please report any bugs.
```

```
## 
## Attaching package: 'lavaan'
```

```
## The following object is masked from 'package:psych':
## 
##     cor2cov
```

```
library(knitr)
library(ggplot2)
```

```
## 
## Attaching package: 'ggplot2'
```

```
## The following objects are masked from 'package:psych':
## 
##     %+%, alpha
```

```
library(ggExtra)
library(ggrepel)
```

We also read in the state-level datafile.

```
data<-read.csv("Pers and Fert Data 1.3.18.csv")
```

We can examine descriptive statistics of the dataset. The variables include: LineID and STATE\_ABBR which identify which state the row refers to. rz\_ext-rz\_opn are state-level personality means across the entire population. mzextra-mzopen are state-level personality means in reference to the male population, and fzextra-fzopen in reference to the female population. E\_LT30-O\_LT30 are state-level personality means in reference to the <30 years old population, and E\_GT30-O\_GT30 in reference to the >30 years old population. GenD\_E-GenD\_O represent to the difference between male and female state-level personality means, and AgeD\_E-AgeD\_O are similarly for the age groups. TFR is the total fertility rate. alpha is the initiation age for a state (i.e., the age at which fertility is non-zero). peak is the age at which fertility is highest within a state. stop is the extent to which fertility following peak fertility declines at a more rapid rate than would be expected based on a linear decrease. ageFB and t\_ageFM are age at first birth and age at first marriage. nevermar is the percent of individuals that never marry. divorce is percent of marriages that ended in divorce in the last year (in reference to the total married population). abortion is the number of abortions per 1000 women aged 15-44. t\_nmf is the total nonmarital fertility rate. unintprg is an estimate of the percent of pregnancies that unintended. famplnpw is the amount of funding provided per woman in need of contraceptives in each state. med\_inc is the median income. perAA/perHisp/perFem are the percentages of African American, Hispanic, and Female individuals in each state. perBA is the percent of the state population with a college degree. perUrb is the percent of the state population that lives in an urban area. voteO is the state’s vote percentage for Obama in the 2008 election. vryrel is the percent of individuals within the state that claim religion is very important to them.

```
descriptives<-describe(data)
```

```
## Warning in describe(data): NAs introduced by coercion
```

```
## Warning in FUN(newX[, i], ...): no non-missing arguments to min; returning Inf
```

```
## Warning in FUN(newX[, i], ...): no non-missing arguments to max; returning -Inf
```

```
kable(descriptives)
```

|  | vars | n | mean | sd | median | trimmed | mad | min | max | range | skew | kurtosis | se |
| --- | --- | --- | --- | --- | --- | --- | --- | --- | --- | --- | --- | --- | --- |
| LineID | 1 | 50 | 25.5000000 | 14.5773797 | 25.5000000 | 25.5000000 | 18.5325000 | 1.000000 | 50.000000 | 49.000000 | 0.0000000 | -1.2722024 | 2.0615528 |
| STATE\_ABBR\* | 2 | 50 | NaN | NA | NA | NaN | NA | Inf | -Inf | -Inf | NA | NA | NA |
| rz\_ext | 3 | 50 | -0.0012589 | 0.0350554 | 0.0016970 | -0.0004011 | 0.0316854 | -0.125762 | 0.080370 | 0.206132 | -0.6173260 | 1.6534830 | 0.0049576 |
| rz\_agr | 4 | 50 | -0.0002911 | 0.0559589 | 0.0000335 | 0.0017501 | 0.0432215 | -0.151489 | 0.114152 | 0.265641 | -0.3496762 | 0.3983994 | 0.0079138 |
| rz\_cns | 5 | 50 | -0.0002793 | 0.0495392 | 0.0045515 | -0.0000213 | 0.0544433 | -0.109966 | 0.091976 | 0.201942 | -0.0960221 | -0.9241831 | 0.0070059 |
| rz\_neu | 6 | 50 | 0.0043762 | 0.0508551 | -0.0067240 | 0.0011553 | 0.0559793 | -0.079314 | 0.136903 | 0.216217 | 0.5332872 | -0.5520376 | 0.0071920 |
| rz\_opn | 7 | 50 | -0.0194917 | 0.0621664 | -0.0215440 | -0.0198825 | 0.0783183 | -0.165218 | 0.092209 | 0.257427 | -0.0067522 | -0.9067447 | 0.0087917 |
| mzextra | 8 | 50 | 0.0914512 | 0.0299739 | 0.0924185 | 0.0919324 | 0.0274748 | -0.004242 | 0.174489 | 0.178731 | -0.2731427 | 1.1328628 | 0.0042389 |
| mzagree | 9 | 50 | 0.4723827 | 0.0255645 | 0.4728725 | 0.4740069 | 0.0164591 | 0.393204 | 0.522723 | 0.129519 | -0.6868446 | 1.1014251 | 0.0036154 |
| mzconsc | 10 | 50 | 0.3237002 | 0.0297091 | 0.3248825 | 0.3243433 | 0.0342495 | 0.262539 | 0.381757 | 0.119218 | -0.1620906 | -0.7698302 | 0.0042015 |
| mzneuro | 11 | 50 | -0.2876826 | 0.0288741 | -0.2883130 | -0.2887093 | 0.0314022 | -0.338151 | -0.216116 | 0.122035 | 0.2995277 | -0.6605218 | 0.0040834 |
| mzopen | 12 | 50 | 0.4329898 | 0.0316628 | 0.4341580 | 0.4324946 | 0.0328285 | 0.369251 | 0.511094 | 0.141843 | 0.1314487 | -0.4860494 | 0.0044778 |
| fzextra | 13 | 50 | 0.2135298 | 0.0234514 | 0.2117080 | 0.2135886 | 0.0233799 | 0.139469 | 0.264565 | 0.125096 | -0.2952398 | 0.5848203 | 0.0033165 |
| fzagree | 14 | 50 | 0.5791427 | 0.0305173 | 0.5799280 | 0.5800636 | 0.0265852 | 0.498455 | 0.641183 | 0.142728 | -0.3256127 | 0.1310393 | 0.0043158 |
| fzconsc | 15 | 50 | 0.4071533 | 0.0273830 | 0.4096465 | 0.4080221 | 0.0269047 | 0.337558 | 0.456236 | 0.118678 | -0.2709106 | -0.5301130 | 0.0038725 |
| fzneuro | 16 | 50 | 0.0230990 | 0.0347251 | 0.0172470 | 0.0209350 | 0.0399464 | -0.044549 | 0.116392 | 0.160941 | 0.4902210 | -0.3549809 | 0.0049109 |
| fzopen | 17 | 50 | 0.3559685 | 0.0365967 | 0.3511235 | 0.3556113 | 0.0383297 | 0.265262 | 0.422513 | 0.157251 | 0.0289765 | -0.6682519 | 0.0051756 |
| E\_LT30 | 18 | 50 | -0.0060348 | 0.0413786 | -0.0043575 | -0.0047589 | 0.0426196 | -0.140225 | 0.088809 | 0.229034 | -0.4896354 | 0.7847905 | 0.0058518 |
| A\_LT30 | 19 | 50 | 0.0089145 | 0.0650987 | 0.0075155 | 0.0098914 | 0.0435751 | -0.163454 | 0.148942 | 0.312396 | -0.2281651 | 0.5001142 | 0.0092063 |
| C\_LT30 | 20 | 50 | 0.0175192 | 0.0594652 | 0.0167965 | 0.0188510 | 0.0556301 | -0.142583 | 0.130334 | 0.272917 | -0.3044157 | -0.3416518 | 0.0084096 |
| N\_LT30 | 21 | 50 | 0.0004047 | 0.0526839 | -0.0069445 | -0.0027841 | 0.0587065 | -0.091282 | 0.124414 | 0.215696 | 0.4761568 | -0.7068673 | 0.0074506 |
| O\_LT30 | 22 | 50 | -0.0156524 | 0.0612774 | -0.0151465 | -0.0151143 | 0.0587362 | -0.167380 | 0.107735 | 0.275115 | -0.1275539 | -0.6522869 | 0.0086659 |
| E\_GT30 | 23 | 50 | 0.0093769 | 0.0310743 | 0.0100920 | 0.0108232 | 0.0296246 | -0.093362 | 0.065151 | 0.158513 | -0.6350462 | 0.9474117 | 0.0043946 |
| A\_GT30 | 24 | 50 | -0.0275029 | 0.0408792 | -0.0283750 | -0.0260265 | 0.0347781 | -0.138535 | 0.062225 | 0.200760 | -0.3526713 | 0.4071775 | 0.0057812 |
| C\_GT30 | 25 | 50 | -0.0492268 | 0.0415839 | -0.0464845 | -0.0487785 | 0.0415973 | -0.138512 | 0.023852 | 0.162364 | -0.0185756 | -0.7707952 | 0.0058809 |
| N\_GT30 | 26 | 50 | 0.0161027 | 0.0560015 | 0.0115790 | 0.0154878 | 0.0652729 | -0.124214 | 0.167351 | 0.291565 | 0.1198162 | -0.1952924 | 0.0079198 |
| O\_GT30 | 27 | 50 | -0.0266653 | 0.0811296 | -0.0395415 | -0.0274035 | 0.0852569 | -0.180599 | 0.129633 | 0.310232 | 0.1553325 | -0.8592534 | 0.0114735 |
| GenD\_E | 28 | 50 | -0.1220786 | 0.0176418 | -0.1217495 | -0.1214734 | 0.0174747 | -0.171483 | -0.089123 | 0.082360 | -0.3902011 | 0.0222258 | 0.0024949 |
| GenD\_A | 29 | 50 | -0.1067600 | 0.0159473 | -0.1075250 | -0.1067449 | 0.0178764 | -0.137146 | -0.070730 | 0.066416 | 0.0463070 | -0.5605585 | 0.0022553 |
| GenD\_C | 30 | 50 | -0.0834531 | 0.0230353 | -0.0850160 | -0.0837244 | 0.0213383 | -0.144157 | -0.032074 | 0.112083 | 0.0342125 | 0.0411638 | 0.0032577 |
| GenD\_N | 31 | 50 | -0.3107817 | 0.0218855 | -0.3111060 | -0.3115482 | 0.0135280 | -0.351285 | -0.227214 | 0.124071 | 0.8744158 | 2.8586611 | 0.0030951 |
| GenD\_O | 32 | 50 | 0.0770213 | 0.0241548 | 0.0784785 | 0.0776980 | 0.0214940 | 0.007724 | 0.137533 | 0.129809 | -0.2904789 | 0.5862872 | 0.0034160 |
| AgeD\_E | 33 | 50 | -0.0154117 | 0.0370710 | -0.0119160 | -0.0145160 | 0.0410769 | -0.100669 | 0.050312 | 0.150981 | -0.2476340 | -0.5344234 | 0.0052426 |
| AgeD\_A | 34 | 50 | 0.0364174 | 0.0494405 | 0.0385465 | 0.0337386 | 0.0333007 | -0.093073 | 0.166388 | 0.259461 | 0.4212693 | 1.0444077 | 0.0069919 |
| AgeD\_C | 35 | 50 | 0.0667460 | 0.0588395 | 0.0659500 | 0.0683774 | 0.0499992 | -0.105684 | 0.209985 | 0.315669 | -0.1937317 | 0.8091968 | 0.0083212 |
| AgeD\_N | 36 | 50 | -0.0156980 | 0.0399434 | -0.0124330 | -0.0152922 | 0.0369545 | -0.111473 | 0.090267 | 0.201740 | 0.0369919 | 0.4579975 | 0.0056488 |
| AgeD\_O | 37 | 50 | 0.0110129 | 0.0586769 | 0.0136255 | 0.0117065 | 0.0707749 | -0.107645 | 0.139709 | 0.247354 | -0.0265957 | -0.7351018 | 0.0082982 |
| TFR | 38 | 50 | 1.9492000 | 0.1713186 | 1.9390000 | 1.9420375 | 0.1160134 | 1.628000 | 2.449000 | 0.821000 | 0.5604791 | 0.5317421 | 0.0242281 |
| alpha | 39 | 50 | 10.5097352 | 1.6756315 | 10.7661130 | 10.6990008 | 1.6266049 | 6.058643 | 13.338400 | 7.279757 | -0.8308116 | -0.0428767 | 0.2369701 |
| peak | 40 | 50 | 26.8309940 | 2.4935804 | 26.7962855 | 26.7396512 | 3.0772905 | 22.721855 | 32.161682 | 9.439827 | 0.1885605 | -1.0366280 | 0.3526455 |
| stop | 41 | 50 | 3.8716215 | 0.8150643 | 4.0209235 | 3.9332885 | 0.8452814 | 1.282019 | 5.163898 | 3.881879 | -0.8170332 | 0.3256983 | 0.1152675 |
| ageFB | 42 | 50 | 24.8420000 | 1.1943899 | 24.6500000 | 24.7875000 | 1.1860800 | 22.600000 | 27.700000 | 5.100000 | 0.3930525 | -0.5547954 | 0.1689122 |
| t\_ageFM | 43 | 50 | 27.3110000 | 1.1296690 | 27.3250000 | 27.3425000 | 1.1119500 | 24.450000 | 29.750000 | 5.300000 | -0.2598585 | -0.0821149 | 0.1597593 |
| nevermar | 44 | 50 | 0.3070344 | 0.0284632 | 0.3095515 | 0.3063935 | 0.0284244 | 0.248992 | 0.375297 | 0.126305 | 0.1438948 | -0.4227545 | 0.0040253 |
| divorce | 45 | 50 | 0.0145996 | 0.0021740 | 0.0143845 | 0.0145656 | 0.0020638 | 0.009969 | 0.019180 | 0.009211 | 0.1022380 | -0.6811983 | 0.0003075 |
| cohabit | 46 | 50 | 6.8780000 | 1.0999610 | 6.5500000 | 6.8450000 | 1.1860800 | 4.600000 | 9.300000 | 4.700000 | 0.2575540 | -0.5983224 | 0.1555580 |
| abortion | 47 | 50 | 15.6160000 | 8.4317999 | 15.3500000 | 14.8275000 | 6.8940900 | 0.900000 | 40.000000 | 39.100000 | 0.8294242 | 0.4594493 | 1.1924366 |
| t\_nmf | 48 | 50 | 35.5460000 | 6.2819537 | 35.8500000 | 35.4125000 | 5.8562700 | 15.800000 | 51.200000 | 35.400000 | -0.0853276 | 0.8843391 | 0.8884024 |
| unintprg | 49 | 50 | 51.5600000 | 5.4964181 | 52.5000000 | 51.6750000 | 5.1891000 | 38.000000 | 65.000000 | 27.000000 | -0.1613631 | -0.3561262 | 0.7773109 |
| famplnpw | 50 | 50 | 107.4600000 | 43.7489172 | 106.0000000 | 104.9000000 | 51.8910000 | 31.000000 | 245.000000 | 214.000000 | 0.6107290 | 0.2389748 | 6.1870312 |
| med\_inc | 51 | 50 | 49755.0600000 | 8060.3641469 | 48332.5000000 | 49141.7000000 | 8435.9940000 | 36851.000000 | 68854.000000 | 32003.000000 | 0.6265369 | -0.5048382 | 1139.9076294 |
| perAA | 52 | 50 | 10.3380000 | 9.5512984 | 7.0000000 | 8.9050000 | 8.2284300 | 0.400000 | 37.000000 | 36.600000 | 1.0598117 | 0.1843740 | 1.3507576 |
| perHisp | 53 | 50 | 10.6100000 | 9.9803531 | 8.2000000 | 8.6050000 | 6.0786600 | 1.200000 | 46.300000 | 45.100000 | 1.8283967 | 3.0026216 | 1.4114351 |
| perFem | 54 | 50 | 50.6600000 | 0.7505100 | 50.7500000 | 50.7300000 | 0.6671700 | 47.900000 | 51.700000 | 3.800000 | -1.1390932 | 1.9267794 | 0.1061381 |
| perBA | 55 | 50 | 27.1600000 | 4.7641947 | 26.3500000 | 27.0300000 | 4.5219300 | 17.300000 | 38.300000 | 21.000000 | 0.2585064 | -0.5080374 | 0.6737589 |
| perUrb | 56 | 50 | 73.5818000 | 14.5645566 | 73.7350000 | 74.4960000 | 17.3315940 | 38.660000 | 94.950000 | 56.290000 | -0.4330903 | -0.5506156 | 2.0597393 |
| voteO | 57 | 50 | 50.5130000 | 9.4918922 | 51.2650000 | 50.5845000 | 10.5486990 | 32.540000 | 71.850000 | 39.310000 | 0.0068983 | -0.9318732 | 1.3423563 |
| vryrel | 58 | 50 | 39.6200000 | 8.6915864 | 39.0500000 | 39.4475000 | 9.7110300 | 23.800000 | 56.600000 | 32.800000 | 0.1795774 | -0.8436091 | 1.2291759 |

# 2 Preliminary Analysis

How does state-level personality compare to established correlates of fertility which have been the primary focus of demographic and sociological research? Does one set of variables capture more variance in the total fertility rate?

```
lm.tfr.big5<-lm(TFR~rz_ext+rz_agr+rz_cns+rz_neu+rz_opn, data=data)
summary(lm.tfr.big5)
```

```
## 
## Call:
## lm(formula = TFR ~ rz_ext + rz_agr + rz_cns + rz_neu + rz_opn, 
##     data = data)
## 
## Residuals:
##      Min       1Q   Median       3Q      Max 
## -0.18974 -0.08739 -0.02399  0.07876  0.46421 
## 
## Coefficients:
##             Estimate Std. Error t value Pr(>|t|)    
## (Intercept)  1.92875    0.01944  99.219  < 2e-16 ***
## rz_ext      -1.44067    0.89211  -1.615  0.11348    
## rz_agr      -0.75813    0.54905  -1.381  0.17432    
## rz_cns       0.08630    0.78948   0.109  0.91345    
## rz_neu      -2.24026    0.43342  -5.169 5.51e-06 ***
## rz_opn      -1.44882    0.39787  -3.641  0.00071 ***
## ---
## Signif. codes:  0 '***' 0.001 '**' 0.01 '*' 0.05 '.' 0.1 ' ' 1
## 
## Residual standard error: 0.1252 on 44 degrees of freedom
## Multiple R-squared:  0.5206, Adjusted R-squared:  0.4661 
## F-statistic: 9.556 on 5 and 44 DF,  p-value: 3.177e-06
```

```
lm.tfr.conv<-lm(TFR~med_inc+perAA+perFem+perBA+perUrb+perHisp+voteO+vryrel, data=data)
summary(lm.tfr.conv)
```

```
## 
## Call:
## lm(formula = TFR ~ med_inc + perAA + perFem + perBA + perUrb + 
##     perHisp + voteO + vryrel, data = data)
## 
## Residuals:
##      Min       1Q   Median       3Q      Max 
## -0.28531 -0.06116 -0.02127  0.06574  0.29707 
## 
## Coefficients:
##               Estimate Std. Error t value Pr(>|t|)    
## (Intercept)  1.035e+01  1.876e+00   5.518 2.10e-06 ***
## med_inc     -2.277e-06  4.829e-06  -0.472    0.640    
## perAA        2.986e-03  2.671e-03   1.118    0.270    
## perFem      -1.665e-01  3.753e-02  -4.437 6.71e-05 ***
## perBA       -4.117e-03  7.509e-03  -0.548    0.586    
## perUrb       2.590e-03  2.015e-03   1.285    0.206    
## perHisp      2.944e-04  2.458e-03   0.120    0.905    
## voteO       -1.391e-03  2.654e-03  -0.524    0.603    
## vryrel       2.621e-03  2.797e-03   0.937    0.354    
## ---
## Signif. codes:  0 '***' 0.001 '**' 0.01 '*' 0.05 '.' 0.1 ' ' 1
## 
## Residual standard error: 0.1224 on 41 degrees of freedom
## Multiple R-squared:  0.5725, Adjusted R-squared:  0.4891 
## F-statistic: 6.865 on 8 and 41 DF,  p-value: 1.072e-05
```

```
lm.tfr.all<-lm(TFR~rz_ext+rz_agr+rz_cns+rz_neu+rz_opn+med_inc+perAA+perFem+perBA+perUrb+perHisp+voteO+vryrel, data=data)
summary(lm.tfr.all)
```

```
## 
## Call:
## lm(formula = TFR ~ rz_ext + rz_agr + rz_cns + rz_neu + rz_opn + 
##     med_inc + perAA + perFem + perBA + perUrb + perHisp + voteO + 
##     vryrel, data = data)
## 
## Residuals:
##       Min        1Q    Median        3Q       Max 
## -0.141436 -0.070078 -0.001355  0.056803  0.306559 
## 
## Coefficients:
##               Estimate Std. Error t value Pr(>|t|)  
## (Intercept)  5.770e+00  3.007e+00   1.919   0.0630 .
## rz_ext      -1.394e+00  8.231e-01  -1.694   0.0989 .
## rz_agr       3.195e-01  5.465e-01   0.585   0.5624  
## rz_cns       2.757e-01  7.563e-01   0.365   0.7176  
## rz_neu      -7.818e-01  7.460e-01  -1.048   0.3016  
## rz_opn      -1.271e+00  5.328e-01  -2.386   0.0224 *
## med_inc      1.744e-06  5.221e-06   0.334   0.7403  
## perAA       -6.718e-04  3.054e-03  -0.220   0.8271  
## perFem      -8.066e-02  6.110e-02  -1.320   0.1951  
## perBA       -3.662e-03  8.898e-03  -0.412   0.6831  
## perUrb       1.732e-03  1.972e-03   0.878   0.3856  
## perHisp      4.159e-03  2.325e-03   1.789   0.0820 .
## voteO       -1.776e-03  2.375e-03  -0.748   0.4595  
## vryrel       4.563e-03  2.529e-03   1.804   0.0795 .
## ---
## Signif. codes:  0 '***' 0.001 '**' 0.01 '*' 0.05 '.' 0.1 ' ' 1
## 
## Residual standard error: 0.1017 on 36 degrees of freedom
## Multiple R-squared:  0.7413, Adjusted R-squared:  0.6479 
## F-statistic: 7.936 on 13 and 36 DF,  p-value: 3.932e-07
```

State-level personality compares surprisingly well to the set of established correlates. State-level personality captures 52% of the variance in total fertility, whereas the established correlates capture 57% of the variance. Much of this variance is unique. When both sets of variables are entered, the full set of variables captures 74% of the variance. (Note, one should be skeptical of the standard errors of these models as we have not evaluated spatial autocorrelation yet.)

# 3 Residualize for established correlates

In order to ensure that the identified associations are novel (i.e., not simply reproducing the established correlate associations as state-level personality is known to be correlated with many of the established correlates), we regress the personality and fertility variables on the established correlates and save the residuals for analysis. Because the residuals are not associated with the established correlates, associations among the residuals are not due to the established correlates.

## 3.1 Big Five

```
lm.rz_ext<-lm(rz_ext~med_inc+perAA+perFem+perBA+perUrb+perHisp+voteO+vryrel, data=data)
summary(lm.rz_ext)
```

```
## 
## Call:
## lm(formula = rz_ext ~ med_inc + perAA + perFem + perBA + perUrb + 
##     perHisp + voteO + vryrel, data = data)
## 
## Residuals:
##       Min        1Q    Median        3Q       Max 
## -0.062157 -0.019203  0.000529  0.014634  0.072398 
## 
## Coefficients:
##               Estimate Std. Error t value Pr(>|t|)  
## (Intercept) -8.440e-01  4.931e-01  -1.712   0.0945 .
## med_inc     -1.460e-06  1.269e-06  -1.150   0.2568  
## perAA       -8.124e-04  7.021e-04  -1.157   0.2539  
## perFem       1.928e-02  9.863e-03   1.954   0.0575 .
## perBA        1.066e-04  1.974e-03   0.054   0.9572  
## perUrb       5.390e-04  5.297e-04   1.018   0.3149  
## perHisp     -8.392e-04  6.460e-04  -1.299   0.2012  
## voteO       -1.112e-03  6.977e-04  -1.593   0.1188  
## vryrel      -7.651e-04  7.351e-04  -1.041   0.3041  
## ---
## Signif. codes:  0 '***' 0.001 '**' 0.01 '*' 0.05 '.' 0.1 ' ' 1
## 
## Residual standard error: 0.03218 on 41 degrees of freedom
## Multiple R-squared:  0.2947, Adjusted R-squared:  0.1571 
## F-statistic: 2.142 on 8 and 41 DF,  p-value: 0.05335
```

```
lm.rz_agr<-lm(rz_agr~med_inc+perAA+perFem+perBA+perUrb+perHisp+voteO+vryrel, data=data)
summary(lm.rz_agr)
```

```
## 
## Call:
## lm(formula = rz_agr ~ med_inc + perAA + perFem + perBA + perUrb + 
##     perHisp + voteO + vryrel, data = data)
## 
## Residuals:
##       Min        1Q    Median        3Q       Max 
## -0.103285 -0.028390 -0.002435  0.030194  0.101336 
## 
## Coefficients:
##               Estimate Std. Error t value Pr(>|t|)  
## (Intercept)  2.703e-01  8.121e-01   0.333   0.7410  
## med_inc     -4.606e-06  2.090e-06  -2.203   0.0332 *
## perAA        3.579e-04  1.156e-03   0.310   0.7585  
## perFem      -4.144e-03  1.624e-02  -0.255   0.7999  
## perBA        4.001e-03  3.250e-03   1.231   0.2254  
## perUrb       7.396e-04  8.724e-04   0.848   0.4015  
## perHisp     -2.456e-03  1.064e-03  -2.308   0.0261 *
## voteO        5.871e-04  1.149e-03   0.511   0.6121  
## vryrel      -4.677e-05  1.211e-03  -0.039   0.9694  
## ---
## Signif. codes:  0 '***' 0.001 '**' 0.01 '*' 0.05 '.' 0.1 ' ' 1
## 
## Residual standard error: 0.053 on 41 degrees of freedom
## Multiple R-squared:  0.2494, Adjusted R-squared:  0.1029 
## F-statistic: 1.702 on 8 and 41 DF,  p-value: 0.127
```

```
lm.rz_cns<-lm(rz_cns~med_inc+perAA+perFem+perBA+perUrb+perHisp+voteO+vryrel, data=data)
summary(lm.rz_cns)
```

```
## 
## Call:
## lm(formula = rz_cns ~ med_inc + perAA + perFem + perBA + perUrb + 
##     perHisp + voteO + vryrel, data = data)
## 
## Residuals:
##       Min        1Q    Median        3Q       Max 
## -0.074873 -0.029865  0.008008  0.025809  0.083713 
## 
## Coefficients:
##               Estimate Std. Error t value Pr(>|t|)   
## (Intercept)  5.144e-01  6.752e-01   0.762  0.45055   
## med_inc     -5.715e-06  1.738e-06  -3.288  0.00208 **
## perAA        3.855e-04  9.613e-04   0.401  0.69052   
## perFem      -7.663e-03  1.351e-02  -0.567  0.57355   
## perBA        5.003e-03  2.703e-03   1.851  0.07135 . 
## perUrb       1.318e-03  7.254e-04   1.818  0.07645 . 
## perHisp     -2.048e-03  8.846e-04  -2.315  0.02569 * 
## voteO       -7.621e-04  9.554e-04  -0.798  0.42966   
## vryrel      -4.730e-04  1.007e-03  -0.470  0.64091   
## ---
## Signif. codes:  0 '***' 0.001 '**' 0.01 '*' 0.05 '.' 0.1 ' ' 1
## 
## Residual standard error: 0.04407 on 41 degrees of freedom
## Multiple R-squared:  0.3378, Adjusted R-squared:  0.2086 
## F-statistic: 2.615 on 8 and 41 DF,  p-value: 0.0207
```

```
lm.rz_neu<-lm(rz_neu~med_inc+perAA+perFem+perBA+perUrb+perHisp+voteO+vryrel, data=data)
summary(lm.rz_neu)
```

```
## 
## Call:
## lm(formula = rz_neu ~ med_inc + perAA + perFem + perBA + perUrb + 
##     perHisp + voteO + vryrel, data = data)
## 
## Residuals:
##       Min        1Q    Median        3Q       Max 
## -0.063392 -0.027024 -0.000608  0.022705  0.070663 
## 
## Coefficients:
##               Estimate Std. Error t value Pr(>|t|)    
## (Intercept) -3.619e+00  5.607e-01  -6.455 9.76e-08 ***
## med_inc      5.196e-06  1.443e-06   3.600 0.000850 ***
## perAA       -2.985e-03  7.983e-04  -3.740 0.000564 ***
## perFem       7.311e-02  1.122e-02   6.519 7.91e-08 ***
## perBA       -7.846e-03  2.244e-03  -3.496 0.001150 ** 
## perUrb      -1.752e-03  6.024e-04  -2.908 0.005851 ** 
## perHisp      7.211e-04  7.346e-04   0.982 0.332036    
## voteO       -8.954e-05  7.934e-04  -0.113 0.910689    
## vryrel       7.781e-04  8.359e-04   0.931 0.357372    
## ---
## Signif. codes:  0 '***' 0.001 '**' 0.01 '*' 0.05 '.' 0.1 ' ' 1
## 
## Residual standard error: 0.0366 on 41 degrees of freedom
## Multiple R-squared:  0.5667, Adjusted R-squared:  0.4821 
## F-statistic: 6.702 on 8 and 41 DF,  p-value: 1.377e-05
```

```
lm.rz_opn<-lm(rz_opn~med_inc+perAA+perFem+perBA+perUrb+perHisp+voteO+vryrel, data=data)
summary(lm.rz_opn)
```

```
## 
## Call:
## lm(formula = rz_opn ~ med_inc + perAA + perFem + perBA + perUrb + 
##     perHisp + voteO + vryrel, data = data)
## 
## Residuals:
##       Min        1Q    Median        3Q       Max 
## -0.103753 -0.031361 -0.000801  0.023117  0.087036 
## 
## Coefficients:
##               Estimate Std. Error t value Pr(>|t|)  
## (Intercept) -2.730e-01  7.161e-01  -0.381   0.7050  
## med_inc     -8.279e-07  1.843e-06  -0.449   0.6557  
## perAA        2.295e-05  1.020e-03   0.023   0.9821  
## perFem      -1.287e-03  1.432e-02  -0.090   0.9289  
## perBA        7.156e-03  2.866e-03   2.497   0.0166 *
## perUrb       2.826e-04  7.693e-04   0.367   0.7153  
## perHisp      2.456e-03  9.382e-04   2.617   0.0124 *
## voteO        9.542e-04  1.013e-03   0.942   0.3518  
## vryrel       1.773e-03  1.068e-03   1.661   0.1043  
## ---
## Signif. codes:  0 '***' 0.001 '**' 0.01 '*' 0.05 '.' 0.1 ' ' 1
## 
## Residual standard error: 0.04674 on 41 degrees of freedom
## Multiple R-squared:  0.5271, Adjusted R-squared:  0.4348 
## F-statistic: 5.711 on 8 and 41 DF,  p-value: 6.791e-05
```

```
#save standardized residuals in data

data$Zext<-scale(residuals(lm.rz_ext))
data$Zagr<-scale(residuals(lm.rz_agr))
data$Zcns<-scale(residuals(lm.rz_cns))
data$Zneu<-scale(residuals(lm.rz_neu))
data$Zopn<-scale(residuals(lm.rz_opn))
```

## 3.2 Gendered Big Five

```
lm.mextra<-lm(mzextra~med_inc+perAA+perFem+perBA+perUrb+perHisp+voteO+vryrel, data=data)
summary(lm.mextra)
```

```
## 
## Call:
## lm(formula = mzextra ~ med_inc + perAA + perFem + perBA + perUrb + 
##     perHisp + voteO + vryrel, data = data)
## 
## Residuals:
##       Min        1Q    Median        3Q       Max 
## -0.067781 -0.015323 -0.000484  0.013710  0.071776 
## 
## Coefficients:
##               Estimate Std. Error t value Pr(>|t|)  
## (Intercept) -6.233e-01  4.182e-01  -1.490   0.1438  
## med_inc     -1.192e-06  1.077e-06  -1.108   0.2745  
## perAA       -1.025e-03  5.955e-04  -1.721   0.0928 .
## perFem       1.636e-02  8.366e-03   1.956   0.0573 .
## perBA       -7.149e-05  1.674e-03  -0.043   0.9661  
## perUrb       6.685e-04  4.493e-04   1.488   0.1445  
## perHisp     -9.436e-04  5.480e-04  -1.722   0.0926 .
## voteO       -1.131e-03  5.918e-04  -1.910   0.0631 .
## vryrel      -6.152e-04  6.235e-04  -0.987   0.3296  
## ---
## Signif. codes:  0 '***' 0.001 '**' 0.01 '*' 0.05 '.' 0.1 ' ' 1
## 
## Residual standard error: 0.0273 on 41 degrees of freedom
## Multiple R-squared:  0.306,  Adjusted R-squared:  0.1705 
## F-statistic: 2.259 on 8 and 41 DF,  p-value: 0.04214
```

```
lm.magree<-lm(mzagree~med_inc+perAA+perFem+perBA+perUrb+perHisp+voteO+vryrel, data=data)
summary(lm.magree)
```

```
## 
## Call:
## lm(formula = mzagree ~ med_inc + perAA + perFem + perBA + perUrb + 
##     perHisp + voteO + vryrel, data = data)
## 
## Residuals:
##       Min        1Q    Median        3Q       Max 
## -0.050381 -0.012152 -0.000819  0.009874  0.050981 
## 
## Coefficients:
##               Estimate Std. Error t value Pr(>|t|)  
## (Intercept)  2.937e-01  3.750e-01   0.783   0.4381  
## med_inc     -1.996e-06  9.654e-07  -2.068   0.0450 *
## perAA       -8.339e-05  5.340e-04  -0.156   0.8767  
## perFem       3.571e-03  7.502e-03   0.476   0.6366  
## perBA        1.663e-03  1.501e-03   1.108   0.2745  
## perUrb       4.725e-04  4.029e-04   1.173   0.2477  
## perHisp     -1.021e-03  4.914e-04  -2.079   0.0439 *
## voteO        3.546e-04  5.307e-04   0.668   0.5077  
## vryrel       2.779e-04  5.591e-04   0.497   0.6219  
## ---
## Signif. codes:  0 '***' 0.001 '**' 0.01 '*' 0.05 '.' 0.1 ' ' 1
## 
## Residual standard error: 0.02448 on 41 degrees of freedom
## Multiple R-squared:  0.2328, Adjusted R-squared:  0.08312 
## F-statistic: 1.555 on 8 and 41 DF,  p-value: 0.1686
```

```
lm.mconsc<-lm(mzconsc~med_inc+perAA+perFem+perBA+perUrb+perHisp+voteO+vryrel, data=data)
summary(lm.mconsc)
```

```
## 
## Call:
## lm(formula = mzconsc ~ med_inc + perAA + perFem + perBA + perUrb + 
##     perHisp + voteO + vryrel, data = data)
## 
## Residuals:
##       Min        1Q    Median        3Q       Max 
## -0.047398 -0.010597 -0.000142  0.012602  0.060899 
## 
## Coefficients:
##               Estimate Std. Error t value Pr(>|t|)    
## (Intercept)  9.386e-01  3.510e-01   2.674 0.010712 *  
## med_inc     -3.403e-06  9.035e-07  -3.766 0.000521 ***
## perAA        8.616e-04  4.998e-04   1.724 0.092246 .  
## perFem      -1.090e-02  7.021e-03  -1.552 0.128327    
## perBA        1.489e-03  1.405e-03   1.059 0.295589    
## perUrb       6.337e-04  3.771e-04   1.680 0.100470    
## perHisp     -3.395e-04  4.599e-04  -0.738 0.464579    
## voteO       -3.355e-05  4.966e-04  -0.068 0.946473    
## vryrel       3.984e-04  5.233e-04   0.761 0.450767    
## ---
## Signif. codes:  0 '***' 0.001 '**' 0.01 '*' 0.05 '.' 0.1 ' ' 1
## 
## Residual standard error: 0.02291 on 41 degrees of freedom
## Multiple R-squared:  0.5024, Adjusted R-squared:  0.4053 
## F-statistic: 5.175 on 8 and 41 DF,  p-value: 0.0001688
```

```
lm.mneuro<-lm(mzneuro~med_inc+perAA+perFem+perBA+perUrb+perHisp+voteO+vryrel, data=data)
summary(lm.mneuro)
```

```
## 
## Call:
## lm(formula = mzneuro ~ med_inc + perAA + perFem + perBA + perUrb + 
##     perHisp + voteO + vryrel, data = data)
## 
## Residuals:
##      Min       1Q   Median       3Q      Max 
## -0.03838 -0.01347 -0.00243  0.01559  0.05505 
## 
## Coefficients:
##               Estimate Std. Error t value Pr(>|t|)    
## (Intercept) -2.281e+00  3.506e-01  -6.507 8.22e-08 ***
## med_inc      2.828e-06  9.025e-07   3.134  0.00318 ** 
## perAA       -1.640e-03  4.992e-04  -3.285  0.00209 ** 
## perFem       3.923e-02  7.013e-03   5.593 1.64e-06 ***
## perBA       -4.008e-03  1.403e-03  -2.856  0.00671 ** 
## perUrb      -6.209e-04  3.767e-04  -1.648  0.10693    
## perHisp      4.054e-04  4.594e-04   0.882  0.38265    
## voteO        2.119e-04  4.961e-04   0.427  0.67144    
## vryrel       5.632e-04  5.227e-04   1.077  0.28760    
## ---
## Signif. codes:  0 '***' 0.001 '**' 0.01 '*' 0.05 '.' 0.1 ' ' 1
## 
## Residual standard error: 0.02288 on 41 degrees of freedom
## Multiple R-squared:  0.4744, Adjusted R-squared:  0.3719 
## F-statistic: 4.626 on 8 and 41 DF,  p-value: 0.000444
```

```
lm.mopen<-lm(mzopen~med_inc+perAA+perFem+perBA+perUrb+perHisp+voteO+vryrel, data=data)
summary(lm.mopen)
```

```
## 
## Call:
## lm(formula = mzopen ~ med_inc + perAA + perFem + perBA + perUrb + 
##     perHisp + voteO + vryrel, data = data)
## 
## Residuals:
##       Min        1Q    Median        3Q       Max 
## -0.047924 -0.017925  0.003943  0.012904  0.037995 
## 
## Coefficients:
##               Estimate Std. Error t value Pr(>|t|)    
## (Intercept)  4.854e-01  3.619e-01   1.341 0.187248    
## med_inc     -1.272e-06  9.317e-07  -1.365 0.179756    
## perAA        4.990e-04  5.153e-04   0.968 0.338573    
## perFem      -2.912e-03  7.240e-03  -0.402 0.689594    
## perBA        3.667e-03  1.449e-03   2.531 0.015290 *  
## perUrb      -8.049e-05  3.888e-04  -0.207 0.837042    
## perHisp      2.002e-03  4.742e-04   4.221 0.000131 ***
## voteO        2.669e-04  5.121e-04   0.521 0.605078    
## vryrel       6.257e-04  5.396e-04   1.160 0.252913    
## ---
## Signif. codes:  0 '***' 0.001 '**' 0.01 '*' 0.05 '.' 0.1 ' ' 1
## 
## Residual standard error: 0.02362 on 41 degrees of freedom
## Multiple R-squared:  0.5342, Adjusted R-squared:  0.4433 
## F-statistic: 5.878 on 8 and 41 DF,  p-value: 5.156e-05
```

```
lm.fextra<-lm(fzextra~med_inc+perAA+perFem+perBA+perUrb+perHisp+voteO+vryrel, data=data)
summary(lm.fextra)
```

```
## 
## Call:
## lm(formula = fzextra ~ med_inc + perAA + perFem + perBA + perUrb + 
##     perHisp + voteO + vryrel, data = data)
## 
## Residuals:
##       Min        1Q    Median        3Q       Max 
## -0.039807 -0.011537 -0.000513  0.012962  0.045004 
## 
## Coefficients:
##               Estimate Std. Error t value Pr(>|t|)
## (Intercept) -2.912e-01  3.445e-01  -0.846    0.403
## med_inc     -4.908e-07  8.866e-07  -0.553    0.583
## perAA       -3.928e-04  4.904e-04  -0.801    0.428
## perFem       1.150e-02  6.890e-03   1.669    0.103
## perBA       -9.866e-05  1.379e-03  -0.072    0.943
## perUrb       3.354e-04  3.700e-04   0.906    0.370
## perHisp     -6.648e-04  4.513e-04  -1.473    0.148
## voteO       -7.277e-04  4.874e-04  -1.493    0.143
## vryrel      -6.909e-04  5.135e-04  -1.345    0.186
## 
## Residual standard error: 0.02248 on 41 degrees of freedom
## Multiple R-squared:  0.231,  Adjusted R-squared:  0.08096 
## F-statistic:  1.54 on 8 and 41 DF,  p-value: 0.1737
```

```
lm.fagree<-lm(fzagree~med_inc+perAA+perFem+perBA+perUrb+perHisp+voteO+vryrel, data=data)
summary(lm.fagree)
```

```
## 
## Call:
## lm(formula = fzagree ~ med_inc + perAA + perFem + perBA + perUrb + 
##     perHisp + voteO + vryrel, data = data)
## 
## Residuals:
##       Min        1Q    Median        3Q       Max 
## -0.056846 -0.014894  0.000685  0.014867  0.056656 
## 
## Coefficients:
##               Estimate Std. Error t value Pr(>|t|)  
## (Intercept)  9.824e-01  4.340e-01   2.264   0.0289 *
## med_inc     -2.810e-06  1.117e-06  -2.516   0.0159 *
## perAA        4.728e-04  6.179e-04   0.765   0.4485  
## perFem      -7.120e-03  8.680e-03  -0.820   0.4168  
## perBA        2.446e-03  1.737e-03   1.408   0.1667  
## perUrb       1.746e-04  4.662e-04   0.374   0.7100  
## perHisp     -1.110e-03  5.686e-04  -1.952   0.0578 .
## voteO        4.924e-04  6.140e-04   0.802   0.4272  
## vryrel       1.166e-07  6.470e-04   0.000   0.9999  
## ---
## Signif. codes:  0 '***' 0.001 '**' 0.01 '*' 0.05 '.' 0.1 ' ' 1
## 
## Residual standard error: 0.02832 on 41 degrees of freedom
## Multiple R-squared:  0.2792, Adjusted R-squared:  0.1386 
## F-statistic: 1.985 on 8 and 41 DF,  p-value: 0.07283
```

```
lm.fconsc<-lm(fzconsc~med_inc+perAA+perFem+perBA+perUrb+perHisp+voteO+vryrel, data=data)
summary(lm.fconsc)
```

```
## 
## Call:
## lm(formula = fzconsc ~ med_inc + perAA + perFem + perBA + perUrb + 
##     perHisp + voteO + vryrel, data = data)
## 
## Residuals:
##       Min        1Q    Median        3Q       Max 
## -0.046491 -0.015474  0.000427  0.017088  0.039957 
## 
## Coefficients:
##               Estimate Std. Error t value Pr(>|t|)    
## (Intercept)  7.017e-01  3.606e-01   1.946 0.058531 .  
## med_inc     -3.808e-06  9.281e-07  -4.103 0.000189 ***
## perAA        3.378e-04  5.134e-04   0.658 0.514190    
## perFem      -4.848e-03  7.212e-03  -0.672 0.505197    
## perBA        4.064e-03  1.443e-03   2.816 0.007443 ** 
## perUrb       2.371e-04  3.874e-04   0.612 0.543848    
## perHisp     -4.367e-04  4.724e-04  -0.924 0.360637    
## voteO        1.540e-04  5.102e-04   0.302 0.764351    
## vryrel       1.545e-04  5.375e-04   0.287 0.775227    
## ---
## Signif. codes:  0 '***' 0.001 '**' 0.01 '*' 0.05 '.' 0.1 ' ' 1
## 
## Residual standard error: 0.02353 on 41 degrees of freedom
## Multiple R-squared:  0.382,  Adjusted R-squared:  0.2614 
## F-statistic: 3.167 on 8 and 41 DF,  p-value: 0.006919
```

```
lm.fneuro<-lm(fzneuro~med_inc+perAA+perFem+perBA+perUrb+perHisp+voteO+vryrel, data=data)
summary(lm.fneuro)
```

```
## 
## Call:
## lm(formula = fzneuro ~ med_inc + perAA + perFem + perBA + perUrb + 
##     perHisp + voteO + vryrel, data = data)
## 
## Residuals:
##       Min        1Q    Median        3Q       Max 
## -0.051389 -0.019154  0.000691  0.014079  0.041352 
## 
## Coefficients:
##               Estimate Std. Error t value Pr(>|t|)    
## (Intercept) -2.416e+00  3.746e-01  -6.450 9.90e-08 ***
## med_inc      3.488e-06  9.643e-07   3.617 0.000809 ***
## perAA       -2.055e-03  5.334e-04  -3.852 0.000404 ***
## perFem       4.972e-02  7.493e-03   6.635 5.41e-08 ***
## perBA       -5.399e-03  1.499e-03  -3.601 0.000849 ***
## perUrb      -1.160e-03  4.024e-04  -2.882 0.006262 ** 
## perHisp      2.658e-04  4.908e-04   0.542 0.591005    
## voteO       -3.257e-04  5.301e-04  -0.614 0.542334    
## vryrel       3.530e-04  5.585e-04   0.632 0.530847    
## ---
## Signif. codes:  0 '***' 0.001 '**' 0.01 '*' 0.05 '.' 0.1 ' ' 1
## 
## Residual standard error: 0.02445 on 41 degrees of freedom
## Multiple R-squared:  0.5852, Adjusted R-squared:  0.5042 
## F-statistic: 7.229 on 8 and 41 DF,  p-value: 6.159e-06
```

```
lm.fopen<-lm(fzopen~med_inc+perAA+perFem+perBA+perUrb+perHisp+voteO+vryrel, data=data)
summary(lm.fopen)
```

```
## 
## Call:
## lm(formula = fzopen ~ med_inc + perAA + perFem + perBA + perUrb + 
##     perHisp + voteO + vryrel, data = data)
## 
## Residuals:
##       Min        1Q    Median        3Q       Max 
## -0.069730 -0.017070 -0.000078  0.010848  0.058696 
## 
## Coefficients:
##               Estimate Std. Error t value Pr(>|t|)  
## (Intercept)  1.669e-01  4.417e-01   0.378   0.7075  
## med_inc     -6.953e-08  1.137e-06  -0.061   0.9515  
## perAA       -1.630e-04  6.288e-04  -0.259   0.7967  
## perFem      -3.466e-04  8.834e-03  -0.039   0.9689  
## perBA        3.702e-03  1.768e-03   2.094   0.0425 *
## perUrb       1.609e-04  4.745e-04   0.339   0.7362  
## perHisp      1.085e-03  5.787e-04   1.875   0.0679 .
## voteO        7.908e-04  6.249e-04   1.265   0.2128  
## vryrel       1.210e-03  6.584e-04   1.838   0.0734 .
## ---
## Signif. codes:  0 '***' 0.001 '**' 0.01 '*' 0.05 '.' 0.1 ' ' 1
## 
## Residual standard error: 0.02883 on 41 degrees of freedom
## Multiple R-squared:  0.4808, Adjusted R-squared:  0.3795 
## F-statistic: 4.747 on 8 and 41 DF,  p-value: 0.0003581
```

```
data$Zmext<-scale(residuals(lm.mextra))
data$Zmagr<-scale(residuals(lm.magree))
data$Zmcns<-scale(residuals(lm.mconsc))
data$Zmneu<-scale(residuals(lm.mneuro))
data$Zmopn<-scale(residuals(lm.mopen))

data$Zfext<-scale(residuals(lm.fextra))
data$Zfagr<-scale(residuals(lm.fagree))
data$Zfcns<-scale(residuals(lm.fconsc))
data$Zfneu<-scale(residuals(lm.fneuro))
data$Zfopn<-scale(residuals(lm.fopen))
```

## 3.3 Age Big Five

```
lm.E_LT30<-lm(E_LT30~med_inc+perAA+perFem+perBA+perUrb+perHisp+voteO+vryrel, data=data)
summary(lm.E_LT30)
```

```
## 
## Call:
## lm(formula = E_LT30 ~ med_inc + perAA + perFem + perBA + perUrb + 
##     perHisp + voteO + vryrel, data = data)
## 
## Residuals:
##       Min        1Q    Median        3Q       Max 
## -0.077898 -0.024119  0.001588  0.017478  0.079580 
## 
## Coefficients:
##               Estimate Std. Error t value Pr(>|t|)  
## (Intercept) -8.953e-01  5.502e-01  -1.627   0.1114  
## med_inc     -1.874e-06  1.416e-06  -1.323   0.1931  
## perAA       -1.115e-03  7.834e-04  -1.423   0.1624  
## perFem       2.113e-02  1.101e-02   1.920   0.0619 .
## perBA       -2.585e-04  2.202e-03  -0.117   0.9071  
## perUrb       8.902e-04  5.911e-04   1.506   0.1397  
## perHisp     -1.621e-03  7.209e-04  -2.248   0.0300 *
## voteO       -1.423e-03  7.785e-04  -1.828   0.0749 .
## vryrel      -1.152e-03  8.203e-04  -1.405   0.1676  
## ---
## Signif. codes:  0 '***' 0.001 '**' 0.01 '*' 0.05 '.' 0.1 ' ' 1
## 
## Residual standard error: 0.03591 on 41 degrees of freedom
## Multiple R-squared:  0.3697, Adjusted R-squared:  0.2468 
## F-statistic: 3.007 on 8 and 41 DF,  p-value: 0.009491
```

```
lm.A_LT30<-lm(A_LT30~med_inc+perAA+perFem+perBA+perUrb+perHisp+voteO+vryrel, data=data)
summary(lm.A_LT30)
```

```
## 
## Call:
## lm(formula = A_LT30 ~ med_inc + perAA + perFem + perBA + perUrb + 
##     perHisp + voteO + vryrel, data = data)
## 
## Residuals:
##      Min       1Q   Median       3Q      Max 
## -0.11748 -0.03369 -0.00563  0.03210  0.13393 
## 
## Coefficients:
##               Estimate Std. Error t value Pr(>|t|)  
## (Intercept)  1.454e-01  9.415e-01   0.154   0.8780  
## med_inc     -5.366e-06  2.423e-06  -2.214   0.0324 *
## perAA        3.357e-05  1.340e-03   0.025   0.9801  
## perFem      -6.307e-04  1.883e-02  -0.033   0.9734  
## perBA        3.927e-03  3.768e-03   1.042   0.3035  
## perUrb       1.077e-03  1.011e-03   1.064   0.2934  
## perHisp     -2.844e-03  1.233e-03  -2.305   0.0263 *
## voteO        4.617e-04  1.332e-03   0.347   0.7307  
## vryrel      -4.282e-04  1.404e-03  -0.305   0.7619  
## ---
## Signif. codes:  0 '***' 0.001 '**' 0.01 '*' 0.05 '.' 0.1 ' ' 1
## 
## Residual standard error: 0.06145 on 41 degrees of freedom
## Multiple R-squared:  0.2545, Adjusted R-squared:  0.109 
## F-statistic: 1.749 on 8 and 41 DF,  p-value: 0.116
```

```
lm.C_LT30<-lm(C_LT30~med_inc+perAA+perFem+perBA+perUrb+perHisp+voteO+vryrel, data=data)
summary(lm.C_LT30)
```

```
## 
## Call:
## lm(formula = C_LT30 ~ med_inc + perAA + perFem + perBA + perUrb + 
##     perHisp + voteO + vryrel, data = data)
## 
## Residuals:
##       Min        1Q    Median        3Q       Max 
## -0.093949 -0.035499  0.008473  0.027404  0.084146 
## 
## Coefficients:
##               Estimate Std. Error t value Pr(>|t|)    
## (Intercept)  4.715e-01  7.615e-01   0.619 0.539200    
## med_inc     -7.418e-06  1.960e-06  -3.785 0.000494 ***
## perAA        2.118e-04  1.084e-03   0.195 0.846049    
## perFem      -4.845e-03  1.523e-02  -0.318 0.752053    
## perBA        6.759e-03  3.048e-03   2.217 0.032196 *  
## perUrb       1.176e-03  8.181e-04   1.437 0.158321    
## perHisp     -2.194e-03  9.977e-04  -2.199 0.033588 *  
## voteO       -1.024e-03  1.077e-03  -0.951 0.347268    
## vryrel      -9.265e-04  1.135e-03  -0.816 0.419155    
## ---
## Signif. codes:  0 '***' 0.001 '**' 0.01 '*' 0.05 '.' 0.1 ' ' 1
## 
## Residual standard error: 0.0497 on 41 degrees of freedom
## Multiple R-squared:  0.4155, Adjusted R-squared:  0.3014 
## F-statistic: 3.643 on 8 and 41 DF,  p-value: 0.002754
```

```
lm.N_LT30<-lm(N_LT30~med_inc+perAA+perFem+perBA+perUrb+perHisp+voteO+vryrel, data=data)
summary(lm.N_LT30)
```

```
## 
## Call:
## lm(formula = N_LT30 ~ med_inc + perAA + perFem + perBA + perUrb + 
##     perHisp + voteO + vryrel, data = data)
## 
## Residuals:
##       Min        1Q    Median        3Q       Max 
## -0.074480 -0.025832 -0.003847  0.025847  0.061628 
## 
## Coefficients:
##               Estimate Std. Error t value Pr(>|t|)    
## (Intercept) -3.839e+00  5.671e-01  -6.770 3.48e-08 ***
## med_inc      5.558e-06  1.460e-06   3.807 0.000461 ***
## perAA       -2.889e-03  8.074e-04  -3.578 0.000905 ***
## perFem       7.629e-02  1.134e-02   6.725 4.04e-08 ***
## perBA       -7.400e-03  2.270e-03  -3.260 0.002247 ** 
## perUrb      -2.027e-03  6.093e-04  -3.328 0.001857 ** 
## perHisp      9.671e-04  7.430e-04   1.302 0.200330    
## voteO        3.229e-04  8.024e-04   0.402 0.689501    
## vryrel       1.313e-03  8.455e-04   1.552 0.128231    
## ---
## Signif. codes:  0 '***' 0.001 '**' 0.01 '*' 0.05 '.' 0.1 ' ' 1
## 
## Residual standard error: 0.03702 on 41 degrees of freedom
## Multiple R-squared:  0.587,  Adjusted R-squared:  0.5064 
## F-statistic: 7.283 on 8 and 41 DF,  p-value: 5.682e-06
```

```
lm.O_LT30<-lm(O_LT30~med_inc+perAA+perFem+perBA+perUrb+perHisp+voteO+vryrel, data=data)
summary(lm.O_LT30)
```

```
## 
## Call:
## lm(formula = O_LT30 ~ med_inc + perAA + perFem + perBA + perUrb + 
##     perHisp + voteO + vryrel, data = data)
## 
## Residuals:
##       Min        1Q    Median        3Q       Max 
## -0.108932 -0.029627  0.003709  0.029081  0.078865 
## 
## Coefficients:
##               Estimate Std. Error t value Pr(>|t|)  
## (Intercept)  2.371e-01  7.511e-01   0.316   0.7539  
## med_inc     -1.657e-06  1.933e-06  -0.857   0.3964  
## perAA        8.565e-04  1.069e-03   0.801   0.4278  
## perFem      -1.063e-02  1.502e-02  -0.708   0.4831  
## perBA        6.164e-03  3.006e-03   2.050   0.0468 *
## perUrb       4.928e-04  8.069e-04   0.611   0.5448  
## perHisp      2.594e-03  9.840e-04   2.636   0.0118 *
## voteO        9.946e-04  1.063e-03   0.936   0.3548  
## vryrel       1.969e-03  1.120e-03   1.759   0.0861 .
## ---
## Signif. codes:  0 '***' 0.001 '**' 0.01 '*' 0.05 '.' 0.1 ' ' 1
## 
## Residual standard error: 0.04902 on 41 degrees of freedom
## Multiple R-squared:  0.4645, Adjusted R-squared:   0.36 
## F-statistic: 4.446 on 8 and 41 DF,  p-value: 0.0006152
```

```
lm.E_GT30<-lm(E_GT30~med_inc+perAA+perFem+perBA+perUrb+perHisp+voteO+vryrel, data=data)
summary(lm.E_GT30)
```

```
## 
## Call:
## lm(formula = E_GT30 ~ med_inc + perAA + perFem + perBA + perUrb + 
##     perHisp + voteO + vryrel, data = data)
## 
## Residuals:
##       Min        1Q    Median        3Q       Max 
## -0.064274 -0.021670 -0.000183  0.015147  0.065727 
## 
## Coefficients:
##               Estimate Std. Error t value Pr(>|t|)  
## (Intercept) -8.159e-01  4.664e-01  -1.749   0.0877 .
## med_inc     -2.932e-07  1.201e-06  -0.244   0.8083  
## perAA       -9.436e-05  6.641e-04  -0.142   0.8877  
## perFem       1.627e-02  9.330e-03   1.744   0.0887 .
## perBA        1.119e-03  1.867e-03   0.599   0.5524  
## perUrb      -2.961e-04  5.011e-04  -0.591   0.5578  
## perHisp      1.002e-03  6.111e-04   1.639   0.1088  
## voteO       -3.005e-04  6.600e-04  -0.455   0.6512  
## vryrel       3.203e-04  6.954e-04   0.461   0.6475  
## ---
## Signif. codes:  0 '***' 0.001 '**' 0.01 '*' 0.05 '.' 0.1 ' ' 1
## 
## Residual standard error: 0.03044 on 41 degrees of freedom
## Multiple R-squared:  0.1969, Adjusted R-squared:  0.04017 
## F-statistic: 1.256 on 8 and 41 DF,  p-value: 0.2925
```

```
lm.A_GT30<-lm(A_GT30~med_inc+perAA+perFem+perBA+perUrb+perHisp+voteO+vryrel, data=data)
summary(lm.A_GT30)
```

```
## 
## Call:
## lm(formula = A_GT30 ~ med_inc + perAA + perFem + perBA + perUrb + 
##     perHisp + voteO + vryrel, data = data)
## 
## Residuals:
##       Min        1Q    Median        3Q       Max 
## -0.074514 -0.020921  0.004182  0.017471  0.100983 
## 
## Coefficients:
##               Estimate Std. Error t value Pr(>|t|)  
## (Intercept)  5.520e-01  5.965e-01   0.925   0.3602  
## med_inc     -2.892e-06  1.535e-06  -1.884   0.0667 .
## perAA        1.233e-03  8.493e-04   1.452   0.1540  
## perFem      -1.269e-02  1.193e-02  -1.063   0.2938  
## perBA        4.497e-03  2.388e-03   1.883   0.0667 .
## perUrb      -1.870e-04  6.408e-04  -0.292   0.7718  
## perHisp     -1.253e-03  7.815e-04  -1.604   0.1165  
## voteO        1.089e-03  8.440e-04   1.291   0.2040  
## vryrel       1.118e-03  8.893e-04   1.257   0.2159  
## ---
## Signif. codes:  0 '***' 0.001 '**' 0.01 '*' 0.05 '.' 0.1 ' ' 1
## 
## Residual standard error: 0.03893 on 41 degrees of freedom
## Multiple R-squared:  0.2411, Adjusted R-squared:  0.09299 
## F-statistic: 1.628 on 8 and 41 DF,  p-value: 0.1467
```

```
lm.C_GT30<-lm(C_GT30~med_inc+perAA+perFem+perBA+perUrb+perHisp+voteO+vryrel, data=data)
summary(lm.C_GT30)
```

```
## 
## Call:
## lm(formula = C_GT30 ~ med_inc + perAA + perFem + perBA + perUrb + 
##     perHisp + voteO + vryrel, data = data)
## 
## Residuals:
##       Min        1Q    Median        3Q       Max 
## -0.082965 -0.019898 -0.001642  0.025650  0.086251 
## 
## Coefficients:
##               Estimate Std. Error t value Pr(>|t|)  
## (Intercept)  4.302e-01  6.217e-01   0.692   0.4928  
## med_inc     -1.397e-06  1.600e-06  -0.873   0.3877  
## perAA        8.336e-04  8.851e-04   0.942   0.3518  
## perFem      -1.155e-02  1.244e-02  -0.929   0.3585  
## perBA        5.307e-04  2.488e-03   0.213   0.8322  
## perUrb       1.407e-03  6.679e-04   2.106   0.0413 *
## perHisp     -1.130e-03  8.145e-04  -1.387   0.1729  
## voteO        3.239e-04  8.796e-04   0.368   0.7146  
## vryrel       1.115e-03  9.268e-04   1.203   0.2360  
## ---
## Signif. codes:  0 '***' 0.001 '**' 0.01 '*' 0.05 '.' 0.1 ' ' 1
## 
## Residual standard error: 0.04058 on 41 degrees of freedom
## Multiple R-squared:  0.2033, Adjusted R-squared:  0.0479 
## F-statistic: 1.308 on 8 and 41 DF,  p-value: 0.2667
```

```
lm.N_GT30<-lm(N_GT30~med_inc+perAA+perFem+perBA+perUrb+perHisp+voteO+vryrel, data=data)
summary(lm.N_GT30)
```

```
## 
## Call:
## lm(formula = N_GT30 ~ med_inc + perAA + perFem + perBA + perUrb + 
##     perHisp + voteO + vryrel, data = data)
## 
## Residuals:
##       Min        1Q    Median        3Q       Max 
## -0.109869 -0.031134 -0.001824  0.028360  0.087174 
## 
## Coefficients:
##               Estimate Std. Error t value Pr(>|t|)    
## (Intercept) -3.058e+00  6.940e-01  -4.407 7.38e-05 ***
## med_inc      4.329e-06  1.787e-06   2.423  0.01989 *  
## perAA       -3.319e-03  9.882e-04  -3.359  0.00170 ** 
## perFem       6.550e-02  1.388e-02   4.718 2.76e-05 ***
## perBA       -8.932e-03  2.778e-03  -3.215  0.00254 ** 
## perUrb      -1.075e-03  7.456e-04  -1.442  0.15682    
## perHisp      2.618e-05  9.093e-04   0.029  0.97717    
## voteO       -1.472e-03  9.820e-04  -1.499  0.14155    
## vryrel      -7.348e-04  1.035e-03  -0.710  0.48163    
## ---
## Signif. codes:  0 '***' 0.001 '**' 0.01 '*' 0.05 '.' 0.1 ' ' 1
## 
## Residual standard error: 0.0453 on 41 degrees of freedom
## Multiple R-squared:  0.4525, Adjusted R-squared:  0.3457 
## F-statistic: 4.236 on 8 and 41 DF,  p-value: 0.0009035
```

```
lm.O_GT30<-lm(O_GT30~med_inc+perAA+perFem+perBA+perUrb+perHisp+voteO+vryrel, data=data)
summary(lm.O_GT30)
```

```
## 
## Call:
## lm(formula = O_GT30 ~ med_inc + perAA + perFem + perBA + perUrb + 
##     perHisp + voteO + vryrel, data = data)
## 
## Residuals:
##       Min        1Q    Median        3Q       Max 
## -0.094195 -0.034145 -0.008307  0.028653  0.096797 
## 
## Coefficients:
##               Estimate Std. Error t value Pr(>|t|)   
## (Intercept) -1.634e+00  7.890e-01  -2.071  0.04470 * 
## med_inc      1.435e-06  2.031e-06   0.707  0.48383   
## perAA       -2.062e-03  1.123e-03  -1.836  0.07368 . 
## perFem       2.379e-02  1.578e-02   1.508  0.13930   
## perBA        9.373e-03  3.158e-03   2.968  0.00499 **
## perUrb      -2.167e-04  8.476e-04  -0.256  0.79948   
## perHisp      2.148e-03  1.034e-03   2.078  0.04399 * 
## voteO        7.833e-04  1.116e-03   0.702  0.48684   
## vryrel       1.282e-03  1.176e-03   1.090  0.28215   
## ---
## Signif. codes:  0 '***' 0.001 '**' 0.01 '*' 0.05 '.' 0.1 ' ' 1
## 
## Residual standard error: 0.0515 on 41 degrees of freedom
## Multiple R-squared:  0.6629, Adjusted R-squared:  0.5971 
## F-statistic: 10.08 on 8 and 41 DF,  p-value: 1.237e-07
```

```
data$Ze_LT30<-scale(residuals(lm.E_LT30))
data$Za_LT30<-scale(residuals(lm.A_LT30))
data$Zc_LT30<-scale(residuals(lm.C_LT30))
data$Zn_LT30<-scale(residuals(lm.N_LT30))
data$Zo_LT30<-scale(residuals(lm.O_LT30))

data$Ze_GT30<-scale(residuals(lm.E_GT30))
data$Za_GT30<-scale(residuals(lm.A_GT30))
data$Zc_GT30<-scale(residuals(lm.C_GT30))
data$Zn_GT30<-scale(residuals(lm.N_GT30))
data$Zo_GT30<-scale(residuals(lm.O_GT30))
```

## 3.4 Age and Gender Differences Big Five

```
lm.GenD_E<-lm(GenD_E~med_inc+perAA+perFem+perBA+perUrb+perHisp+voteO+vryrel, data=data)
summary(lm.GenD_E)
```

```
## 
## Call:
## lm(formula = GenD_E ~ med_inc + perAA + perFem + perBA + perUrb + 
##     perHisp + voteO + vryrel, data = data)
## 
## Residuals:
##       Min        1Q    Median        3Q       Max 
## -0.042371 -0.007339  0.002070  0.011156  0.036824 
## 
## Coefficients:
##               Estimate Std. Error t value Pr(>|t|)
## (Intercept) -3.321e-01  2.684e-01  -1.237    0.223
## med_inc     -7.017e-07  6.909e-07  -1.016    0.316
## perAA       -6.321e-04  3.822e-04  -1.654    0.106
## perFem       4.866e-03  5.369e-03   0.906    0.370
## perBA        2.717e-05  1.074e-03   0.025    0.980
## perUrb       3.331e-04  2.884e-04   1.155    0.255
## perHisp     -2.788e-04  3.517e-04  -0.793    0.432
## voteO       -4.028e-04  3.798e-04  -1.061    0.295
## vryrel       7.577e-05  4.002e-04   0.189    0.851
## 
## Residual standard error: 0.01752 on 41 degrees of freedom
## Multiple R-squared:  0.1748, Adjusted R-squared:  0.01378 
## F-statistic: 1.086 on 8 and 41 DF,  p-value: 0.3923
```

```
lm.GenD_A<-lm(GenD_A~med_inc+perAA+perFem+perBA+perUrb+perHisp+voteO+vryrel, data=data)
summary(lm.GenD_A)
```

```
## 
## Call:
## lm(formula = GenD_A ~ med_inc + perAA + perFem + perBA + perUrb + 
##     perHisp + voteO + vryrel, data = data)
## 
## Residuals:
##       Min        1Q    Median        3Q       Max 
## -0.029633 -0.008523 -0.001368  0.010204  0.026678 
## 
## Coefficients:
##               Estimate Std. Error t value Pr(>|t|)   
## (Intercept) -6.887e-01  2.247e-01  -3.065  0.00384 **
## med_inc      8.139e-07  5.784e-07   1.407  0.16694   
## perAA       -5.562e-04  3.199e-04  -1.739  0.08962 . 
## perFem       1.069e-02  4.495e-03   2.378  0.02213 * 
## perBA       -7.829e-04  8.995e-04  -0.870  0.38915   
## perUrb       2.980e-04  2.414e-04   1.234  0.22415   
## perHisp      8.823e-05  2.944e-04   0.300  0.76594   
## voteO       -1.377e-04  3.180e-04  -0.433  0.66714   
## vryrel       2.777e-04  3.350e-04   0.829  0.41187   
## ---
## Signif. codes:  0 '***' 0.001 '**' 0.01 '*' 0.05 '.' 0.1 ' ' 1
## 
## Residual standard error: 0.01467 on 41 degrees of freedom
## Multiple R-squared:  0.2922, Adjusted R-squared:  0.1541 
## F-statistic: 2.116 on 8 and 41 DF,  p-value: 0.05613
```

```
lm.GenD_C<-lm(GenD_C~med_inc+perAA+perFem+perBA+perUrb+perHisp+voteO+vryrel, data=data)
summary(lm.GenD_C)
```

```
## 
## Call:
## lm(formula = GenD_C ~ med_inc + perAA + perFem + perBA + perUrb + 
##     perHisp + voteO + vryrel, data = data)
## 
## Residuals:
##       Min        1Q    Median        3Q       Max 
## -0.038861 -0.013989 -0.001062  0.010119  0.040994 
## 
## Coefficients:
##               Estimate Std. Error t value Pr(>|t|)  
## (Intercept)  2.369e-01  3.109e-01   0.762   0.4504  
## med_inc      4.053e-07  8.003e-07   0.506   0.6152  
## perAA        5.237e-04  4.427e-04   1.183   0.2436  
## perFem      -6.049e-03  6.219e-03  -0.973   0.3365  
## perBA       -2.576e-03  1.245e-03  -2.070   0.0448 *
## perUrb       3.966e-04  3.340e-04   1.187   0.2420  
## perHisp      9.724e-05  4.074e-04   0.239   0.8125  
## voteO       -1.875e-04  4.399e-04  -0.426   0.6722  
## vryrel       2.439e-04  4.635e-04   0.526   0.6016  
## ---
## Signif. codes:  0 '***' 0.001 '**' 0.01 '*' 0.05 '.' 0.1 ' ' 1
## 
## Residual standard error: 0.02029 on 41 degrees of freedom
## Multiple R-squared:  0.3506, Adjusted R-squared:  0.2239 
## F-statistic: 2.767 on 8 and 41 DF,  p-value: 0.01528
```

```
lm.GenD_N<-lm(GenD_N~med_inc+perAA+perFem+perBA+perUrb+perHisp+voteO+vryrel, data=data)
summary(lm.GenD_N)
```

```
## 
## Call:
## lm(formula = GenD_N ~ med_inc + perAA + perFem + perBA + perUrb + 
##     perHisp + voteO + vryrel, data = data)
## 
## Residuals:
##       Min        1Q    Median        3Q       Max 
## -0.042971 -0.011050 -0.001262  0.007852  0.064896 
## 
## Coefficients:
##               Estimate Std. Error t value Pr(>|t|)  
## (Intercept)  1.349e-01  3.107e-01   0.434    0.666  
## med_inc     -6.597e-07  7.999e-07  -0.825    0.414  
## perAA        4.148e-04  4.424e-04   0.938    0.354  
## perFem      -1.049e-02  6.216e-03  -1.688    0.099 .
## perBA        1.391e-03  1.244e-03   1.118    0.270  
## perUrb       5.390e-04  3.338e-04   1.615    0.114  
## perHisp      1.395e-04  4.071e-04   0.343    0.734  
## voteO        5.376e-04  4.397e-04   1.223    0.228  
## vryrel       2.102e-04  4.633e-04   0.454    0.652  
## ---
## Signif. codes:  0 '***' 0.001 '**' 0.01 '*' 0.05 '.' 0.1 ' ' 1
## 
## Residual standard error: 0.02028 on 41 degrees of freedom
## Multiple R-squared:  0.2814, Adjusted R-squared:  0.1412 
## F-statistic: 2.007 on 8 and 41 DF,  p-value: 0.06977
```

```
lm.GenD_O<-lm(GenD_O~med_inc+perAA+perFem+perBA+perUrb+perHisp+voteO+vryrel, data=data)
summary(lm.GenD_O)
```

```
## 
## Call:
## lm(formula = GenD_O ~ med_inc + perAA + perFem + perBA + perUrb + 
##     perHisp + voteO + vryrel, data = data)
## 
## Residuals:
##       Min        1Q    Median        3Q       Max 
## -0.047733 -0.012594 -0.000573  0.015292  0.031939 
## 
## Coefficients:
##               Estimate Std. Error t value Pr(>|t|)  
## (Intercept)  3.185e-01  3.192e-01   0.998   0.3241  
## med_inc     -1.202e-06  8.215e-07  -1.463   0.1511  
## perAA        6.620e-04  4.544e-04   1.457   0.1528  
## perFem      -2.566e-03  6.384e-03  -0.402   0.6899  
## perBA       -3.433e-05  1.278e-03  -0.027   0.9787  
## perUrb      -2.414e-04  3.429e-04  -0.704   0.4853  
## perHisp      9.165e-04  4.182e-04   2.192   0.0341 *
## voteO       -5.239e-04  4.516e-04  -1.160   0.2527  
## vryrel      -5.842e-04  4.758e-04  -1.228   0.2265  
## ---
## Signif. codes:  0 '***' 0.001 '**' 0.01 '*' 0.05 '.' 0.1 ' ' 1
## 
## Residual standard error: 0.02083 on 41 degrees of freedom
## Multiple R-squared:  0.3777, Adjusted R-squared:  0.2562 
## F-statistic:  3.11 on 8 and 41 DF,  p-value: 0.007738
```

```
lm.AgeD_E<-lm(AgeD_E~med_inc+perAA+perFem+perBA+perUrb+perHisp+voteO+vryrel, data=data)
summary(lm.AgeD_E)
```

```
## 
## Call:
## lm(formula = AgeD_E ~ med_inc + perAA + perFem + perBA + perUrb + 
##     perHisp + voteO + vryrel, data = data)
## 
## Residuals:
##       Min        1Q    Median        3Q       Max 
## -0.076841 -0.014801  0.001133  0.014805  0.043532 
## 
## Coefficients:
##               Estimate Std. Error t value Pr(>|t|)    
## (Intercept) -7.931e-02  4.319e-01  -0.184   0.8552    
## med_inc     -1.581e-06  1.112e-06  -1.422   0.1625    
## perAA       -1.020e-03  6.149e-04  -1.659   0.1047    
## perFem       4.858e-03  8.639e-03   0.562   0.5770    
## perBA       -1.377e-03  1.729e-03  -0.797   0.4303    
## perUrb       1.186e-03  4.640e-04   2.557   0.0144 *  
## perHisp     -2.622e-03  5.658e-04  -4.634 3.61e-05 ***
## voteO       -1.122e-03  6.111e-04  -1.837   0.0735 .  
## vryrel      -1.473e-03  6.439e-04  -2.287   0.0274 *  
## ---
## Signif. codes:  0 '***' 0.001 '**' 0.01 '*' 0.05 '.' 0.1 ' ' 1
## 
## Residual standard error: 0.02819 on 41 degrees of freedom
## Multiple R-squared:  0.5162, Adjusted R-squared:  0.4218 
## F-statistic: 5.468 on 8 and 41 DF,  p-value: 0.0001022
```

```
lm.AgeD_A<-lm(AgeD_A~med_inc+perAA+perFem+perBA+perUrb+perHisp+voteO+vryrel, data=data)
summary(lm.AgeD_A)
```

```
## 
## Call:
## lm(formula = AgeD_A ~ med_inc + perAA + perFem + perBA + perUrb + 
##     perHisp + voteO + vryrel, data = data)
## 
## Residuals:
##      Min       1Q   Median       3Q      Max 
## -0.06084 -0.02869 -0.01224  0.02253  0.12178 
## 
## Coefficients:
##               Estimate Std. Error t value Pr(>|t|)
## (Intercept) -4.066e-01  7.288e-01  -0.558    0.580
## med_inc     -2.474e-06  1.876e-06  -1.319    0.195
## perAA       -1.200e-03  1.038e-03  -1.156    0.254
## perFem       1.206e-02  1.458e-02   0.827    0.413
## perBA       -5.699e-04  2.917e-03  -0.195    0.846
## perUrb       1.264e-03  7.829e-04   1.614    0.114
## perHisp     -1.590e-03  9.548e-04  -1.665    0.103
## voteO       -6.278e-04  1.031e-03  -0.609    0.546
## vryrel      -1.546e-03  1.087e-03  -1.423    0.162
## 
## Residual standard error: 0.04757 on 41 degrees of freedom
## Multiple R-squared:  0.2254, Adjusted R-squared:  0.0743 
## F-statistic: 1.492 on 8 and 41 DF,  p-value: 0.1901
```

```
lm.AgeD_C<-lm(AgeD_C~med_inc+perAA+perFem+perBA+perUrb+perHisp+voteO+vryrel, data=data)
summary(lm.AgeD_C)
```

```
## 
## Call:
## lm(formula = AgeD_C ~ med_inc + perAA + perFem + perBA + perUrb + 
##     perHisp + voteO + vryrel, data = data)
## 
## Residuals:
##       Min        1Q    Median        3Q       Max 
## -0.075844 -0.030775  0.003687  0.026221  0.118041 
## 
## Coefficients:
##               Estimate Std. Error t value Pr(>|t|)   
## (Intercept)  4.128e-02  7.018e-01   0.059  0.95338   
## med_inc     -6.021e-06  1.807e-06  -3.333  0.00183 **
## perAA       -6.217e-04  9.992e-04  -0.622  0.53726   
## perFem       6.704e-03  1.404e-02   0.478  0.63553   
## perBA        6.228e-03  2.809e-03   2.217  0.03223 * 
## perUrb      -2.312e-04  7.540e-04  -0.307  0.76067   
## perHisp     -1.064e-03  9.195e-04  -1.157  0.25396   
## voteO       -1.348e-03  9.930e-04  -1.358  0.18194   
## vryrel      -2.041e-03  1.046e-03  -1.951  0.05793 . 
## ---
## Signif. codes:  0 '***' 0.001 '**' 0.01 '*' 0.05 '.' 0.1 ' ' 1
## 
## Residual standard error: 0.04581 on 41 degrees of freedom
## Multiple R-squared:  0.4929, Adjusted R-squared:  0.3939 
## F-statistic: 4.981 on 8 and 41 DF,  p-value: 0.0002368
```

```
lm.AgeD_N<-lm(AgeD_N~med_inc+perAA+perFem+perBA+perUrb+perHisp+voteO+vryrel, data=data)
summary(lm.AgeD_N)
```

```
## 
## Call:
## lm(formula = AgeD_N ~ med_inc + perAA + perFem + perBA + perUrb + 
##     perHisp + voteO + vryrel, data = data)
## 
## Residuals:
##       Min        1Q    Median        3Q       Max 
## -0.086164 -0.018946 -0.006172  0.017232  0.080838 
## 
## Coefficients:
##               Estimate Std. Error t value Pr(>|t|)  
## (Intercept) -7.810e-01  5.516e-01  -1.416   0.1644  
## med_inc      1.229e-06  1.420e-06   0.866   0.3917  
## perAA        4.298e-04  7.854e-04   0.547   0.5871  
## perFem       1.079e-02  1.103e-02   0.977   0.3341  
## perBA        1.532e-03  2.208e-03   0.694   0.4916  
## perUrb      -9.520e-04  5.926e-04  -1.606   0.1159  
## perHisp      9.409e-04  7.227e-04   1.302   0.2002  
## voteO        1.795e-03  7.805e-04   2.300   0.0266 *
## vryrel       2.047e-03  8.224e-04   2.490   0.0169 *
## ---
## Signif. codes:  0 '***' 0.001 '**' 0.01 '*' 0.05 '.' 0.1 ' ' 1
## 
## Residual standard error: 0.036 on 41 degrees of freedom
## Multiple R-squared:  0.3201, Adjusted R-squared:  0.1875 
## F-statistic: 2.413 on 8 and 41 DF,  p-value: 0.03097
```

```
lm.AgeD_O<-lm(AgeD_O~med_inc+perAA+perFem+perBA+perUrb+perHisp+voteO+vryrel, data=data)
summary(lm.AgeD_O)
```

```
## 
## Call:
## lm(formula = AgeD_O ~ med_inc + perAA + perFem + perBA + perUrb + 
##     perHisp + voteO + vryrel, data = data)
## 
## Residuals:
##       Min        1Q    Median        3Q       Max 
## -0.066368 -0.029409 -0.004619  0.025462  0.096767 
## 
## Coefficients:
##               Estimate Std. Error t value Pr(>|t|)   
## (Intercept)  1.871e+00  5.941e-01   3.149  0.00305 **
## med_inc     -3.092e-06  1.529e-06  -2.022  0.04974 * 
## perAA        2.918e-03  8.458e-04   3.450  0.00131 **
## perFem      -3.443e-02  1.188e-02  -2.897  0.00602 **
## perBA       -3.209e-03  2.378e-03  -1.349  0.18463   
## perUrb       7.095e-04  6.382e-04   1.112  0.27276   
## perHisp      4.457e-04  7.783e-04   0.573  0.57004   
## voteO        2.113e-04  8.406e-04   0.251  0.80278   
## vryrel       6.871e-04  8.856e-04   0.776  0.44232   
## ---
## Signif. codes:  0 '***' 0.001 '**' 0.01 '*' 0.05 '.' 0.1 ' ' 1
## 
## Residual standard error: 0.03877 on 41 degrees of freedom
## Multiple R-squared:  0.6346, Adjusted R-squared:  0.5633 
## F-statistic: 8.902 on 8 and 41 DF,  p-value: 5.708e-07
```

```
data$ZgenD_E<-scale(residuals(lm.GenD_E))
data$ZgenD_A<-scale(residuals(lm.GenD_A))
data$ZgenD_C<-scale(residuals(lm.GenD_C))
data$ZgenD_N<-scale(residuals(lm.GenD_N))
data$ZgenD_O<-scale(residuals(lm.GenD_O))

data$ZageD_E<-scale(residuals(lm.AgeD_E))
data$ZageD_A<-scale(residuals(lm.AgeD_A))
data$ZageD_C<-scale(residuals(lm.AgeD_C))
data$ZageD_N<-scale(residuals(lm.AgeD_N))
data$ZageD_O<-scale(residuals(lm.AgeD_O))
```

## 3.5 Fertility Schedule and Other Fertility Outcomes

```
lm.tfr<-lm(TFR~med_inc+perAA+perFem+perBA+perUrb+perHisp+voteO+vryrel, data=data)
summary(lm.tfr)
```

```
## 
## Call:
## lm(formula = TFR ~ med_inc + perAA + perFem + perBA + perUrb + 
##     perHisp + voteO + vryrel, data = data)
## 
## Residuals:
##      Min       1Q   Median       3Q      Max 
## -0.28531 -0.06116 -0.02127  0.06574  0.29707 
## 
## Coefficients:
##               Estimate Std. Error t value Pr(>|t|)    
## (Intercept)  1.035e+01  1.876e+00   5.518 2.10e-06 ***
## med_inc     -2.277e-06  4.829e-06  -0.472    0.640    
## perAA        2.986e-03  2.671e-03   1.118    0.270    
## perFem      -1.665e-01  3.753e-02  -4.437 6.71e-05 ***
## perBA       -4.117e-03  7.509e-03  -0.548    0.586    
## perUrb       2.590e-03  2.015e-03   1.285    0.206    
## perHisp      2.944e-04  2.458e-03   0.120    0.905    
## voteO       -1.391e-03  2.654e-03  -0.524    0.603    
## vryrel       2.621e-03  2.797e-03   0.937    0.354    
## ---
## Signif. codes:  0 '***' 0.001 '**' 0.01 '*' 0.05 '.' 0.1 ' ' 1
## 
## Residual standard error: 0.1224 on 41 degrees of freedom
## Multiple R-squared:  0.5725, Adjusted R-squared:  0.4891 
## F-statistic: 6.865 on 8 and 41 DF,  p-value: 1.072e-05
```

```
lm.alpha<-lm(alpha~med_inc+perAA+perFem+perBA+perUrb+perHisp+voteO+vryrel, data=data)
summary(lm.alpha)
```

```
## 
## Call:
## lm(formula = alpha ~ med_inc + perAA + perFem + perBA + perUrb + 
##     perHisp + voteO + vryrel, data = data)
## 
## Residuals:
##     Min      1Q  Median      3Q     Max 
## -3.2847 -0.4692  0.1108  0.5392  2.7982 
## 
## Coefficients:
##               Estimate Std. Error t value Pr(>|t|)  
## (Intercept)  4.204e+01  1.868e+01   2.250   0.0299 *
## med_inc      1.619e-05  4.810e-05   0.337   0.7381  
## perAA       -1.530e-03  2.660e-02  -0.057   0.9544  
## perFem      -5.138e-01  3.737e-01  -1.375   0.1767  
## perBA       -1.452e-01  7.479e-02  -1.941   0.0591 .
## perUrb      -5.126e-02  2.007e-02  -2.554   0.0145 *
## perHisp      1.877e-02  2.448e-02   0.767   0.4476  
## voteO        1.582e-03  2.644e-02   0.060   0.9526  
## vryrel       2.892e-02  2.786e-02   1.038   0.3053  
## ---
## Signif. codes:  0 '***' 0.001 '**' 0.01 '*' 0.05 '.' 0.1 ' ' 1
## 
## Residual standard error: 1.22 on 41 degrees of freedom
## Multiple R-squared:  0.5568, Adjusted R-squared:  0.4703 
## F-statistic: 6.438 on 8 and 41 DF,  p-value: 2.086e-05
```

```
lm.peak<-lm(peak~med_inc+perAA+perFem+perBA+perUrb+perHisp+voteO+vryrel, data=data)
summary(lm.peak)
```

```
## 
## Call:
## lm(formula = peak ~ med_inc + perAA + perFem + perBA + perUrb + 
##     perHisp + voteO + vryrel, data = data)
## 
## Residuals:
##     Min      1Q  Median      3Q     Max 
## -3.3918 -0.6857 -0.2349  0.7067  2.5862 
## 
## Coefficients:
##               Estimate Std. Error t value Pr(>|t|)    
## (Intercept) -5.039e+01  1.869e+01  -2.696 0.010136 *  
## med_inc      6.961e-05  4.812e-05   1.447 0.155607    
## perAA       -9.989e-02  2.661e-02  -3.753 0.000542 ***
## perFem       1.356e+00  3.739e-01   3.626 0.000789 ***
## perBA        2.106e-01  7.482e-02   2.815 0.007470 ** 
## perUrb       5.011e-02  2.008e-02   2.495 0.016711 *  
## perHisp     -8.219e-02  2.449e-02  -3.356 0.001715 ** 
## voteO       -1.960e-02  2.645e-02  -0.741 0.462848    
## vryrel      -3.613e-02  2.787e-02  -1.296 0.202066    
## ---
## Signif. codes:  0 '***' 0.001 '**' 0.01 '*' 0.05 '.' 0.1 ' ' 1
## 
## Residual standard error: 1.22 on 41 degrees of freedom
## Multiple R-squared:  0.7997, Adjusted R-squared:  0.7606 
## F-statistic: 20.46 on 8 and 41 DF,  p-value: 4.86e-12
```

```
lm.stop<-lm(stop~med_inc+perAA+perFem+perBA+perUrb+perHisp+voteO+vryrel, data=data)
summary(lm.stop)
```

```
## 
## Call:
## lm(formula = stop ~ med_inc + perAA + perFem + perBA + perUrb + 
##     perHisp + voteO + vryrel, data = data)
## 
## Residuals:
##      Min       1Q   Median       3Q      Max 
## -2.05776 -0.28151  0.06568  0.35137  1.03202 
## 
## Coefficients:
##               Estimate Std. Error t value Pr(>|t|)  
## (Intercept) -1.408e+01  9.478e+00  -1.486   0.1450  
## med_inc      1.255e-05  2.440e-05   0.515   0.6096  
## perAA       -3.316e-02  1.349e-02  -2.457   0.0183 *
## perFem       4.301e-01  1.896e-01   2.269   0.0286 *
## perBA       -3.260e-02  3.794e-02  -0.859   0.3952  
## perUrb      -1.198e-02  1.018e-02  -1.176   0.2463  
## perHisp     -3.276e-02  1.242e-02  -2.638   0.0117 *
## voteO       -2.480e-02  1.341e-02  -1.849   0.0716 .
## vryrel      -1.894e-02  1.413e-02  -1.340   0.1875  
## ---
## Signif. codes:  0 '***' 0.001 '**' 0.01 '*' 0.05 '.' 0.1 ' ' 1
## 
## Residual standard error: 0.6186 on 41 degrees of freedom
## Multiple R-squared:  0.518,  Adjusted R-squared:  0.424 
## F-statistic: 5.508 on 8 and 41 DF,  p-value: 9.552e-05
```

```
lm.ageFB<-lm(ageFB~med_inc+perAA+perFem+perBA+perUrb+perHisp+voteO+vryrel, data=data)
summary(lm.ageFB)
```

```
## 
## Call:
## lm(formula = ageFB ~ med_inc + perAA + perFem + perBA + perUrb + 
##     perHisp + voteO + vryrel, data = data)
## 
## Residuals:
##      Min       1Q   Median       3Q      Max 
## -1.10903 -0.24694  0.03315  0.25127  1.14552 
## 
## Coefficients:
##               Estimate Std. Error t value Pr(>|t|)    
## (Intercept) -1.272e+01  7.478e+00  -1.701 0.096450 .  
## med_inc      5.385e-05  1.925e-05   2.798 0.007807 ** 
## perAA       -3.703e-02  1.065e-02  -3.478 0.001210 ** 
## perFem       6.233e-01  1.496e-01   4.167 0.000155 ***
## perBA        1.096e-01  2.993e-02   3.663 0.000707 ***
## perUrb       3.805e-03  8.033e-03   0.474 0.638261    
## perHisp     -1.934e-02  9.797e-03  -1.974 0.055111 .  
## voteO        1.174e-02  1.058e-02   1.110 0.273518    
## vryrel       1.126e-03  1.115e-02   0.101 0.920048    
## ---
## Signif. codes:  0 '***' 0.001 '**' 0.01 '*' 0.05 '.' 0.1 ' ' 1
## 
## Residual standard error: 0.4881 on 41 degrees of freedom
## Multiple R-squared:  0.8603, Adjusted R-squared:  0.833 
## F-statistic: 31.56 on 8 and 41 DF,  p-value: 3.705e-15
```

```
lm.ageFM<-lm(t_ageFM~med_inc+perAA+perFem+perBA+perUrb+perHisp+voteO+vryrel, data=data)
summary(lm.ageFM)
```

```
## 
## Call:
## lm(formula = t_ageFM ~ med_inc + perAA + perFem + perBA + perUrb + 
##     perHisp + voteO + vryrel, data = data)
## 
## Residuals:
##     Min      1Q  Median      3Q     Max 
## -2.1489 -0.4046  0.0522  0.4244  1.2106 
## 
## Coefficients:
##               Estimate Std. Error t value Pr(>|t|)    
## (Intercept) -2.049e+01  1.157e+01  -1.771 0.084076 .  
## med_inc      2.618e-05  2.979e-05   0.879 0.384523    
## perAA       -6.378e-03  1.648e-02  -0.387 0.700682    
## perFem       8.518e-01  2.315e-01   3.680 0.000673 ***
## perBA        6.358e-02  4.632e-02   1.372 0.177371    
## perUrb      -3.773e-03  1.243e-02  -0.303 0.763076    
## perHisp      1.345e-02  1.516e-02   0.887 0.380283    
## voteO        2.762e-02  1.637e-02   1.687 0.099278 .  
## vryrel       1.067e-02  1.725e-02   0.619 0.539577    
## ---
## Signif. codes:  0 '***' 0.001 '**' 0.01 '*' 0.05 '.' 0.1 ' ' 1
## 
## Residual standard error: 0.7553 on 41 degrees of freedom
## Multiple R-squared:  0.6259, Adjusted R-squared:  0.5529 
## F-statistic: 8.576 on 8 and 41 DF,  p-value: 8.894e-07
```

```
lm.nvrmarr<-lm(nevermar~med_inc+perAA+perFem+perBA+perUrb+perHisp+voteO+vryrel, data=data)
summary(lm.nvrmarr)
```

```
## 
## Call:
## lm(formula = nevermar ~ med_inc + perAA + perFem + perBA + perUrb + 
##     perHisp + voteO + vryrel, data = data)
## 
## Residuals:
##       Min        1Q    Median        3Q       Max 
## -0.039330 -0.011945 -0.002977  0.010916  0.038814 
## 
## Coefficients:
##               Estimate Std. Error t value Pr(>|t|)  
## (Intercept) -1.609e-01  2.930e-01  -0.549   0.5859  
## med_inc      5.213e-07  7.542e-07   0.691   0.4934  
## perAA        9.388e-04  4.172e-04   2.250   0.0299 *
## perFem       5.760e-03  5.861e-03   0.983   0.3314  
## perBA        1.546e-03  1.173e-03   1.318   0.1947  
## perUrb       3.780e-04  3.148e-04   1.201   0.2367  
## perHisp      4.982e-04  3.839e-04   1.298   0.2016  
## voteO        7.353e-04  4.146e-04   1.774   0.0836 .
## vryrel       7.126e-04  4.368e-04   1.631   0.1105  
## ---
## Signif. codes:  0 '***' 0.001 '**' 0.01 '*' 0.05 '.' 0.1 ' ' 1
## 
## Residual standard error: 0.01912 on 41 degrees of freedom
## Multiple R-squared:  0.6223, Adjusted R-squared:  0.5485 
## F-statistic: 8.442 on 8 and 41 DF,  p-value: 1.07e-06
```

```
lm.div<-lm(divorce~med_inc+perAA+perFem+perBA+perUrb+perHisp+voteO+vryrel, data=data)
summary(lm.div)
```

```
## 
## Call:
## lm(formula = divorce ~ med_inc + perAA + perFem + perBA + perUrb + 
##     perHisp + voteO + vryrel, data = data)
## 
## Residuals:
##        Min         1Q     Median         3Q        Max 
## -0.0033755 -0.0015601  0.0001484  0.0014268  0.0033318 
## 
## Coefficients:
##               Estimate Std. Error t value Pr(>|t|)  
## (Intercept)  6.205e-02  2.817e-02   2.203   0.0333 *
## med_inc     -1.115e-08  7.251e-08  -0.154   0.8786  
## perAA        5.202e-05  4.011e-05   1.297   0.2019  
## perFem      -7.914e-04  5.635e-04  -1.405   0.1677  
## perBA       -2.327e-04  1.128e-04  -2.064   0.0454 *
## perUrb      -5.426e-07  3.026e-05  -0.018   0.9858  
## perHisp      2.366e-05  3.691e-05   0.641   0.5251  
## voteO       -1.080e-05  3.986e-05  -0.271   0.7877  
## vryrel      -1.725e-05  4.200e-05  -0.411   0.6835  
## ---
## Signif. codes:  0 '***' 0.001 '**' 0.01 '*' 0.05 '.' 0.1 ' ' 1
## 
## Residual standard error: 0.001839 on 41 degrees of freedom
## Multiple R-squared:  0.4015, Adjusted R-squared:  0.2848 
## F-statistic: 3.438 on 8 and 41 DF,  p-value: 0.004079
```

```
lm.coh<-lm(cohabit~med_inc+perAA+perFem+perBA+perUrb+perHisp+voteO+vryrel, data=data)
summary(lm.coh)
```

```
## 
## Call:
## lm(formula = cohabit ~ med_inc + perAA + perFem + perBA + perUrb + 
##     perHisp + voteO + vryrel, data = data)
## 
## Residuals:
##     Min      1Q  Median      3Q     Max 
## -1.6096 -0.4681 -0.1012  0.4701  2.2165 
## 
## Coefficients:
##               Estimate Std. Error t value Pr(>|t|)  
## (Intercept)  2.933e+01  1.276e+01   2.299   0.0267 *
## med_inc     -2.265e-05  3.284e-05  -0.690   0.4941  
## perAA       -2.978e-02  1.816e-02  -1.640   0.1087  
## perFem      -5.092e-01  2.552e-01  -1.996   0.0527 .
## perBA        1.192e-01  5.106e-02   2.334   0.0246 *
## perUrb       7.644e-04  1.370e-02   0.056   0.9558  
## perHisp     -1.255e-03  1.671e-02  -0.075   0.9405  
## voteO        2.654e-02  1.805e-02   1.470   0.1491  
## vryrel       3.954e-03  1.902e-02   0.208   0.8363  
## ---
## Signif. codes:  0 '***' 0.001 '**' 0.01 '*' 0.05 '.' 0.1 ' ' 1
## 
## Residual standard error: 0.8326 on 41 degrees of freedom
## Multiple R-squared:  0.5205, Adjusted R-squared:  0.427 
## F-statistic: 5.564 on 8 and 41 DF,  p-value: 8.689e-05
```

```
lm.nmf<-lm(t_nmf~med_inc+perAA+perFem+perBA+perUrb+perHisp+voteO+vryrel, data=data)
summary(lm.nmf)
```

```
## 
## Call:
## lm(formula = t_nmf ~ med_inc + perAA + perFem + perBA + perUrb + 
##     perHisp + voteO + vryrel, data = data)
## 
## Residuals:
##      Min       1Q   Median       3Q      Max 
## -10.6274  -1.9122   0.2301   2.3059   7.3740 
## 
## Coefficients:
##               Estimate Std. Error t value Pr(>|t|)   
## (Intercept) -8.450e+01  5.630e+01  -1.501  0.14109   
## med_inc     -3.185e-05  1.449e-04  -0.220  0.82712   
## perAA        2.806e-01  8.016e-02   3.501  0.00113 **
## perFem       2.529e+00  1.126e+00   2.245  0.03020 * 
## perBA       -5.013e-01  2.254e-01  -2.224  0.03169 * 
## perUrb      -1.323e-01  6.049e-02  -2.188  0.03446 * 
## perHisp      1.819e-01  7.377e-02   2.465  0.01797 * 
## voteO        1.663e-01  7.967e-02   2.088  0.04309 * 
## vryrel       9.194e-02  8.394e-02   1.095  0.27975   
## ---
## Signif. codes:  0 '***' 0.001 '**' 0.01 '*' 0.05 '.' 0.1 ' ' 1
## 
## Residual standard error: 3.675 on 41 degrees of freedom
## Multiple R-squared:  0.7137, Adjusted R-squared:  0.6578 
## F-statistic: 12.77 on 8 and 41 DF,  p-value: 5.356e-09
```

```
lm.unint<-lm(unintprg~med_inc+perAA+perFem+perBA+perUrb+perHisp+voteO+vryrel, data=data)
summary(lm.unint)
```

```
## 
## Call:
## lm(formula = unintprg ~ med_inc + perAA + perFem + perBA + perUrb + 
##     perHisp + voteO + vryrel, data = data)
## 
## Residuals:
##     Min      1Q  Median      3Q     Max 
## -9.0646 -2.1128  0.0923  1.7095  8.8319 
## 
## Coefficients:
##               Estimate Std. Error t value Pr(>|t|)    
## (Intercept)  3.487e+01  5.649e+01   0.617   0.5405    
## med_inc      4.639e-05  1.454e-04   0.319   0.7514    
## perAA        4.137e-01  8.043e-02   5.144 7.06e-06 ***
## perFem       1.165e-01  1.130e+00   0.103   0.9184    
## perBA       -2.162e-01  2.261e-01  -0.956   0.3447    
## perUrb      -5.690e-02  6.069e-02  -0.938   0.3540    
## perHisp      1.702e-01  7.402e-02   2.299   0.0267 *  
## voteO        1.851e-01  7.993e-02   2.315   0.0257 *  
## vryrel       7.857e-02  8.422e-02   0.933   0.3563    
## ---
## Signif. codes:  0 '***' 0.001 '**' 0.01 '*' 0.05 '.' 0.1 ' ' 1
## 
## Residual standard error: 3.687 on 41 degrees of freedom
## Multiple R-squared:  0.6234, Adjusted R-squared:  0.5499 
## F-statistic: 8.484 on 8 and 41 DF,  p-value: 1.009e-06
```

```
lm.abr<-lm(abortion~med_inc+perAA+perFem+perBA+perUrb+perHisp+voteO+vryrel, data=data)
summary(lm.abr)
```

```
## 
## Call:
## lm(formula = abortion ~ med_inc + perAA + perFem + perBA + perUrb + 
##     perHisp + voteO + vryrel, data = data)
## 
## Residuals:
##     Min      1Q  Median      3Q     Max 
## -8.1489 -3.7143 -0.2134  2.7764 15.0207 
## 
## Coefficients:
##               Estimate Std. Error t value Pr(>|t|)  
## (Intercept) -1.910e+02  8.430e+01  -2.266   0.0288 *
## med_inc      3.705e-04  2.170e-04   1.708   0.0953 .
## perAA        1.344e-01  1.200e-01   1.120   0.2692  
## perFem       3.260e+00  1.686e+00   1.933   0.0601 .
## perBA       -2.920e-02  3.374e-01  -0.087   0.9315  
## perUrb       1.626e-01  9.056e-02   1.795   0.0800 .
## perHisp      1.697e-01  1.104e-01   1.537   0.1321  
## voteO        1.412e-01  1.193e-01   1.184   0.2431  
## vryrel       3.882e-02  1.257e-01   0.309   0.7590  
## ---
## Signif. codes:  0 '***' 0.001 '**' 0.01 '*' 0.05 '.' 0.1 ' ' 1
## 
## Residual standard error: 5.502 on 41 degrees of freedom
## Multiple R-squared:  0.6437, Adjusted R-squared:  0.5742 
## F-statistic: 9.259 on 8 and 41 DF,  p-value: 3.543e-07
```

```
lm.fmpl<-lm(famplnpw~med_inc+perAA+perFem+perBA+perUrb+perHisp+voteO+vryrel, data=data)
summary(lm.fmpl)
```

```
## 
## Call:
## lm(formula = famplnpw ~ med_inc + perAA + perFem + perBA + perUrb + 
##     perHisp + voteO + vryrel, data = data)
## 
## Residuals:
##    Min     1Q Median     3Q    Max 
## -60.95 -35.96 -10.04  21.77 127.19 
## 
## Coefficients:
##               Estimate Std. Error t value Pr(>|t|)
## (Intercept) -4.350e+02  6.908e+02  -0.630    0.532
## med_inc      1.981e-03  1.778e-03   1.114    0.272
## perAA        8.881e-01  9.836e-01   0.903    0.372
## perFem       1.117e+01  1.382e+01   0.809    0.423
## perBA       -1.662e+00  2.765e+00  -0.601    0.551
## perUrb      -9.122e-01  7.422e-01  -1.229    0.226
## perHisp      1.144e+00  9.051e-01   1.264    0.213
## voteO       -4.892e-01  9.775e-01  -0.500    0.619
## vryrel      -1.647e-01  1.030e+00  -0.160    0.874
## 
## Residual standard error: 45.09 on 41 degrees of freedom
## Multiple R-squared:  0.1112, Adjusted R-squared:  -0.06225 
## F-statistic: 0.6411 on 8 and 41 DF,  p-value: 0.7387
```

```
data$Ztfr<-scale(residuals(lm.tfr))
data$Zalpha<-scale(residuals(lm.alpha))
data$Zpeak<-scale(residuals(lm.peak))
data$Zstop<-scale(residuals(lm.stop))

data$Zafb<-scale(residuals(lm.ageFB))
data$Zafm<-scale(residuals(lm.ageFM))
data$Znvm<-scale(residuals(lm.nvrmarr))
data$Zdiv<-scale(residuals(lm.div))
data$Zcoh<-scale(residuals(lm.coh))
data$Znmf<-scale(residuals(lm.nmf))
data$Zuni<-scale(residuals(lm.unint))
data$Zabr<-scale(residuals(lm.abr))
data$Zfmp<-scale(residuals(lm.fmpl))
```

# 4 Spatial autocorrelation

## 4.1 Read in data

In this section, we test whether the raw variables or the residuals display spatial autocorrelation. Spatial autocorrelation occurs when things that are geographically similar are also similar in their other variables (regions could also be systematically dissimilar, but that is a less common result).

To test for spatial autocorrelation, we read in a spatial datafile containing a map of the United States and merge with the personality/fertility data. Then, we create a matrix reflecting whether states are adjacent to one another using queen’s contiguity rules (i.e., if states’ borders touch in any way, then the state is a neighbor). We set zero.policy = TRUE to allow for geographic islands, such as Alaska and Hawaii.

```
map <- readOGR(dsn=getwd(), layer="US Map")
```

```
## OGR data source with driver: ESRI Shapefile 
## Source: "C:\Users\Daniel\Dropbox\1. research\Personality and Fertility\Personality Science Submission\R1\PerSci Rmark", layer: "US Map"
## with 50 features
## It has 5 fields
```

```
plot(map)
```

```
map.join <- merge(map, data, by="STATE_ABBR")

mapgal <- poly2nb(map.join)
weightQ1 <- nb2listw(mapgal, zero.policy = TRUE)
```

## 4.2 Test for spatial autocorrelation of raw variables

To test for spatial autocorrelation, we calculate Moran’s I. This statistic ranges from -1 to 1. Scores of 0 indicate no spatial autocorrelation, neighbors are random. A score near 1 indicates that neighbors are very similar to one another. A score near -1 indicates that neighbors are very dissimilar to one another. If there is evidence of spatial autocorrelation, then standard assumptions of statistical models, such as independent and identically distributed errors, do not hold.

To calculate Moran’s I, we use the moran.test() function. We use a queen’s contiguity weight matrix, indicate that we want to use a two sided p-value test, and that we will allow for geographic islands by setting zero.policy = TRUE.

### 4.2.1 Big Five

```
moran.test(map.join$rz_ext, weightQ1, alternative="two.sided", zero.policy = TRUE)
```

```
## 
##  Moran I test under randomisation
## 
## data:  map.join$rz_ext  
## weights: weightQ1  n reduced by no-neighbour observations
##   
## 
## Moran I statistic standard deviate = 2.536, p-value = 0.01121
## alternative hypothesis: two.sided
## sample estimates:
## Moran I statistic       Expectation          Variance 
##       0.219749516      -0.021276596       0.009032623
```

```
moran.test(map.join$rz_agr, weightQ1, alternative="two.sided", zero.policy = TRUE)
```

```
## 
##  Moran I test under randomisation
## 
## data:  map.join$rz_agr  
## weights: weightQ1  n reduced by no-neighbour observations
##   
## 
## Moran I statistic standard deviate = 2.7802, p-value = 0.005433
## alternative hypothesis: two.sided
## sample estimates:
## Moran I statistic       Expectation          Variance 
##       0.247086544      -0.021276596       0.009317691
```

```
moran.test(map.join$rz_cns, weightQ1, alternative="two.sided", zero.policy = TRUE)
```

```
## 
##  Moran I test under randomisation
## 
## data:  map.join$rz_cns  
## weights: weightQ1  n reduced by no-neighbour observations
##   
## 
## Moran I statistic standard deviate = 3.652, p-value = 0.0002602
## alternative hypothesis: two.sided
## sample estimates:
## Moran I statistic       Expectation          Variance 
##       0.336877933      -0.021276596       0.009618091
```

```
moran.test(map.join$rz_neu, weightQ1, alternative="two.sided", zero.policy = TRUE)
```

```
## 
##  Moran I test under randomisation
## 
## data:  map.join$rz_neu  
## weights: weightQ1  n reduced by no-neighbour observations
##   
## 
## Moran I statistic standard deviate = 5.5617, p-value = 2.672e-08
## alternative hypothesis: two.sided
## sample estimates:
## Moran I statistic       Expectation          Variance 
##       0.521763716      -0.021276596       0.009533565
```

```
moran.test(map.join$rz_opn, weightQ1, alternative="two.sided", zero.policy = TRUE)
```

```
## 
##  Moran I test under randomisation
## 
## data:  map.join$rz_opn  
## weights: weightQ1  n reduced by no-neighbour observations
##   
## 
## Moran I statistic standard deviate = 3.7539, p-value = 0.0001741
## alternative hypothesis: two.sided
## sample estimates:
## Moran I statistic       Expectation          Variance 
##        0.34679975       -0.02127660        0.00961413
```

### 4.2.2 Gendered Big Five

```
moran.test(map.join$mzextra, weightQ1, alternative="two.sided", zero.policy = TRUE)
```

```
## 
##  Moran I test under randomisation
## 
## data:  map.join$mzextra  
## weights: weightQ1  n reduced by no-neighbour observations
##   
## 
## Moran I statistic standard deviate = 2.6992, p-value = 0.006951
## alternative hypothesis: two.sided
## sample estimates:
## Moran I statistic       Expectation          Variance 
##       0.236927978      -0.021276596       0.009150872
```

```
moran.test(map.join$mzagree, weightQ1, alternative="two.sided", zero.policy = TRUE)
```

```
## 
##  Moran I test under randomisation
## 
## data:  map.join$mzagree  
## weights: weightQ1  n reduced by no-neighbour observations
##   
## 
## Moran I statistic standard deviate = 1.136, p-value = 0.256
## alternative hypothesis: two.sided
## sample estimates:
## Moran I statistic       Expectation          Variance 
##       0.087435697      -0.021276596       0.009158013
```

```
moran.test(map.join$mzconsc, weightQ1, alternative="two.sided", zero.policy = TRUE)
```

```
## 
##  Moran I test under randomisation
## 
## data:  map.join$mzconsc  
## weights: weightQ1  n reduced by no-neighbour observations
##   
## 
## Moran I statistic standard deviate = 4.2922, p-value = 1.769e-05
## alternative hypothesis: two.sided
## sample estimates:
## Moran I statistic       Expectation          Variance 
##       0.398898205      -0.021276596       0.009583032
```

```
moran.test(map.join$mzneuro, weightQ1, alternative="two.sided", zero.policy = TRUE)
```

```
## 
##  Moran I test under randomisation
## 
## data:  map.join$mzneuro  
## weights: weightQ1  n reduced by no-neighbour observations
##   
## 
## Moran I statistic standard deviate = 5.3252, p-value = 1.008e-07
## alternative hypothesis: two.sided
## sample estimates:
## Moran I statistic       Expectation          Variance 
##       0.499347396      -0.021276596       0.009558205
```

```
moran.test(map.join$mzopen, weightQ1, alternative="two.sided", zero.policy = TRUE)
```

```
## 
##  Moran I test under randomisation
## 
## data:  map.join$mzopen  
## weights: weightQ1  n reduced by no-neighbour observations
##   
## 
## Moran I statistic standard deviate = 3.2209, p-value = 0.001278
## alternative hypothesis: two.sided
## sample estimates:
## Moran I statistic       Expectation          Variance 
##       0.292967393      -0.021276596       0.009518577
```

```
moran.test(map.join$fzextra, weightQ1, alternative="two.sided", zero.policy = TRUE)
```

```
## 
##  Moran I test under randomisation
## 
## data:  map.join$fzextra  
## weights: weightQ1  n reduced by no-neighbour observations
##   
## 
## Moran I statistic standard deviate = 2.278, p-value = 0.02273
## alternative hypothesis: two.sided
## sample estimates:
## Moran I statistic       Expectation          Variance 
##       0.198113843      -0.021276596       0.009275349
```

```
moran.test(map.join$fzagree, weightQ1, alternative="two.sided", zero.policy = TRUE)
```

```
## 
##  Moran I test under randomisation
## 
## data:  map.join$fzagree  
## weights: weightQ1  n reduced by no-neighbour observations
##   
## 
## Moran I statistic standard deviate = 3.4069, p-value = 0.000657
## alternative hypothesis: two.sided
## sample estimates:
## Moran I statistic       Expectation          Variance 
##       0.308658522      -0.021276596       0.009378417
```

```
moran.test(map.join$fzconsc, weightQ1, alternative="two.sided", zero.policy = TRUE)
```

```
## 
##  Moran I test under randomisation
## 
## data:  map.join$fzconsc  
## weights: weightQ1  n reduced by no-neighbour observations
##   
## 
## Moran I statistic standard deviate = 3.3999, p-value = 0.0006742
## alternative hypothesis: two.sided
## sample estimates:
## Moran I statistic       Expectation          Variance 
##       0.310598685      -0.021276596       0.009528585
```

```
moran.test(map.join$fzneuro, weightQ1, alternative="two.sided", zero.policy = TRUE)
```

```
## 
##  Moran I test under randomisation
## 
## data:  map.join$fzneuro  
## weights: weightQ1  n reduced by no-neighbour observations
##   
## 
## Moran I statistic standard deviate = 5.4715, p-value = 4.462e-08
## alternative hypothesis: two.sided
## sample estimates:
## Moran I statistic       Expectation          Variance 
##       0.511707413      -0.021276596       0.009488807
```

```
moran.test(map.join$fzopen, weightQ1, alternative="two.sided", zero.policy = TRUE)
```

```
## 
##  Moran I test under randomisation
## 
## data:  map.join$fzopen  
## weights: weightQ1  n reduced by no-neighbour observations
##   
## 
## Moran I statistic standard deviate = 3.941, p-value = 8.114e-05
## alternative hypothesis: two.sided
## sample estimates:
## Moran I statistic       Expectation          Variance 
##       0.364056654      -0.021276596       0.009559961
```

### 4.2.3 Age Big Five

```
moran.test(map.join$E_LT30, weightQ1, alternative="two.sided", zero.policy = TRUE)
```

```
## 
##  Moran I test under randomisation
## 
## data:  map.join$E_LT30  
## weights: weightQ1  n reduced by no-neighbour observations
##   
## 
## Moran I statistic standard deviate = 2.6631, p-value = 0.007742
## alternative hypothesis: two.sided
## sample estimates:
## Moran I statistic       Expectation          Variance 
##        0.23457424       -0.02127660        0.00922993
```

```
moran.test(map.join$A_LT30, weightQ1, alternative="two.sided", zero.policy = TRUE)
```

```
## 
##  Moran I test under randomisation
## 
## data:  map.join$A_LT30  
## weights: weightQ1  n reduced by no-neighbour observations
##   
## 
## Moran I statistic standard deviate = 2.7362, p-value = 0.006215
## alternative hypothesis: two.sided
## sample estimates:
## Moran I statistic       Expectation          Variance 
##       0.242519680      -0.021276596       0.009294589
```

```
moran.test(map.join$C_LT30, weightQ1, alternative="two.sided", zero.policy = TRUE)
```

```
## 
##  Moran I test under randomisation
## 
## data:  map.join$C_LT30  
## weights: weightQ1  n reduced by no-neighbour observations
##   
## 
## Moran I statistic standard deviate = 3.1183, p-value = 0.001819
## alternative hypothesis: two.sided
## sample estimates:
## Moran I statistic       Expectation          Variance 
##        0.28242689       -0.02127660        0.00948578
```

```
moran.test(map.join$N_LT30, weightQ1, alternative="two.sided", zero.policy = TRUE)
```

```
## 
##  Moran I test under randomisation
## 
## data:  map.join$N_LT30  
## weights: weightQ1  n reduced by no-neighbour observations
##   
## 
## Moran I statistic standard deviate = 6.2483, p-value = 4.149e-10
## alternative hypothesis: two.sided
## sample estimates:
## Moran I statistic       Expectation          Variance 
##       0.589932389      -0.021276596       0.009568732
```

```
moran.test(map.join$O_LT30, weightQ1, alternative="two.sided", zero.policy = TRUE)
```

```
## 
##  Moran I test under randomisation
## 
## data:  map.join$O_LT30  
## weights: weightQ1  n reduced by no-neighbour observations
##   
## 
## Moran I statistic standard deviate = 3.2835, p-value = 0.001025
## alternative hypothesis: two.sided
## sample estimates:
## Moran I statistic       Expectation          Variance 
##       0.299706950      -0.021276596       0.009556335
```

```
moran.test(map.join$E_GT30, weightQ1, alternative="two.sided", zero.policy = TRUE)
```

```
## 
##  Moran I test under randomisation
## 
## data:  map.join$E_GT30  
## weights: weightQ1  n reduced by no-neighbour observations
##   
## 
## Moran I statistic standard deviate = 1.2784, p-value = 0.2011
## alternative hypothesis: two.sided
## sample estimates:
## Moran I statistic       Expectation          Variance 
##       0.101300647      -0.021276596       0.009192994
```

```
moran.test(map.join$A_GT30, weightQ1, alternative="two.sided", zero.policy = TRUE)
```

```
## 
##  Moran I test under randomisation
## 
## data:  map.join$A_GT30  
## weights: weightQ1  n reduced by no-neighbour observations
##   
## 
## Moran I statistic standard deviate = 1.4007, p-value = 0.1613
## alternative hypothesis: two.sided
## sample estimates:
## Moran I statistic       Expectation          Variance 
##       0.113914323      -0.021276596       0.009315698
```

```
moran.test(map.join$C_GT30, weightQ1, alternative="two.sided", zero.policy = TRUE)
```

```
## 
##  Moran I test under randomisation
## 
## data:  map.join$C_GT30  
## weights: weightQ1  n reduced by no-neighbour observations
##   
## 
## Moran I statistic standard deviate = 2.3552, p-value = 0.01851
## alternative hypothesis: two.sided
## sample estimates:
## Moran I statistic       Expectation          Variance 
##       0.209286091      -0.021276596       0.009583252
```

```
moran.test(map.join$N_GT30, weightQ1, alternative="two.sided", zero.policy = TRUE)
```

```
## 
##  Moran I test under randomisation
## 
## data:  map.join$N_GT30  
## weights: weightQ1  n reduced by no-neighbour observations
##   
## 
## Moran I statistic standard deviate = 2.5089, p-value = 0.01211
## alternative hypothesis: two.sided
## sample estimates:
## Moran I statistic       Expectation          Variance 
##       0.222651169      -0.021276596       0.009452537
```

```
moran.test(map.join$O_GT30, weightQ1, alternative="two.sided", zero.policy = TRUE)
```

```
## 
##  Moran I test under randomisation
## 
## data:  map.join$O_GT30  
## weights: weightQ1  n reduced by no-neighbour observations
##   
## 
## Moran I statistic standard deviate = 5.3748, p-value = 7.667e-08
## alternative hypothesis: two.sided
## sample estimates:
## Moran I statistic       Expectation          Variance 
##       0.505434804      -0.021276596       0.009603343
```

### 4.2.4 Gender and Age Differences

```
moran.test(map.join$AgeD_E, weightQ1, alternative="two.sided", zero.policy = TRUE)
```

```
## 
##  Moran I test under randomisation
## 
## data:  map.join$AgeD_E  
## weights: weightQ1  n reduced by no-neighbour observations
##   
## 
## Moran I statistic standard deviate = 2.0197, p-value = 0.04341
## alternative hypothesis: two.sided
## sample estimates:
## Moran I statistic       Expectation          Variance 
##       0.175888708      -0.021276596       0.009529564
```

```
moran.test(map.join$AgeD_A, weightQ1, alternative="two.sided", zero.policy = TRUE)
```

```
## 
##  Moran I test under randomisation
## 
## data:  map.join$AgeD_A  
## weights: weightQ1  n reduced by no-neighbour observations
##   
## 
## Moran I statistic standard deviate = 1.7387, p-value = 0.0821
## alternative hypothesis: two.sided
## sample estimates:
## Moran I statistic       Expectation          Variance 
##       0.145225596      -0.021276596       0.009170963
```

```
moran.test(map.join$AgeD_C, weightQ1, alternative="two.sided", zero.policy = TRUE)
```

```
## 
##  Moran I test under randomisation
## 
## data:  map.join$AgeD_C  
## weights: weightQ1  n reduced by no-neighbour observations
##   
## 
## Moran I statistic standard deviate = 0.79404, p-value = 0.4272
## alternative hypothesis: two.sided
## sample estimates:
## Moran I statistic       Expectation          Variance 
##       0.054985773      -0.021276596       0.009224387
```

```
moran.test(map.join$AgeD_N, weightQ1, alternative="two.sided", zero.policy = TRUE)
```

```
## 
##  Moran I test under randomisation
## 
## data:  map.join$AgeD_N  
## weights: weightQ1  n reduced by no-neighbour observations
##   
## 
## Moran I statistic standard deviate = 1.7464, p-value = 0.08074
## alternative hypothesis: two.sided
## sample estimates:
## Moran I statistic       Expectation          Variance 
##       0.147179922      -0.021276596       0.009304155
```

```
moran.test(map.join$AgeD_O, weightQ1, alternative="two.sided", zero.policy = TRUE)
```

```
## 
##  Moran I test under randomisation
## 
## data:  map.join$AgeD_O  
## weights: weightQ1  n reduced by no-neighbour observations
##   
## 
## Moran I statistic standard deviate = 6.0669, p-value = 1.304e-09
## alternative hypothesis: two.sided
## sample estimates:
## Moran I statistic       Expectation          Variance 
##       0.572387438      -0.021276596       0.009575145
```

```
moran.test(map.join$GenD_E, weightQ1, alternative="two.sided", zero.policy = TRUE)
```

```
## 
##  Moran I test under randomisation
## 
## data:  map.join$GenD_E  
## weights: weightQ1  n reduced by no-neighbour observations
##   
## 
## Moran I statistic standard deviate = 0.064115, p-value = 0.9489
## alternative hypothesis: two.sided
## sample estimates:
## Moran I statistic       Expectation          Variance 
##      -0.015059340      -0.021276596       0.009403132
```

```
moran.test(map.join$GenD_A, weightQ1, alternative="two.sided", zero.policy = TRUE)
```

```
## 
##  Moran I test under randomisation
## 
## data:  map.join$GenD_A  
## weights: weightQ1  n reduced by no-neighbour observations
##   
## 
## Moran I statistic standard deviate = 0.90486, p-value = 0.3655
## alternative hypothesis: two.sided
## sample estimates:
## Moran I statistic       Expectation          Variance 
##        0.06708334       -0.02127660        0.00953550
```

```
moran.test(map.join$GenD_C, weightQ1, alternative="two.sided", zero.policy = TRUE)
```

```
## 
##  Moran I test under randomisation
## 
## data:  map.join$GenD_C  
## weights: weightQ1  n reduced by no-neighbour observations
##   
## 
## Moran I statistic standard deviate = 1.4275, p-value = 0.1534
## alternative hypothesis: two.sided
## sample estimates:
## Moran I statistic       Expectation          Variance 
##       0.117120883      -0.021276596       0.009398831
```

```
moran.test(map.join$GenD_N, weightQ1, alternative="two.sided", zero.policy = TRUE)
```

```
## 
##  Moran I test under randomisation
## 
## data:  map.join$GenD_N  
## weights: weightQ1  n reduced by no-neighbour observations
##   
## 
## Moran I statistic standard deviate = 1.8666, p-value = 0.06196
## alternative hypothesis: two.sided
## sample estimates:
## Moran I statistic       Expectation          Variance 
##        0.15341387       -0.02127660        0.00875889
```

```
moran.test(map.join$GenD_O, weightQ1, alternative="two.sided", zero.policy = TRUE)
```

```
## 
##  Moran I test under randomisation
## 
## data:  map.join$GenD_O  
## weights: weightQ1  n reduced by no-neighbour observations
##   
## 
## Moran I statistic standard deviate = 3.708, p-value = 0.0002089
## alternative hypothesis: two.sided
## sample estimates:
## Moran I statistic       Expectation          Variance 
##       0.335833517      -0.021276596       0.009275016
```

### 4.2.5 Fertility Schedule and Other Fertility Outcomes

```
moran.test(map.join$TFR, weightQ1, alternative="two.sided", zero.policy = TRUE)
```

```
## 
##  Moran I test under randomisation
## 
## data:  map.join$TFR  
## weights: weightQ1  n reduced by no-neighbour observations
##   
## 
## Moran I statistic standard deviate = 5.4802, p-value = 4.248e-08
## alternative hypothesis: two.sided
## sample estimates:
## Moran I statistic       Expectation          Variance 
##       0.506859457      -0.021276596       0.009287405
```

```
moran.test(map.join$alpha, weightQ1, alternative="two.sided", zero.policy = TRUE)
```

```
## 
##  Moran I test under randomisation
## 
## data:  map.join$alpha  
## weights: weightQ1  n reduced by no-neighbour observations
##   
## 
## Moran I statistic standard deviate = 2.188, p-value = 0.02867
## alternative hypothesis: two.sided
## sample estimates:
## Moran I statistic       Expectation          Variance 
##       0.191061168      -0.021276596       0.009417919
```

```
moran.test(map.join$peak, weightQ1, alternative="two.sided", zero.policy = TRUE)
```

```
## 
##  Moran I test under randomisation
## 
## data:  map.join$peak  
## weights: weightQ1  n reduced by no-neighbour observations
##   
## 
## Moran I statistic standard deviate = 6.3153, p-value = 2.696e-10
## alternative hypothesis: two.sided
## sample estimates:
## Moran I statistic       Expectation          Variance 
##       0.598902700      -0.021276596       0.009643631
```

```
moran.test(map.join$stop, weightQ1, alternative="two.sided", zero.policy = TRUE)
```

```
## 
##  Moran I test under randomisation
## 
## data:  map.join$stop  
## weights: weightQ1  n reduced by no-neighbour observations
##   
## 
## Moran I statistic standard deviate = 3.077, p-value = 0.002091
## alternative hypothesis: two.sided
## sample estimates:
## Moran I statistic       Expectation          Variance 
##       0.276006840      -0.021276596       0.009334204
```

```
moran.test(map.join$ageFB, weightQ1, alternative="two.sided", zero.policy = TRUE)
```

```
## 
##  Moran I test under randomisation
## 
## data:  map.join$ageFB  
## weights: weightQ1  n reduced by no-neighbour observations
##   
## 
## Moran I statistic standard deviate = 6.8028, p-value = 1.026e-11
## alternative hypothesis: two.sided
## sample estimates:
## Moran I statistic       Expectation          Variance 
##       0.642975157      -0.021276596       0.009534191
```

```
moran.test(map.join$t_ageFM, weightQ1, alternative="two.sided", zero.policy = TRUE)
```

```
## 
##  Moran I test under randomisation
## 
## data:  map.join$t_ageFM  
## weights: weightQ1  n reduced by no-neighbour observations
##   
## 
## Moran I statistic standard deviate = 5.1301, p-value = 2.895e-07
## alternative hypothesis: two.sided
## sample estimates:
## Moran I statistic       Expectation          Variance 
##       0.476817819      -0.021276596       0.009426831
```

```
moran.test(map.join$nevermar, weightQ1, alternative="two.sided", zero.policy = TRUE)
```

```
## 
##  Moran I test under randomisation
## 
## data:  map.join$nevermar  
## weights: weightQ1  n reduced by no-neighbour observations
##   
## 
## Moran I statistic standard deviate = 3.742, p-value = 0.0001826
## alternative hypothesis: two.sided
## sample estimates:
## Moran I statistic       Expectation          Variance 
##       0.343528461      -0.021276596       0.009504201
```

```
moran.test(map.join$divorce, weightQ1, alternative="two.sided", zero.policy = TRUE)
```

```
## 
##  Moran I test under randomisation
## 
## data:  map.join$divorce  
## weights: weightQ1  n reduced by no-neighbour observations
##   
## 
## Moran I statistic standard deviate = 4.8246, p-value = 1.403e-06
## alternative hypothesis: two.sided
## sample estimates:
## Moran I statistic       Expectation          Variance 
##       0.450521394      -0.021276596       0.009562901
```

```
moran.test(map.join$cohabit, weightQ1, alternative="two.sided", zero.policy = TRUE)
```

```
## 
##  Moran I test under randomisation
## 
## data:  map.join$cohabit  
## weights: weightQ1  n reduced by no-neighbour observations
##   
## 
## Moran I statistic standard deviate = 5.3039, p-value = 1.134e-07
## alternative hypothesis: two.sided
## sample estimates:
## Moran I statistic       Expectation          Variance 
##       0.496877705      -0.021276596       0.009544078
```

```
moran.test(map.join$t_nmf, weightQ1, alternative="two.sided", zero.policy = TRUE)
```

```
## 
##  Moran I test under randomisation
## 
## data:  map.join$t_nmf  
## weights: weightQ1  n reduced by no-neighbour observations
##   
## 
## Moran I statistic standard deviate = 4.1381, p-value = 3.502e-05
## alternative hypothesis: two.sided
## sample estimates:
## Moran I statistic       Expectation          Variance 
##        0.37579225       -0.02127660        0.00920732
```

```
moran.test(map.join$unintprg, weightQ1, alternative="two.sided", zero.policy = TRUE)
```

```
## 
##  Moran I test under randomisation
## 
## data:  map.join$unintprg  
## weights: weightQ1  n reduced by no-neighbour observations
##   
## 
## Moran I statistic standard deviate = 5.1155, p-value = 3.13e-07
## alternative hypothesis: two.sided
## sample estimates:
## Moran I statistic       Expectation          Variance 
##       0.477030185      -0.021276596       0.009489068
```

```
moran.test(map.join$abortion, weightQ1, alternative="two.sided", zero.policy = TRUE)
```

```
## 
##  Moran I test under randomisation
## 
## data:  map.join$abortion  
## weights: weightQ1  n reduced by no-neighbour observations
##   
## 
## Moran I statistic standard deviate = 3.9119, p-value = 9.159e-05
## alternative hypothesis: two.sided
## sample estimates:
## Moran I statistic       Expectation          Variance 
##       0.356046132      -0.021276596       0.009303825
```

```
moran.test(map.join$famplnpw, weightQ1, alternative="two.sided", zero.policy = TRUE)
```

```
## 
##  Moran I test under randomisation
## 
## data:  map.join$famplnpw  
## weights: weightQ1  n reduced by no-neighbour observations
##   
## 
## Moran I statistic standard deviate = 1.4564, p-value = 0.1453
## alternative hypothesis: two.sided
## sample estimates:
## Moran I statistic       Expectation          Variance 
##       0.119579506      -0.021276596       0.009353902
```

### 4.2.6 Established Correlates

```
moran.test(map.join$med_inc, weightQ1, alternative="two.sided", zero.policy = TRUE)
```

```
## 
##  Moran I test under randomisation
## 
## data:  map.join$med_inc  
## weights: weightQ1  n reduced by no-neighbour observations
##   
## 
## Moran I statistic standard deviate = 3.0362, p-value = 0.002396
## alternative hypothesis: two.sided
## sample estimates:
## Moran I statistic       Expectation          Variance 
##       0.275012208      -0.021276596       0.009522845
```

```
moran.test(map.join$perAA, weightQ1, alternative="two.sided", zero.policy = TRUE)
```

```
## 
##  Moran I test under randomisation
## 
## data:  map.join$perAA  
## weights: weightQ1  n reduced by no-neighbour observations
##   
## 
## Moran I statistic standard deviate = 6.8386, p-value = 8e-12
## alternative hypothesis: two.sided
## sample estimates:
## Moran I statistic       Expectation          Variance 
##       0.640556470      -0.021276596       0.009366303
```

```
moran.test(map.join$perFem, weightQ1, alternative="two.sided", zero.policy = TRUE)
```

```
## 
##  Moran I test under randomisation
## 
## data:  map.join$perFem  
## weights: weightQ1  n reduced by no-neighbour observations
##   
## 
## Moran I statistic standard deviate = 5.7766, p-value = 7.621e-09
## alternative hypothesis: two.sided
## sample estimates:
## Moran I statistic       Expectation          Variance 
##       0.525846446      -0.021276596       0.008970549
```

```
moran.test(map.join$perBA, weightQ1, alternative="two.sided", zero.policy = TRUE)
```

```
## 
##  Moran I test under randomisation
## 
## data:  map.join$perBA  
## weights: weightQ1  n reduced by no-neighbour observations
##   
## 
## Moran I statistic standard deviate = 3.8548, p-value = 0.0001158
## alternative hypothesis: two.sided
## sample estimates:
## Moran I statistic       Expectation          Variance 
##       0.354907378      -0.021276596       0.009523571
```

```
moran.test(map.join$perUrb, weightQ1, alternative="two.sided", zero.policy = TRUE)
```

```
## 
##  Moran I test under randomisation
## 
## data:  map.join$perUrb  
## weights: weightQ1  n reduced by no-neighbour observations
##   
## 
## Moran I statistic standard deviate = 3.3336, p-value = 0.0008572
## alternative hypothesis: two.sided
## sample estimates:
## Moran I statistic       Expectation          Variance 
##       0.304214630      -0.021276596       0.009533242
```

```
moran.test(map.join$perHisp, weightQ1, alternative="two.sided", zero.policy = TRUE)
```

```
## 
##  Moran I test under randomisation
## 
## data:  map.join$perHisp  
## weights: weightQ1  n reduced by no-neighbour observations
##   
## 
## Moran I statistic standard deviate = 4.7694, p-value = 1.847e-06
## alternative hypothesis: two.sided
## sample estimates:
## Moran I statistic       Expectation          Variance 
##       0.424255949      -0.021276596       0.008726192
```

```
moran.test(map.join$voteO, weightQ1, alternative="two.sided", zero.policy = TRUE)
```

```
## 
##  Moran I test under randomisation
## 
## data:  map.join$voteO  
## weights: weightQ1  n reduced by no-neighbour observations
##   
## 
## Moran I statistic standard deviate = 2.0837, p-value = 0.03719
## alternative hypothesis: two.sided
## sample estimates:
## Moran I statistic       Expectation          Variance 
##       0.183092255      -0.021276596       0.009619837
```

```
moran.test(map.join$vryrel, weightQ1, alternative="two.sided", zero.policy = TRUE)
```

```
## 
##  Moran I test under randomisation
## 
## data:  map.join$vryrel  
## weights: weightQ1  n reduced by no-neighbour observations
##   
## 
## Moran I statistic standard deviate = 1.318, p-value = 0.1875
## alternative hypothesis: two.sided
## sample estimates:
## Moran I statistic       Expectation          Variance 
##        0.10786038       -0.02127660        0.00959979
```

### 4.2.7 General Description

As can be seen, most variables display at least moderate spatial autocorrelation. Importantly, the set of established correlates are all spatially autocorrelated as well. It may be the case that removing this source of variance from personality and fertility substantially reduces the spatial autocorrelation.

## 4.3 Test for spatial autocorrelation of residualized variables

Next, we perform the same analyses on the estimated residuals.

### 4.3.1 Big Five

```
moran.test(map.join$Zext, weightQ1, alternative="two.sided", zero.policy = TRUE)
```

```
## 
##  Moran I test under randomisation
## 
## data:  map.join$Zext  
## weights: weightQ1  n reduced by no-neighbour observations
##   
## 
## Moran I statistic standard deviate = 1.6863, p-value = 0.09174
## alternative hypothesis: two.sided
## sample estimates:
## Moran I statistic       Expectation          Variance 
##       0.142463813      -0.021276596       0.009428753
```

```
moran.test(map.join$Zagr, weightQ1, alternative="two.sided", zero.policy = TRUE)
```

```
## 
##  Moran I test under randomisation
## 
## data:  map.join$Zagr  
## weights: weightQ1  n reduced by no-neighbour observations
##   
## 
## Moran I statistic standard deviate = 1.5006, p-value = 0.1334
## alternative hypothesis: two.sided
## sample estimates:
## Moran I statistic       Expectation          Variance 
##       0.124792312      -0.021276596       0.009474584
```

```
moran.test(map.join$Zcns, weightQ1, alternative="two.sided", zero.policy = TRUE)
```

```
## 
##  Moran I test under randomisation
## 
## data:  map.join$Zcns  
## weights: weightQ1  n reduced by no-neighbour observations
##   
## 
## Moran I statistic standard deviate = 1.6077, p-value = 0.1079
## alternative hypothesis: two.sided
## sample estimates:
## Moran I statistic       Expectation          Variance 
##       0.136242506      -0.021276596       0.009599538
```

```
moran.test(map.join$Zneu, weightQ1, alternative="two.sided", zero.policy = TRUE)#sig
```

```
## 
##  Moran I test under randomisation
## 
## data:  map.join$Zneu  
## weights: weightQ1  n reduced by no-neighbour observations
##   
## 
## Moran I statistic standard deviate = 2.1213, p-value = 0.0339
## alternative hypothesis: two.sided
## sample estimates:
## Moran I statistic       Expectation          Variance 
##       0.186530528      -0.021276596       0.009596554
```

```
moran.test(map.join$Zopn, weightQ1, alternative="two.sided", zero.policy = TRUE)
```

```
## 
##  Moran I test under randomisation
## 
## data:  map.join$Zopn  
## weights: weightQ1  n reduced by no-neighbour observations
##   
## 
## Moran I statistic standard deviate = 1.78, p-value = 0.07508
## alternative hypothesis: two.sided
## sample estimates:
## Moran I statistic       Expectation          Variance 
##        0.15225319       -0.02127660        0.00950455
```

### 4.3.2 Gendered Big Five

```
moran.test(map.join$Zmext, weightQ1, alternative="two.sided", zero.policy = TRUE)
```

```
## 
##  Moran I test under randomisation
## 
## data:  map.join$Zmext  
## weights: weightQ1  n reduced by no-neighbour observations
##   
## 
## Moran I statistic standard deviate = 1.8786, p-value = 0.0603
## alternative hypothesis: two.sided
## sample estimates:
## Moran I statistic       Expectation          Variance 
##       0.159259611      -0.021276596       0.009235763
```

```
moran.test(map.join$Zmagr, weightQ1, alternative="two.sided", zero.policy = TRUE)
```

```
## 
##  Moran I test under randomisation
## 
## data:  map.join$Zmagr  
## weights: weightQ1  n reduced by no-neighbour observations
##   
## 
## Moran I statistic standard deviate = 0.54529, p-value = 0.5856
## alternative hypothesis: two.sided
## sample estimates:
## Moran I statistic       Expectation          Variance 
##         0.0317577        -0.0212766         0.0094594
```

```
moran.test(map.join$Zmcns, weightQ1, alternative="two.sided", zero.policy = TRUE)
```

```
## 
##  Moran I test under randomisation
## 
## data:  map.join$Zmcns  
## weights: weightQ1  n reduced by no-neighbour observations
##   
## 
## Moran I statistic standard deviate = 0.19717, p-value = 0.8437
## alternative hypothesis: two.sided
## sample estimates:
## Moran I statistic       Expectation          Variance 
##      -0.002306995      -0.021276596       0.009255939
```

```
moran.test(map.join$Zmneu, weightQ1, alternative="two.sided", zero.policy = TRUE)
```

```
## 
##  Moran I test under randomisation
## 
## data:  map.join$Zmneu  
## weights: weightQ1  n reduced by no-neighbour observations
##   
## 
## Moran I statistic standard deviate = 1.8028, p-value = 0.07142
## alternative hypothesis: two.sided
## sample estimates:
## Moran I statistic       Expectation          Variance 
##       0.154232698      -0.021276596       0.009478004
```

```
moran.test(map.join$Zmopn, weightQ1, alternative="two.sided", zero.policy = TRUE)
```

```
## 
##  Moran I test under randomisation
## 
## data:  map.join$Zmopn  
## weights: weightQ1  n reduced by no-neighbour observations
##   
## 
## Moran I statistic standard deviate = 0.19015, p-value = 0.8492
## alternative hypothesis: two.sided
## sample estimates:
## Moran I statistic       Expectation          Variance 
##      -0.002626617      -0.021276596       0.009619554
```

```
moran.test(map.join$Zfext, weightQ1, alternative="two.sided", zero.policy = TRUE)
```

```
## 
##  Moran I test under randomisation
## 
## data:  map.join$Zfext  
## weights: weightQ1  n reduced by no-neighbour observations
##   
## 
## Moran I statistic standard deviate = 1.2364, p-value = 0.2163
## alternative hypothesis: two.sided
## sample estimates:
## Moran I statistic       Expectation          Variance 
##       0.099402712      -0.021276596       0.009527437
```

```
moran.test(map.join$Zfagr, weightQ1, alternative="two.sided", zero.policy = TRUE)
```

```
## 
##  Moran I test under randomisation
## 
## data:  map.join$Zfagr  
## weights: weightQ1  n reduced by no-neighbour observations
##   
## 
## Moran I statistic standard deviate = 1.6848, p-value = 0.09203
## alternative hypothesis: two.sided
## sample estimates:
## Moran I statistic       Expectation          Variance 
##       0.142484175      -0.021276596       0.009447731
```

```
moran.test(map.join$Zfcns, weightQ1, alternative="two.sided", zero.policy = TRUE)
```

```
## 
##  Moran I test under randomisation
## 
## data:  map.join$Zfcns  
## weights: weightQ1  n reduced by no-neighbour observations
##   
## 
## Moran I statistic standard deviate = 1.5141, p-value = 0.13
## alternative hypothesis: two.sided
## sample estimates:
## Moran I statistic       Expectation          Variance 
##       0.126954035      -0.021276596       0.009584924
```

```
moran.test(map.join$Zfneu, weightQ1, alternative="two.sided", zero.policy = TRUE)#sig
```

```
## 
##  Moran I test under randomisation
## 
## data:  map.join$Zfneu  
## weights: weightQ1  n reduced by no-neighbour observations
##   
## 
## Moran I statistic standard deviate = 2.0581, p-value = 0.03958
## alternative hypothesis: two.sided
## sample estimates:
## Moran I statistic       Expectation          Variance 
##       0.179884093      -0.021276596       0.009553224
```

```
moran.test(map.join$Zfopn, weightQ1, alternative="two.sided", zero.policy = TRUE)
```

```
## 
##  Moran I test under randomisation
## 
## data:  map.join$Zfopn  
## weights: weightQ1  n reduced by no-neighbour observations
##   
## 
## Moran I statistic standard deviate = 1.9141, p-value = 0.0556
## alternative hypothesis: two.sided
## sample estimates:
## Moran I statistic       Expectation          Variance 
##        0.16394261       -0.02127660        0.00936334
```

### 4.3.3 Age Big Five

```
moran.test(map.join$Ze_LT30, weightQ1, alternative="two.sided", zero.policy = TRUE)
```

```
## 
##  Moran I test under randomisation
## 
## data:  map.join$Ze_LT30  
## weights: weightQ1  n reduced by no-neighbour observations
##   
## 
## Moran I statistic standard deviate = 1.4395, p-value = 0.15
## alternative hypothesis: two.sided
## sample estimates:
## Moran I statistic       Expectation          Variance 
##       0.118683779      -0.021276596       0.009452912
```

```
moran.test(map.join$Za_LT30, weightQ1, alternative="two.sided", zero.policy = TRUE)
```

```
## 
##  Moran I test under randomisation
## 
## data:  map.join$Za_LT30  
## weights: weightQ1  n reduced by no-neighbour observations
##   
## 
## Moran I statistic standard deviate = 1.6687, p-value = 0.09517
## alternative hypothesis: two.sided
## sample estimates:
## Moran I statistic       Expectation          Variance 
##       0.140636724      -0.021276596       0.009414306
```

```
moran.test(map.join$Zc_LT30, weightQ1, alternative="two.sided", zero.policy = TRUE)
```

```
## 
##  Moran I test under randomisation
## 
## data:  map.join$Zc_LT30  
## weights: weightQ1  n reduced by no-neighbour observations
##   
## 
## Moran I statistic standard deviate = 1.1202, p-value = 0.2626
## alternative hypothesis: two.sided
## sample estimates:
## Moran I statistic       Expectation          Variance 
##       0.088523394      -0.021276596       0.009607387
```

```
moran.test(map.join$Zn_LT30, weightQ1, alternative="two.sided", zero.policy = TRUE)
```

```
## 
##  Moran I test under randomisation
## 
## data:  map.join$Zn_LT30  
## weights: weightQ1  n reduced by no-neighbour observations
##   
## 
## Moran I statistic standard deviate = 1.914, p-value = 0.05562
## alternative hypothesis: two.sided
## sample estimates:
## Moran I statistic       Expectation          Variance 
##       0.166276906      -0.021276596       0.009602078
```

```
moran.test(map.join$Zo_LT30, weightQ1, alternative="two.sided", zero.policy = TRUE)
```

```
## 
##  Moran I test under randomisation
## 
## data:  map.join$Zo_LT30  
## weights: weightQ1  n reduced by no-neighbour observations
##   
## 
## Moran I statistic standard deviate = 1.6565, p-value = 0.09761
## alternative hypothesis: two.sided
## sample estimates:
## Moran I statistic       Expectation          Variance 
##       0.140560573      -0.021276596       0.009544599
```

```
moran.test(map.join$Ze_GT30, weightQ1, alternative="two.sided", zero.policy = TRUE)
```

```
## 
##  Moran I test under randomisation
## 
## data:  map.join$Ze_GT30  
## weights: weightQ1  n reduced by no-neighbour observations
##   
## 
## Moran I statistic standard deviate = 1.2012, p-value = 0.2297
## alternative hypothesis: two.sided
## sample estimates:
## Moran I statistic       Expectation          Variance 
##       0.095309158      -0.021276596       0.009419947
```

```
moran.test(map.join$Za_GT30, weightQ1, alternative="two.sided", zero.policy = TRUE)
```

```
## 
##  Moran I test under randomisation
## 
## data:  map.join$Za_GT30  
## weights: weightQ1  n reduced by no-neighbour observations
##   
## 
## Moran I statistic standard deviate = -0.44782, p-value = 0.6543
## alternative hypothesis: two.sided
## sample estimates:
## Moran I statistic       Expectation          Variance 
##      -0.064479389      -0.021276596       0.009307013
```

```
moran.test(map.join$Zc_GT30, weightQ1, alternative="two.sided", zero.policy = TRUE)
```

```
## 
##  Moran I test under randomisation
## 
## data:  map.join$Zc_GT30  
## weights: weightQ1  n reduced by no-neighbour observations
##   
## 
## Moran I statistic standard deviate = 1.7263, p-value = 0.08429
## alternative hypothesis: two.sided
## sample estimates:
## Moran I statistic       Expectation          Variance 
##       0.146954134      -0.021276596       0.009496705
```

```
moran.test(map.join$Zn_GT30, weightQ1, alternative="two.sided", zero.policy = TRUE)
```

```
## 
##  Moran I test under randomisation
## 
## data:  map.join$Zn_GT30  
## weights: weightQ1  n reduced by no-neighbour observations
##   
## 
## Moran I statistic standard deviate = 1.2682, p-value = 0.2047
## alternative hypothesis: two.sided
## sample estimates:
## Moran I statistic       Expectation          Variance 
##        0.10207849       -0.02127660        0.00946082
```

```
moran.test(map.join$Zo_GT30, weightQ1, alternative="two.sided", zero.policy = TRUE)
```

```
## 
##  Moran I test under randomisation
## 
## data:  map.join$Zo_GT30  
## weights: weightQ1  n reduced by no-neighbour observations
##   
## 
## Moran I statistic standard deviate = 1.7647, p-value = 0.07762
## alternative hypothesis: two.sided
## sample estimates:
## Moran I statistic       Expectation          Variance 
##        0.15129645       -0.02127660        0.00956355
```

### 4.3.4 Gender and Age Differences

```
moran.test(map.join$ZgenD_E, weightQ1, alternative="two.sided", zero.policy = TRUE)
```

```
## 
##  Moran I test under randomisation
## 
## data:  map.join$ZgenD_E  
## weights: weightQ1  n reduced by no-neighbour observations
##   
## 
## Moran I statistic standard deviate = -0.57557, p-value = 0.5649
## alternative hypothesis: two.sided
## sample estimates:
## Moran I statistic       Expectation          Variance 
##      -0.077112590      -0.021276596       0.009411032
```

```
moran.test(map.join$ZgenD_A, weightQ1, alternative="two.sided", zero.policy = TRUE)
```

```
## 
##  Moran I test under randomisation
## 
## data:  map.join$ZgenD_A  
## weights: weightQ1  n reduced by no-neighbour observations
##   
## 
## Moran I statistic standard deviate = -0.58174, p-value = 0.5607
## alternative hypothesis: two.sided
## sample estimates:
## Moran I statistic       Expectation          Variance 
##      -0.078113792      -0.021276596       0.009545655
```

```
moran.test(map.join$ZgenD_C, weightQ1, alternative="two.sided", zero.policy = TRUE)
```

```
## 
##  Moran I test under randomisation
## 
## data:  map.join$ZgenD_C  
## weights: weightQ1  n reduced by no-neighbour observations
##   
## 
## Moran I statistic standard deviate = 0.099089, p-value = 0.9211
## alternative hypothesis: two.sided
## sample estimates:
## Moran I statistic       Expectation          Variance 
##      -0.011596979      -0.021276596       0.009542664
```

```
moran.test(map.join$ZgenD_N, weightQ1, alternative="two.sided", zero.policy = TRUE)
```

```
## 
##  Moran I test under randomisation
## 
## data:  map.join$ZgenD_N  
## weights: weightQ1  n reduced by no-neighbour observations
##   
## 
## Moran I statistic standard deviate = 0.97522, p-value = 0.3294
## alternative hypothesis: two.sided
## sample estimates:
## Moran I statistic       Expectation          Variance 
##       0.071570419      -0.021276596       0.009064134
```

```
moran.test(map.join$ZgenD_O, weightQ1, alternative="two.sided", zero.policy = TRUE)
```

```
## 
##  Moran I test under randomisation
## 
## data:  map.join$ZgenD_O  
## weights: weightQ1  n reduced by no-neighbour observations
##   
## 
## Moran I statistic standard deviate = -0.41163, p-value = 0.6806
## alternative hypothesis: two.sided
## sample estimates:
## Moran I statistic       Expectation          Variance 
##      -0.061421294      -0.021276596       0.009511145
```

```
moran.test(map.join$ZageD_E, weightQ1, alternative="two.sided", zero.policy = TRUE)
```

```
## 
##  Moran I test under randomisation
## 
## data:  map.join$ZageD_E  
## weights: weightQ1  n reduced by no-neighbour observations
##   
## 
## Moran I statistic standard deviate = -0.2692, p-value = 0.7878
## alternative hypothesis: two.sided
## sample estimates:
## Moran I statistic       Expectation          Variance 
##      -0.047332134      -0.021276596       0.009368116
```

```
moran.test(map.join$ZageD_A, weightQ1, alternative="two.sided", zero.policy = TRUE)
```

```
## 
##  Moran I test under randomisation
## 
## data:  map.join$ZageD_A  
## weights: weightQ1  n reduced by no-neighbour observations
##   
## 
## Moran I statistic standard deviate = 1.3066, p-value = 0.1914
## alternative hypothesis: two.sided
## sample estimates:
## Moran I statistic       Expectation          Variance 
##       0.104139909      -0.021276596       0.009213604
```

```
moran.test(map.join$ZageD_C, weightQ1, alternative="two.sided", zero.policy = TRUE)
```

```
## 
##  Moran I test under randomisation
## 
## data:  map.join$ZageD_C  
## weights: weightQ1  n reduced by no-neighbour observations
##   
## 
## Moran I statistic standard deviate = -0.29786, p-value = 0.7658
## alternative hypothesis: two.sided
## sample estimates:
## Moran I statistic       Expectation          Variance 
##      -0.050252247      -0.021276596       0.009463232
```

```
moran.test(map.join$ZageD_N, weightQ1, alternative="two.sided", zero.policy = TRUE)
```

```
## 
##  Moran I test under randomisation
## 
## data:  map.join$ZageD_N  
## weights: weightQ1  n reduced by no-neighbour observations
##   
## 
## Moran I statistic standard deviate = -0.63478, p-value = 0.5256
## alternative hypothesis: two.sided
## sample estimates:
## Moran I statistic       Expectation          Variance 
##      -0.082559641      -0.021276596       0.009320287
```

```
moran.test(map.join$ZageD_O, weightQ1, alternative="two.sided", zero.policy = TRUE)
```

```
## 
##  Moran I test under randomisation
## 
## data:  map.join$ZageD_O  
## weights: weightQ1  n reduced by no-neighbour observations
##   
## 
## Moran I statistic standard deviate = 1.3097, p-value = 0.1903
## alternative hypothesis: two.sided
## sample estimates:
## Moran I statistic       Expectation          Variance 
##       0.105670283      -0.021276596       0.009395541
```

### 4.3.5 Fertility Schedule and Other Fertility Outcomes

```
moran.test(map.join$Ztfr, weightQ1, alternative="two.sided", zero.policy = TRUE)
```

```
## 
##  Moran I test under randomisation
## 
## data:  map.join$Ztfr  
## weights: weightQ1  n reduced by no-neighbour observations
##   
## 
## Moran I statistic standard deviate = 0.41829, p-value = 0.6757
## alternative hypothesis: two.sided
## sample estimates:
## Moran I statistic       Expectation          Variance 
##       0.019147579      -0.021276596       0.009339417
```

```
moran.test(map.join$Zalpha, weightQ1, alternative="two.sided", zero.policy = TRUE)
```

```
## 
##  Moran I test under randomisation
## 
## data:  map.join$Zalpha  
## weights: weightQ1  n reduced by no-neighbour observations
##   
## 
## Moran I statistic standard deviate = 1.5887, p-value = 0.1121
## alternative hypothesis: two.sided
## sample estimates:
## Moran I statistic       Expectation          Variance 
##       0.130828298      -0.021276596       0.009166919
```

```
moran.test(map.join$Zpeak, weightQ1, alternative="two.sided", zero.policy = TRUE) #sig
```

```
## 
##  Moran I test under randomisation
## 
## data:  map.join$Zpeak  
## weights: weightQ1  n reduced by no-neighbour observations
##   
## 
## Moran I statistic standard deviate = 2.6405, p-value = 0.008278
## alternative hypothesis: two.sided
## sample estimates:
## Moran I statistic       Expectation          Variance 
##       0.233391250      -0.021276596       0.009302006
```

```
moran.test(map.join$Zstop, weightQ1, alternative="two.sided", zero.policy = TRUE)
```

```
## 
##  Moran I test under randomisation
## 
## data:  map.join$Zstop  
## weights: weightQ1  n reduced by no-neighbour observations
##   
## 
## Moran I statistic standard deviate = 1.3112, p-value = 0.1898
## alternative hypothesis: two.sided
## sample estimates:
## Moran I statistic       Expectation          Variance 
##        0.10286954       -0.02127660        0.00896456
```

```
moran.test(map.join$Zafb, weightQ1, alternative="two.sided", zero.policy = TRUE)#sig
```

```
## 
##  Moran I test under randomisation
## 
## data:  map.join$Zafb  
## weights: weightQ1  n reduced by no-neighbour observations
##   
## 
## Moran I statistic standard deviate = 2.4223, p-value = 0.01542
## alternative hypothesis: two.sided
## sample estimates:
## Moran I statistic       Expectation          Variance 
##       0.212584213      -0.021276596       0.009320917
```

```
moran.test(map.join$Zafm, weightQ1, alternative="two.sided", zero.policy = TRUE)
```

```
## 
##  Moran I test under randomisation
## 
## data:  map.join$Zafm  
## weights: weightQ1  n reduced by no-neighbour observations
##   
## 
## Moran I statistic standard deviate = 1.0714, p-value = 0.284
## alternative hypothesis: two.sided
## sample estimates:
## Moran I statistic       Expectation          Variance 
##       0.081285091      -0.021276596       0.009163914
```

```
moran.test(map.join$Znvm, weightQ1, alternative="two.sided", zero.policy = TRUE)#sig
```

```
## 
##  Moran I test under randomisation
## 
## data:  map.join$Znvm  
## weights: weightQ1  n reduced by no-neighbour observations
##   
## 
## Moran I statistic standard deviate = 2.0043, p-value = 0.04504
## alternative hypothesis: two.sided
## sample estimates:
## Moran I statistic       Expectation          Variance 
##       0.174336803      -0.021276596       0.009524978
```

```
moran.test(map.join$Zdiv, weightQ1, alternative="two.sided", zero.policy = TRUE)#sig
```

```
## 
##  Moran I test under randomisation
## 
## data:  map.join$Zdiv  
## weights: weightQ1  n reduced by no-neighbour observations
##   
## 
## Moran I statistic standard deviate = 3.4038, p-value = 0.0006646
## alternative hypothesis: two.sided
## sample estimates:
## Moran I statistic       Expectation          Variance 
##       0.313508070      -0.021276596       0.009674109
```

```
moran.test(map.join$Zcoh, weightQ1, alternative="two.sided", zero.policy = TRUE)
```

```
## 
##  Moran I test under randomisation
## 
## data:  map.join$Zcoh  
## weights: weightQ1  n reduced by no-neighbour observations
##   
## 
## Moran I statistic standard deviate = 0.90792, p-value = 0.3639
## alternative hypothesis: two.sided
## sample estimates:
## Moran I statistic       Expectation          Variance 
##       0.066600041      -0.021276596       0.009368192
```

```
moran.test(map.join$Znmf, weightQ1, alternative="two.sided", zero.policy = TRUE)
```

```
## 
##  Moran I test under randomisation
## 
## data:  map.join$Znmf  
## weights: weightQ1  n reduced by no-neighbour observations
##   
## 
## Moran I statistic standard deviate = 0.29754, p-value = 0.7661
## alternative hypothesis: two.sided
## sample estimates:
## Moran I statistic       Expectation          Variance 
##       0.007106091      -0.021276596       0.009099488
```

```
moran.test(map.join$Zuni, weightQ1, alternative="two.sided", zero.policy = TRUE)
```

```
## 
##  Moran I test under randomisation
## 
## data:  map.join$Zuni  
## weights: weightQ1  n reduced by no-neighbour observations
##   
## 
## Moran I statistic standard deviate = 0.14296, p-value = 0.8863
## alternative hypothesis: two.sided
## sample estimates:
## Moran I statistic       Expectation          Variance 
##      -0.007500628      -0.021276596       0.009286091
```

```
moran.test(map.join$Zabr, weightQ1, alternative="two.sided", zero.policy = TRUE)
```

```
## 
##  Moran I test under randomisation
## 
## data:  map.join$Zabr  
## weights: weightQ1  n reduced by no-neighbour observations
##   
## 
## Moran I statistic standard deviate = -0.55708, p-value = 0.5775
## alternative hypothesis: two.sided
## sample estimates:
## Moran I statistic       Expectation          Variance 
##      -0.074838252      -0.021276596       0.009244384
```

```
moran.test(map.join$Zfmp, weightQ1, alternative="two.sided", zero.policy = TRUE)
```

```
## 
##  Moran I test under randomisation
## 
## data:  map.join$Zfmp  
## weights: weightQ1  n reduced by no-neighbour observations
##   
## 
## Moran I statistic standard deviate = 0.68951, p-value = 0.4905
## alternative hypothesis: two.sided
## sample estimates:
## Moran I statistic       Expectation          Variance 
##        0.04531429       -0.02127660        0.00932724
```

### 4.3.6 General Description

For many of the personality and fertility variables, removing the variance associated with the set of established correlates reduces spatial autocorrelation to non-significance.

There are only six remaining variabiles for which spatial autocorrelation is significant: neuroticism, female neuroticism, peak fertility, age at first birth, never married, and divorce.

## 4.4 Spatial regression

Because six variables displayed significant spatial autocorrelation following residualization, we turned to spatial regression models. Spatial regression models explicitly model the geographic relation between regions. Spatial lag models specify that the influence of one region spreads to neighbor regions, and spatial error models specify correlated error terms among neighbors. Models can also combine these two elements. Fit statistics can be used to infer the best fitting model.

Our goal for testing spatial regression models was that this approach could more effectively reduce the spatial autocorrelation of the residuals. The following code runs the spatial regression model, determines the best fitting version, and tests whether residuals from that model still display spatial autocorrelation.

### 4.4.1 Peak

```
spt.peak<- lm(peak~med_inc+perAA+perFem+perBA+perUrb+perHisp+voteO+vryrel, data = map.join)
moran.test(spt.peak$residuals, weightQ1, alternative="two.sided", zero.policy = TRUE)
```

```
## 
##  Moran I test under randomisation
## 
## data:  spt.peak$residuals  
## weights: weightQ1  n reduced by no-neighbour observations
##   
## 
## Moran I statistic standard deviate = 2.6405, p-value = 0.008278
## alternative hypothesis: two.sided
## sample estimates:
## Moran I statistic       Expectation          Variance 
##       0.233391250      -0.021276596       0.009302006
```

```
lm.LMtests(spt.peak, weightQ1, zero.policy = TRUE, test=c("LMerr", "LMlag", "RLMerr","RLMlag", "SARMA"))
```

```
## 
##  Lagrange multiplier diagnostics for spatial dependence
## 
## data:  
## model: lm(formula = peak ~ med_inc + perAA + perFem + perBA + perUrb +
## perHisp + voteO + vryrel, data = map.join)
## weights: weightQ1
## 
## LMerr = 5.6871, df = 1, p-value = 0.01709
## 
## 
##  Lagrange multiplier diagnostics for spatial dependence
## 
## data:  
## model: lm(formula = peak ~ med_inc + perAA + perFem + perBA + perUrb +
## perHisp + voteO + vryrel, data = map.join)
## weights: weightQ1
## 
## LMlag = 18.929, df = 1, p-value = 1.356e-05
## 
## 
##  Lagrange multiplier diagnostics for spatial dependence
## 
## data:  
## model: lm(formula = peak ~ med_inc + perAA + perFem + perBA + perUrb +
## perHisp + voteO + vryrel, data = map.join)
## weights: weightQ1
## 
## RLMerr = 2.4962, df = 1, p-value = 0.1141
## 
## 
##  Lagrange multiplier diagnostics for spatial dependence
## 
## data:  
## model: lm(formula = peak ~ med_inc + perAA + perFem + perBA + perUrb +
## perHisp + voteO + vryrel, data = map.join)
## weights: weightQ1
## 
## RLMlag = 15.738, df = 1, p-value = 7.273e-05
## 
## 
##  Lagrange multiplier diagnostics for spatial dependence
## 
## data:  
## model: lm(formula = peak ~ med_inc + perAA + perFem + perBA + perUrb +
## perHisp + voteO + vryrel, data = map.join)
## weights: weightQ1
## 
## SARMA = 21.426, df = 2, p-value = 2.226e-05
```

```
#supports using spatial error model
err.peak<-errorsarlm(peak~med_inc+perAA+perFem+perBA+perUrb+perHisp+voteO+vryrel, 
                     data = map.join, weightQ1, method="eigen", quiet = FALSE, zero.policy=TRUE, tol.solve=1.0e-15)
```

```
## Warning: Function errorsarlm moved to the spatialreg package
```

```
## Warning in errorsarlm(peak ~ med_inc + perAA + perFem + perBA + perUrb + :
## install the spatialreg package
```

```
## 
## Spatial autoregressive error model
```

```
## Warning: Function can.be.simmed moved to the spatialreg package
```

```
## Warning in can.be.simmed(listw): install the spatialreg package
```

```
## 
## Jacobian calculated using
```

```
## Warning: Function jacobianSetup moved to the spatialreg package
```

```
## Warning in jacobianSetup(method, env, con, pre_eig = con$pre_eig, trs = trs, :
## install the spatialreg package
```

```
## neighbourhood matrix eigenvalues
```

```
## Warning: Function eigen_setup moved to the spatialreg package
```

```
## Warning in eigen_setup(env, which = which): install the spatialreg package
```

```
## Computing eigenvalues ...
```

```
## Warning: Function as_dgRMatrix_listw moved to the spatialreg package
```

```
## Warning in as_dgRMatrix_listw(from): install the spatialreg package
```

```
## Warning: Function do_ldet moved to the spatialreg package
```

```
## Warning in do_ldet(lambda, env): install the spatialreg package
```

```
## lambda: -0.4785762  function: -82.16793  Jacobian: -1.219999  SSE: 74.59424
```

```
## Warning: Function do_ldet moved to the spatialreg package

## Warning: install the spatialreg package
```

```
## lambda: 0.08618964  function: -74.94502  Jacobian: -0.04223568  SSE: 58.57204
```

```
## Warning: Function do_ldet moved to the spatialreg package

## Warning: install the spatialreg package
```

```
## lambda: 0.4352341  function: -71.37736  Jacobian: -1.215038  SSE: 48.45516
```

```
## Warning: Function do_ldet moved to the spatialreg package

## Warning: install the spatialreg package
```

```
## lambda: 0.6509555  function: -69.66746  Jacobian: -3.0968  SSE: 41.97072
```

```
## Warning: Function do_ldet moved to the spatialreg package

## Warning: install the spatialreg package
```

```
## lambda: 0.7842786  function: -68.96308  Jacobian: -5.08969  SSE: 37.6782
```

```
## Warning: Function do_ldet moved to the spatialreg package

## Warning: install the spatialreg package
```

```
## lambda: 0.8666769  function: -68.54821  Jacobian: -6.949411  SSE: 34.40142
```

```
## Warning: Function do_ldet moved to the spatialreg package

## Warning: install the spatialreg package
```

```
## lambda: 0.9176018  function: -68.23658  Jacobian: -8.585276  SSE: 31.82329
```

```
## Warning: Function do_ldet moved to the spatialreg package

## Warning: install the spatialreg package
```

```
## lambda: 0.9490751  function: -68.2971  Jacobian: -9.980033  SSE: 30.16942
```

```
## Warning: Function do_ldet moved to the spatialreg package

## Warning: install the spatialreg package
```

```
## lambda: 0.923488  function: -68.21618  Jacobian: -8.815633  SSE: 31.50568
```

```
## Warning: Function do_ldet moved to the spatialreg package

## Warning: install the spatialreg package
```

```
## lambda: 0.928774  function: -68.20639  Jacobian: -9.032677  SSE: 31.22111
```

```
## Warning: Function do_ldet moved to the spatialreg package

## Warning: install the spatialreg package
```

```
## lambda: 0.9365283  function: -68.21217  Jacobian: -9.37098  SSE: 30.80858
```

```
## Warning: Function do_ldet moved to the spatialreg package

## Warning: install the spatialreg package
```

```
## lambda: 0.9307808  function: -68.20531  Jacobian: -9.117836  SSE: 31.1136
```

```
## Warning: Function do_ldet moved to the spatialreg package

## Warning: install the spatialreg package
```

```
## lambda: 0.9309789  function: -68.2053  Jacobian: -9.126329  SSE: 31.10301
```

```
## Warning: Function do_ldet moved to the spatialreg package

## Warning: install the spatialreg package
```

```
## lambda: 0.9310918  function: -68.20529  Jacobian: -9.131175  SSE: 31.09698
```

```
## Warning: Function do_ldet moved to the spatialreg package

## Warning: install the spatialreg package
```

```
## lambda: 0.9310841  function: -68.20529  Jacobian: -9.130847  SSE: 31.09739
```

```
## Warning: Function do_ldet moved to the spatialreg package

## Warning: install the spatialreg package
```

```
## lambda: 0.9310839  function: -68.20529  Jacobian: -9.130837  SSE: 31.0974
```

```
## Warning: Function do_ldet moved to the spatialreg package

## Warning: install the spatialreg package
```

```
## lambda: 0.9310839  function: -68.20529  Jacobian: -9.130838  SSE: 31.0974
```

```
## Warning: Function do_ldet moved to the spatialreg package

## Warning: install the spatialreg package
```

```
## lambda: 0.9310438  function: -68.20529  Jacobian: -9.129115  SSE: 31.09954
```

```
## Warning: Function do_ldet moved to the spatialreg package

## Warning: install the spatialreg package
```

```
## lambda: 0.9310827  function: -68.20529  Jacobian: -9.130785  SSE: 31.09747
```

```
## Warning: Function do_ldet moved to the spatialreg package

## Warning: install the spatialreg package
```

```
## lambda: 0.9310834  function: -68.20529  Jacobian: -9.130817  SSE: 31.09743
```

```
## Warning: Function do_ldet moved to the spatialreg package

## Warning: install the spatialreg package
```

```
## lambda: 0.9310837  function: -68.20529  Jacobian: -9.130829  SSE: 31.09741
```

```
## Warning: Function do_ldet moved to the spatialreg package

## Warning: install the spatialreg package
```

```
## lambda: 0.9310838  function: -68.20529  Jacobian: -9.130835  SSE: 31.0974
```

```
## Warning: Function do_ldet moved to the spatialreg package

## Warning: install the spatialreg package
```

```
## lambda: 0.9310839  function: -68.20529  Jacobian: -9.130836  SSE: 31.0974
```

```
## Warning: Function do_ldet moved to the spatialreg package

## Warning: install the spatialreg package
```

```
## lambda: 0.9310839  function: -68.20529  Jacobian: -9.130837  SSE: 31.0974
```

```
## Warning in if (class(asyvar1) == "try-error") {: the condition has length > 1
## and only the first element will be used
```

```
summary(err.peak)
```

```
## Warning: Method summary.sarlm moved to the spatialreg package
```

```
## Warning in summary.sarlm(err.peak): install the spatialreg package
```

```
## Warning: Method Wald1.sarlm moved to the spatialreg package
```

```
## Warning in Wald1.sarlm(object): install the spatialreg package
```

```
## Warning: Method logLik.sarlm moved to the spatialreg package
```

```
## Warning in logLik.sarlm(object): install the spatialreg package
```

```
## Warning: Method residuals.sarlm moved to the spatialreg package
```

```
## Warning in residuals.sarlm(object): install the spatialreg package
```

```
## Warning: Method LR1.sarlm moved to the spatialreg package
```

```
## Warning in LR1.sarlm(object): install the spatialreg package
```

```
## Warning: Method logLik.sarlm moved to the spatialreg package
```

```
## Warning in logLik.sarlm(object): install the spatialreg package
```

```
## Warning: Method residuals.sarlm moved to the spatialreg package
```

```
## Warning in residuals.sarlm(object): install the spatialreg package
```

```
## Warning: Method print.summary.sarlm moved to the spatialreg package
```

```
## Warning in print.summary.sarlm(x): install the spatialreg package
```

```
## 
## Call:errorsarlm(formula = peak ~ med_inc + perAA + perFem + perBA + 
##     perUrb + perHisp + voteO + vryrel, data = map.join, listw = weightQ1, 
##     method = "eigen", quiet = FALSE, zero.policy = TRUE, tol.solve = 1e-15)
## 
## Residuals:
```

```
## Warning: Method residuals.sarlm moved to the spatialreg package
```

```
## Warning in residuals.sarlm(x): install the spatialreg package
```

```
##      Min       1Q   Median       3Q      Max 
## -1.31090 -0.40717  0.13031  0.79339  2.16224 
## 
## Type: error 
## Regions with no neighbours included:
##  0 49 
## Coefficients: (asymptotic standard errors) 
##                Estimate  Std. Error z value  Pr(>|z|)
## (Intercept) -2.2574e+00  2.2067e+01 -0.1023 0.9185202
## med_inc      3.0690e-05  4.0505e-05  0.7577 0.4486458
## perAA       -2.7967e-02  2.5807e-02 -1.0837 0.2784987
## perFem       3.3276e-01  4.3653e-01  0.7623 0.4458826
## perBA        1.8982e-01  5.3217e-02  3.5669 0.0003613
## perUrb       5.9115e-02  1.6625e-02  3.5558 0.0003768
## perHisp     -4.6480e-02  1.9568e-02 -2.3754 0.0175321
## voteO        5.1126e-03  1.7171e-02  0.2978 0.7658894
## vryrel      -1.8827e-02  1.7036e-02 -1.1051 0.2691018
## 
## Lambda: 0.93108, LR test value: 15.452, p-value: 8.4649e-05
## Asymptotic standard error: 0.03564
##     z-value: 26.125, p-value: < 2.22e-16
## Wald statistic: 682.5, p-value: < 2.22e-16
```

```
## Warning: Method logLik.sarlm moved to the spatialreg package
```

```
## Warning in logLik.sarlm(x): install the spatialreg package
```

```
## Warning: Method residuals.sarlm moved to the spatialreg package
```

```
## Warning in residuals.sarlm(object): install the spatialreg package
```

```
## 
## Log likelihood: -68.20529 for error model
## ML residual variance (sigma squared): 0.62195, (sigma: 0.78864)
## Number of observations: 50 
## Number of parameters estimated: 11
```

```
## Warning: Method logLik.sarlm moved to the spatialreg package
```

```
## Warning in logLik.sarlm(object): install the spatialreg package
```

```
## Warning: Method residuals.sarlm moved to the spatialreg package
```

```
## Warning in residuals.sarlm(object): install the spatialreg package
```

```
## AIC: 158.41, (AIC for lm: 171.86)
```

```
moran.test(err.peak$residuals, weightQ1, alternative="two.sided", zero.policy = TRUE)
```

```
## 
##  Moran I test under randomisation
## 
## data:  err.peak$residuals  
## weights: weightQ1  n reduced by no-neighbour observations
##   
## 
## Moran I statistic standard deviate = -1.6767, p-value = 0.0936
## alternative hypothesis: two.sided
## sample estimates:
## Moran I statistic       Expectation          Variance 
##      -0.184496331      -0.021276596       0.009476351
```

```
#residuals from spatial regression model no longer show autocorrelation
#use residuals from spatial error model

map.join$ZSpeak<-scale(residuals(err.peak))
```

```
## Warning: Method residuals.sarlm moved to the spatialreg package
```

```
## Warning in residuals.sarlm(err.peak): install the spatialreg package
```

### 4.4.2 Neuroticism

```
spt.neu<- lm(rz_neu~med_inc+perAA+perFem+perBA+perUrb+perHisp+voteO+vryrel, data = map.join)
moran.test(spt.neu$residuals, weightQ1, alternative="two.sided", zero.policy = TRUE)
```

```
## 
##  Moran I test under randomisation
## 
## data:  spt.neu$residuals  
## weights: weightQ1  n reduced by no-neighbour observations
##   
## 
## Moran I statistic standard deviate = 2.1213, p-value = 0.0339
## alternative hypothesis: two.sided
## sample estimates:
## Moran I statistic       Expectation          Variance 
##       0.186530528      -0.021276596       0.009596554
```

```
lm.LMtests(spt.neu, weightQ1, zero.policy = TRUE, test=c("LMerr", "LMlag", "RLMerr","RLMlag", "SARMA"))
```

```
## 
##  Lagrange multiplier diagnostics for spatial dependence
## 
## data:  
## model: lm(formula = rz_neu ~ med_inc + perAA + perFem + perBA + perUrb
## + perHisp + voteO + vryrel, data = map.join)
## weights: weightQ1
## 
## LMerr = 3.6326, df = 1, p-value = 0.05666
## 
## 
##  Lagrange multiplier diagnostics for spatial dependence
## 
## data:  
## model: lm(formula = rz_neu ~ med_inc + perAA + perFem + perBA + perUrb
## + perHisp + voteO + vryrel, data = map.join)
## weights: weightQ1
## 
## LMlag = 11.879, df = 1, p-value = 0.0005678
## 
## 
##  Lagrange multiplier diagnostics for spatial dependence
## 
## data:  
## model: lm(formula = rz_neu ~ med_inc + perAA + perFem + perBA + perUrb
## + perHisp + voteO + vryrel, data = map.join)
## weights: weightQ1
## 
## RLMerr = 4.3337, df = 1, p-value = 0.03736
## 
## 
##  Lagrange multiplier diagnostics for spatial dependence
## 
## data:  
## model: lm(formula = rz_neu ~ med_inc + perAA + perFem + perBA + perUrb
## + perHisp + voteO + vryrel, data = map.join)
## weights: weightQ1
## 
## RLMlag = 12.58, df = 1, p-value = 0.00039
## 
## 
##  Lagrange multiplier diagnostics for spatial dependence
## 
## data:  
## model: lm(formula = rz_neu ~ med_inc + perAA + perFem + perBA + perUrb
## + perHisp + voteO + vryrel, data = map.join)
## weights: weightQ1
## 
## SARMA = 16.212, df = 2, p-value = 0.0003017
```

```
#supports using spatial lag model
lag.neu<-lagsarlm(rz_neu~med_inc+perAA+perFem+perBA+perUrb+perHisp+voteO+vryrel, data = map.join, 
                  weightQ1, method="eigen", quiet = FALSE, zero.policy=TRUE, tol.solve=1.0e-15)
```

```
## Warning: Function lagsarlm moved to the spatialreg package
```

```
## Warning in lagsarlm(rz_neu ~ med_inc + perAA + perFem + perBA + perUrb + :
## install the spatialreg package
```

```
## Warning: Function can.be.simmed moved to the spatialreg package
```

```
## Warning in can.be.simmed(listw): install the spatialreg package
```

```
## 
## Spatial lag model
## Jacobian calculated using
```

```
## Warning: Function jacobianSetup moved to the spatialreg package
```

```
## Warning in jacobianSetup(method, env, con, pre_eig = con$pre_eig, trs = trs, :
## install the spatialreg package
```

```
## neighbourhood matrix eigenvalues
```

```
## Warning: Function eigen_setup moved to the spatialreg package
```

```
## Warning in eigen_setup(env, which = which): install the spatialreg package
```

```
## Computing eigenvalues ...
```

```
## Warning: Function as_dgRMatrix_listw moved to the spatialreg package
```

```
## Warning in as_dgRMatrix_listw(from): install the spatialreg package
```

```
## Warning: Function do_ldet moved to the spatialreg package
```

```
## Warning in do_ldet(rho, env): install the spatialreg package
```

```
## rho:  -0.4785762     function value:  88.51894
```

```
## Warning: Function do_ldet moved to the spatialreg package

## Warning: install the spatialreg package
```

```
## rho:  0.08618964     function value:  101.0266
```

```
## Warning: Function do_ldet moved to the spatialreg package

## Warning: install the spatialreg package
```

```
## rho:  0.4352341  function value:  105.7532
```

```
## Warning: Function do_ldet moved to the spatialreg package

## Warning: install the spatialreg package
```

```
## rho:  0.6509555  function value:  106.3609
```

```
## Warning: Function do_ldet moved to the spatialreg package

## Warning: install the spatialreg package
```

```
## rho:  0.6172643  function value:  106.4314
```

```
## Warning: Function do_ldet moved to the spatialreg package

## Warning: install the spatialreg package
```

```
## rho:  0.595313   function value:  106.4402
```

```
## Warning: Function do_ldet moved to the spatialreg package

## Warning: install the spatialreg package
```

```
## rho:  0.5996707  function value:  106.4407
```

```
## Warning: Function do_ldet moved to the spatialreg package

## Warning: install the spatialreg package
```

```
## rho:  0.5993797  function value:  106.4407
```

```
## Warning: Function do_ldet moved to the spatialreg package

## Warning: install the spatialreg package
```

```
## rho:  0.5994074  function value:  106.4407
```

```
## Warning: Function do_ldet moved to the spatialreg package

## Warning: install the spatialreg package
```

```
## rho:  0.5994077  function value:  106.4407
```

```
## Warning: Function do_ldet moved to the spatialreg package

## Warning: install the spatialreg package
```

```
## rho:  0.5994078  function value:  106.4407
```

```
## Warning: Function do_ldet moved to the spatialreg package

## Warning: install the spatialreg package
```

```
## rho:  0.5994078  function value:  106.4407
```

```
## Warning: Function do_ldet moved to the spatialreg package

## Warning: install the spatialreg package
```

```
## rho:  0.5994078  function value:  106.4407
```

```
## Warning in if (class(varb) == "try-error") {: the condition has length > 1 and
## only the first element will be used
```

```
summary(lag.neu)
```

```
## Warning: Method summary.sarlm moved to the spatialreg package
```

```
## Warning in summary.sarlm(lag.neu): install the spatialreg package
```

```
## Warning: Method Wald1.sarlm moved to the spatialreg package
```

```
## Warning in Wald1.sarlm(object): install the spatialreg package
```

```
## Warning: Method logLik.sarlm moved to the spatialreg package
```

```
## Warning in logLik.sarlm(object): install the spatialreg package
```

```
## Warning: Method residuals.sarlm moved to the spatialreg package
```

```
## Warning in residuals.sarlm(object): install the spatialreg package
```

```
## Warning: Method LR1.sarlm moved to the spatialreg package
```

```
## Warning in LR1.sarlm(object): install the spatialreg package
```

```
## Warning: Method logLik.sarlm moved to the spatialreg package
```

```
## Warning in logLik.sarlm(object): install the spatialreg package
```

```
## Warning: Method residuals.sarlm moved to the spatialreg package
```

```
## Warning in residuals.sarlm(object): install the spatialreg package
```

```
## Warning: Method print.summary.sarlm moved to the spatialreg package
```

```
## Warning in print.summary.sarlm(x): install the spatialreg package
```

```
## 
## Call:lagsarlm(formula = rz_neu ~ med_inc + perAA + perFem + perBA + 
##     perUrb + perHisp + voteO + vryrel, data = map.join, listw = weightQ1, 
##     method = "eigen", quiet = FALSE, zero.policy = TRUE, tol.solve = 1e-15)
## 
## Residuals:
```

```
## Warning: Method residuals.sarlm moved to the spatialreg package
```

```
## Warning in residuals.sarlm(x): install the spatialreg package
```

```
##         Min          1Q      Median          3Q         Max 
## -4.6812e-02 -2.3225e-02  2.5339e-05  1.6876e-02  7.4832e-02 
## 
## Type: lag 
## Regions with no neighbours included:
##  0 49 
## Coefficients: (asymptotic standard errors) 
##                Estimate  Std. Error z value  Pr(>|z|)
## (Intercept) -2.1325e+00  4.4833e-01 -4.7565 1.970e-06
## med_inc      2.7880e-06  1.1094e-06  2.5131 0.0119692
## perAA       -2.2304e-03  5.9702e-04 -3.7358 0.0001871
## perFem       4.5106e-02  8.9321e-03  5.0499 4.421e-07
## perBA       -6.2319e-03  1.6948e-03 -3.6771 0.0002359
## perUrb      -1.0571e-03  4.6059e-04 -2.2950 0.0217325
## perHisp      6.3060e-04  5.5050e-04  1.1455 0.2519962
## voteO       -5.5759e-04  5.9461e-04 -0.9377 0.3483795
## vryrel       1.7390e-05  6.2929e-04  0.0276 0.9779539
## 
## Rho: 0.59941, LR test value: 14.073, p-value: 0.00017582
## Asymptotic standard error: 0.11276
##     z-value: 5.3158, p-value: 1.0618e-07
## Wald statistic: 28.258, p-value: 1.0618e-07
```

```
## Warning: Method logLik.sarlm moved to the spatialreg package
```

```
## Warning in logLik.sarlm(x): install the spatialreg package
```

```
## Warning: Method residuals.sarlm moved to the spatialreg package
```

```
## Warning in residuals.sarlm(object): install the spatialreg package
```

```
## 
## Log likelihood: 106.4407 for lag model
## ML residual variance (sigma squared): 0.00074905, (sigma: 0.027369)
## Number of observations: 50 
## Number of parameters estimated: 11
```

```
## Warning: Method logLik.sarlm moved to the spatialreg package
```

```
## Warning in logLik.sarlm(object): install the spatialreg package
```

```
## Warning: Method residuals.sarlm moved to the spatialreg package
```

```
## Warning in residuals.sarlm(object): install the spatialreg package
```

```
## AIC: -190.88, (AIC for lm: -178.81)
## LM test for residual autocorrelation
## test value: 0.23722, p-value: 0.62622
```

```
moran.test(lag.neu$residuals, weightQ1, alternative="two.sided", zero.policy = TRUE)
```

```
## 
##  Moran I test under randomisation
## 
## data:  lag.neu$residuals  
## weights: weightQ1  n reduced by no-neighbour observations
##   
## 
## Moran I statistic standard deviate = -0.05085, p-value = 0.9594
## alternative hypothesis: two.sided
## sample estimates:
## Moran I statistic       Expectation          Variance 
##      -0.026224651      -0.021276596       0.009468622
```

```
#residuals from spatial regression model no longer show autocorrelation
#use residuals from spatial lag model

map.join$ZSneu<-scale(residuals(lag.neu))
```

```
## Warning: Method residuals.sarlm moved to the spatialreg package
```

```
## Warning in residuals.sarlm(lag.neu): install the spatialreg package
```

### 4.4.3 Female Neuroticism

```
spt.fneu<- lm(fzneuro~med_inc+perAA+perFem+perBA+perUrb+perHisp+voteO+vryrel, data = map.join)
moran.test(spt.fneu$residuals, weightQ1, alternative="two.sided", zero.policy = TRUE)
```

```
## 
##  Moran I test under randomisation
## 
## data:  spt.fneu$residuals  
## weights: weightQ1  n reduced by no-neighbour observations
##   
## 
## Moran I statistic standard deviate = 2.0581, p-value = 0.03958
## alternative hypothesis: two.sided
## sample estimates:
## Moran I statistic       Expectation          Variance 
##       0.179884093      -0.021276596       0.009553224
```

```
lm.LMtests(spt.fneu, weightQ1, zero.policy = TRUE, test=c("LMerr", "LMlag", "RLMerr","RLMlag", "SARMA"))
```

```
## 
##  Lagrange multiplier diagnostics for spatial dependence
## 
## data:  
## model: lm(formula = fzneuro ~ med_inc + perAA + perFem + perBA + perUrb
## + perHisp + voteO + vryrel, data = map.join)
## weights: weightQ1
## 
## LMerr = 3.3784, df = 1, p-value = 0.06606
## 
## 
##  Lagrange multiplier diagnostics for spatial dependence
## 
## data:  
## model: lm(formula = fzneuro ~ med_inc + perAA + perFem + perBA + perUrb
## + perHisp + voteO + vryrel, data = map.join)
## weights: weightQ1
## 
## LMlag = 14.248, df = 1, p-value = 0.0001602
## 
## 
##  Lagrange multiplier diagnostics for spatial dependence
## 
## data:  
## model: lm(formula = fzneuro ~ med_inc + perAA + perFem + perBA + perUrb
## + perHisp + voteO + vryrel, data = map.join)
## weights: weightQ1
## 
## RLMerr = 6.4982, df = 1, p-value = 0.0108
## 
## 
##  Lagrange multiplier diagnostics for spatial dependence
## 
## data:  
## model: lm(formula = fzneuro ~ med_inc + perAA + perFem + perBA + perUrb
## + perHisp + voteO + vryrel, data = map.join)
## weights: weightQ1
## 
## RLMlag = 17.368, df = 1, p-value = 3.08e-05
## 
## 
##  Lagrange multiplier diagnostics for spatial dependence
## 
## data:  
## model: lm(formula = fzneuro ~ med_inc + perAA + perFem + perBA + perUrb
## + perHisp + voteO + vryrel, data = map.join)
## weights: weightQ1
## 
## SARMA = 20.746, df = 2, p-value = 3.126e-05
```

```
#supports using spatial error model
err.fneu<-errorsarlm(fzneuro~med_inc+perAA+perFem+perBA+perUrb+perHisp+voteO+vryrel, data = map.join,
                     weightQ1, method="eigen", quiet = FALSE, zero.policy=TRUE, tol.solve=1.0e-15)
```

```
## Warning: Function errorsarlm moved to the spatialreg package
```

```
## Warning in errorsarlm(fzneuro ~ med_inc + perAA + perFem + perBA + perUrb + :
## install the spatialreg package
```

```
## 
## Spatial autoregressive error model
```

```
## Warning: Function can.be.simmed moved to the spatialreg package
```

```
## Warning in can.be.simmed(listw): install the spatialreg package
```

```
## 
## Jacobian calculated using
```

```
## Warning: Function jacobianSetup moved to the spatialreg package
```

```
## Warning in jacobianSetup(method, env, con, pre_eig = con$pre_eig, trs = trs, :
## install the spatialreg package
```

```
## neighbourhood matrix eigenvalues
```

```
## Warning: Function eigen_setup moved to the spatialreg package
```

```
## Warning in eigen_setup(env, which = which): install the spatialreg package
```

```
## Computing eigenvalues ...
```

```
## Warning: Function as_dgRMatrix_listw moved to the spatialreg package
```

```
## Warning in as_dgRMatrix_listw(from): install the spatialreg package
```

```
## Warning: Function do_ldet moved to the spatialreg package
```

```
## Warning in do_ldet(lambda, env): install the spatialreg package
```

```
## lambda: -0.4785762  function: 114.581  Jacobian: -1.219999  SSE: 0.02849878
```

```
## Warning: Function do_ldet moved to the spatialreg package

## Warning: install the spatialreg package
```

```
## lambda: 0.08618964  function: 120.3352  Jacobian: -0.04223568  SSE: 0.02373153
```

```
## Warning: Function do_ldet moved to the spatialreg package

## Warning: install the spatialreg package
```

```
## lambda: 0.4352341  function: 123.6771  Jacobian: -1.215038  SSE: 0.01981057
```

```
## Warning: Function do_ldet moved to the spatialreg package

## Warning: install the spatialreg package
```

```
## lambda: 0.6509555  function: 126.7521  Jacobian: -3.0968  SSE: 0.01624762
```

```
## Warning: Function do_ldet moved to the spatialreg package

## Warning: install the spatialreg package
```

```
## lambda: 0.7842786  function: 129.3836  Jacobian: -5.08969  SSE: 0.01350376
```

```
## Warning: Function do_ldet moved to the spatialreg package

## Warning: install the spatialreg package
```

```
## lambda: 0.8666769  function: 131.2615  Jacobian: -6.949411  SSE: 0.01162857
```

```
## Warning: Function do_ldet moved to the spatialreg package

## Warning: install the spatialreg package
```

```
## lambda: 0.9176018  function: 132.392  Jacobian: -8.585276  SSE: 0.01041044
```

```
## Warning: Function do_ldet moved to the spatialreg package

## Warning: install the spatialreg package
```

```
## lambda: 0.9490751  function: 132.7372  Jacobian: -9.980033  SSE: 0.00971054
```

```
## Warning: Function do_ldet moved to the spatialreg package

## Warning: install the spatialreg package
```

```
## lambda: 0.9735657  function: 132.2991  Jacobian: -11.52774  SSE: 0.009288952
```

```
## Warning: Function do_ldet moved to the spatialreg package

## Warning: install the spatialreg package
```

```
## lambda: 0.9439745  function: 132.7265  Jacobian: -9.721983  SSE: 0.009815499
```

```
## Warning: Function do_ldet moved to the spatialreg package

## Warning: install the spatialreg package
```

```
## lambda: 0.9496509  function: 132.7368  Jacobian: -10.01018  SSE: 0.009698986
```

```
## Warning: Function do_ldet moved to the spatialreg package

## Warning: install the spatialreg package
```

```
## lambda: 0.9486785  function: 132.7373  Jacobian: -9.959394  SSE: 0.009718534
```

```
## Warning: Function do_ldet moved to the spatialreg package

## Warning: install the spatialreg package
```

```
## lambda: 0.948716  function: 132.7373  Jacobian: -9.961342  SSE: 0.009717777
```

```
## Warning: Function do_ldet moved to the spatialreg package

## Warning: install the spatialreg package
```

```
## lambda: 0.9487142  function: 132.7373  Jacobian: -9.961246  SSE: 0.009717814
```

```
## Warning: Function do_ldet moved to the spatialreg package

## Warning: install the spatialreg package
```

```
## lambda: 0.9487142  function: 132.7373  Jacobian: -9.96125  SSE: 0.009717813
```

```
## Warning: Function do_ldet moved to the spatialreg package

## Warning: install the spatialreg package
```

```
## lambda: 0.9487143  function: 132.7373  Jacobian: -9.961251  SSE: 0.009717812
```

```
## Warning: Function do_ldet moved to the spatialreg package

## Warning: install the spatialreg package
```

```
## lambda: 0.9487142  function: 132.7373  Jacobian: -9.961249  SSE: 0.009717813
```

```
## Warning: Function do_ldet moved to the spatialreg package

## Warning: install the spatialreg package
```

```
## lambda: 0.9487142  function: 132.7373  Jacobian: -9.96125  SSE: 0.009717813
```

```
## Warning in if (class(asyvar1) == "try-error") {: the condition has length > 1
## and only the first element will be used
```

```
summary(err.fneu)
```

```
## Warning: Method summary.sarlm moved to the spatialreg package
```

```
## Warning in summary.sarlm(err.fneu): install the spatialreg package
```

```
## Warning: Method Wald1.sarlm moved to the spatialreg package
```

```
## Warning in Wald1.sarlm(object): install the spatialreg package
```

```
## Warning: Method logLik.sarlm moved to the spatialreg package
```

```
## Warning in logLik.sarlm(object): install the spatialreg package
```

```
## Warning: Method residuals.sarlm moved to the spatialreg package
```

```
## Warning in residuals.sarlm(object): install the spatialreg package
```

```
## Warning: Method LR1.sarlm moved to the spatialreg package
```

```
## Warning in LR1.sarlm(object): install the spatialreg package
```

```
## Warning: Method logLik.sarlm moved to the spatialreg package
```

```
## Warning in logLik.sarlm(object): install the spatialreg package
```

```
## Warning: Method residuals.sarlm moved to the spatialreg package
```

```
## Warning in residuals.sarlm(object): install the spatialreg package
```

```
## Warning: Method print.summary.sarlm moved to the spatialreg package
```

```
## Warning in print.summary.sarlm(x): install the spatialreg package
```

```
## 
## Call:errorsarlm(formula = fzneuro ~ med_inc + perAA + perFem + perBA + 
##     perUrb + perHisp + voteO + vryrel, data = map.join, listw = weightQ1, 
##     method = "eigen", quiet = FALSE, zero.policy = TRUE, tol.solve = 1e-15)
## 
## Residuals:
```

```
## Warning: Method residuals.sarlm moved to the spatialreg package
```

```
## Warning in residuals.sarlm(x): install the spatialreg package
```

```
##         Min          1Q      Median          3Q         Max 
## -0.02221990 -0.00610685 -0.00021236  0.01479283  0.03657923 
## 
## Type: error 
## Regions with no neighbours included:
##  0 49 
## Coefficients: (asymptotic standard errors) 
##                Estimate  Std. Error z value  Pr(>|z|)
## (Intercept)  5.1445e-01  3.9463e-01  1.3036 0.1923585
## med_inc     -5.2594e-07  7.1750e-07 -0.7330 0.4635418
## perAA        1.8546e-04  4.5828e-04  0.4047 0.6857091
## perFem      -7.6322e-03  7.8234e-03 -0.9756 0.3292824
## perBA       -3.1971e-03  9.4163e-04 -3.3953 0.0006854
## perUrb       9.4092e-05  2.9503e-04  0.3189 0.7497830
## perHisp      6.2065e-05  3.4854e-04  0.1781 0.8586683
## voteO       -5.1025e-04  3.0472e-04 -1.6745 0.0940305
## vryrel      -5.1722e-04  3.0073e-04 -1.7199 0.0854494
## 
## Lambda: 0.94871, LR test value: 26.337, p-value: 2.8677e-07
## Asymptotic standard error: 0.028195
##     z-value: 33.648, p-value: < 2.22e-16
## Wald statistic: 1132.2, p-value: < 2.22e-16
```

```
## Warning: Method logLik.sarlm moved to the spatialreg package
```

```
## Warning in logLik.sarlm(x): install the spatialreg package
```

```
## Warning: Method residuals.sarlm moved to the spatialreg package
```

```
## Warning in residuals.sarlm(object): install the spatialreg package
```

```
## 
## Log likelihood: 132.7373 for error model
## ML residual variance (sigma squared): 0.00019436, (sigma: 0.013941)
## Number of observations: 50 
## Number of parameters estimated: 11
```

```
## Warning: Method logLik.sarlm moved to the spatialreg package
```

```
## Warning in logLik.sarlm(object): install the spatialreg package
```

```
## Warning: Method residuals.sarlm moved to the spatialreg package
```

```
## Warning in residuals.sarlm(object): install the spatialreg package
```

```
## AIC: -243.47, (AIC for lm: -219.14)
```

```
moran.test(err.fneu$residuals, weightQ1, alternative="two.sided", zero.policy = TRUE)
```

```
## 
##  Moran I test under randomisation
## 
## data:  err.fneu$residuals  
## weights: weightQ1  n reduced by no-neighbour observations
##   
## 
## Moran I statistic standard deviate = -1.0633, p-value = 0.2877
## alternative hypothesis: two.sided
## sample estimates:
## Moran I statistic       Expectation          Variance 
##      -0.125061741      -0.021276596       0.009527579
```

```
#residuals from spatial regression model no longer show autocorrelation
#use residuals from spatial error model

map.join$ZSfneu<-scale(residuals(err.fneu))
```

```
## Warning: Method residuals.sarlm moved to the spatialreg package
```

```
## Warning in residuals.sarlm(err.fneu): install the spatialreg package
```

### 4.4.4 Age at First Birth

```
spt.ageFB<-lm(ageFB~med_inc+perAA+perFem+perBA+perUrb+perHisp+voteO+vryrel, data=map.join)
moran.test(spt.ageFB$residuals, weightQ1, alternative="two.sided", zero.policy = TRUE)
```

```
## 
##  Moran I test under randomisation
## 
## data:  spt.ageFB$residuals  
## weights: weightQ1  n reduced by no-neighbour observations
##   
## 
## Moran I statistic standard deviate = 2.4223, p-value = 0.01542
## alternative hypothesis: two.sided
## sample estimates:
## Moran I statistic       Expectation          Variance 
##       0.212584213      -0.021276596       0.009320917
```

```
lm.LMtests(spt.ageFB,weightQ1, zero.policy = TRUE, test=c("LMerr", "LMlag", "RLMerr","RLMlag", "SARMA"))
```

```
## 
##  Lagrange multiplier diagnostics for spatial dependence
## 
## data:  
## model: lm(formula = ageFB ~ med_inc + perAA + perFem + perBA + perUrb +
## perHisp + voteO + vryrel, data = map.join)
## weights: weightQ1
## 
## LMerr = 4.7183, df = 1, p-value = 0.02984
## 
## 
##  Lagrange multiplier diagnostics for spatial dependence
## 
## data:  
## model: lm(formula = ageFB ~ med_inc + perAA + perFem + perBA + perUrb +
## perHisp + voteO + vryrel, data = map.join)
## weights: weightQ1
## 
## LMlag = 0.66063, df = 1, p-value = 0.4163
## 
## 
##  Lagrange multiplier diagnostics for spatial dependence
## 
## data:  
## model: lm(formula = ageFB ~ med_inc + perAA + perFem + perBA + perUrb +
## perHisp + voteO + vryrel, data = map.join)
## weights: weightQ1
## 
## RLMerr = 4.4498, df = 1, p-value = 0.0349
## 
## 
##  Lagrange multiplier diagnostics for spatial dependence
## 
## data:  
## model: lm(formula = ageFB ~ med_inc + perAA + perFem + perBA + perUrb +
## perHisp + voteO + vryrel, data = map.join)
## weights: weightQ1
## 
## RLMlag = 0.39217, df = 1, p-value = 0.5312
## 
## 
##  Lagrange multiplier diagnostics for spatial dependence
## 
## data:  
## model: lm(formula = ageFB ~ med_inc + perAA + perFem + perBA + perUrb +
## perHisp + voteO + vryrel, data = map.join)
## weights: weightQ1
## 
## SARMA = 5.1105, df = 2, p-value = 0.07767
```

```
#supports using spatial error model
err.ageFB<-errorsarlm(ageFB~med_inc+perAA+perFem+perBA+perUrb+perHisp+voteO+vryrel, data=map.join, 
                      weightQ1,method="eigen",quiet=F,zero.policy=T,tol.solve=1.0e-15)
```

```
## Warning: Function errorsarlm moved to the spatialreg package
```

```
## Warning in errorsarlm(ageFB ~ med_inc + perAA + perFem + perBA + perUrb + :
## install the spatialreg package
```

```
## 
## Spatial autoregressive error model
```

```
## Warning: Function can.be.simmed moved to the spatialreg package
```

```
## Warning in can.be.simmed(listw): install the spatialreg package
```

```
## 
## Jacobian calculated using
```

```
## Warning: Function jacobianSetup moved to the spatialreg package
```

```
## Warning in jacobianSetup(method, env, con, pre_eig = con$pre_eig, trs = trs, :
## install the spatialreg package
```

```
## neighbourhood matrix eigenvalues
```

```
## Warning: Function eigen_setup moved to the spatialreg package
```

```
## Warning in eigen_setup(env, which = which): install the spatialreg package
```

```
## Computing eigenvalues ...
```

```
## Warning: Function as_dgRMatrix_listw moved to the spatialreg package
```

```
## Warning in as_dgRMatrix_listw(from): install the spatialreg package
```

```
## Warning: Function do_ldet moved to the spatialreg package
```

```
## Warning in do_ldet(lambda, env): install the spatialreg package
```

```
## lambda: -0.4785762  function: -36.27282  Jacobian: -1.219999  SSE: 11.89667
```

```
## Warning: Function do_ldet moved to the spatialreg package

## Warning: install the spatialreg package
```

```
## lambda: 0.08618964  function: -29.23234  Jacobian: -0.04223568  SSE: 9.409784
```

```
## Warning: Function do_ldet moved to the spatialreg package

## Warning: install the spatialreg package
```

```
## lambda: 0.4352341  function: -25.99729  Jacobian: -1.215038  SSE: 7.888736
```

```
## Warning: Function do_ldet moved to the spatialreg package

## Warning: install the spatialreg package
```

```
## lambda: 0.6509555  function: -23.836  Jacobian: -3.0968  SSE: 6.710773
```

```
## Warning: Function do_ldet moved to the spatialreg package

## Warning: install the spatialreg package
```

```
## lambda: 0.7842786  function: -22.44505  Jacobian: -5.08969  SSE: 5.861236
```

```
## Warning: Function do_ldet moved to the spatialreg package

## Warning: install the spatialreg package
```

```
## lambda: 0.8666769  function: -21.91823  Jacobian: -6.949411  SSE: 5.327588
```

```
## Warning: Function do_ldet moved to the spatialreg package

## Warning: install the spatialreg package
```

```
## lambda: 0.9176018  function: -22.01748  Jacobian: -8.585276  SSE: 5.009991
```

```
## Warning: Function do_ldet moved to the spatialreg package

## Warning: install the spatialreg package
```

```
## lambda: 0.8765659  function: -21.89954  Jacobian: -7.227524  SSE: 5.264712
```

```
## Warning: Function do_ldet moved to the spatialreg package

## Warning: install the spatialreg package
```

```
## lambda: 0.8817227  function: -21.89558  Jacobian: -7.378937  SSE: 5.232093
```

```
## Warning: Function do_ldet moved to the spatialreg package

## Warning: install the spatialreg package
```

```
## lambda: 0.8843005  function: -21.89523  Jacobian: -7.456386  SSE: 5.215837
```

```
## Warning: Function do_ldet moved to the spatialreg package

## Warning: install the spatialreg package
```

```
## lambda: 0.8838394  function: -21.89521  Jacobian: -7.442445  SSE: 5.218742
```

```
## Warning: Function do_ldet moved to the spatialreg package

## Warning: install the spatialreg package
```

```
## lambda: 0.8838069  function: -21.89521  Jacobian: -7.441462  SSE: 5.218947
```

```
## Warning: Function do_ldet moved to the spatialreg package

## Warning: install the spatialreg package
```

```
## lambda: 0.8838099  function: -21.89521  Jacobian: -7.441554  SSE: 5.218928
```

```
## Warning: Function do_ldet moved to the spatialreg package

## Warning: install the spatialreg package
```

```
## lambda: 0.8838099  function: -21.89521  Jacobian: -7.441553  SSE: 5.218928
```

```
## Warning: Function do_ldet moved to the spatialreg package

## Warning: install the spatialreg package
```

```
## lambda: 0.8838094  function: -21.89521  Jacobian: -7.441538  SSE: 5.218931
```

```
## Warning: Function do_ldet moved to the spatialreg package

## Warning: install the spatialreg package
```

```
## lambda: 0.8838097  function: -21.89521  Jacobian: -7.441547  SSE: 5.218929
```

```
## Warning: Function do_ldet moved to the spatialreg package

## Warning: install the spatialreg package
```

```
## lambda: 0.8838098  function: -21.89521  Jacobian: -7.441551  SSE: 5.218929
```

```
## Warning: Function do_ldet moved to the spatialreg package

## Warning: install the spatialreg package
```

```
## lambda: 0.8838099  function: -21.89521  Jacobian: -7.441552  SSE: 5.218928
```

```
## Warning: Function do_ldet moved to the spatialreg package

## Warning: install the spatialreg package
```

```
## lambda: 0.8838099  function: -21.89521  Jacobian: -7.441554  SSE: 5.218928
```

```
## Warning: Function do_ldet moved to the spatialreg package

## Warning: install the spatialreg package
```

```
## lambda: 0.8838099  function: -21.89521  Jacobian: -7.441553  SSE: 5.218928
```

```
## Warning in if (class(asyvar1) == "try-error") {: the condition has length > 1
## and only the first element will be used
```

```
summary(err.ageFB)
```

```
## Warning: Method summary.sarlm moved to the spatialreg package
```

```
## Warning in summary.sarlm(err.ageFB): install the spatialreg package
```

```
## Warning: Method Wald1.sarlm moved to the spatialreg package
```

```
## Warning in Wald1.sarlm(object): install the spatialreg package
```

```
## Warning: Method logLik.sarlm moved to the spatialreg package
```

```
## Warning in logLik.sarlm(object): install the spatialreg package
```

```
## Warning: Method residuals.sarlm moved to the spatialreg package
```

```
## Warning in residuals.sarlm(object): install the spatialreg package
```

```
## Warning: Method LR1.sarlm moved to the spatialreg package
```

```
## Warning in LR1.sarlm(object): install the spatialreg package
```

```
## Warning: Method logLik.sarlm moved to the spatialreg package
```

```
## Warning in logLik.sarlm(object): install the spatialreg package
```

```
## Warning: Method residuals.sarlm moved to the spatialreg package
```

```
## Warning in residuals.sarlm(object): install the spatialreg package
```

```
## Warning: Method print.summary.sarlm moved to the spatialreg package
```

```
## Warning in print.summary.sarlm(x): install the spatialreg package
```

```
## 
## Call:errorsarlm(formula = ageFB ~ med_inc + perAA + perFem + perBA + 
##     perUrb + perHisp + voteO + vryrel, data = map.join, listw = weightQ1, 
##     method = "eigen", quiet = F, zero.policy = T, tol.solve = 1e-15)
## 
## Residuals:
```

```
## Warning: Method residuals.sarlm moved to the spatialreg package
```

```
## Warning in residuals.sarlm(x): install the spatialreg package
```

```
##       Min        1Q    Median        3Q       Max 
## -0.872289 -0.144002  0.075069  0.227498  0.773008 
## 
## Type: error 
## Regions with no neighbours included:
##  0 49 
## Coefficients: (asymptotic standard errors) 
##                Estimate  Std. Error z value  Pr(>|z|)
## (Intercept)  2.4102e+01  8.7257e+00  2.7622  0.005741
## med_inc      3.2822e-06  1.6449e-05  0.1995  0.841841
## perAA       -1.7094e-02  1.0422e-02 -1.6402  0.100957
## perFem      -8.1044e-02  1.7157e-01 -0.4724  0.636667
## perBA        1.3174e-01  2.1714e-02  6.0671 1.303e-09
## perUrb       1.8643e-02  6.7319e-03  2.7693  0.005618
## perHisp     -5.6405e-03  7.8625e-03 -0.7174  0.473135
## voteO        1.1780e-03  6.9442e-03  0.1696  0.865291
## vryrel      -9.0266e-03  7.0015e-03 -1.2892  0.197313
## 
## Lambda: 0.88381, LR test value: 16.449, p-value: 4.998e-05
## Asymptotic standard error: 0.053229
##     z-value: 16.604, p-value: < 2.22e-16
## Wald statistic: 275.69, p-value: < 2.22e-16
```

```
## Warning: Method logLik.sarlm moved to the spatialreg package
```

```
## Warning in logLik.sarlm(x): install the spatialreg package
```

```
## Warning: Method residuals.sarlm moved to the spatialreg package
```

```
## Warning in residuals.sarlm(object): install the spatialreg package
```

```
## 
## Log likelihood: -21.89521 for error model
## ML residual variance (sigma squared): 0.10438, (sigma: 0.32308)
## Number of observations: 50 
## Number of parameters estimated: 11
```

```
## Warning: Method logLik.sarlm moved to the spatialreg package
```

```
## Warning in logLik.sarlm(object): install the spatialreg package
```

```
## Warning: Method residuals.sarlm moved to the spatialreg package
```

```
## Warning in residuals.sarlm(object): install the spatialreg package
```

```
## AIC: 65.79, (AIC for lm: 80.239)
```

```
moran.test(err.ageFB$residuals,weightQ1,alternative="two.sided",zero.policy=T)
```

```
## 
##  Moran I test under randomisation
## 
## data:  err.ageFB$residuals  
## weights: weightQ1  n reduced by no-neighbour observations
##   
## 
## Moran I statistic standard deviate = 0.44724, p-value = 0.6547
## alternative hypothesis: two.sided
## sample estimates:
## Moran I statistic       Expectation          Variance 
##        0.02187509       -0.02127660        0.00930911
```

```
#residuals from spatial regression model no longer show autocorrelation
#use residuals from spatial error model

map.join$ZSafb<-scale(residuals(err.ageFB))
```

```
## Warning: Method residuals.sarlm moved to the spatialreg package
```

```
## Warning in residuals.sarlm(err.ageFB): install the spatialreg package
```

### 4.4.5 Percent Never Married

```
spt.nvm<-lm(nevermar~med_inc+perAA+perFem+perBA+perUrb+perHisp+voteO+vryrel, data=map.join)
moran.test(spt.nvm$residuals, weightQ1, alternative="two.sided", zero.policy = TRUE)
```

```
## 
##  Moran I test under randomisation
## 
## data:  spt.nvm$residuals  
## weights: weightQ1  n reduced by no-neighbour observations
##   
## 
## Moran I statistic standard deviate = 2.0043, p-value = 0.04504
## alternative hypothesis: two.sided
## sample estimates:
## Moran I statistic       Expectation          Variance 
##       0.174336803      -0.021276596       0.009524978
```

```
lm.LMtests(spt.nvm,weightQ1, zero.policy = TRUE, test=c("LMerr", "LMlag", "RLMerr","RLMlag", "SARMA"))
```

```
## 
##  Lagrange multiplier diagnostics for spatial dependence
## 
## data:  
## model: lm(formula = nevermar ~ med_inc + perAA + perFem + perBA +
## perUrb + perHisp + voteO + vryrel, data = map.join)
## weights: weightQ1
## 
## LMerr = 3.1732, df = 1, p-value = 0.07485
## 
## 
##  Lagrange multiplier diagnostics for spatial dependence
## 
## data:  
## model: lm(formula = nevermar ~ med_inc + perAA + perFem + perBA +
## perUrb + perHisp + voteO + vryrel, data = map.join)
## weights: weightQ1
## 
## LMlag = 1.5669, df = 1, p-value = 0.2107
## 
## 
##  Lagrange multiplier diagnostics for spatial dependence
## 
## data:  
## model: lm(formula = nevermar ~ med_inc + perAA + perFem + perBA +
## perUrb + perHisp + voteO + vryrel, data = map.join)
## weights: weightQ1
## 
## RLMerr = 4.9085, df = 1, p-value = 0.02673
## 
## 
##  Lagrange multiplier diagnostics for spatial dependence
## 
## data:  
## model: lm(formula = nevermar ~ med_inc + perAA + perFem + perBA +
## perUrb + perHisp + voteO + vryrel, data = map.join)
## weights: weightQ1
## 
## RLMlag = 3.3021, df = 1, p-value = 0.06919
## 
## 
##  Lagrange multiplier diagnostics for spatial dependence
## 
## data:  
## model: lm(formula = nevermar ~ med_inc + perAA + perFem + perBA +
## perUrb + perHisp + voteO + vryrel, data = map.join)
## weights: weightQ1
## 
## SARMA = 6.4753, df = 2, p-value = 0.03926
```

```
#supports spatial error model
err.nvrmarr<-errorsarlm(nevermar~med_inc+perAA+perFem+perBA+perUrb+perHisp+voteO+vryrel, data=map.join,
                        weightQ1,method="eigen",quiet=F,zero.policy=T,tol.solve=1.0e-15)
```

```
## Warning: Function errorsarlm moved to the spatialreg package
```

```
## Warning in errorsarlm(nevermar ~ med_inc + perAA + perFem + perBA + perUrb + :
## install the spatialreg package
```

```
## 
## Spatial autoregressive error model
```

```
## Warning: Function can.be.simmed moved to the spatialreg package
```

```
## Warning in can.be.simmed(listw): install the spatialreg package
```

```
## 
## Jacobian calculated using
```

```
## Warning: Function jacobianSetup moved to the spatialreg package
```

```
## Warning in jacobianSetup(method, env, con, pre_eig = con$pre_eig, trs = trs, :
## install the spatialreg package
```

```
## neighbourhood matrix eigenvalues
```

```
## Warning: Function eigen_setup moved to the spatialreg package
```

```
## Warning in eigen_setup(env, which = which): install the spatialreg package
```

```
## Computing eigenvalues ...
```

```
## Warning: Function as_dgRMatrix_listw moved to the spatialreg package
```

```
## Warning in as_dgRMatrix_listw(from): install the spatialreg package
```

```
## Warning: Function do_ldet moved to the spatialreg package
```

```
## Warning in do_ldet(lambda, env): install the spatialreg package
```

```
## lambda: -0.4785762  function: 126.1094  Jacobian: -1.219999  SSE: 0.01797041
```

```
## Warning: Function do_ldet moved to the spatialreg package

## Warning: install the spatialreg package
```

```
## lambda: 0.08618964  function: 132.5563  Jacobian: -0.04223568  SSE: 0.01455534
```

```
## Warning: Function do_ldet moved to the spatialreg package

## Warning: install the spatialreg package
```

```
## lambda: 0.4352341  function: 134.5741  Jacobian: -1.215038  SSE: 0.0128114
```

```
## Warning: Function do_ldet moved to the spatialreg package

## Warning: install the spatialreg package
```

```
## lambda: 0.7294846  function: 135.7947  Jacobian: -4.158972  SSE: 0.01084555
```

```
## Warning: Function do_ldet moved to the spatialreg package

## Warning: install the spatialreg package
```

```
## lambda: 0.6170909  function: 135.3278  Jacobian: -2.714454  SSE: 0.01170727
```

```
## Warning: Function do_ldet moved to the spatialreg package

## Warning: install the spatialreg package
```

```
## lambda: 0.8328123  function: 136.4071  Jacobian: -6.099368  SSE: 0.009792749
```

```
## Warning: Function do_ldet moved to the spatialreg package

## Warning: install the spatialreg package
```

```
## lambda: 0.8966723  function: 137.1013  Jacobian: -7.845955  SSE: 0.008881847
```

```
## Warning: Function do_ldet moved to the spatialreg package

## Warning: install the spatialreg package
```

```
## lambda: 0.93614  function: 137.4655  Jacobian: -9.353419  SSE: 0.008241174
```

```
## Warning: Function do_ldet moved to the spatialreg package

## Warning: install the spatialreg package
```

```
## lambda: 0.9605323  function: 137.2205  Jacobian: -10.62646  SSE: 0.007909175
```

```
## Warning: Function do_ldet moved to the spatialreg package

## Warning: install the spatialreg package
```

```
## lambda: 0.9316932  function: 137.4515  Jacobian: -9.157085  SSE: 0.00831082
```

```
## Warning: Function do_ldet moved to the spatialreg package

## Warning: install the spatialreg package
```

```
## lambda: 0.9373666  function: 137.4672  Jacobian: -9.409126  SSE: 0.008222292
```

```
## Warning: Function do_ldet moved to the spatialreg package

## Warning: install the spatialreg package
```

```
## lambda: 0.9388343  function: 137.4677  Jacobian: -9.476715  SSE: 0.008199911
```

```
## Warning: Function do_ldet moved to the spatialreg package

## Warning: install the spatialreg package
```

```
## lambda: 0.9386275  function: 137.4678  Jacobian: -9.467127  SSE: 0.00820305
```

```
## Warning: Function do_ldet moved to the spatialreg package

## Warning: install the spatialreg package
```

```
## lambda: 0.938609  function: 137.4678  Jacobian: -9.466269  SSE: 0.008203332
```

```
## Warning: Function do_ldet moved to the spatialreg package

## Warning: install the spatialreg package
```

```
## lambda: 0.9386111  function: 137.4678  Jacobian: -9.466368  SSE: 0.008203299
```

```
## Warning: Function do_ldet moved to the spatialreg package

## Warning: install the spatialreg package
```

```
## lambda: 0.9386111  function: 137.4678  Jacobian: -9.466367  SSE: 0.0082033
```

```
## Warning: Function do_ldet moved to the spatialreg package

## Warning: install the spatialreg package
```

```
## lambda: 0.9386111  function: 137.4678  Jacobian: -9.466366  SSE: 0.0082033
```

```
## Warning: Function do_ldet moved to the spatialreg package

## Warning: install the spatialreg package
```

```
## lambda: 0.9386111  function: 137.4678  Jacobian: -9.466367  SSE: 0.0082033
```

```
## Warning in if (class(asyvar1) == "try-error") {: the condition has length > 1
## and only the first element will be used
```

```
summary(err.nvrmarr)
```

```
## Warning: Method summary.sarlm moved to the spatialreg package
```

```
## Warning in summary.sarlm(err.nvrmarr): install the spatialreg package
```

```
## Warning: Method Wald1.sarlm moved to the spatialreg package
```

```
## Warning in Wald1.sarlm(object): install the spatialreg package
```

```
## Warning: Method logLik.sarlm moved to the spatialreg package
```

```
## Warning in logLik.sarlm(object): install the spatialreg package
```

```
## Warning: Method residuals.sarlm moved to the spatialreg package
```

```
## Warning in residuals.sarlm(object): install the spatialreg package
```

```
## Warning: Method LR1.sarlm moved to the spatialreg package
```

```
## Warning in LR1.sarlm(object): install the spatialreg package
```

```
## Warning: Method logLik.sarlm moved to the spatialreg package
```

```
## Warning in logLik.sarlm(object): install the spatialreg package
```

```
## Warning: Method residuals.sarlm moved to the spatialreg package
```

```
## Warning in residuals.sarlm(object): install the spatialreg package
```

```
## Warning: Method print.summary.sarlm moved to the spatialreg package
```

```
## Warning in print.summary.sarlm(x): install the spatialreg package
```

```
## 
## Call:errorsarlm(formula = nevermar ~ med_inc + perAA + perFem + perBA + 
##     perUrb + perHisp + voteO + vryrel, data = map.join, listw = weightQ1, 
##     method = "eigen", quiet = F, zero.policy = T, tol.solve = 1e-15)
## 
## Residuals:
```

```
## Warning: Method residuals.sarlm moved to the spatialreg package
```

```
## Warning in residuals.sarlm(x): install the spatialreg package
```

```
##        Min         1Q     Median         3Q        Max 
## -0.0311574 -0.0093996 -0.0047650  0.0048663  0.0339813 
## 
## Type: error 
## Regions with no neighbours included:
##  0 49 
## Coefficients: (asymptotic standard errors) 
##                Estimate  Std. Error z value  Pr(>|z|)
## (Intercept)  4.4750e-01  3.6025e-01  1.2422  0.214161
## med_inc     -1.7638e-06  6.5851e-07 -2.6784  0.007397
## perAA        2.6703e-03  4.1999e-04  6.3580 2.044e-10
## perFem      -3.2495e-03  7.1333e-03 -0.4555  0.648716
## perBA        2.1738e-03  8.6472e-04  2.5139  0.011940
## perUrb       7.0993e-04  2.7048e-04  2.6247  0.008672
## perHisp      9.6808e-04  3.1885e-04  3.0362  0.002396
## voteO        1.4083e-04  2.7938e-04  0.5041  0.614194
## vryrel       1.1237e-04  2.7653e-04  0.4064  0.684475
## 
## Lambda: 0.93861, LR test value: 11.229, p-value: 0.00080537
## Asymptotic standard error: 0.032539
##     z-value: 28.846, p-value: < 2.22e-16
## Wald statistic: 832.07, p-value: < 2.22e-16
```

```
## Warning: Method logLik.sarlm moved to the spatialreg package
```

```
## Warning in logLik.sarlm(x): install the spatialreg package
```

```
## Warning: Method residuals.sarlm moved to the spatialreg package
```

```
## Warning in residuals.sarlm(object): install the spatialreg package
```

```
## 
## Log likelihood: 137.4678 for error model
## ML residual variance (sigma squared): 0.00016407, (sigma: 0.012809)
## Number of observations: 50 
## Number of parameters estimated: 11
```

```
## Warning: Method logLik.sarlm moved to the spatialreg package
```

```
## Warning in logLik.sarlm(object): install the spatialreg package
```

```
## Warning: Method residuals.sarlm moved to the spatialreg package
```

```
## Warning in residuals.sarlm(object): install the spatialreg package
```

```
## AIC: -252.94, (AIC for lm: -243.71)
```

```
moran.test(err.nvrmarr$residuals,weightQ1,alternative="two.sided",zero.policy=T)
```

```
## 
##  Moran I test under randomisation
## 
## data:  err.nvrmarr$residuals  
## weights: weightQ1  n reduced by no-neighbour observations
##   
## 
## Moran I statistic standard deviate = -1.5194, p-value = 0.1287
## alternative hypothesis: two.sided
## sample estimates:
## Moran I statistic       Expectation          Variance 
##      -0.167881900      -0.021276596       0.009310673
```

```
#residuals from spatial regression model no longer show autocorrelation
#use residuals from spatial error model

map.join$ZSnvm<-scale(residuals(err.nvrmarr))
```

```
## Warning: Method residuals.sarlm moved to the spatialreg package
```

```
## Warning in residuals.sarlm(err.nvrmarr): install the spatialreg package
```

### 4.4.6 Divorce

```
spt.div<-lm(divorce~med_inc+perAA+perFem+perBA+perUrb+perHisp+voteO+vryrel, data=map.join)
moran.test(spt.div$residuals, weightQ1, alternative="two.sided", zero.policy = TRUE)
```

```
## 
##  Moran I test under randomisation
## 
## data:  spt.div$residuals  
## weights: weightQ1  n reduced by no-neighbour observations
##   
## 
## Moran I statistic standard deviate = 3.4038, p-value = 0.0006646
## alternative hypothesis: two.sided
## sample estimates:
## Moran I statistic       Expectation          Variance 
##       0.313508070      -0.021276596       0.009674109
```

```
lm.LMtests(spt.div,weightQ1, zero.policy = TRUE, test=c("LMerr", "LMlag", "RLMerr","RLMlag", "SARMA"))
```

```
## 
##  Lagrange multiplier diagnostics for spatial dependence
## 
## data:  
## model: lm(formula = divorce ~ med_inc + perAA + perFem + perBA + perUrb
## + perHisp + voteO + vryrel, data = map.join)
## weights: weightQ1
## 
## LMerr = 10.262, df = 1, p-value = 0.001358
## 
## 
##  Lagrange multiplier diagnostics for spatial dependence
## 
## data:  
## model: lm(formula = divorce ~ med_inc + perAA + perFem + perBA + perUrb
## + perHisp + voteO + vryrel, data = map.join)
## weights: weightQ1
## 
## LMlag = 3.2868, df = 1, p-value = 0.06984
## 
## 
##  Lagrange multiplier diagnostics for spatial dependence
## 
## data:  
## model: lm(formula = divorce ~ med_inc + perAA + perFem + perBA + perUrb
## + perHisp + voteO + vryrel, data = map.join)
## weights: weightQ1
## 
## RLMerr = 7.0941, df = 1, p-value = 0.007734
## 
## 
##  Lagrange multiplier diagnostics for spatial dependence
## 
## data:  
## model: lm(formula = divorce ~ med_inc + perAA + perFem + perBA + perUrb
## + perHisp + voteO + vryrel, data = map.join)
## weights: weightQ1
## 
## RLMlag = 0.11914, df = 1, p-value = 0.73
## 
## 
##  Lagrange multiplier diagnostics for spatial dependence
## 
## data:  
## model: lm(formula = divorce ~ med_inc + perAA + perFem + perBA + perUrb
## + perHisp + voteO + vryrel, data = map.join)
## weights: weightQ1
## 
## SARMA = 10.381, df = 2, p-value = 0.00557
```

```
#supports spatial error model
err.div<-errorsarlm(divorce~med_inc+perAA+perFem+perBA+perUrb+perHisp+voteO+vryrel, data=map.join, 
                    weightQ1,method="eigen",quiet=F,zero.policy=T,tol.solve=1.0e-15)
```

```
## Warning: Function errorsarlm moved to the spatialreg package
```

```
## Warning in errorsarlm(divorce ~ med_inc + perAA + perFem + perBA + perUrb + :
## install the spatialreg package
```

```
## 
## Spatial autoregressive error model
```

```
## Warning: Function can.be.simmed moved to the spatialreg package
```

```
## Warning in can.be.simmed(listw): install the spatialreg package
```

```
## 
## Jacobian calculated using
```

```
## Warning: Function jacobianSetup moved to the spatialreg package
```

```
## Warning in jacobianSetup(method, env, con, pre_eig = con$pre_eig, trs = trs, :
## install the spatialreg package
```

```
## neighbourhood matrix eigenvalues
```

```
## Warning: Function eigen_setup moved to the spatialreg package
```

```
## Warning in eigen_setup(env, which = which): install the spatialreg package
```

```
## Computing eigenvalues ...
```

```
## Warning: Function as_dgRMatrix_listw moved to the spatialreg package
```

```
## Warning in as_dgRMatrix_listw(from): install the spatialreg package
```

```
## Warning: Function do_ldet moved to the spatialreg package
```

```
## Warning in do_ldet(lambda, env): install the spatialreg package
```

```
## lambda: -0.4785762  function: 240.5771  Jacobian: -1.219999  SSE: 0.0001845224
```

```
## Warning: Function do_ldet moved to the spatialreg package

## Warning: install the spatialreg package
```

```
## lambda: 0.08618964  function: 250.2674  Jacobian: -0.04223568  SSE: 0.0001312719
```

```
## Warning: Function do_ldet moved to the spatialreg package

## Warning: install the spatialreg package
```

```
## lambda: 0.4352341  function: 254.6404  Jacobian: -1.215038  SSE: 0.0001051554
```

```
## Warning: Function do_ldet moved to the spatialreg package

## Warning: install the spatialreg package
```

```
## lambda: 0.6509555  function: 256.0534  Jacobian: -3.0968  SSE: 9.21711e-05
```

```
## Warning: Function do_ldet moved to the spatialreg package

## Warning: install the spatialreg package
```

```
## lambda: 0.8525076  function: 255.4256  Jacobian: -6.576055  SSE: 8.223583e-05
```

```
## Warning: Function do_ldet moved to the spatialreg package

## Warning: install the spatialreg package
```

```
## lambda: 0.6844888  function: 256.123  Jacobian: -3.517415  SSE: 9.038152e-05
```

```
## Warning: Function do_ldet moved to the spatialreg package

## Warning: install the spatialreg package
```

```
## lambda: 0.7013011  function: 256.1376  Jacobian: -3.745971  SSE: 8.950671e-05
```

```
## Warning: Function do_ldet moved to the spatialreg package

## Warning: install the spatialreg package
```

```
## lambda: 0.7110251  function: 256.1394  Jacobian: -3.884058  SSE: 8.900738e-05
```

```
## Warning: Function do_ldet moved to the spatialreg package

## Warning: install the spatialreg package
```

```
## lambda: 0.7096862  function: 256.1394  Jacobian: -3.864778  SSE: 8.907585e-05
```

```
## Warning: Function do_ldet moved to the spatialreg package

## Warning: install the spatialreg package
```

```
## lambda: 0.7095702  function: 256.1394  Jacobian: -3.863112  SSE: 8.908178e-05
```

```
## Warning: Function do_ldet moved to the spatialreg package

## Warning: install the spatialreg package
```

```
## lambda: 0.7095789  function: 256.1394  Jacobian: -3.863238  SSE: 8.908133e-05
```

```
## Warning: Function do_ldet moved to the spatialreg package

## Warning: install the spatialreg package
```

```
## lambda: 0.7095788  function: 256.1394  Jacobian: -3.863236  SSE: 8.908134e-05
```

```
## Warning: Function do_ldet moved to the spatialreg package

## Warning: install the spatialreg package
```

```
## lambda: 0.7095788  function: 256.1394  Jacobian: -3.863236  SSE: 8.908134e-05
```

```
## Warning: Function do_ldet moved to the spatialreg package

## Warning: install the spatialreg package
```

```
## lambda: 0.7095788  function: 256.1394  Jacobian: -3.863236  SSE: 8.908134e-05
```

```
## Warning: Function do_ldet moved to the spatialreg package

## Warning: install the spatialreg package
```

```
## lambda: 0.7095789  function: 256.1394  Jacobian: -3.863237  SSE: 8.908134e-05
```

```
## Warning: Function do_ldet moved to the spatialreg package

## Warning: install the spatialreg package
```

```
## lambda: 0.7095788  function: 256.1394  Jacobian: -3.863236  SSE: 8.908134e-05
```

```
## Warning: Function do_ldet moved to the spatialreg package

## Warning: install the spatialreg package
```

```
## lambda: 0.7095788  function: 256.1394  Jacobian: -3.863236  SSE: 8.908134e-05
```

```
## Warning in if (class(asyvar1) == "try-error") {: the condition has length > 1
## and only the first element will be used
```

```
summary(err.div)
```

```
## Warning: Method summary.sarlm moved to the spatialreg package
```

```
## Warning in summary.sarlm(err.div): install the spatialreg package
```

```
## Warning: Method Wald1.sarlm moved to the spatialreg package
```

```
## Warning in Wald1.sarlm(object): install the spatialreg package
```

```
## Warning: Method logLik.sarlm moved to the spatialreg package
```

```
## Warning in logLik.sarlm(object): install the spatialreg package
```

```
## Warning: Method residuals.sarlm moved to the spatialreg package
```

```
## Warning in residuals.sarlm(object): install the spatialreg package
```

```
## Warning: Method LR1.sarlm moved to the spatialreg package
```

```
## Warning in LR1.sarlm(object): install the spatialreg package
```

```
## Warning: Method logLik.sarlm moved to the spatialreg package
```

```
## Warning in logLik.sarlm(object): install the spatialreg package
```

```
## Warning: Method residuals.sarlm moved to the spatialreg package
```

```
## Warning in residuals.sarlm(object): install the spatialreg package
```

```
## Warning: Method print.summary.sarlm moved to the spatialreg package
```

```
## Warning in print.summary.sarlm(x): install the spatialreg package
```

```
## 
## Call:errorsarlm(formula = divorce ~ med_inc + perAA + perFem + perBA + 
##     perUrb + perHisp + voteO + vryrel, data = map.join, listw = weightQ1, 
##     method = "eigen", quiet = F, zero.policy = T, tol.solve = 1e-15)
## 
## Residuals:
```

```
## Warning: Method residuals.sarlm moved to the spatialreg package
```

```
## Warning in residuals.sarlm(x): install the spatialreg package
```

```
##         Min          1Q      Median          3Q         Max 
## -0.00218602 -0.00104410 -0.00010426  0.00099710  0.00247779 
## 
## Type: error 
## Regions with no neighbours included:
##  0 49 
## Coefficients: (asymptotic standard errors) 
##                Estimate  Std. Error z value Pr(>|z|)
## (Intercept)  6.3535e-02  3.1665e-02  2.0064 0.044809
## med_inc      2.2482e-08  6.4160e-08  0.3504 0.726039
## perAA        5.1374e-05  3.9972e-05  1.2852 0.198707
## perFem      -8.5604e-04  6.1670e-04 -1.3881 0.165107
## perBA       -2.3358e-04  8.7771e-05 -2.6612 0.007786
## perUrb      -3.7649e-06  2.6533e-05 -0.1419 0.887161
## perHisp     -3.9572e-05  3.0979e-05 -1.2774 0.201469
## voteO       -1.6465e-06  2.7970e-05 -0.0589 0.953060
## vryrel      -1.7164e-05  2.9205e-05 -0.5877 0.556734
## 
## Lambda: 0.70958, LR test value: 14.376, p-value: 0.00014969
## Asymptotic standard error: 0.10323
##     z-value: 6.8736, p-value: 6.2585e-12
## Wald statistic: 47.247, p-value: 6.2584e-12
```

```
## Warning: Method logLik.sarlm moved to the spatialreg package
```

```
## Warning in logLik.sarlm(x): install the spatialreg package
```

```
## Warning: Method residuals.sarlm moved to the spatialreg package
```

```
## Warning in residuals.sarlm(object): install the spatialreg package
```

```
## 
## Log likelihood: 256.1394 for error model
## ML residual variance (sigma squared): 1.7816e-06, (sigma: 0.0013348)
## Number of observations: 50 
## Number of parameters estimated: 11
```

```
## Warning: Method logLik.sarlm moved to the spatialreg package
```

```
## Warning in logLik.sarlm(object): install the spatialreg package
```

```
## Warning: Method residuals.sarlm moved to the spatialreg package
```

```
## Warning in residuals.sarlm(object): install the spatialreg package
```

```
## AIC: -490.28, (AIC for lm: -477.9)
```

```
moran.test(err.div$residuals,weightQ1,alternative="two.sided",zero.policy=T)
```

```
## 
##  Moran I test under randomisation
## 
## data:  err.div$residuals  
## weights: weightQ1  n reduced by no-neighbour observations
##   
## 
## Moran I statistic standard deviate = 0.64121, p-value = 0.5214
## alternative hypothesis: two.sided
## sample estimates:
## Moran I statistic       Expectation          Variance 
##       0.041678572      -0.021276596       0.009639627
```

```
#residuals from spatial regression model no longer show autocorrelation
#use residuals from spatial error model

map.join$ZSdiv<-scale(residuals(err.div))
```

```
## Warning: Method residuals.sarlm moved to the spatialreg package
```

```
## Warning in residuals.sarlm(err.div): install the spatialreg package
```

### 4.4.7 General Description

For all variables, fitting a spatial regression model reduced spatial autocorrelation to non-significance. Therefore, we save these residuals and use for all subsequent analyses.

# 5 Descriptives of full dataset

Now that the final analytic variables are included, we can re-run the descriptives for completeness.

```
res.desc<-describe(map.join@data)
```

```
## Warning in describe(map.join@data): NAs introduced by coercion

## Warning in describe(map.join@data): NAs introduced by coercion

## Warning in describe(map.join@data): NAs introduced by coercion
```

```
## Warning in FUN(newX[, i], ...): no non-missing arguments to min; returning Inf

## Warning in FUN(newX[, i], ...): no non-missing arguments to min; returning Inf

## Warning in FUN(newX[, i], ...): no non-missing arguments to min; returning Inf
```

```
## Warning in FUN(newX[, i], ...): no non-missing arguments to max; returning -Inf

## Warning in FUN(newX[, i], ...): no non-missing arguments to max; returning -Inf

## Warning in FUN(newX[, i], ...): no non-missing arguments to max; returning -Inf
```

```
kable(res.desc, digits = 2)
```

|  | vars | n | mean | sd | median | trimmed | mad | min | max | range | skew | kurtosis | se |
| --- | --- | --- | --- | --- | --- | --- | --- | --- | --- | --- | --- | --- | --- |
| STATE\_ABBR\* | 1 | 50 | NaN | NA | NA | NaN | NA | Inf | -Inf | -Inf | NA | NA | NA |
| ObjectID | 2 | 50 | 24.96 | 15.01 | 24.50 | 24.95 | 19.27 | 0.00 | 50.00 | 50.00 | 0.01 | -1.30 | 2.12 |
| STATE\_NAME\* | 3 | 50 | NaN | NA | NA | NaN | NA | Inf | -Inf | -Inf | NA | NA | NA |
| STATE\_FIPS\* | 4 | 50 | 29.32 | 15.78 | 29.50 | 29.48 | 18.53 | 1.00 | 56.00 | 55.00 | -0.06 | -1.15 | 2.23 |
| SUB\_REGION\* | 5 | 50 | NaN | NA | NA | NaN | NA | Inf | -Inf | -Inf | NA | NA | NA |
| LineID | 6 | 50 | 25.50 | 14.58 | 25.50 | 25.50 | 18.53 | 1.00 | 50.00 | 49.00 | 0.00 | -1.27 | 2.06 |
| rz\_ext | 7 | 50 | 0.00 | 0.04 | 0.00 | 0.00 | 0.03 | -0.13 | 0.08 | 0.21 | -0.62 | 1.65 | 0.00 |
| rz\_agr | 8 | 50 | 0.00 | 0.06 | 0.00 | 0.00 | 0.04 | -0.15 | 0.11 | 0.27 | -0.35 | 0.40 | 0.01 |
| rz\_cns | 9 | 50 | 0.00 | 0.05 | 0.00 | 0.00 | 0.05 | -0.11 | 0.09 | 0.20 | -0.10 | -0.92 | 0.01 |
| rz\_neu | 10 | 50 | 0.00 | 0.05 | -0.01 | 0.00 | 0.06 | -0.08 | 0.14 | 0.22 | 0.53 | -0.55 | 0.01 |
| rz\_opn | 11 | 50 | -0.02 | 0.06 | -0.02 | -0.02 | 0.08 | -0.17 | 0.09 | 0.26 | -0.01 | -0.91 | 0.01 |
| mzextra | 12 | 50 | 0.09 | 0.03 | 0.09 | 0.09 | 0.03 | 0.00 | 0.17 | 0.18 | -0.27 | 1.13 | 0.00 |
| mzagree | 13 | 50 | 0.47 | 0.03 | 0.47 | 0.47 | 0.02 | 0.39 | 0.52 | 0.13 | -0.69 | 1.10 | 0.00 |
| mzconsc | 14 | 50 | 0.32 | 0.03 | 0.32 | 0.32 | 0.03 | 0.26 | 0.38 | 0.12 | -0.16 | -0.77 | 0.00 |
| mzneuro | 15 | 50 | -0.29 | 0.03 | -0.29 | -0.29 | 0.03 | -0.34 | -0.22 | 0.12 | 0.30 | -0.66 | 0.00 |
| mzopen | 16 | 50 | 0.43 | 0.03 | 0.43 | 0.43 | 0.03 | 0.37 | 0.51 | 0.14 | 0.13 | -0.49 | 0.00 |
| fzextra | 17 | 50 | 0.21 | 0.02 | 0.21 | 0.21 | 0.02 | 0.14 | 0.26 | 0.13 | -0.30 | 0.58 | 0.00 |
| fzagree | 18 | 50 | 0.58 | 0.03 | 0.58 | 0.58 | 0.03 | 0.50 | 0.64 | 0.14 | -0.33 | 0.13 | 0.00 |
| fzconsc | 19 | 50 | 0.41 | 0.03 | 0.41 | 0.41 | 0.03 | 0.34 | 0.46 | 0.12 | -0.27 | -0.53 | 0.00 |
| fzneuro | 20 | 50 | 0.02 | 0.03 | 0.02 | 0.02 | 0.04 | -0.04 | 0.12 | 0.16 | 0.49 | -0.35 | 0.00 |
| fzopen | 21 | 50 | 0.36 | 0.04 | 0.35 | 0.36 | 0.04 | 0.27 | 0.42 | 0.16 | 0.03 | -0.67 | 0.01 |
| E\_LT30 | 22 | 50 | -0.01 | 0.04 | 0.00 | 0.00 | 0.04 | -0.14 | 0.09 | 0.23 | -0.49 | 0.78 | 0.01 |
| A\_LT30 | 23 | 50 | 0.01 | 0.07 | 0.01 | 0.01 | 0.04 | -0.16 | 0.15 | 0.31 | -0.23 | 0.50 | 0.01 |
| C\_LT30 | 24 | 50 | 0.02 | 0.06 | 0.02 | 0.02 | 0.06 | -0.14 | 0.13 | 0.27 | -0.30 | -0.34 | 0.01 |
| N\_LT30 | 25 | 50 | 0.00 | 0.05 | -0.01 | 0.00 | 0.06 | -0.09 | 0.12 | 0.22 | 0.48 | -0.71 | 0.01 |
| O\_LT30 | 26 | 50 | -0.02 | 0.06 | -0.02 | -0.02 | 0.06 | -0.17 | 0.11 | 0.28 | -0.13 | -0.65 | 0.01 |
| E\_GT30 | 27 | 50 | 0.01 | 0.03 | 0.01 | 0.01 | 0.03 | -0.09 | 0.07 | 0.16 | -0.64 | 0.95 | 0.00 |
| A\_GT30 | 28 | 50 | -0.03 | 0.04 | -0.03 | -0.03 | 0.03 | -0.14 | 0.06 | 0.20 | -0.35 | 0.41 | 0.01 |
| C\_GT30 | 29 | 50 | -0.05 | 0.04 | -0.05 | -0.05 | 0.04 | -0.14 | 0.02 | 0.16 | -0.02 | -0.77 | 0.01 |
| N\_GT30 | 30 | 50 | 0.02 | 0.06 | 0.01 | 0.02 | 0.07 | -0.12 | 0.17 | 0.29 | 0.12 | -0.20 | 0.01 |
| O\_GT30 | 31 | 50 | -0.03 | 0.08 | -0.04 | -0.03 | 0.09 | -0.18 | 0.13 | 0.31 | 0.16 | -0.86 | 0.01 |
| GenD\_E | 32 | 50 | -0.12 | 0.02 | -0.12 | -0.12 | 0.02 | -0.17 | -0.09 | 0.08 | -0.39 | 0.02 | 0.00 |
| GenD\_A | 33 | 50 | -0.11 | 0.02 | -0.11 | -0.11 | 0.02 | -0.14 | -0.07 | 0.07 | 0.05 | -0.56 | 0.00 |
| GenD\_C | 34 | 50 | -0.08 | 0.02 | -0.09 | -0.08 | 0.02 | -0.14 | -0.03 | 0.11 | 0.03 | 0.04 | 0.00 |
| GenD\_N | 35 | 50 | -0.31 | 0.02 | -0.31 | -0.31 | 0.01 | -0.35 | -0.23 | 0.12 | 0.87 | 2.86 | 0.00 |
| GenD\_O | 36 | 50 | 0.08 | 0.02 | 0.08 | 0.08 | 0.02 | 0.01 | 0.14 | 0.13 | -0.29 | 0.59 | 0.00 |
| AgeD\_E | 37 | 50 | -0.02 | 0.04 | -0.01 | -0.01 | 0.04 | -0.10 | 0.05 | 0.15 | -0.25 | -0.53 | 0.01 |
| AgeD\_A | 38 | 50 | 0.04 | 0.05 | 0.04 | 0.03 | 0.03 | -0.09 | 0.17 | 0.26 | 0.42 | 1.04 | 0.01 |
| AgeD\_C | 39 | 50 | 0.07 | 0.06 | 0.07 | 0.07 | 0.05 | -0.11 | 0.21 | 0.32 | -0.19 | 0.81 | 0.01 |
| AgeD\_N | 40 | 50 | -0.02 | 0.04 | -0.01 | -0.02 | 0.04 | -0.11 | 0.09 | 0.20 | 0.04 | 0.46 | 0.01 |
| AgeD\_O | 41 | 50 | 0.01 | 0.06 | 0.01 | 0.01 | 0.07 | -0.11 | 0.14 | 0.25 | -0.03 | -0.74 | 0.01 |
| TFR | 42 | 50 | 1.95 | 0.17 | 1.94 | 1.94 | 0.12 | 1.63 | 2.45 | 0.82 | 0.56 | 0.53 | 0.02 |
| alpha | 43 | 50 | 10.51 | 1.68 | 10.77 | 10.70 | 1.63 | 6.06 | 13.34 | 7.28 | -0.83 | -0.04 | 0.24 |
| peak | 44 | 50 | 26.83 | 2.49 | 26.80 | 26.74 | 3.08 | 22.72 | 32.16 | 9.44 | 0.19 | -1.04 | 0.35 |
| stop | 45 | 50 | 3.87 | 0.82 | 4.02 | 3.93 | 0.85 | 1.28 | 5.16 | 3.88 | -0.82 | 0.33 | 0.12 |
| ageFB | 46 | 50 | 24.84 | 1.19 | 24.65 | 24.79 | 1.19 | 22.60 | 27.70 | 5.10 | 0.39 | -0.55 | 0.17 |
| t\_ageFM | 47 | 50 | 27.31 | 1.13 | 27.33 | 27.34 | 1.11 | 24.45 | 29.75 | 5.30 | -0.26 | -0.08 | 0.16 |
| nevermar | 48 | 50 | 0.31 | 0.03 | 0.31 | 0.31 | 0.03 | 0.25 | 0.38 | 0.13 | 0.14 | -0.42 | 0.00 |
| divorce | 49 | 50 | 0.01 | 0.00 | 0.01 | 0.01 | 0.00 | 0.01 | 0.02 | 0.01 | 0.10 | -0.68 | 0.00 |
| cohabit | 50 | 50 | 6.88 | 1.10 | 6.55 | 6.84 | 1.19 | 4.60 | 9.30 | 4.70 | 0.26 | -0.60 | 0.16 |
| abortion | 51 | 50 | 15.62 | 8.43 | 15.35 | 14.83 | 6.89 | 0.90 | 40.00 | 39.10 | 0.83 | 0.46 | 1.19 |
| t\_nmf | 52 | 50 | 35.55 | 6.28 | 35.85 | 35.41 | 5.86 | 15.80 | 51.20 | 35.40 | -0.09 | 0.88 | 0.89 |
| unintprg | 53 | 50 | 51.56 | 5.50 | 52.50 | 51.67 | 5.19 | 38.00 | 65.00 | 27.00 | -0.16 | -0.36 | 0.78 |
| famplnpw | 54 | 50 | 107.46 | 43.75 | 106.00 | 104.90 | 51.89 | 31.00 | 245.00 | 214.00 | 0.61 | 0.24 | 6.19 |
| med\_inc | 55 | 50 | 49755.06 | 8060.36 | 48332.50 | 49141.70 | 8435.99 | 36851.00 | 68854.00 | 32003.00 | 0.63 | -0.50 | 1139.91 |
| perAA | 56 | 50 | 10.34 | 9.55 | 7.00 | 8.90 | 8.23 | 0.40 | 37.00 | 36.60 | 1.06 | 0.18 | 1.35 |
| perHisp | 57 | 50 | 10.61 | 9.98 | 8.20 | 8.61 | 6.08 | 1.20 | 46.30 | 45.10 | 1.83 | 3.00 | 1.41 |
| perFem | 58 | 50 | 50.66 | 0.75 | 50.75 | 50.73 | 0.67 | 47.90 | 51.70 | 3.80 | -1.14 | 1.93 | 0.11 |
| perBA | 59 | 50 | 27.16 | 4.76 | 26.35 | 27.03 | 4.52 | 17.30 | 38.30 | 21.00 | 0.26 | -0.51 | 0.67 |
| perUrb | 60 | 50 | 73.58 | 14.56 | 73.74 | 74.50 | 17.33 | 38.66 | 94.95 | 56.29 | -0.43 | -0.55 | 2.06 |
| voteO | 61 | 50 | 50.51 | 9.49 | 51.26 | 50.58 | 10.55 | 32.54 | 71.85 | 39.31 | 0.01 | -0.93 | 1.34 |
| vryrel | 62 | 50 | 39.62 | 8.69 | 39.05 | 39.45 | 9.71 | 23.80 | 56.60 | 32.80 | 0.18 | -0.84 | 1.23 |
| Zext | 63 | 50 | 0.00 | 1.00 | 0.02 | 0.01 | 0.75 | -2.11 | 2.46 | 4.57 | 0.01 | -0.09 | 0.14 |
| Zagr | 64 | 50 | 0.00 | 1.00 | -0.05 | -0.01 | 0.89 | -2.13 | 2.09 | 4.22 | 0.09 | -0.29 | 0.14 |
| Zcns | 65 | 50 | 0.00 | 1.00 | 0.20 | 0.01 | 0.97 | -1.86 | 2.08 | 3.93 | -0.14 | -0.84 | 0.14 |
| Zneu | 66 | 50 | 0.00 | 1.00 | -0.02 | -0.04 | 1.11 | -1.89 | 2.11 | 4.00 | 0.24 | -0.83 | 0.14 |
| Zopn | 67 | 50 | 0.00 | 1.00 | -0.02 | -0.01 | 0.96 | -2.43 | 2.04 | 4.46 | 0.01 | -0.42 | 0.14 |
| Zmext | 68 | 50 | 0.00 | 1.00 | -0.02 | -0.01 | 0.86 | -2.71 | 2.87 | 5.59 | 0.08 | 0.76 | 0.14 |
| Zmagr | 69 | 50 | 0.00 | 1.00 | -0.04 | -0.03 | 0.74 | -2.25 | 2.28 | 4.53 | 0.22 | -0.23 | 0.14 |
| Zmcns | 70 | 50 | 0.00 | 1.00 | -0.01 | 0.02 | 0.82 | -2.26 | 2.91 | 5.17 | 0.13 | 0.67 | 0.14 |
| Zmneu | 71 | 50 | 0.00 | 1.00 | -0.12 | -0.02 | 0.99 | -1.83 | 2.63 | 4.46 | 0.30 | -0.31 | 0.14 |
| Zmopn | 72 | 50 | 0.00 | 1.00 | 0.18 | 0.02 | 1.15 | -2.22 | 1.76 | 3.98 | -0.21 | -0.93 | 0.14 |
| Zfext | 73 | 50 | 0.00 | 1.00 | -0.02 | 0.00 | 0.93 | -1.94 | 2.19 | 4.12 | 0.01 | -0.53 | 0.14 |
| Zfagr | 74 | 50 | 0.00 | 1.00 | 0.03 | 0.01 | 0.87 | -2.19 | 2.19 | 4.38 | -0.03 | -0.17 | 0.14 |
| Zfcns | 75 | 50 | 0.00 | 1.00 | 0.02 | 0.02 | 1.14 | -2.16 | 1.86 | 4.02 | -0.16 | -0.78 | 0.14 |
| Zfneu | 76 | 50 | 0.00 | 1.00 | 0.03 | 0.00 | 1.04 | -2.30 | 1.85 | 4.15 | -0.03 | -0.64 | 0.14 |
| Zfopn | 77 | 50 | 0.00 | 1.00 | 0.00 | -0.03 | 0.74 | -2.64 | 2.23 | 4.87 | 0.15 | 0.20 | 0.14 |
| Ze\_LT30 | 78 | 50 | 0.00 | 1.00 | 0.05 | 0.00 | 0.90 | -2.37 | 2.42 | 4.79 | 0.01 | -0.20 | 0.14 |
| Za\_LT30 | 79 | 50 | 0.00 | 1.00 | -0.10 | -0.04 | 0.92 | -2.09 | 2.38 | 4.47 | 0.33 | -0.03 | 0.14 |
| Zc\_LT30 | 80 | 50 | 0.00 | 1.00 | 0.19 | 0.01 | 1.05 | -2.07 | 1.85 | 3.92 | -0.11 | -0.88 | 0.14 |
| Zn\_LT30 | 81 | 50 | 0.00 | 1.00 | -0.11 | -0.02 | 1.08 | -2.20 | 1.82 | 4.02 | 0.17 | -0.85 | 0.14 |
| Zo\_LT30 | 82 | 50 | 0.00 | 1.00 | 0.08 | 0.01 | 1.03 | -2.43 | 1.76 | 4.19 | -0.12 | -0.60 | 0.14 |
| Ze\_GT30 | 83 | 50 | 0.00 | 1.00 | -0.01 | -0.02 | 0.96 | -2.31 | 2.36 | 4.67 | 0.10 | -0.05 | 0.14 |
| Za\_GT30 | 84 | 50 | 0.00 | 1.00 | 0.12 | 0.01 | 0.81 | -2.09 | 2.84 | 4.93 | 0.04 | 0.45 | 0.14 |
| Zc\_GT30 | 85 | 50 | 0.00 | 1.00 | -0.04 | 0.00 | 1.06 | -2.24 | 2.32 | 4.56 | -0.01 | -0.39 | 0.14 |
| Zn\_GT30 | 86 | 50 | 0.00 | 1.00 | -0.04 | 0.01 | 1.06 | -2.65 | 2.10 | 4.76 | -0.15 | -0.23 | 0.14 |
| Zo\_GT30 | 87 | 50 | 0.00 | 1.00 | -0.18 | -0.03 | 0.96 | -2.00 | 2.05 | 4.05 | 0.27 | -0.68 | 0.14 |
| ZgenD\_E | 88 | 50 | 0.00 | 1.00 | 0.13 | 0.03 | 0.87 | -2.64 | 2.30 | 4.94 | -0.26 | -0.01 | 0.14 |
| ZgenD\_A | 89 | 50 | 0.00 | 1.00 | -0.10 | -0.03 | 1.19 | -2.21 | 1.99 | 4.20 | 0.13 | -0.61 | 0.14 |
| ZgenD\_C | 90 | 50 | 0.00 | 1.00 | -0.06 | -0.03 | 0.97 | -2.09 | 2.21 | 4.30 | 0.28 | -0.59 | 0.14 |
| ZgenD\_N | 91 | 50 | 0.00 | 1.00 | -0.07 | -0.03 | 0.78 | -2.32 | 3.50 | 5.81 | 0.68 | 1.51 | 0.14 |
| ZgenD\_O | 92 | 50 | 0.00 | 1.00 | -0.03 | 0.05 | 1.07 | -2.50 | 1.68 | 4.18 | -0.34 | -0.45 | 0.14 |
| ZageD\_E | 93 | 50 | 0.00 | 1.00 | 0.04 | 0.04 | 0.88 | -2.98 | 1.69 | 4.67 | -0.41 | 0.18 | 0.14 |
| ZageD\_A | 94 | 50 | 0.00 | 1.00 | -0.28 | -0.14 | 0.65 | -1.40 | 2.80 | 4.20 | 1.17 | 0.86 | 0.14 |
| ZageD\_C | 95 | 50 | 0.00 | 1.00 | 0.09 | -0.02 | 1.02 | -1.81 | 2.82 | 4.63 | 0.23 | -0.24 | 0.14 |
| ZageD\_N | 96 | 50 | 0.00 | 1.00 | -0.19 | -0.04 | 0.85 | -2.62 | 2.45 | 5.07 | 0.25 | 0.39 | 0.14 |
| ZageD\_O | 97 | 50 | 0.00 | 1.00 | -0.13 | -0.08 | 1.23 | -1.87 | 2.73 | 4.60 | 0.58 | 0.06 | 0.14 |
| Ztfr | 98 | 50 | 0.00 | 1.00 | -0.19 | -0.06 | 0.91 | -2.55 | 2.65 | 5.20 | 0.41 | 0.30 | 0.14 |
| Zalpha | 99 | 50 | 0.00 | 1.00 | 0.10 | 0.05 | 0.73 | -2.94 | 2.51 | 5.45 | -0.52 | 1.06 | 0.14 |
| Zpeak | 100 | 50 | 0.00 | 1.00 | -0.21 | -0.03 | 0.83 | -3.04 | 2.32 | 5.36 | 0.04 | 0.47 | 0.14 |
| Zstop | 101 | 50 | 0.00 | 1.00 | 0.12 | 0.04 | 0.86 | -3.64 | 1.82 | 5.46 | -0.83 | 1.95 | 0.14 |
| Zafb | 102 | 50 | 0.00 | 1.00 | 0.07 | 0.01 | 0.84 | -2.48 | 2.57 | 5.05 | -0.13 | 0.38 | 0.14 |
| Zafm | 103 | 50 | 0.00 | 1.00 | 0.08 | 0.06 | 0.97 | -3.11 | 1.75 | 4.86 | -0.75 | 1.08 | 0.14 |
| Znvm | 104 | 50 | 0.00 | 1.00 | -0.17 | -0.04 | 1.06 | -2.25 | 2.22 | 4.47 | 0.28 | -0.51 | 0.14 |
| Zdiv | 105 | 50 | 0.00 | 1.00 | 0.09 | 0.01 | 1.28 | -2.01 | 1.98 | 3.99 | -0.09 | -1.17 | 0.14 |
| Zcoh | 106 | 50 | 0.00 | 1.00 | -0.13 | -0.05 | 0.86 | -2.11 | 2.91 | 5.02 | 0.47 | 0.18 | 0.14 |
| Znmf | 107 | 50 | 0.00 | 1.00 | 0.07 | 0.04 | 0.93 | -3.16 | 2.19 | 5.36 | -0.67 | 1.36 | 0.14 |
| Zuni | 108 | 50 | 0.00 | 1.00 | 0.03 | 0.00 | 0.89 | -2.69 | 2.62 | 5.31 | 0.00 | 0.54 | 0.14 |
| Zabr | 109 | 50 | 0.00 | 1.00 | -0.04 | -0.07 | 0.94 | -1.62 | 2.98 | 4.60 | 0.79 | 0.72 | 0.14 |
| Zfmp | 110 | 50 | 0.00 | 1.00 | -0.24 | -0.10 | 1.04 | -1.48 | 3.08 | 4.56 | 0.84 | 0.36 | 0.14 |
| ZSpeak | 111 | 50 | 0.00 | 1.00 | -0.06 | -0.01 | 1.05 | -1.91 | 2.56 | 4.47 | 0.26 | -0.30 | 0.14 |
| ZSneu | 112 | 50 | 0.00 | 1.00 | 0.00 | -0.06 | 1.20 | -1.69 | 2.71 | 4.40 | 0.47 | -0.27 | 0.14 |
| ZSfneu | 113 | 50 | 0.00 | 1.00 | -0.18 | -0.02 | 1.06 | -1.76 | 2.47 | 4.23 | 0.33 | -0.53 | 0.14 |
| ZSafb | 114 | 50 | 0.00 | 1.00 | 0.12 | 0.05 | 0.88 | -2.80 | 2.27 | 5.07 | -0.50 | 0.44 | 0.14 |
| ZSnvm | 115 | 50 | 0.00 | 1.00 | -0.20 | -0.05 | 0.71 | -2.27 | 2.84 | 5.11 | 0.51 | 0.43 | 0.14 |
| ZSdiv | 116 | 50 | 0.00 | 1.00 | -0.12 | -0.03 | 1.19 | -1.66 | 1.80 | 3.46 | 0.18 | -1.02 | 0.14 |

```
kable(cor(map.join@data[,7:116]), digits = 2)
```

|  | rz\_ext | rz\_agr | rz\_cns | rz\_neu | rz\_opn | mzextra | mzagree | mzconsc | mzneuro | mzopen | fzextra | fzagree | fzconsc | fzneuro | fzopen | E\_LT30 | A\_LT30 | C\_LT30 | N\_LT30 | O\_LT30 | E\_GT30 | A\_GT30 | C\_GT30 | N\_GT30 | O\_GT30 | GenD\_E | GenD\_A | GenD\_C | GenD\_N | GenD\_O | AgeD\_E | AgeD\_A | AgeD\_C | AgeD\_N | AgeD\_O | TFR | alpha | peak | stop | ageFB | t\_ageFM | nevermar | divorce | cohabit | abortion | t\_nmf | unintprg | famplnpw | med\_inc | perAA | perHisp | perFem | perBA | perUrb | voteO | vryrel | Zext | Zagr | Zcns | Zneu | Zopn | Zmext | Zmagr | Zmcns | Zmneu | Zmopn | Zfext | Zfagr | Zfcns | Zfneu | Zfopn | Ze\_LT30 | Za\_LT30 | Zc\_LT30 | Zn\_LT30 | Zo\_LT30 | Ze\_GT30 | Za\_GT30 | Zc\_GT30 | Zn\_GT30 | Zo\_GT30 | ZgenD\_E | ZgenD\_A | ZgenD\_C | ZgenD\_N | ZgenD\_O | ZageD\_E | ZageD\_A | ZageD\_C | ZageD\_N | ZageD\_O | Ztfr | Zalpha | Zpeak | Zstop | Zafb | Zafm | Znvm | Zdiv | Zcoh | Znmf | Zuni | Zabr | Zfmp | ZSpeak | ZSneu | ZSfneu | ZSafb | ZSnvm | ZSdiv |
| --- | --- | --- | --- | --- | --- | --- | --- | --- | --- | --- | --- | --- | --- | --- | --- | --- | --- | --- | --- | --- | --- | --- | --- | --- | --- | --- | --- | --- | --- | --- | --- | --- | --- | --- | --- | --- | --- | --- | --- | --- | --- | --- | --- | --- | --- | --- | --- | --- | --- | --- | --- | --- | --- | --- | --- | --- | --- | --- | --- | --- | --- | --- | --- | --- | --- | --- | --- | --- | --- | --- | --- | --- | --- | --- | --- | --- | --- | --- | --- | --- | --- | --- | --- | --- | --- | --- | --- | --- | --- | --- | --- | --- | --- | --- | --- | --- | --- | --- | --- | --- | --- | --- | --- | --- | --- | --- | --- | --- | --- | --- |
| rz\_ext | 1.00 | 0.62 | 0.77 | -0.10 | -0.58 | 0.93 | 0.61 | 0.43 | -0.26 | -0.51 | 0.95 | 0.54 | 0.59 | 0.03 | -0.60 | 0.98 | 0.62 | 0.74 | -0.12 | -0.60 | 0.68 | 0.43 | 0.45 | 0.00 | -0.42 | 0.31 | -0.07 | -0.14 | -0.40 | 0.23 | 0.52 | 0.47 | 0.43 | -0.16 | -0.05 | -0.06 | -0.08 | 0.12 | 0.46 | -0.11 | -0.08 | -0.13 | -0.14 | -0.37 | -0.22 | 0.12 | -0.20 | -0.21 | -0.34 | 0.10 | -0.20 | 0.32 | -0.18 | -0.15 | -0.17 | -0.04 | 0.84 | 0.50 | 0.64 | -0.45 | -0.56 | 0.76 | 0.46 | 0.35 | -0.54 | -0.54 | 0.80 | 0.44 | 0.47 | -0.29 | -0.54 | 0.82 | 0.47 | 0.59 | -0.44 | -0.57 | 0.66 | 0.41 | 0.51 | -0.32 | -0.43 | 0.16 | -0.07 | -0.14 | -0.25 | 0.13 | 0.34 | 0.28 | 0.19 | -0.05 | -0.15 | 0.20 | -0.14 | 0.31 | 0.30 | -0.05 | -0.14 | 0.00 | -0.22 | -0.22 | -0.08 | -0.31 | -0.17 | -0.22 | 0.22 | -0.44 | -0.31 | 0.02 | -0.02 | -0.06 |
| rz\_agr | 0.62 | 1.00 | 0.78 | -0.36 | -0.63 | 0.54 | 0.93 | 0.46 | -0.31 | -0.53 | 0.60 | 0.97 | 0.71 | -0.31 | -0.64 | 0.61 | 0.99 | 0.79 | -0.35 | -0.65 | 0.43 | 0.75 | 0.31 | -0.31 | -0.47 | 0.11 | -0.37 | -0.25 | 0.08 | 0.29 | 0.31 | 0.68 | 0.58 | -0.02 | -0.03 | 0.16 | 0.11 | -0.03 | 0.28 | -0.19 | -0.12 | -0.09 | -0.15 | -0.39 | -0.32 | 0.10 | -0.05 | 0.02 | -0.31 | 0.12 | -0.35 | 0.18 | -0.15 | -0.25 | -0.04 | -0.02 | 0.51 | 0.87 | 0.64 | -0.66 | -0.60 | 0.43 | 0.81 | 0.39 | -0.44 | -0.48 | 0.50 | 0.85 | 0.60 | -0.63 | -0.60 | 0.47 | 0.86 | 0.66 | -0.62 | -0.61 | 0.49 | 0.64 | 0.32 | -0.56 | -0.45 | 0.03 | -0.29 | -0.25 | 0.26 | 0.29 | 0.07 | 0.59 | 0.43 | 0.06 | -0.17 | 0.44 | 0.06 | 0.06 | 0.07 | -0.23 | -0.13 | 0.09 | -0.24 | -0.37 | -0.07 | -0.10 | -0.23 | 0.06 | 0.20 | -0.64 | -0.39 | -0.08 | 0.14 | -0.18 |
| rz\_cns | 0.77 | 0.78 | 1.00 | -0.43 | -0.58 | 0.72 | 0.71 | 0.72 | -0.54 | -0.45 | 0.71 | 0.75 | 0.88 | -0.31 | -0.61 | 0.78 | 0.75 | 0.98 | -0.46 | -0.57 | 0.41 | 0.67 | 0.55 | -0.22 | -0.49 | 0.26 | -0.31 | -0.12 | -0.22 | 0.33 | 0.52 | 0.43 | 0.60 | -0.30 | 0.08 | 0.19 | 0.06 | -0.04 | 0.40 | -0.31 | -0.27 | -0.24 | -0.02 | -0.42 | -0.39 | 0.05 | -0.23 | -0.19 | -0.43 | 0.12 | -0.25 | 0.15 | -0.22 | -0.19 | -0.24 | 0.08 | 0.62 | 0.61 | 0.81 | -0.61 | -0.55 | 0.56 | 0.54 | 0.57 | -0.62 | -0.47 | 0.60 | 0.58 | 0.71 | -0.49 | -0.54 | 0.62 | 0.57 | 0.80 | -0.64 | -0.56 | 0.44 | 0.57 | 0.54 | -0.39 | -0.42 | 0.11 | -0.23 | -0.18 | -0.10 | 0.22 | 0.32 | 0.26 | 0.38 | -0.16 | -0.16 | 0.35 | -0.04 | 0.22 | 0.29 | -0.22 | -0.21 | -0.09 | -0.15 | -0.35 | -0.16 | -0.36 | -0.27 | -0.15 | 0.21 | -0.59 | -0.48 | -0.18 | -0.04 | -0.03 |
| rz\_neu | -0.10 | -0.36 | -0.43 | 1.00 | 0.08 | -0.07 | -0.29 | -0.33 | 0.89 | 0.00 | -0.10 | -0.39 | -0.42 | 0.98 | 0.11 | -0.05 | -0.33 | -0.34 | 0.98 | 0.07 | -0.21 | -0.40 | -0.52 | 0.85 | 0.09 | 0.02 | 0.27 | 0.08 | -0.37 | -0.18 | 0.12 | -0.10 | 0.03 | 0.10 | -0.05 | -0.60 | -0.08 | 0.12 | 0.31 | 0.20 | 0.36 | 0.05 | -0.02 | -0.11 | 0.13 | 0.30 | 0.14 | 0.06 | -0.12 | 0.09 | -0.22 | 0.49 | -0.13 | -0.26 | 0.10 | -0.06 | -0.35 | -0.50 | -0.50 | 0.66 | 0.36 | -0.29 | -0.46 | -0.33 | 0.55 | 0.26 | -0.32 | -0.50 | -0.53 | 0.63 | 0.36 | -0.31 | -0.47 | -0.47 | 0.64 | 0.39 | -0.38 | -0.46 | -0.43 | 0.54 | 0.24 | -0.03 | 0.20 | 0.24 | -0.14 | -0.20 | 0.02 | -0.23 | -0.12 | -0.02 | 0.17 | -0.32 | -0.15 | 0.08 | 0.02 | 0.26 | 0.22 | 0.12 | 0.06 | 0.16 | 0.04 | 0.09 | 0.19 | -0.05 | 0.02 | 0.61 | 0.59 | 0.31 | -0.04 | 0.01 |
| rz\_opn | -0.58 | -0.63 | -0.58 | 0.08 | 1.00 | -0.53 | -0.49 | -0.33 | 0.21 | 0.87 | -0.57 | -0.65 | -0.46 | -0.01 | 0.98 | -0.62 | -0.64 | -0.60 | 0.12 | 0.96 | -0.20 | -0.41 | -0.18 | -0.05 | 0.86 | -0.14 | 0.45 | 0.12 | 0.29 | -0.35 | -0.53 | -0.50 | -0.48 | 0.22 | -0.18 | -0.27 | -0.39 | 0.29 | -0.52 | 0.48 | 0.45 | 0.44 | -0.11 | 0.51 | 0.52 | -0.18 | 0.11 | 0.09 | 0.44 | -0.09 | 0.54 | 0.02 | 0.54 | 0.54 | 0.25 | -0.01 | -0.46 | -0.48 | -0.46 | 0.38 | 0.69 | -0.41 | -0.40 | -0.18 | 0.31 | 0.57 | -0.45 | -0.47 | -0.42 | 0.33 | 0.67 | -0.45 | -0.48 | -0.47 | 0.36 | 0.67 | -0.38 | -0.32 | -0.24 | 0.31 | 0.58 | -0.05 | 0.24 | 0.29 | -0.04 | -0.28 | -0.16 | -0.36 | -0.30 | -0.03 | 0.08 | -0.36 | -0.03 | -0.06 | -0.15 | 0.25 | 0.19 | 0.01 | 0.21 | 0.33 | 0.05 | 0.12 | 0.13 | 0.10 | -0.07 | 0.37 | 0.26 | 0.11 | 0.09 | 0.14 |
| mzextra | 0.93 | 0.54 | 0.72 | -0.07 | -0.53 | 1.00 | 0.54 | 0.39 | -0.22 | -0.52 | 0.81 | 0.45 | 0.49 | 0.06 | -0.53 | 0.93 | 0.54 | 0.68 | -0.10 | -0.55 | 0.55 | 0.34 | 0.41 | 0.06 | -0.38 | 0.62 | 0.02 | -0.07 | -0.38 | 0.13 | 0.58 | 0.43 | 0.40 | -0.23 | -0.04 | -0.01 | -0.10 | 0.17 | 0.42 | -0.07 | -0.07 | -0.15 | -0.20 | -0.33 | -0.26 | -0.02 | -0.30 | -0.23 | -0.31 | 0.02 | -0.21 | 0.25 | -0.17 | -0.12 | -0.21 | -0.01 | 0.76 | 0.41 | 0.57 | -0.36 | -0.49 | 0.83 | 0.40 | 0.32 | -0.46 | -0.52 | 0.64 | 0.34 | 0.37 | -0.23 | -0.46 | 0.76 | 0.39 | 0.52 | -0.37 | -0.49 | 0.55 | 0.33 | 0.46 | -0.24 | -0.39 | 0.48 | 0.01 | -0.06 | -0.24 | 0.05 | 0.38 | 0.23 | 0.16 | -0.08 | -0.11 | 0.20 | -0.18 | 0.35 | 0.23 | 0.04 | -0.06 | 0.02 | -0.29 | -0.21 | -0.18 | -0.34 | -0.20 | -0.22 | 0.27 | -0.33 | -0.20 | 0.08 | 0.01 | -0.12 |
| mzagree | 0.61 | 0.93 | 0.71 | -0.29 | -0.49 | 0.54 | 1.00 | 0.50 | -0.25 | -0.42 | 0.56 | 0.85 | 0.64 | -0.24 | -0.50 | 0.58 | 0.92 | 0.70 | -0.28 | -0.50 | 0.47 | 0.71 | 0.34 | -0.26 | -0.39 | 0.18 | -0.03 | -0.11 | 0.06 | 0.21 | 0.25 | 0.63 | 0.47 | 0.00 | 0.01 | 0.12 | 0.05 | 0.02 | 0.20 | -0.13 | -0.07 | -0.05 | -0.12 | -0.39 | -0.22 | 0.06 | -0.02 | 0.01 | -0.27 | 0.14 | -0.26 | 0.28 | -0.11 | -0.13 | 0.00 | 0.02 | 0.48 | 0.82 | 0.58 | -0.61 | -0.52 | 0.42 | 0.88 | 0.45 | -0.44 | -0.42 | 0.44 | 0.75 | 0.51 | -0.57 | -0.50 | 0.44 | 0.80 | 0.59 | -0.59 | -0.50 | 0.48 | 0.63 | 0.33 | -0.50 | -0.44 | 0.09 | 0.01 | -0.08 | 0.19 | 0.21 | 0.05 | 0.53 | 0.35 | 0.03 | -0.05 | 0.45 | 0.08 | 0.04 | 0.04 | -0.23 | -0.19 | 0.00 | -0.14 | -0.36 | -0.19 | -0.12 | -0.23 | 0.04 | 0.21 | -0.60 | -0.30 | -0.06 | 0.07 | -0.09 |
| mzconsc | 0.43 | 0.46 | 0.72 | -0.33 | -0.33 | 0.39 | 0.50 | 1.00 | -0.46 | -0.10 | 0.28 | 0.49 | 0.68 | -0.25 | -0.37 | 0.43 | 0.41 | 0.69 | -0.35 | -0.18 | 0.22 | 0.59 | 0.52 | -0.20 | -0.55 | 0.29 | -0.13 | 0.48 | -0.22 | 0.44 | 0.29 | 0.06 | 0.33 | -0.19 | 0.58 | 0.36 | 0.33 | -0.47 | 0.12 | -0.63 | -0.45 | -0.29 | 0.39 | -0.53 | -0.42 | 0.24 | 0.00 | -0.12 | -0.61 | 0.27 | -0.03 | 0.05 | -0.51 | -0.16 | -0.38 | 0.35 | 0.30 | 0.32 | 0.49 | -0.35 | -0.18 | 0.27 | 0.37 | 0.71 | -0.42 | -0.10 | 0.22 | 0.30 | 0.44 | -0.27 | -0.18 | 0.30 | 0.26 | 0.47 | -0.35 | -0.15 | 0.19 | 0.49 | 0.40 | -0.26 | -0.25 | 0.14 | 0.03 | 0.29 | -0.15 | 0.14 | 0.17 | -0.07 | 0.15 | -0.03 | 0.15 | 0.29 | 0.09 | 0.01 | 0.14 | -0.25 | -0.27 | -0.18 | 0.09 | -0.30 | -0.19 | -0.31 | -0.28 | -0.13 | 0.09 | -0.33 | -0.27 | -0.25 | -0.12 | 0.12 |
| mzneuro | -0.26 | -0.31 | -0.54 | 0.89 | 0.21 | -0.22 | -0.25 | -0.46 | 1.00 | 0.14 | -0.25 | -0.34 | -0.48 | 0.78 | 0.22 | -0.24 | -0.26 | -0.45 | 0.89 | 0.17 | -0.19 | -0.42 | -0.55 | 0.69 | 0.24 | -0.04 | 0.24 | -0.03 | 0.08 | -0.15 | -0.11 | 0.00 | -0.07 | 0.21 | -0.15 | -0.56 | -0.20 | 0.20 | 0.06 | 0.34 | 0.49 | 0.22 | -0.13 | -0.02 | 0.33 | 0.23 | 0.26 | 0.15 | 0.04 | 0.07 | -0.08 | 0.48 | 0.02 | -0.03 | 0.21 | -0.08 | -0.47 | -0.37 | -0.55 | 0.60 | 0.33 | -0.40 | -0.37 | -0.43 | 0.72 | 0.30 | -0.43 | -0.35 | -0.52 | 0.46 | 0.31 | -0.45 | -0.33 | -0.51 | 0.60 | 0.35 | -0.37 | -0.43 | -0.51 | 0.45 | 0.25 | -0.06 | 0.07 | 0.11 | 0.26 | -0.08 | -0.17 | -0.07 | -0.11 | 0.05 | 0.11 | -0.27 | -0.11 | 0.00 | -0.14 | 0.30 | 0.28 | 0.16 | 0.01 | 0.17 | 0.06 | 0.26 | 0.27 | 0.07 | 0.03 | 0.59 | 0.56 | 0.40 | 0.00 | -0.04 |
| mzopen | -0.51 | -0.53 | -0.45 | 0.00 | 0.87 | -0.52 | -0.42 | -0.10 | 0.14 | 1.00 | -0.56 | -0.48 | -0.21 | -0.11 | 0.76 | -0.58 | -0.53 | -0.43 | 0.03 | 0.90 | -0.10 | -0.27 | -0.17 | -0.11 | 0.62 | -0.13 | 0.25 | 0.12 | 0.36 | 0.16 | -0.56 | -0.48 | -0.32 | 0.18 | 0.07 | -0.15 | -0.21 | 0.00 | -0.55 | 0.17 | 0.27 | 0.29 | 0.09 | 0.30 | 0.40 | -0.04 | 0.20 | 0.11 | 0.18 | 0.02 | 0.66 | 0.04 | 0.32 | 0.45 | 0.13 | 0.05 | -0.44 | -0.38 | -0.39 | 0.27 | 0.57 | -0.43 | -0.33 | -0.10 | 0.28 | 0.68 | -0.45 | -0.33 | -0.24 | 0.19 | 0.48 | -0.44 | -0.39 | -0.38 | 0.26 | 0.58 | -0.34 | -0.17 | -0.22 | 0.21 | 0.42 | -0.09 | 0.09 | 0.17 | 0.09 | 0.11 | -0.19 | -0.37 | -0.21 | 0.00 | 0.18 | -0.26 | 0.06 | -0.19 | -0.16 | 0.06 | 0.05 | -0.12 | 0.27 | 0.22 | -0.04 | 0.09 | 0.08 | 0.07 | -0.22 | 0.30 | 0.19 | -0.01 | 0.01 | 0.22 |
| fzextra | 0.95 | 0.60 | 0.71 | -0.10 | -0.57 | 0.81 | 0.56 | 0.28 | -0.25 | -0.56 | 1.00 | 0.52 | 0.49 | 0.03 | -0.57 | 0.93 | 0.61 | 0.68 | -0.12 | -0.63 | 0.65 | 0.38 | 0.40 | 0.00 | -0.35 | 0.04 | -0.10 | -0.22 | -0.38 | 0.14 | 0.49 | 0.48 | 0.40 | -0.17 | -0.17 | -0.10 | -0.15 | 0.23 | 0.51 | -0.02 | -0.02 | -0.06 | -0.20 | -0.32 | -0.14 | 0.10 | -0.18 | -0.21 | -0.20 | 0.10 | -0.24 | 0.30 | -0.08 | -0.12 | -0.09 | -0.11 | 0.84 | 0.51 | 0.64 | -0.43 | -0.58 | 0.67 | 0.45 | 0.28 | -0.52 | -0.58 | 0.88 | 0.45 | 0.44 | -0.27 | -0.55 | 0.82 | 0.49 | 0.60 | -0.44 | -0.60 | 0.67 | 0.37 | 0.48 | -0.29 | -0.40 | -0.07 | -0.12 | -0.20 | -0.26 | 0.11 | 0.32 | 0.33 | 0.23 | -0.08 | -0.23 | 0.17 | -0.17 | 0.36 | 0.39 | -0.06 | -0.12 | 0.05 | -0.23 | -0.22 | 0.00 | -0.26 | -0.13 | -0.23 | 0.25 | -0.46 | -0.36 | -0.01 | 0.00 | -0.07 |
| fzagree | 0.54 | 0.97 | 0.75 | -0.39 | -0.65 | 0.45 | 0.85 | 0.49 | -0.34 | -0.48 | 0.52 | 1.00 | 0.76 | -0.35 | -0.67 | 0.53 | 0.96 | 0.78 | -0.36 | -0.65 | 0.38 | 0.76 | 0.28 | -0.35 | -0.51 | 0.07 | -0.55 | -0.28 | 0.12 | 0.38 | 0.27 | 0.63 | 0.59 | 0.01 | 0.03 | 0.19 | 0.20 | -0.16 | 0.24 | -0.28 | -0.18 | -0.14 | -0.09 | -0.38 | -0.37 | 0.18 | 0.00 | 0.06 | -0.38 | 0.12 | -0.34 | 0.13 | -0.22 | -0.33 | -0.05 | -0.01 | 0.44 | 0.83 | 0.61 | -0.65 | -0.58 | 0.35 | 0.73 | 0.36 | -0.41 | -0.42 | 0.43 | 0.85 | 0.62 | -0.65 | -0.59 | 0.40 | 0.82 | 0.63 | -0.60 | -0.60 | 0.44 | 0.63 | 0.30 | -0.58 | -0.43 | -0.01 | -0.43 | -0.31 | 0.32 | 0.35 | 0.03 | 0.54 | 0.41 | 0.12 | -0.18 | 0.42 | 0.09 | -0.01 | 0.03 | -0.25 | -0.12 | 0.09 | -0.23 | -0.35 | 0.00 | -0.06 | -0.21 | 0.10 | 0.13 | -0.62 | -0.41 | -0.09 | 0.14 | -0.18 |
| fzconsc | 0.59 | 0.71 | 0.88 | -0.42 | -0.46 | 0.49 | 0.64 | 0.68 | -0.48 | -0.21 | 0.49 | 0.76 | 1.00 | -0.36 | -0.52 | 0.57 | 0.68 | 0.89 | -0.43 | -0.44 | 0.38 | 0.67 | 0.45 | -0.32 | -0.42 | 0.17 | -0.44 | -0.32 | -0.05 | 0.52 | 0.31 | 0.35 | 0.58 | -0.12 | 0.12 | 0.11 | 0.16 | -0.21 | 0.19 | -0.33 | -0.22 | -0.28 | 0.07 | -0.32 | -0.34 | 0.16 | -0.11 | -0.05 | -0.49 | 0.11 | -0.12 | 0.18 | -0.20 | -0.24 | -0.14 | 0.06 | 0.44 | 0.54 | 0.69 | -0.63 | -0.48 | 0.35 | 0.46 | 0.49 | -0.56 | -0.28 | 0.40 | 0.58 | 0.79 | -0.58 | -0.50 | 0.42 | 0.50 | 0.68 | -0.63 | -0.49 | 0.33 | 0.55 | 0.48 | -0.48 | -0.38 | 0.03 | -0.35 | -0.36 | 0.07 | 0.38 | 0.18 | 0.20 | 0.32 | -0.04 | -0.11 | 0.29 | 0.08 | -0.04 | 0.08 | -0.27 | -0.24 | -0.21 | -0.03 | -0.25 | -0.10 | -0.25 | -0.21 | -0.02 | -0.03 | -0.57 | -0.47 | -0.14 | -0.09 | 0.06 |
| fzneuro | 0.03 | -0.31 | -0.31 | 0.98 | -0.01 | 0.06 | -0.24 | -0.25 | 0.78 | -0.11 | 0.03 | -0.35 | -0.36 | 1.00 | 0.03 | 0.10 | -0.28 | -0.23 | 0.94 | -0.02 | -0.17 | -0.35 | -0.45 | 0.87 | 0.02 | 0.05 | 0.28 | 0.12 | -0.56 | -0.20 | 0.25 | -0.08 | 0.09 | 0.02 | -0.04 | -0.57 | -0.07 | 0.13 | 0.43 | 0.15 | 0.29 | 0.00 | -0.01 | -0.17 | 0.03 | 0.28 | 0.05 | -0.01 | -0.16 | 0.09 | -0.28 | 0.48 | -0.17 | -0.31 | 0.04 | -0.05 | -0.23 | -0.47 | -0.39 | 0.61 | 0.31 | -0.18 | -0.42 | -0.25 | 0.41 | 0.18 | -0.20 | -0.49 | -0.48 | 0.64 | 0.31 | -0.17 | -0.45 | -0.37 | 0.58 | 0.33 | -0.32 | -0.42 | -0.35 | 0.53 | 0.19 | -0.01 | 0.24 | 0.28 | -0.32 | -0.23 | 0.12 | -0.23 | -0.09 | -0.06 | 0.17 | -0.29 | -0.16 | 0.13 | 0.12 | 0.22 | 0.18 | 0.12 | 0.05 | 0.11 | 0.01 | -0.02 | 0.11 | -0.11 | 0.05 | 0.55 | 0.55 | 0.24 | -0.04 | 0.03 |
| fzopen | -0.60 | -0.64 | -0.61 | 0.11 | 0.98 | -0.53 | -0.50 | -0.37 | 0.22 | 0.76 | -0.57 | -0.67 | -0.52 | 0.03 | 1.00 | -0.63 | -0.65 | -0.63 | 0.15 | 0.93 | -0.24 | -0.43 | -0.16 | -0.03 | 0.87 | -0.14 | 0.48 | 0.13 | 0.24 | -0.52 | -0.50 | -0.49 | -0.52 | 0.24 | -0.24 | -0.29 | -0.39 | 0.34 | -0.49 | 0.54 | 0.48 | 0.45 | -0.14 | 0.55 | 0.51 | -0.18 | 0.09 | 0.10 | 0.48 | -0.11 | 0.45 | 0.00 | 0.55 | 0.51 | 0.29 | -0.02 | -0.47 | -0.50 | -0.48 | 0.39 | 0.70 | -0.40 | -0.41 | -0.19 | 0.30 | 0.51 | -0.46 | -0.50 | -0.46 | 0.35 | 0.72 | -0.45 | -0.50 | -0.50 | 0.38 | 0.68 | -0.39 | -0.36 | -0.22 | 0.31 | 0.61 | -0.04 | 0.28 | 0.32 | -0.08 | -0.42 | -0.16 | -0.35 | -0.35 | -0.01 | 0.05 | -0.39 | -0.04 | -0.04 | -0.17 | 0.30 | 0.23 | 0.04 | 0.19 | 0.37 | 0.09 | 0.14 | 0.14 | 0.12 | -0.04 | 0.37 | 0.28 | 0.16 | 0.10 | 0.12 |
| E\_LT30 | 0.98 | 0.61 | 0.78 | -0.05 | -0.62 | 0.93 | 0.58 | 0.43 | -0.24 | -0.58 | 0.93 | 0.53 | 0.57 | 0.10 | -0.63 | 1.00 | 0.60 | 0.76 | -0.09 | -0.64 | 0.51 | 0.41 | 0.39 | 0.11 | -0.47 | 0.35 | -0.08 | -0.12 | -0.47 | 0.19 | 0.69 | 0.46 | 0.49 | -0.28 | -0.03 | -0.07 | -0.07 | 0.14 | 0.56 | -0.13 | -0.13 | -0.20 | -0.10 | -0.38 | -0.30 | 0.07 | -0.29 | -0.25 | -0.36 | 0.07 | -0.31 | 0.30 | -0.22 | -0.20 | -0.19 | -0.06 | 0.78 | 0.43 | 0.61 | -0.37 | -0.52 | 0.72 | 0.40 | 0.33 | -0.49 | -0.51 | 0.74 | 0.38 | 0.43 | -0.21 | -0.50 | 0.79 | 0.41 | 0.57 | -0.39 | -0.53 | 0.52 | 0.37 | 0.44 | -0.22 | -0.41 | 0.18 | -0.06 | -0.12 | -0.30 | 0.11 | 0.45 | 0.22 | 0.23 | -0.12 | -0.12 | 0.17 | -0.16 | 0.33 | 0.34 | -0.05 | -0.15 | -0.01 | -0.19 | -0.23 | -0.12 | -0.37 | -0.19 | -0.24 | 0.24 | -0.37 | -0.26 | 0.01 | -0.05 | -0.03 |
| A\_LT30 | 0.62 | 0.99 | 0.75 | -0.33 | -0.64 | 0.54 | 0.92 | 0.41 | -0.26 | -0.53 | 0.61 | 0.96 | 0.68 | -0.28 | -0.65 | 0.60 | 1.00 | 0.77 | -0.32 | -0.67 | 0.44 | 0.65 | 0.25 | -0.26 | -0.46 | 0.11 | -0.35 | -0.28 | 0.10 | 0.28 | 0.31 | 0.78 | 0.60 | -0.06 | -0.06 | 0.14 | 0.09 | -0.02 | 0.27 | -0.18 | -0.10 | -0.08 | -0.17 | -0.38 | -0.30 | 0.12 | -0.04 | 0.03 | -0.33 | 0.09 | -0.33 | 0.20 | -0.17 | -0.24 | -0.03 | -0.05 | 0.49 | 0.85 | 0.60 | -0.62 | -0.60 | 0.40 | 0.79 | 0.31 | -0.39 | -0.49 | 0.49 | 0.83 | 0.55 | -0.60 | -0.60 | 0.44 | 0.86 | 0.62 | -0.59 | -0.62 | 0.49 | 0.55 | 0.26 | -0.52 | -0.44 | 0.01 | -0.28 | -0.29 | 0.28 | 0.27 | 0.03 | 0.67 | 0.44 | 0.05 | -0.21 | 0.42 | 0.04 | 0.06 | 0.05 | -0.21 | -0.09 | 0.12 | -0.26 | -0.36 | -0.04 | -0.06 | -0.20 | 0.08 | 0.19 | -0.61 | -0.35 | -0.05 | 0.15 | -0.20 |
| C\_LT30 | 0.74 | 0.79 | 0.98 | -0.34 | -0.60 | 0.68 | 0.70 | 0.69 | -0.45 | -0.43 | 0.68 | 0.78 | 0.89 | -0.23 | -0.63 | 0.76 | 0.77 | 1.00 | -0.38 | -0.59 | 0.35 | 0.63 | 0.36 | -0.12 | -0.50 | 0.25 | -0.35 | -0.17 | -0.24 | 0.39 | 0.55 | 0.49 | 0.75 | -0.33 | 0.07 | 0.14 | 0.09 | -0.05 | 0.47 | -0.32 | -0.27 | -0.28 | 0.00 | -0.42 | -0.44 | 0.06 | -0.26 | -0.17 | -0.50 | 0.09 | -0.27 | 0.18 | -0.24 | -0.27 | -0.23 | 0.01 | 0.54 | 0.58 | 0.75 | -0.54 | -0.52 | 0.48 | 0.51 | 0.51 | -0.54 | -0.42 | 0.52 | 0.56 | 0.66 | -0.44 | -0.53 | 0.55 | 0.55 | 0.76 | -0.57 | -0.54 | 0.35 | 0.51 | 0.38 | -0.32 | -0.40 | 0.08 | -0.24 | -0.19 | -0.08 | 0.25 | 0.32 | 0.29 | 0.49 | -0.19 | -0.15 | 0.33 | -0.04 | 0.21 | 0.31 | -0.24 | -0.22 | -0.07 | -0.13 | -0.35 | -0.19 | -0.37 | -0.28 | -0.13 | 0.20 | -0.52 | -0.41 | -0.17 | -0.01 | -0.03 |
| N\_LT30 | -0.12 | -0.35 | -0.46 | 0.98 | 0.12 | -0.10 | -0.28 | -0.35 | 0.89 | 0.03 | -0.12 | -0.36 | -0.43 | 0.94 | 0.15 | -0.09 | -0.32 | -0.38 | 1.00 | 0.10 | -0.14 | -0.34 | -0.49 | 0.73 | 0.14 | -0.01 | 0.25 | 0.05 | -0.32 | -0.20 | 0.01 | -0.14 | -0.04 | 0.29 | -0.10 | -0.62 | -0.10 | 0.13 | 0.22 | 0.25 | 0.42 | 0.14 | -0.08 | -0.09 | 0.20 | 0.33 | 0.22 | 0.13 | -0.06 | 0.12 | -0.19 | 0.52 | -0.07 | -0.22 | 0.15 | -0.04 | -0.34 | -0.46 | -0.50 | 0.62 | 0.34 | -0.29 | -0.43 | -0.32 | 0.53 | 0.24 | -0.32 | -0.45 | -0.51 | 0.58 | 0.34 | -0.31 | -0.44 | -0.48 | 0.64 | 0.36 | -0.32 | -0.41 | -0.40 | 0.41 | 0.23 | -0.03 | 0.15 | 0.23 | -0.10 | -0.19 | -0.06 | -0.23 | -0.17 | 0.15 | 0.14 | -0.31 | -0.11 | 0.02 | -0.04 | 0.27 | 0.23 | 0.16 | 0.04 | 0.17 | 0.09 | 0.16 | 0.21 | 0.01 | -0.01 | 0.57 | 0.56 | 0.33 | 0.00 | -0.01 |
| O\_LT30 | -0.60 | -0.65 | -0.57 | 0.07 | 0.96 | -0.55 | -0.50 | -0.18 | 0.17 | 0.90 | -0.63 | -0.65 | -0.44 | -0.02 | 0.93 | -0.64 | -0.67 | -0.59 | 0.10 | 1.00 | -0.24 | -0.39 | -0.14 | -0.03 | 0.69 | -0.10 | 0.45 | 0.29 | 0.25 | -0.23 | -0.52 | -0.56 | -0.50 | 0.17 | 0.09 | -0.16 | -0.29 | 0.10 | -0.55 | 0.28 | 0.32 | 0.36 | 0.05 | 0.38 | 0.44 | -0.10 | 0.18 | 0.06 | 0.29 | 0.01 | 0.56 | -0.01 | 0.36 | 0.52 | 0.14 | 0.11 | -0.50 | -0.52 | -0.51 | 0.43 | 0.71 | -0.43 | -0.42 | -0.15 | 0.35 | 0.62 | -0.50 | -0.51 | -0.45 | 0.38 | 0.69 | -0.48 | -0.53 | -0.51 | 0.41 | 0.73 | -0.42 | -0.31 | -0.25 | 0.36 | 0.51 | -0.03 | 0.29 | 0.36 | -0.06 | -0.25 | -0.16 | -0.43 | -0.33 | -0.03 | 0.24 | -0.35 | -0.02 | -0.10 | -0.16 | 0.20 | 0.14 | -0.06 | 0.25 | 0.29 | 0.00 | 0.11 | 0.10 | 0.05 | -0.09 | 0.42 | 0.31 | 0.05 | 0.02 | 0.17 |
| E\_GT30 | 0.68 | 0.43 | 0.41 | -0.21 | -0.20 | 0.55 | 0.47 | 0.22 | -0.19 | -0.10 | 0.65 | 0.38 | 0.38 | -0.17 | -0.24 | 0.51 | 0.44 | 0.35 | -0.14 | -0.24 | 1.00 | 0.29 | 0.46 | -0.34 | -0.09 | 0.06 | 0.03 | -0.17 | 0.02 | 0.23 | -0.27 | 0.33 | 0.03 | 0.29 | -0.12 | -0.01 | -0.14 | 0.07 | -0.05 | 0.04 | 0.16 | 0.18 | -0.27 | -0.21 | 0.16 | 0.22 | 0.19 | 0.01 | -0.07 | 0.17 | 0.21 | 0.33 | 0.07 | 0.10 | 0.00 | 0.05 | 0.70 | 0.51 | 0.48 | -0.51 | -0.50 | 0.59 | 0.49 | 0.24 | -0.46 | -0.44 | 0.68 | 0.47 | 0.38 | -0.44 | -0.48 | 0.58 | 0.51 | 0.41 | -0.44 | -0.52 | 0.90 | 0.35 | 0.52 | -0.53 | -0.35 | 0.04 | -0.09 | -0.17 | 0.02 | 0.16 | -0.23 | 0.37 | -0.02 | 0.22 | -0.18 | 0.25 | -0.03 | 0.07 | 0.03 | -0.04 | -0.07 | 0.00 | -0.26 | -0.14 | 0.07 | 0.04 | -0.02 | -0.07 | 0.05 | -0.50 | -0.35 | 0.05 | 0.07 | -0.14 |
| A\_GT30 | 0.43 | 0.75 | 0.67 | -0.40 | -0.41 | 0.34 | 0.71 | 0.59 | -0.42 | -0.27 | 0.38 | 0.76 | 0.67 | -0.35 | -0.43 | 0.41 | 0.65 | 0.63 | -0.34 | -0.39 | 0.29 | 1.00 | 0.48 | -0.44 | -0.39 | 0.06 | -0.32 | -0.04 | 0.00 | 0.30 | 0.21 | 0.03 | 0.30 | 0.17 | 0.14 | 0.21 | 0.22 | -0.10 | 0.21 | -0.20 | -0.23 | -0.13 | -0.01 | -0.35 | -0.32 | 0.02 | -0.04 | 0.01 | -0.19 | 0.21 | -0.32 | 0.12 | -0.07 | -0.26 | -0.03 | 0.12 | 0.42 | 0.64 | 0.61 | -0.61 | -0.40 | 0.35 | 0.62 | 0.61 | -0.52 | -0.21 | 0.37 | 0.65 | 0.61 | -0.57 | -0.43 | 0.41 | 0.55 | 0.59 | -0.56 | -0.37 | 0.34 | 0.87 | 0.49 | -0.58 | -0.42 | 0.07 | -0.21 | -0.01 | 0.11 | 0.36 | 0.15 | 0.00 | 0.21 | 0.15 | 0.09 | 0.43 | 0.20 | -0.02 | 0.13 | -0.33 | -0.34 | -0.13 | -0.04 | -0.35 | -0.25 | -0.22 | -0.30 | 0.01 | 0.15 | -0.59 | -0.44 | -0.22 | -0.01 | -0.02 |
| C\_GT30 | 0.45 | 0.31 | 0.55 | -0.52 | -0.18 | 0.41 | 0.34 | 0.52 | -0.55 | -0.17 | 0.40 | 0.28 | 0.45 | -0.45 | -0.16 | 0.39 | 0.25 | 0.36 | -0.49 | -0.14 | 0.46 | 0.48 | 1.00 | -0.49 | -0.22 | 0.16 | 0.01 | 0.13 | -0.01 | 0.03 | 0.05 | -0.07 | -0.34 | 0.04 | 0.15 | 0.29 | 0.00 | -0.07 | -0.15 | -0.14 | -0.15 | -0.04 | 0.01 | -0.25 | 0.00 | 0.05 | 0.05 | -0.11 | -0.02 | 0.20 | 0.01 | 0.00 | -0.08 | 0.22 | -0.13 | 0.30 | 0.54 | 0.33 | 0.59 | -0.58 | -0.31 | 0.49 | 0.33 | 0.51 | -0.62 | -0.29 | 0.49 | 0.32 | 0.54 | -0.49 | -0.27 | 0.50 | 0.27 | 0.45 | -0.56 | -0.31 | 0.52 | 0.50 | 0.89 | -0.49 | -0.25 | 0.14 | -0.06 | -0.05 | -0.11 | 0.04 | 0.07 | -0.06 | -0.31 | 0.05 | -0.06 | 0.23 | 0.03 | 0.01 | -0.01 | -0.11 | -0.16 | -0.25 | -0.08 | -0.21 | -0.01 | -0.11 | -0.13 | -0.11 | 0.01 | -0.55 | -0.48 | -0.13 | -0.25 | 0.06 |
| N\_GT30 | 0.00 | -0.31 | -0.22 | 0.85 | -0.05 | 0.06 | -0.26 | -0.20 | 0.69 | -0.11 | 0.00 | -0.35 | -0.32 | 0.87 | -0.03 | 0.11 | -0.26 | -0.12 | 0.73 | -0.03 | -0.34 | -0.44 | -0.49 | 1.00 | -0.06 | 0.11 | 0.25 | 0.12 | -0.47 | -0.09 | 0.40 | 0.02 | 0.22 | -0.44 | 0.05 | -0.43 | -0.02 | 0.08 | 0.50 | 0.01 | 0.11 | -0.19 | 0.11 | -0.16 | -0.10 | 0.14 | -0.12 | -0.12 | -0.25 | -0.01 | -0.26 | 0.32 | -0.27 | -0.31 | -0.08 | -0.08 | -0.29 | -0.47 | -0.35 | 0.60 | 0.34 | -0.21 | -0.43 | -0.27 | 0.46 | 0.23 | -0.25 | -0.51 | -0.45 | 0.60 | 0.32 | -0.21 | -0.44 | -0.31 | 0.47 | 0.36 | -0.44 | -0.49 | -0.41 | 0.74 | 0.21 | -0.01 | 0.27 | 0.21 | -0.21 | -0.19 | 0.22 | -0.17 | 0.03 | -0.45 | 0.18 | -0.27 | -0.21 | 0.22 | 0.19 | 0.19 | 0.15 | 0.02 | 0.09 | 0.09 | -0.11 | -0.13 | 0.08 | -0.18 | 0.11 | 0.58 | 0.54 | 0.17 | -0.12 | 0.06 |
| O\_GT30 | -0.42 | -0.47 | -0.49 | 0.09 | 0.86 | -0.38 | -0.39 | -0.55 | 0.24 | 0.62 | -0.35 | -0.51 | -0.42 | 0.02 | 0.87 | -0.47 | -0.46 | -0.50 | 0.14 | 0.69 | -0.09 | -0.39 | -0.22 | -0.06 | 1.00 | -0.19 | 0.36 | -0.22 | 0.29 | -0.51 | -0.44 | -0.28 | -0.35 | 0.27 | -0.66 | -0.42 | -0.51 | 0.61 | -0.35 | 0.77 | 0.64 | 0.53 | -0.39 | 0.65 | 0.56 | -0.29 | -0.05 | 0.14 | 0.62 | -0.24 | 0.38 | 0.08 | 0.75 | 0.49 | 0.41 | -0.24 | -0.30 | -0.30 | -0.30 | 0.21 | 0.49 | -0.27 | -0.29 | -0.21 | 0.20 | 0.35 | -0.27 | -0.30 | -0.28 | 0.17 | 0.49 | -0.30 | -0.29 | -0.30 | 0.21 | 0.41 | -0.23 | -0.28 | -0.16 | 0.17 | 0.58 | -0.08 | 0.09 | 0.09 | 0.01 | -0.28 | -0.14 | -0.15 | -0.19 | 0.01 | -0.25 | -0.34 | -0.04 | 0.05 | -0.11 | 0.31 | 0.27 | 0.16 | 0.07 | 0.36 | 0.14 | 0.12 | 0.16 | 0.18 | -0.03 | 0.20 | 0.14 | 0.22 | 0.21 | 0.06 |
| GenD\_E | 0.31 | 0.11 | 0.26 | 0.02 | -0.14 | 0.62 | 0.18 | 0.29 | -0.04 | -0.13 | 0.04 | 0.07 | 0.17 | 0.05 | -0.14 | 0.35 | 0.11 | 0.25 | -0.01 | -0.10 | 0.06 | 0.06 | 0.16 | 0.11 | -0.19 | 1.00 | 0.16 | 0.17 | -0.14 | 0.04 | 0.34 | 0.09 | 0.14 | -0.16 | 0.15 | 0.11 | 0.03 | -0.03 | 0.04 | -0.09 | -0.09 | -0.18 | -0.08 | -0.13 | -0.24 | -0.16 | -0.27 | -0.12 | -0.26 | -0.11 | -0.04 | 0.03 | -0.18 | -0.04 | -0.23 | 0.13 | 0.17 | 0.03 | 0.12 | -0.05 | -0.07 | 0.52 | 0.09 | 0.17 | -0.08 | -0.12 | -0.08 | -0.01 | 0.03 | -0.02 | -0.05 | 0.20 | 0.01 | 0.09 | -0.05 | -0.04 | 0.04 | 0.07 | 0.14 | -0.01 | -0.12 | 0.91 | 0.18 | 0.16 | -0.07 | -0.06 | 0.22 | -0.05 | -0.02 | -0.03 | 0.11 | 0.12 | -0.08 | 0.11 | -0.12 | 0.14 | 0.06 | -0.03 | -0.19 | -0.08 | -0.31 | -0.23 | -0.16 | -0.07 | 0.14 | 0.05 | 0.14 | 0.14 | 0.03 | -0.11 |
| GenD\_A | -0.07 | -0.37 | -0.31 | 0.27 | 0.45 | 0.02 | -0.03 | -0.13 | 0.24 | 0.25 | -0.10 | -0.55 | -0.44 | 0.28 | 0.48 | -0.08 | -0.35 | -0.35 | 0.25 | 0.45 | 0.03 | -0.32 | 0.01 | 0.25 | 0.36 | 0.16 | 1.00 | 0.36 | -0.13 | -0.40 | -0.12 | -0.20 | -0.36 | -0.02 | -0.03 | -0.17 | -0.32 | 0.33 | -0.14 | 0.33 | 0.23 | 0.20 | -0.02 | 0.10 | 0.36 | -0.24 | -0.03 | -0.10 | 0.30 | 0.00 | 0.22 | 0.20 | 0.25 | 0.43 | 0.11 | 0.05 | -0.07 | -0.28 | -0.23 | 0.26 | 0.29 | 0.01 | 0.01 | 0.03 | 0.08 | 0.11 | -0.12 | -0.42 | -0.37 | 0.32 | 0.33 | -0.06 | -0.28 | -0.26 | 0.20 | 0.34 | -0.09 | -0.20 | -0.06 | 0.31 | 0.13 | 0.17 | 0.84 | 0.47 | -0.30 | -0.32 | 0.02 | -0.19 | -0.23 | -0.19 | 0.26 | -0.08 | -0.05 | 0.09 | 0.01 | 0.11 | -0.08 | -0.17 | 0.21 | 0.09 | -0.31 | -0.08 | 0.03 | -0.12 | 0.09 | 0.23 | 0.29 | 0.07 | -0.16 | 0.20 |
| GenD\_C | -0.14 | -0.25 | -0.12 | 0.08 | 0.12 | -0.07 | -0.11 | 0.48 | -0.03 | 0.12 | -0.22 | -0.28 | -0.32 | 0.12 | 0.13 | -0.12 | -0.28 | -0.17 | 0.05 | 0.29 | -0.17 | -0.04 | 0.13 | 0.12 | -0.22 | 0.17 | 0.36 | 1.00 | -0.22 | -0.04 | 0.00 | -0.34 | -0.27 | -0.10 | 0.61 | 0.33 | 0.24 | -0.37 | -0.07 | -0.42 | -0.32 | -0.05 | 0.42 | -0.30 | -0.13 | 0.12 | 0.12 | -0.10 | -0.21 | 0.21 | 0.11 | -0.15 | -0.42 | 0.08 | -0.32 | 0.38 | -0.14 | -0.24 | -0.18 | 0.29 | 0.34 | -0.06 | -0.08 | 0.33 | 0.12 | 0.20 | -0.18 | -0.30 | -0.37 | 0.35 | 0.36 | -0.12 | -0.27 | -0.20 | 0.29 | 0.39 | -0.15 | -0.01 | -0.05 | 0.23 | 0.13 | 0.14 | 0.45 | 0.81 | -0.28 | -0.27 | 0.01 | -0.34 | -0.18 | 0.00 | 0.32 | 0.03 | 0.03 | 0.06 | 0.09 | -0.01 | -0.06 | 0.02 | 0.15 | -0.09 | -0.13 | -0.09 | -0.11 | -0.14 | 0.16 | 0.24 | 0.20 | -0.15 | -0.05 | 0.09 |
| GenD\_N | -0.40 | 0.08 | -0.22 | -0.37 | 0.29 | -0.38 | 0.06 | -0.22 | 0.08 | 0.36 | -0.38 | 0.12 | -0.05 | -0.56 | 0.24 | -0.47 | 0.10 | -0.24 | -0.32 | 0.25 | 0.02 | 0.00 | -0.01 | -0.47 | 0.29 | -0.14 | -0.13 | -0.22 | 1.00 | 0.11 | -0.54 | 0.13 | -0.24 | 0.24 | -0.13 | 0.17 | -0.16 | 0.06 | -0.59 | 0.21 | 0.19 | 0.30 | -0.17 | 0.24 | 0.39 | -0.14 | 0.27 | 0.21 | 0.30 | -0.05 | 0.34 | -0.13 | 0.29 | 0.44 | 0.21 | -0.02 | -0.26 | 0.26 | -0.11 | -0.18 | -0.05 | -0.25 | 0.19 | -0.18 | 0.31 | 0.12 | -0.25 | 0.32 | 0.07 | -0.41 | -0.10 | -0.32 | 0.28 | -0.09 | -0.13 | -0.07 | 0.01 | 0.10 | -0.11 | -0.24 | 0.02 | -0.06 | -0.30 | -0.29 | 0.85 | 0.27 | -0.42 | 0.27 | -0.01 | 0.16 | -0.12 | 0.10 | 0.11 | -0.21 | -0.37 | 0.05 | 0.09 | 0.03 | -0.07 | 0.05 | 0.06 | 0.38 | 0.18 | 0.27 | -0.04 | -0.09 | -0.13 | 0.14 | 0.07 | -0.09 |
| GenD\_O | 0.23 | 0.29 | 0.33 | -0.18 | -0.35 | 0.13 | 0.21 | 0.44 | -0.15 | 0.16 | 0.14 | 0.38 | 0.52 | -0.20 | -0.52 | 0.19 | 0.28 | 0.39 | -0.20 | -0.23 | 0.23 | 0.30 | 0.03 | -0.09 | -0.51 | 0.04 | -0.40 | -0.04 | 0.11 | 1.00 | 0.03 | 0.12 | 0.37 | -0.13 | 0.46 | 0.24 | 0.32 | -0.51 | 0.03 | -0.59 | -0.37 | -0.29 | 0.33 | -0.44 | -0.24 | 0.22 | 0.13 | -0.01 | -0.49 | 0.19 | 0.18 | 0.06 | -0.40 | -0.18 | -0.27 | 0.09 | 0.13 | 0.26 | 0.21 | -0.24 | -0.32 | 0.05 | 0.19 | 0.16 | -0.09 | 0.13 | 0.10 | 0.32 | 0.38 | -0.29 | -0.46 | 0.11 | 0.25 | 0.26 | -0.23 | -0.27 | 0.14 | 0.32 | 0.04 | -0.20 | -0.38 | -0.06 | -0.30 | -0.26 | 0.25 | 0.79 | -0.01 | 0.06 | 0.25 | 0.01 | 0.16 | 0.24 | 0.14 | -0.19 | 0.05 | -0.37 | -0.28 | -0.22 | 0.06 | -0.26 | -0.19 | -0.10 | -0.11 | -0.09 | -0.23 | -0.17 | -0.18 | -0.25 | -0.14 | 0.11 |
| AgeD\_E | 0.52 | 0.31 | 0.52 | 0.12 | -0.53 | 0.58 | 0.25 | 0.29 | -0.11 | -0.56 | 0.49 | 0.27 | 0.31 | 0.25 | -0.50 | 0.69 | 0.31 | 0.55 | 0.01 | -0.52 | -0.27 | 0.21 | 0.05 | 0.40 | -0.44 | 0.34 | -0.12 | 0.00 | -0.54 | 0.03 | 1.00 | 0.23 | 0.52 | -0.55 | 0.07 | -0.07 | 0.05 | 0.09 | 0.67 | -0.18 | -0.28 | -0.37 | 0.11 | -0.25 | -0.46 | -0.10 | -0.48 | -0.28 | -0.35 | -0.07 | -0.52 | 0.06 | -0.31 | -0.31 | -0.21 | -0.10 | 0.28 | 0.05 | 0.28 | 0.02 | -0.17 | 0.31 | 0.04 | 0.17 | -0.17 | -0.20 | 0.25 | 0.03 | 0.16 | 0.13 | -0.16 | 0.40 | 0.03 | 0.29 | -0.06 | -0.15 | -0.17 | 0.12 | 0.06 | 0.20 | -0.16 | 0.17 | 0.02 | 0.01 | -0.34 | -0.01 | 0.70 | -0.06 | 0.27 | -0.32 | 0.02 | -0.02 | -0.16 | 0.31 | 0.35 | -0.02 | -0.10 | 0.00 | 0.01 | -0.14 | -0.19 | -0.45 | -0.20 | -0.21 | 0.23 | 0.00 | 0.01 | -0.04 | -0.12 | 0.09 |
| AgeD\_A | 0.47 | 0.68 | 0.43 | -0.10 | -0.50 | 0.43 | 0.63 | 0.06 | 0.00 | -0.48 | 0.48 | 0.63 | 0.35 | -0.08 | -0.49 | 0.46 | 0.78 | 0.49 | -0.14 | -0.56 | 0.33 | 0.03 | -0.07 | 0.02 | -0.28 | 0.09 | -0.20 | -0.34 | 0.13 | 0.12 | 0.23 | 1.00 | 0.54 | -0.21 | -0.19 | 0.01 | -0.07 | 0.05 | 0.18 | -0.07 | 0.06 | 0.00 | -0.21 | -0.21 | -0.13 | 0.14 | -0.02 | 0.03 | -0.27 | -0.05 | -0.17 | 0.16 | -0.16 | -0.10 | -0.02 | -0.17 | 0.29 | 0.60 | 0.28 | -0.31 | -0.46 | 0.24 | 0.53 | -0.09 | -0.09 | -0.47 | 0.33 | 0.56 | 0.23 | -0.32 | -0.43 | 0.25 | 0.68 | 0.33 | -0.31 | -0.52 | 0.37 | 0.00 | -0.06 | -0.21 | -0.23 | -0.04 | -0.20 | -0.37 | 0.28 | 0.06 | -0.08 | 0.88 | 0.41 | -0.06 | -0.35 | 0.20 | -0.11 | 0.09 | -0.04 | 0.00 | 0.16 | 0.26 | -0.31 | -0.18 | 0.15 | 0.11 | -0.01 | 0.09 | 0.12 | -0.31 | -0.10 | 0.12 | 0.20 | -0.24 |
| AgeD\_C | 0.43 | 0.58 | 0.60 | 0.03 | -0.48 | 0.40 | 0.47 | 0.33 | -0.07 | -0.32 | 0.40 | 0.59 | 0.58 | 0.09 | -0.52 | 0.49 | 0.60 | 0.75 | -0.04 | -0.50 | 0.03 | 0.30 | -0.34 | 0.22 | -0.35 | 0.14 | -0.36 | -0.27 | -0.24 | 0.37 | 0.52 | 0.54 | 1.00 | -0.37 | -0.03 | -0.07 | 0.09 | 0.00 | 0.57 | -0.23 | -0.16 | -0.25 | -0.01 | -0.25 | -0.44 | 0.02 | -0.30 | -0.09 | -0.49 | -0.05 | -0.28 | 0.18 | -0.19 | -0.43 | -0.14 | -0.20 | 0.16 | 0.35 | 0.34 | -0.13 | -0.31 | 0.14 | 0.28 | 0.16 | -0.11 | -0.22 | 0.18 | 0.35 | 0.29 | -0.09 | -0.34 | 0.20 | 0.36 | 0.46 | -0.18 | -0.33 | -0.02 | 0.17 | -0.24 | 0.03 | -0.23 | -0.02 | -0.19 | -0.16 | 0.00 | 0.22 | 0.27 | 0.33 | 0.71 | -0.22 | -0.11 | 0.17 | -0.06 | 0.20 | 0.32 | -0.17 | -0.11 | 0.10 | -0.08 | -0.20 | -0.18 | -0.29 | -0.19 | -0.05 | 0.19 | -0.14 | -0.07 | -0.08 | 0.17 | -0.08 |
| AgeD\_N | -0.16 | -0.02 | -0.30 | 0.10 | 0.22 | -0.23 | 0.00 | -0.19 | 0.21 | 0.18 | -0.17 | 0.01 | -0.12 | 0.02 | 0.24 | -0.28 | -0.06 | -0.33 | 0.29 | 0.17 | 0.29 | 0.17 | 0.04 | -0.44 | 0.27 | -0.16 | -0.02 | -0.10 | 0.24 | -0.13 | -0.55 | -0.21 | -0.37 | 1.00 | -0.20 | -0.21 | -0.10 | 0.05 | -0.40 | 0.32 | 0.40 | 0.46 | -0.25 | 0.11 | 0.40 | 0.24 | 0.46 | 0.33 | 0.27 | 0.18 | 0.11 | 0.24 | 0.29 | 0.14 | 0.31 | 0.06 | -0.05 | 0.06 | -0.17 | -0.03 | -0.03 | -0.08 | 0.03 | -0.04 | 0.05 | 0.00 | -0.08 | 0.12 | -0.04 | -0.08 | -0.01 | -0.13 | 0.05 | -0.20 | 0.19 | -0.04 | 0.20 | 0.14 | 0.04 | -0.50 | 0.01 | -0.03 | -0.18 | 0.00 | 0.16 | 0.01 | -0.38 | -0.06 | -0.25 | 0.82 | -0.06 | -0.03 | 0.14 | -0.29 | -0.33 | 0.09 | 0.11 | 0.19 | -0.08 | 0.09 | 0.27 | 0.40 | 0.17 | 0.27 | -0.16 | -0.06 | -0.01 | 0.20 | 0.16 | -0.10 |
| AgeD\_O | -0.05 | -0.03 | 0.08 | -0.05 | -0.18 | -0.04 | 0.01 | 0.58 | -0.15 | 0.07 | -0.17 | 0.03 | 0.12 | -0.04 | -0.24 | -0.03 | -0.06 | 0.07 | -0.10 | 0.09 | -0.12 | 0.14 | 0.15 | 0.05 | -0.66 | 0.15 | -0.03 | 0.61 | -0.13 | 0.46 | 0.07 | -0.19 | -0.03 | -0.20 | 1.00 | 0.41 | 0.41 | -0.74 | -0.10 | -0.77 | -0.55 | -0.35 | 0.59 | -0.51 | -0.31 | 0.29 | 0.26 | -0.13 | -0.55 | 0.34 | 0.07 | -0.12 | -0.66 | -0.14 | -0.41 | 0.44 | -0.11 | -0.12 | -0.12 | 0.15 | 0.07 | -0.08 | -0.04 | 0.13 | 0.09 | 0.16 | -0.16 | -0.13 | -0.09 | 0.16 | 0.04 | -0.09 | -0.15 | -0.12 | 0.14 | 0.20 | -0.12 | 0.06 | -0.04 | 0.15 | -0.27 | 0.07 | 0.19 | 0.24 | -0.08 | 0.12 | 0.02 | -0.24 | -0.09 | -0.04 | 0.60 | 0.10 | 0.03 | -0.18 | -0.01 | -0.22 | -0.22 | -0.27 | 0.16 | -0.20 | -0.19 | -0.05 | -0.12 | -0.19 | -0.06 | 0.17 | 0.13 | -0.26 | -0.27 | 0.10 |
| TFR | -0.06 | 0.16 | 0.19 | -0.60 | -0.27 | -0.01 | 0.12 | 0.36 | -0.56 | -0.15 | -0.10 | 0.19 | 0.11 | -0.57 | -0.29 | -0.07 | 0.14 | 0.14 | -0.62 | -0.16 | -0.01 | 0.21 | 0.29 | -0.43 | -0.42 | 0.11 | -0.17 | 0.33 | 0.17 | 0.24 | -0.07 | 0.01 | -0.07 | -0.21 | 0.41 | 1.00 | 0.41 | -0.43 | -0.10 | -0.58 | -0.75 | -0.30 | 0.32 | -0.25 | -0.31 | -0.32 | -0.20 | -0.17 | -0.07 | -0.19 | 0.16 | -0.65 | -0.28 | 0.05 | -0.38 | 0.38 | 0.16 | 0.33 | 0.28 | -0.32 | -0.35 | 0.16 | 0.33 | 0.27 | -0.25 | -0.25 | 0.12 | 0.32 | 0.24 | -0.29 | -0.35 | 0.14 | 0.32 | 0.29 | -0.31 | -0.31 | 0.18 | 0.32 | 0.17 | -0.24 | -0.38 | 0.09 | -0.06 | 0.03 | 0.08 | 0.20 | -0.02 | 0.15 | 0.16 | -0.02 | 0.11 | 0.65 | 0.16 | -0.12 | 0.12 | -0.49 | -0.43 | -0.14 | 0.04 | -0.46 | -0.25 | -0.12 | -0.13 | -0.10 | -0.04 | -0.32 | -0.28 | -0.53 | -0.05 | 0.10 |
| alpha | -0.08 | 0.11 | 0.06 | -0.08 | -0.39 | -0.10 | 0.05 | 0.33 | -0.20 | -0.21 | -0.15 | 0.20 | 0.16 | -0.07 | -0.39 | -0.07 | 0.09 | 0.09 | -0.10 | -0.29 | -0.14 | 0.22 | 0.00 | -0.02 | -0.51 | 0.03 | -0.32 | 0.24 | -0.16 | 0.32 | 0.05 | -0.07 | 0.09 | -0.10 | 0.41 | 0.41 | 1.00 | -0.76 | 0.05 | -0.65 | -0.62 | -0.56 | 0.58 | -0.18 | -0.58 | 0.17 | -0.07 | -0.02 | -0.48 | -0.08 | -0.20 | -0.35 | -0.61 | -0.54 | -0.38 | 0.30 | -0.11 | 0.05 | -0.03 | -0.15 | -0.03 | -0.14 | 0.06 | 0.09 | -0.10 | 0.06 | -0.13 | 0.07 | 0.07 | -0.17 | -0.03 | -0.13 | 0.03 | -0.03 | -0.12 | -0.02 | -0.02 | 0.15 | 0.02 | -0.19 | -0.04 | -0.06 | -0.04 | 0.02 | 0.09 | 0.12 | -0.15 | -0.08 | -0.06 | 0.11 | 0.04 | 0.16 | 0.67 | -0.44 | -0.21 | -0.18 | -0.19 | -0.14 | 0.25 | -0.01 | 0.00 | -0.03 | -0.11 | -0.02 | -0.39 | -0.16 | -0.20 | -0.25 | -0.18 | 0.18 |
| peak | 0.12 | -0.03 | -0.04 | 0.12 | 0.29 | 0.17 | 0.02 | -0.47 | 0.20 | 0.00 | 0.23 | -0.16 | -0.21 | 0.13 | 0.34 | 0.14 | -0.02 | -0.05 | 0.13 | 0.10 | 0.07 | -0.10 | -0.07 | 0.08 | 0.61 | -0.03 | 0.33 | -0.37 | 0.06 | -0.51 | 0.09 | 0.05 | 0.00 | 0.05 | -0.74 | -0.43 | -0.76 | 1.00 | 0.22 | 0.87 | 0.61 | 0.46 | -0.71 | 0.38 | 0.44 | -0.45 | -0.31 | -0.15 | 0.67 | -0.21 | -0.05 | 0.24 | 0.78 | 0.40 | 0.44 | -0.42 | 0.16 | 0.03 | 0.12 | 0.05 | -0.04 | 0.19 | 0.02 | 0.01 | 0.00 | -0.12 | 0.18 | -0.01 | -0.02 | 0.09 | -0.03 | 0.19 | 0.03 | 0.12 | 0.01 | -0.06 | 0.04 | -0.01 | 0.01 | 0.14 | 0.04 | 0.05 | 0.05 | 0.03 | -0.11 | -0.11 | 0.20 | 0.05 | 0.13 | -0.16 | -0.13 | -0.08 | -0.29 | 0.45 | 0.31 | 0.17 | 0.14 | 0.18 | -0.22 | 0.03 | -0.04 | -0.12 | 0.04 | -0.10 | 0.37 | 0.07 | 0.11 | 0.21 | 0.21 | -0.16 |
| stop | 0.46 | 0.28 | 0.40 | 0.31 | -0.52 | 0.42 | 0.20 | 0.12 | 0.06 | -0.55 | 0.51 | 0.24 | 0.19 | 0.43 | -0.49 | 0.56 | 0.27 | 0.47 | 0.22 | -0.55 | -0.05 | 0.21 | -0.15 | 0.50 | -0.35 | 0.04 | -0.14 | -0.07 | -0.59 | 0.03 | 0.67 | 0.18 | 0.57 | -0.40 | -0.10 | -0.10 | 0.05 | 0.22 | 1.00 | -0.11 | -0.24 | -0.37 | -0.01 | -0.27 | -0.48 | -0.07 | -0.45 | -0.27 | -0.27 | -0.11 | -0.58 | 0.11 | -0.21 | -0.54 | -0.17 | -0.13 | 0.25 | 0.06 | 0.25 | 0.02 | -0.16 | 0.19 | 0.03 | 0.14 | -0.13 | -0.17 | 0.31 | 0.02 | 0.07 | 0.13 | -0.17 | 0.30 | 0.04 | 0.28 | -0.04 | -0.15 | 0.02 | 0.10 | -0.01 | 0.18 | -0.14 | -0.09 | 0.01 | 0.07 | -0.30 | 0.04 | 0.35 | -0.03 | 0.32 | -0.28 | -0.01 | 0.13 | -0.22 | 0.48 | 0.69 | -0.14 | -0.17 | 0.03 | -0.03 | -0.22 | -0.16 | -0.36 | -0.17 | -0.25 | 0.41 | 0.01 | 0.06 | -0.08 | 0.03 | -0.04 |
| ageFB | -0.11 | -0.19 | -0.31 | 0.20 | 0.48 | -0.07 | -0.13 | -0.63 | 0.34 | 0.17 | -0.02 | -0.28 | -0.33 | 0.15 | 0.54 | -0.13 | -0.18 | -0.32 | 0.25 | 0.28 | 0.04 | -0.20 | -0.14 | 0.01 | 0.77 | -0.09 | 0.33 | -0.42 | 0.21 | -0.59 | -0.18 | -0.07 | -0.23 | 0.32 | -0.77 | -0.58 | -0.65 | 0.87 | -0.11 | 1.00 | 0.76 | 0.51 | -0.67 | 0.54 | 0.56 | -0.35 | -0.13 | 0.02 | 0.75 | -0.18 | 0.01 | 0.25 | 0.85 | 0.39 | 0.54 | -0.41 | -0.02 | -0.10 | -0.10 | 0.15 | 0.13 | 0.02 | -0.10 | -0.13 | 0.16 | 0.03 | -0.02 | -0.11 | -0.13 | 0.13 | 0.16 | -0.02 | -0.09 | -0.12 | 0.15 | 0.10 | -0.02 | -0.14 | -0.05 | 0.10 | 0.20 | 0.06 | 0.05 | 0.00 | 0.02 | -0.18 | -0.01 | 0.00 | -0.09 | 0.04 | -0.14 | -0.28 | -0.10 | 0.15 | -0.08 | 0.37 | 0.28 | 0.12 | -0.13 | 0.21 | 0.07 | 0.06 | 0.15 | 0.05 | 0.10 | 0.16 | 0.18 | 0.41 | 0.15 | -0.11 |
| t\_ageFM | -0.08 | -0.12 | -0.27 | 0.36 | 0.45 | -0.07 | -0.07 | -0.45 | 0.49 | 0.27 | -0.02 | -0.18 | -0.22 | 0.29 | 0.48 | -0.13 | -0.10 | -0.27 | 0.42 | 0.32 | 0.16 | -0.23 | -0.15 | 0.11 | 0.64 | -0.09 | 0.23 | -0.32 | 0.19 | -0.37 | -0.28 | 0.06 | -0.16 | 0.40 | -0.55 | -0.75 | -0.62 | 0.61 | -0.24 | 0.76 | 1.00 | 0.73 | -0.67 | 0.30 | 0.63 | 0.22 | 0.33 | 0.15 | 0.40 | 0.19 | 0.14 | 0.56 | 0.55 | 0.35 | 0.51 | -0.29 | -0.10 | -0.09 | -0.16 | 0.21 | 0.17 | -0.04 | -0.13 | -0.23 | 0.24 | 0.05 | -0.08 | -0.09 | -0.18 | 0.17 | 0.19 | -0.11 | -0.07 | -0.18 | 0.22 | 0.12 | -0.05 | -0.24 | -0.11 | 0.12 | 0.28 | 0.04 | -0.06 | -0.05 | 0.07 | -0.21 | -0.09 | 0.11 | -0.10 | 0.08 | -0.22 | -0.40 | -0.17 | 0.19 | -0.15 | 0.45 | 0.61 | 0.37 | -0.34 | 0.31 | 0.32 | 0.24 | 0.19 | 0.07 | 0.09 | 0.23 | 0.25 | 0.47 | 0.26 | -0.31 |
| nevermar | -0.13 | -0.09 | -0.24 | 0.05 | 0.44 | -0.15 | -0.05 | -0.29 | 0.22 | 0.29 | -0.06 | -0.14 | -0.28 | 0.00 | 0.45 | -0.20 | -0.08 | -0.28 | 0.14 | 0.36 | 0.18 | -0.13 | -0.04 | -0.19 | 0.53 | -0.18 | 0.20 | -0.05 | 0.30 | -0.29 | -0.37 | 0.00 | -0.25 | 0.46 | -0.35 | -0.30 | -0.56 | 0.46 | -0.37 | 0.51 | 0.73 | 1.00 | -0.52 | 0.16 | 0.68 | 0.22 | 0.43 | 0.19 | 0.47 | 0.35 | 0.34 | 0.37 | 0.49 | 0.59 | 0.35 | 0.00 | 0.00 | 0.06 | -0.07 | 0.11 | 0.01 | 0.01 | 0.00 | -0.16 | 0.14 | -0.11 | 0.03 | 0.06 | -0.16 | 0.11 | 0.04 | 0.00 | 0.08 | -0.06 | 0.15 | -0.05 | 0.00 | -0.09 | -0.17 | 0.01 | 0.17 | -0.02 | -0.12 | 0.01 | 0.02 | -0.17 | 0.00 | 0.18 | 0.09 | 0.14 | -0.28 | -0.13 | -0.13 | 0.25 | 0.03 | 0.20 | 0.37 | 0.61 | -0.32 | 0.17 | 0.30 | 0.17 | 0.12 | 0.10 | 0.15 | 0.09 | 0.05 | 0.11 | 0.46 | -0.25 |
| divorce | -0.14 | -0.15 | -0.02 | -0.02 | -0.11 | -0.20 | -0.12 | 0.39 | -0.13 | 0.09 | -0.20 | -0.09 | 0.07 | -0.01 | -0.14 | -0.10 | -0.17 | 0.00 | -0.08 | 0.05 | -0.27 | -0.01 | 0.01 | 0.11 | -0.39 | -0.08 | -0.02 | 0.42 | -0.17 | 0.33 | 0.11 | -0.21 | -0.01 | -0.25 | 0.59 | 0.32 | 0.58 | -0.71 | -0.01 | -0.67 | -0.67 | -0.52 | 1.00 | -0.26 | -0.37 | 0.19 | 0.01 | 0.07 | -0.44 | 0.14 | 0.00 | -0.19 | -0.59 | -0.22 | -0.32 | 0.24 | -0.20 | -0.22 | -0.15 | 0.07 | 0.23 | -0.27 | -0.13 | 0.10 | 0.01 | 0.31 | -0.20 | -0.21 | -0.03 | 0.06 | 0.21 | -0.18 | -0.23 | -0.13 | 0.04 | 0.26 | -0.23 | -0.04 | -0.07 | 0.09 | 0.10 | -0.16 | 0.19 | 0.14 | -0.07 | 0.06 | 0.01 | -0.27 | -0.08 | -0.07 | 0.21 | 0.05 | 0.29 | -0.38 | -0.04 | -0.26 | -0.44 | -0.41 | 0.77 | 0.01 | -0.14 | -0.20 | -0.14 | 0.04 | -0.30 | 0.01 | -0.09 | -0.28 | -0.38 | 0.70 |
| cohabit | -0.37 | -0.39 | -0.42 | -0.11 | 0.51 | -0.33 | -0.39 | -0.53 | -0.02 | 0.30 | -0.32 | -0.38 | -0.32 | -0.17 | 0.55 | -0.38 | -0.38 | -0.42 | -0.09 | 0.38 | -0.21 | -0.35 | -0.25 | -0.16 | 0.65 | -0.13 | 0.10 | -0.30 | 0.24 | -0.44 | -0.25 | -0.21 | -0.25 | 0.11 | -0.51 | -0.25 | -0.18 | 0.38 | -0.27 | 0.54 | 0.30 | 0.16 | -0.26 | 1.00 | 0.23 | -0.37 | -0.28 | 0.06 | 0.43 | -0.55 | 0.16 | -0.38 | 0.49 | 0.17 | 0.32 | -0.28 | -0.18 | -0.30 | -0.30 | 0.17 | 0.34 | -0.18 | -0.28 | -0.29 | 0.16 | 0.23 | -0.17 | -0.28 | -0.22 | 0.12 | 0.35 | -0.20 | -0.29 | -0.31 | 0.18 | 0.27 | -0.11 | -0.28 | -0.16 | 0.09 | 0.43 | -0.06 | 0.08 | -0.07 | 0.04 | -0.23 | -0.14 | -0.14 | -0.20 | 0.07 | -0.22 | -0.48 | -0.01 | 0.04 | -0.22 | 0.40 | 0.35 | 0.19 | 0.01 | 0.69 | 0.26 | 0.14 | 0.28 | 0.22 | -0.08 | 0.21 | 0.04 | 0.28 | 0.28 | -0.04 |
| abortion | -0.22 | -0.32 | -0.39 | 0.13 | 0.52 | -0.26 | -0.22 | -0.42 | 0.33 | 0.40 | -0.14 | -0.37 | -0.34 | 0.03 | 0.51 | -0.30 | -0.30 | -0.44 | 0.20 | 0.44 | 0.16 | -0.32 | 0.00 | -0.10 | 0.56 | -0.24 | 0.36 | -0.13 | 0.39 | -0.24 | -0.46 | -0.13 | -0.44 | 0.40 | -0.31 | -0.31 | -0.58 | 0.44 | -0.48 | 0.56 | 0.63 | 0.68 | -0.37 | 0.23 | 1.00 | 0.06 | 0.45 | 0.15 | 0.52 | 0.26 | 0.40 | 0.37 | 0.50 | 0.66 | 0.41 | -0.13 | -0.12 | -0.16 | -0.20 | 0.17 | 0.11 | -0.14 | -0.16 | -0.24 | 0.22 | 0.07 | -0.09 | -0.15 | -0.16 | 0.10 | 0.12 | -0.15 | -0.14 | -0.22 | 0.19 | 0.08 | -0.01 | -0.20 | -0.09 | 0.06 | 0.17 | -0.10 | 0.02 | -0.08 | 0.13 | -0.09 | -0.17 | -0.01 | -0.16 | 0.12 | -0.11 | -0.12 | -0.10 | 0.05 | -0.15 | 0.24 | 0.18 | 0.12 | -0.10 | 0.24 | 0.10 | 0.30 | 0.60 | 0.07 | -0.08 | 0.21 | 0.09 | 0.20 | 0.02 | -0.01 |
| t\_nmf | 0.12 | 0.10 | 0.05 | 0.30 | -0.18 | -0.02 | 0.06 | 0.24 | 0.23 | -0.04 | 0.10 | 0.18 | 0.16 | 0.28 | -0.18 | 0.07 | 0.12 | 0.06 | 0.33 | -0.10 | 0.22 | 0.02 | 0.05 | 0.14 | -0.29 | -0.16 | -0.24 | 0.12 | -0.14 | 0.22 | -0.10 | 0.14 | 0.02 | 0.24 | 0.29 | -0.32 | 0.17 | -0.45 | -0.07 | -0.35 | 0.22 | 0.22 | 0.19 | -0.37 | 0.06 | 1.00 | 0.72 | 0.24 | -0.51 | 0.64 | -0.02 | 0.52 | -0.49 | -0.23 | -0.03 | 0.18 | -0.05 | -0.04 | -0.11 | 0.03 | 0.04 | -0.12 | -0.12 | -0.14 | 0.05 | -0.03 | 0.00 | 0.00 | -0.07 | 0.01 | 0.07 | -0.08 | -0.02 | -0.13 | 0.08 | 0.00 | 0.04 | -0.15 | -0.01 | -0.08 | 0.13 | -0.18 | -0.19 | -0.08 | 0.04 | -0.13 | -0.15 | 0.09 | -0.14 | 0.18 | -0.16 | -0.21 | 0.00 | -0.05 | -0.12 | 0.11 | 0.28 | 0.26 | -0.09 | 0.20 | 0.54 | 0.26 | 0.09 | 0.06 | -0.08 | 0.00 | -0.07 | 0.09 | 0.03 | -0.12 |
| unintprg | -0.20 | -0.05 | -0.23 | 0.14 | 0.11 | -0.30 | -0.02 | 0.00 | 0.26 | 0.20 | -0.18 | 0.00 | -0.11 | 0.05 | 0.09 | -0.29 | -0.04 | -0.26 | 0.22 | 0.18 | 0.19 | -0.04 | 0.05 | -0.12 | -0.05 | -0.27 | -0.03 | 0.12 | 0.27 | 0.13 | -0.48 | -0.02 | -0.30 | 0.46 | 0.26 | -0.20 | -0.07 | -0.31 | -0.45 | -0.13 | 0.33 | 0.43 | 0.01 | -0.28 | 0.45 | 0.72 | 1.00 | 0.35 | -0.13 | 0.70 | 0.14 | 0.43 | -0.18 | 0.10 | 0.11 | 0.15 | -0.23 | -0.07 | -0.27 | 0.08 | 0.11 | -0.25 | -0.08 | -0.27 | 0.22 | 0.08 | -0.18 | -0.04 | -0.20 | -0.02 | 0.12 | -0.29 | -0.04 | -0.30 | 0.15 | 0.10 | 0.03 | -0.16 | -0.08 | -0.11 | 0.13 | -0.16 | -0.06 | -0.07 | 0.27 | -0.08 | -0.39 | 0.07 | -0.25 | 0.30 | -0.05 | -0.11 | -0.03 | -0.16 | -0.31 | 0.10 | 0.24 | 0.17 | -0.16 | 0.13 | 0.29 | 0.61 | 0.31 | 0.17 | -0.12 | 0.07 | 0.01 | 0.13 | -0.02 | -0.18 |
| famplnpw | -0.21 | 0.02 | -0.19 | 0.06 | 0.09 | -0.23 | 0.01 | -0.12 | 0.15 | 0.11 | -0.21 | 0.06 | -0.05 | -0.01 | 0.10 | -0.25 | 0.03 | -0.17 | 0.13 | 0.06 | 0.01 | 0.01 | -0.11 | -0.12 | 0.14 | -0.12 | -0.10 | -0.10 | 0.21 | -0.01 | -0.28 | 0.03 | -0.09 | 0.33 | -0.13 | -0.17 | -0.02 | -0.15 | -0.27 | 0.02 | 0.15 | 0.19 | 0.07 | 0.06 | 0.15 | 0.24 | 0.35 | 1.00 | 0.00 | 0.24 | 0.04 | 0.17 | -0.03 | -0.03 | -0.04 | 0.04 | -0.25 | 0.07 | -0.17 | -0.07 | 0.13 | -0.24 | 0.05 | -0.17 | 0.09 | 0.10 | -0.24 | 0.11 | -0.02 | -0.16 | 0.16 | -0.28 | 0.08 | -0.16 | 0.02 | 0.07 | -0.08 | 0.01 | -0.11 | -0.23 | 0.29 | -0.07 | -0.14 | -0.17 | 0.30 | -0.10 | -0.28 | 0.10 | -0.07 | 0.31 | -0.30 | -0.14 | -0.03 | -0.21 | -0.35 | 0.14 | 0.11 | 0.16 | 0.05 | 0.31 | 0.10 | 0.26 | 0.11 | 0.94 | -0.03 | -0.04 | 0.06 | 0.20 | 0.26 | -0.03 |
| med\_inc | -0.34 | -0.31 | -0.43 | -0.12 | 0.44 | -0.31 | -0.27 | -0.61 | 0.04 | 0.18 | -0.20 | -0.38 | -0.49 | -0.16 | 0.48 | -0.36 | -0.33 | -0.50 | -0.06 | 0.29 | -0.07 | -0.19 | -0.02 | -0.25 | 0.62 | -0.26 | 0.30 | -0.21 | 0.30 | -0.49 | -0.35 | -0.27 | -0.49 | 0.27 | -0.55 | -0.07 | -0.48 | 0.67 | -0.27 | 0.75 | 0.40 | 0.47 | -0.44 | 0.43 | 0.52 | -0.51 | -0.13 | 0.00 | 1.00 | -0.15 | 0.15 | -0.13 | 0.81 | 0.54 | 0.40 | -0.23 | 0.00 | 0.00 | 0.00 | 0.00 | 0.00 | 0.00 | 0.00 | 0.00 | 0.00 | 0.00 | 0.00 | 0.00 | 0.00 | 0.00 | 0.00 | 0.00 | 0.00 | 0.00 | 0.00 | 0.00 | 0.00 | 0.00 | 0.00 | 0.00 | 0.00 | 0.00 | 0.00 | 0.00 | 0.00 | 0.00 | 0.00 | 0.00 | 0.00 | 0.00 | 0.00 | 0.00 | 0.00 | 0.00 | 0.00 | 0.00 | 0.00 | 0.00 | 0.00 | 0.00 | 0.00 | 0.00 | 0.00 | 0.00 | -0.01 | 0.00 | 0.05 | 0.03 | 0.10 | 0.01 |
| perAA | 0.10 | 0.12 | 0.12 | 0.09 | -0.09 | 0.02 | 0.14 | 0.27 | 0.07 | 0.02 | 0.10 | 0.12 | 0.11 | 0.09 | -0.11 | 0.07 | 0.09 | 0.09 | 0.12 | 0.01 | 0.17 | 0.21 | 0.20 | -0.01 | -0.24 | -0.11 | 0.00 | 0.21 | -0.05 | 0.19 | -0.07 | -0.05 | -0.05 | 0.18 | 0.34 | -0.19 | -0.08 | -0.21 | -0.11 | -0.18 | 0.19 | 0.35 | 0.14 | -0.55 | 0.26 | 0.64 | 0.70 | 0.24 | -0.15 | 1.00 | -0.12 | 0.60 | -0.18 | 0.04 | -0.11 | 0.25 | 0.00 | 0.00 | 0.00 | 0.00 | 0.00 | 0.00 | 0.00 | 0.00 | 0.00 | 0.00 | 0.00 | 0.00 | 0.00 | 0.00 | 0.00 | 0.00 | 0.00 | 0.00 | 0.00 | 0.00 | 0.00 | 0.00 | 0.00 | 0.00 | 0.00 | 0.00 | 0.00 | 0.00 | 0.00 | 0.00 | 0.00 | 0.00 | 0.00 | 0.00 | 0.00 | 0.00 | 0.00 | 0.00 | 0.00 | 0.00 | 0.00 | 0.00 | 0.00 | 0.00 | 0.00 | 0.00 | 0.00 | 0.00 | 0.01 | 0.00 | -0.02 | 0.03 | -0.14 | -0.02 |
| perHisp | -0.20 | -0.35 | -0.25 | -0.22 | 0.54 | -0.21 | -0.26 | -0.03 | -0.08 | 0.66 | -0.24 | -0.34 | -0.12 | -0.28 | 0.45 | -0.31 | -0.33 | -0.27 | -0.19 | 0.56 | 0.21 | -0.32 | 0.01 | -0.26 | 0.38 | -0.04 | 0.22 | 0.11 | 0.34 | 0.18 | -0.52 | -0.17 | -0.28 | 0.11 | 0.07 | 0.16 | -0.20 | -0.05 | -0.58 | 0.01 | 0.14 | 0.34 | 0.00 | 0.16 | 0.40 | -0.02 | 0.14 | 0.04 | 0.15 | -0.12 | 1.00 | -0.10 | 0.18 | 0.61 | 0.12 | 0.01 | 0.00 | 0.00 | 0.00 | 0.00 | 0.00 | 0.00 | 0.00 | 0.00 | 0.00 | 0.00 | 0.00 | 0.00 | 0.00 | 0.00 | 0.00 | 0.00 | 0.00 | 0.00 | 0.00 | 0.00 | 0.00 | 0.00 | 0.00 | 0.00 | 0.00 | 0.00 | 0.00 | 0.00 | 0.00 | 0.00 | 0.00 | 0.00 | 0.00 | 0.00 | 0.00 | 0.00 | 0.00 | 0.00 | 0.00 | 0.00 | 0.00 | 0.00 | 0.00 | 0.00 | 0.00 | 0.00 | 0.00 | 0.00 | -0.09 | 0.00 | -0.05 | -0.12 | 0.04 | 0.11 |
| perFem | 0.32 | 0.18 | 0.15 | 0.49 | 0.02 | 0.25 | 0.28 | 0.05 | 0.48 | 0.04 | 0.30 | 0.13 | 0.18 | 0.48 | 0.00 | 0.30 | 0.20 | 0.18 | 0.52 | -0.01 | 0.33 | 0.12 | 0.00 | 0.32 | 0.08 | 0.03 | 0.20 | -0.15 | -0.13 | 0.06 | 0.06 | 0.16 | 0.18 | 0.24 | -0.12 | -0.65 | -0.35 | 0.24 | 0.11 | 0.25 | 0.56 | 0.37 | -0.19 | -0.38 | 0.37 | 0.52 | 0.43 | 0.17 | -0.13 | 0.60 | -0.10 | 1.00 | 0.09 | 0.08 | 0.23 | -0.13 | 0.00 | 0.00 | 0.00 | 0.00 | 0.00 | 0.00 | 0.00 | 0.00 | 0.00 | 0.00 | 0.00 | 0.00 | 0.00 | 0.00 | 0.00 | 0.00 | 0.00 | 0.00 | 0.00 | 0.00 | 0.00 | 0.00 | 0.00 | 0.00 | 0.00 | 0.00 | 0.00 | 0.00 | 0.00 | 0.00 | 0.00 | 0.00 | 0.00 | 0.00 | 0.00 | 0.00 | 0.00 | 0.00 | 0.00 | 0.00 | 0.00 | 0.00 | 0.00 | 0.00 | 0.00 | 0.00 | 0.00 | 0.00 | 0.03 | 0.00 | 0.10 | 0.13 | -0.09 | 0.01 |
| perBA | -0.18 | -0.15 | -0.22 | -0.13 | 0.54 | -0.17 | -0.11 | -0.51 | 0.02 | 0.32 | -0.08 | -0.22 | -0.20 | -0.17 | 0.55 | -0.22 | -0.17 | -0.24 | -0.07 | 0.36 | 0.07 | -0.07 | -0.08 | -0.27 | 0.75 | -0.18 | 0.25 | -0.42 | 0.29 | -0.40 | -0.31 | -0.16 | -0.19 | 0.29 | -0.66 | -0.28 | -0.61 | 0.78 | -0.21 | 0.85 | 0.55 | 0.49 | -0.59 | 0.49 | 0.50 | -0.49 | -0.18 | -0.03 | 0.81 | -0.18 | 0.18 | 0.09 | 1.00 | 0.47 | 0.44 | -0.36 | 0.00 | 0.00 | 0.00 | 0.00 | 0.00 | 0.00 | 0.00 | 0.00 | 0.00 | 0.00 | 0.00 | 0.00 | 0.00 | 0.00 | 0.00 | 0.00 | 0.00 | 0.00 | 0.00 | 0.00 | 0.00 | 0.00 | 0.00 | 0.00 | 0.00 | 0.00 | 0.00 | 0.00 | 0.00 | 0.00 | 0.00 | 0.00 | 0.00 | 0.00 | 0.00 | 0.00 | 0.00 | 0.00 | 0.00 | 0.00 | 0.00 | 0.00 | 0.00 | 0.00 | 0.00 | 0.00 | 0.00 | 0.00 | 0.01 | 0.00 | 0.04 | 0.04 | 0.10 | 0.01 |
| perUrb | -0.15 | -0.25 | -0.19 | -0.26 | 0.54 | -0.12 | -0.13 | -0.16 | -0.03 | 0.45 | -0.12 | -0.33 | -0.24 | -0.31 | 0.51 | -0.20 | -0.24 | -0.27 | -0.22 | 0.52 | 0.10 | -0.26 | 0.22 | -0.31 | 0.49 | -0.04 | 0.43 | 0.08 | 0.44 | -0.18 | -0.31 | -0.10 | -0.43 | 0.14 | -0.14 | 0.05 | -0.54 | 0.40 | -0.54 | 0.39 | 0.35 | 0.59 | -0.22 | 0.17 | 0.66 | -0.23 | 0.10 | -0.03 | 0.54 | 0.04 | 0.61 | 0.08 | 0.47 | 1.00 | 0.24 | 0.00 | 0.00 | 0.00 | 0.00 | 0.00 | 0.00 | 0.00 | 0.00 | 0.00 | 0.00 | 0.00 | 0.00 | 0.00 | 0.00 | 0.00 | 0.00 | 0.00 | 0.00 | 0.00 | 0.00 | 0.00 | 0.00 | 0.00 | 0.00 | 0.00 | 0.00 | 0.00 | 0.00 | 0.00 | 0.00 | 0.00 | 0.00 | 0.00 | 0.00 | 0.00 | 0.00 | 0.00 | 0.00 | 0.00 | 0.00 | 0.00 | 0.00 | 0.00 | 0.00 | 0.00 | 0.00 | 0.00 | 0.00 | 0.00 | -0.04 | 0.00 | -0.02 | -0.04 | 0.05 | 0.08 |
| voteO | -0.17 | -0.04 | -0.24 | 0.10 | 0.25 | -0.21 | 0.00 | -0.38 | 0.21 | 0.13 | -0.09 | -0.05 | -0.14 | 0.04 | 0.29 | -0.19 | -0.03 | -0.23 | 0.15 | 0.14 | 0.00 | -0.03 | -0.13 | -0.08 | 0.41 | -0.23 | 0.11 | -0.32 | 0.21 | -0.27 | -0.21 | -0.02 | -0.14 | 0.31 | -0.41 | -0.38 | -0.38 | 0.44 | -0.17 | 0.54 | 0.51 | 0.35 | -0.32 | 0.32 | 0.41 | -0.03 | 0.11 | -0.04 | 0.40 | -0.11 | 0.12 | 0.23 | 0.44 | 0.24 | 1.00 | -0.63 | 0.00 | 0.00 | 0.00 | 0.00 | 0.00 | 0.00 | 0.00 | 0.00 | 0.00 | 0.00 | 0.00 | 0.00 | 0.00 | 0.00 | 0.00 | 0.00 | 0.00 | 0.00 | 0.00 | 0.00 | 0.00 | 0.00 | 0.00 | 0.00 | 0.00 | 0.00 | 0.00 | 0.00 | 0.00 | 0.00 | 0.00 | 0.00 | 0.00 | 0.00 | 0.00 | 0.00 | 0.00 | 0.00 | 0.00 | 0.00 | 0.00 | 0.00 | 0.00 | 0.00 | 0.00 | 0.00 | 0.00 | 0.00 | -0.05 | 0.00 | 0.05 | 0.09 | 0.08 | 0.01 |
| vryrel | -0.04 | -0.02 | 0.08 | -0.06 | -0.01 | -0.01 | 0.02 | 0.35 | -0.08 | 0.05 | -0.11 | -0.01 | 0.06 | -0.05 | -0.02 | -0.06 | -0.05 | 0.01 | -0.04 | 0.11 | 0.05 | 0.12 | 0.30 | -0.08 | -0.24 | 0.13 | 0.05 | 0.38 | -0.02 | 0.09 | -0.10 | -0.17 | -0.20 | 0.06 | 0.44 | 0.38 | 0.30 | -0.42 | -0.13 | -0.41 | -0.29 | 0.00 | 0.24 | -0.28 | -0.13 | 0.18 | 0.15 | 0.04 | -0.23 | 0.25 | 0.01 | -0.13 | -0.36 | 0.00 | -0.63 | 1.00 | 0.00 | 0.00 | 0.00 | 0.00 | 0.00 | 0.00 | 0.00 | 0.00 | 0.00 | 0.00 | 0.00 | 0.00 | 0.00 | 0.00 | 0.00 | 0.00 | 0.00 | 0.00 | 0.00 | 0.00 | 0.00 | 0.00 | 0.00 | 0.00 | 0.00 | 0.00 | 0.00 | 0.00 | 0.00 | 0.00 | 0.00 | 0.00 | 0.00 | 0.00 | 0.00 | 0.00 | 0.00 | 0.00 | 0.00 | 0.00 | 0.00 | 0.00 | 0.00 | 0.00 | 0.00 | 0.00 | 0.00 | 0.00 | -0.01 | 0.00 | 0.00 | -0.06 | -0.05 | 0.01 |
| Zext | 0.84 | 0.51 | 0.62 | -0.35 | -0.46 | 0.76 | 0.48 | 0.30 | -0.47 | -0.44 | 0.84 | 0.44 | 0.44 | -0.23 | -0.47 | 0.78 | 0.49 | 0.54 | -0.34 | -0.50 | 0.70 | 0.42 | 0.54 | -0.29 | -0.30 | 0.17 | -0.07 | -0.14 | -0.26 | 0.13 | 0.28 | 0.29 | 0.16 | -0.05 | -0.11 | 0.16 | -0.11 | 0.16 | 0.25 | -0.02 | -0.10 | 0.00 | -0.20 | -0.18 | -0.12 | -0.05 | -0.23 | -0.25 | 0.00 | 0.00 | 0.00 | 0.00 | 0.00 | 0.00 | 0.00 | 0.00 | 1.00 | 0.59 | 0.77 | -0.53 | -0.67 | 0.91 | 0.55 | 0.42 | -0.64 | -0.65 | 0.95 | 0.52 | 0.56 | -0.35 | -0.65 | 0.98 | 0.56 | 0.71 | -0.53 | -0.68 | 0.78 | 0.49 | 0.61 | -0.39 | -0.52 | 0.19 | -0.09 | -0.17 | -0.30 | 0.16 | 0.40 | 0.33 | 0.23 | -0.06 | -0.18 | 0.24 | -0.17 | 0.37 | 0.36 | -0.06 | -0.16 | 0.00 | -0.26 | -0.27 | -0.10 | -0.37 | -0.20 | -0.26 | 0.21 | -0.53 | -0.40 | -0.03 | 0.04 | -0.06 |
| Zagr | 0.50 | 0.87 | 0.61 | -0.50 | -0.48 | 0.41 | 0.82 | 0.32 | -0.37 | -0.38 | 0.51 | 0.83 | 0.54 | -0.47 | -0.50 | 0.43 | 0.85 | 0.58 | -0.46 | -0.52 | 0.51 | 0.64 | 0.33 | -0.47 | -0.30 | 0.03 | -0.28 | -0.24 | 0.26 | 0.26 | 0.05 | 0.60 | 0.35 | 0.06 | -0.12 | 0.33 | 0.05 | 0.03 | 0.06 | -0.10 | -0.09 | 0.06 | -0.22 | -0.30 | -0.16 | -0.04 | -0.07 | 0.07 | 0.00 | 0.00 | 0.00 | 0.00 | 0.00 | 0.00 | 0.00 | 0.00 | 0.59 | 1.00 | 0.74 | -0.76 | -0.70 | 0.49 | 0.93 | 0.45 | -0.51 | -0.55 | 0.58 | 0.98 | 0.69 | -0.73 | -0.69 | 0.54 | 0.99 | 0.76 | -0.71 | -0.71 | 0.57 | 0.73 | 0.37 | -0.64 | -0.52 | 0.03 | -0.33 | -0.29 | 0.30 | 0.33 | 0.08 | 0.68 | 0.49 | 0.07 | -0.20 | 0.51 | 0.07 | 0.07 | 0.09 | -0.27 | -0.15 | 0.10 | -0.28 | -0.43 | -0.08 | -0.11 | -0.26 | 0.07 | 0.19 | -0.74 | -0.45 | -0.14 | 0.20 | -0.16 |
| Zcns | 0.64 | 0.64 | 0.81 | -0.50 | -0.46 | 0.57 | 0.58 | 0.49 | -0.55 | -0.39 | 0.64 | 0.61 | 0.69 | -0.39 | -0.48 | 0.61 | 0.60 | 0.75 | -0.50 | -0.51 | 0.48 | 0.61 | 0.59 | -0.35 | -0.30 | 0.12 | -0.23 | -0.18 | -0.11 | 0.21 | 0.28 | 0.28 | 0.34 | -0.17 | -0.12 | 0.28 | -0.03 | 0.12 | 0.25 | -0.10 | -0.16 | -0.07 | -0.15 | -0.30 | -0.20 | -0.11 | -0.27 | -0.17 | 0.00 | 0.00 | 0.00 | 0.00 | 0.00 | 0.00 | 0.00 | 0.00 | 0.77 | 0.74 | 1.00 | -0.75 | -0.67 | 0.69 | 0.66 | 0.70 | -0.76 | -0.57 | 0.73 | 0.72 | 0.87 | -0.61 | -0.66 | 0.77 | 0.69 | 0.98 | -0.78 | -0.69 | 0.54 | 0.70 | 0.66 | -0.48 | -0.51 | 0.13 | -0.28 | -0.22 | -0.13 | 0.27 | 0.40 | 0.32 | 0.47 | -0.20 | -0.20 | 0.42 | -0.05 | 0.27 | 0.36 | -0.27 | -0.26 | -0.11 | -0.19 | -0.43 | -0.20 | -0.44 | -0.34 | -0.18 | 0.21 | -0.72 | -0.54 | -0.22 | 0.00 | 0.00 |
| Zneu | -0.45 | -0.66 | -0.61 | 0.66 | 0.38 | -0.36 | -0.61 | -0.35 | 0.60 | 0.27 | -0.43 | -0.65 | -0.63 | 0.61 | 0.39 | -0.37 | -0.62 | -0.54 | 0.62 | 0.43 | -0.51 | -0.61 | -0.58 | 0.60 | 0.21 | -0.05 | 0.26 | 0.29 | -0.18 | -0.24 | 0.02 | -0.31 | -0.13 | -0.03 | 0.15 | -0.32 | -0.15 | 0.05 | 0.02 | 0.15 | 0.21 | 0.11 | 0.07 | 0.17 | 0.17 | 0.03 | 0.08 | -0.07 | 0.00 | 0.00 | 0.00 | 0.00 | 0.00 | 0.00 | 0.00 | 0.00 | -0.53 | -0.76 | -0.75 | 1.00 | 0.55 | -0.44 | -0.70 | -0.50 | 0.83 | 0.40 | -0.49 | -0.76 | -0.80 | 0.95 | 0.54 | -0.47 | -0.72 | -0.71 | 0.97 | 0.59 | -0.57 | -0.70 | -0.65 | 0.81 | 0.37 | -0.05 | 0.31 | 0.36 | -0.21 | -0.30 | 0.03 | -0.35 | -0.19 | -0.03 | 0.26 | -0.48 | -0.22 | 0.11 | 0.03 | 0.40 | 0.34 | 0.18 | 0.09 | 0.24 | 0.06 | 0.13 | 0.28 | -0.07 | 0.00 | 0.93 | 0.70 | 0.30 | 0.00 | 0.01 |
| Zopn | -0.56 | -0.60 | -0.55 | 0.36 | 0.69 | -0.49 | -0.52 | -0.18 | 0.33 | 0.57 | -0.58 | -0.58 | -0.48 | 0.31 | 0.70 | -0.52 | -0.60 | -0.52 | 0.34 | 0.71 | -0.50 | -0.40 | -0.31 | 0.34 | 0.49 | -0.07 | 0.29 | 0.34 | -0.05 | -0.32 | -0.17 | -0.46 | -0.31 | -0.03 | 0.07 | -0.35 | -0.03 | -0.04 | -0.16 | 0.13 | 0.17 | 0.01 | 0.23 | 0.34 | 0.11 | 0.04 | 0.11 | 0.13 | 0.00 | 0.00 | 0.00 | 0.00 | 0.00 | 0.00 | 0.00 | 0.00 | -0.67 | -0.70 | -0.67 | 0.55 | 1.00 | -0.59 | -0.59 | -0.26 | 0.46 | 0.83 | -0.66 | -0.69 | -0.61 | 0.48 | 0.98 | -0.66 | -0.70 | -0.69 | 0.52 | 0.97 | -0.55 | -0.46 | -0.34 | 0.46 | 0.84 | -0.07 | 0.34 | 0.42 | -0.06 | -0.41 | -0.24 | -0.53 | -0.44 | -0.04 | 0.11 | -0.53 | -0.04 | -0.08 | -0.22 | 0.36 | 0.28 | 0.02 | 0.30 | 0.49 | 0.07 | 0.18 | 0.19 | 0.14 | -0.04 | 0.54 | 0.38 | 0.22 | 0.04 | 0.12 |
| Zmext | 0.76 | 0.43 | 0.56 | -0.29 | -0.41 | 0.83 | 0.42 | 0.27 | -0.40 | -0.43 | 0.67 | 0.35 | 0.35 | -0.18 | -0.40 | 0.72 | 0.40 | 0.48 | -0.29 | -0.43 | 0.59 | 0.35 | 0.49 | -0.21 | -0.27 | 0.52 | 0.01 | -0.06 | -0.25 | 0.05 | 0.31 | 0.24 | 0.14 | -0.08 | -0.08 | 0.16 | -0.14 | 0.19 | 0.19 | 0.02 | -0.04 | 0.01 | -0.27 | -0.18 | -0.14 | -0.12 | -0.25 | -0.24 | 0.00 | 0.00 | 0.00 | 0.00 | 0.00 | 0.00 | 0.00 | 0.00 | 0.91 | 0.49 | 0.69 | -0.44 | -0.59 | 1.00 | 0.48 | 0.38 | -0.55 | -0.62 | 0.77 | 0.41 | 0.44 | -0.27 | -0.55 | 0.91 | 0.47 | 0.63 | -0.45 | -0.59 | 0.66 | 0.40 | 0.55 | -0.28 | -0.46 | 0.57 | 0.01 | -0.08 | -0.29 | 0.06 | 0.45 | 0.28 | 0.20 | -0.10 | -0.13 | 0.24 | -0.21 | 0.42 | 0.28 | 0.05 | -0.07 | 0.02 | -0.35 | -0.26 | -0.22 | -0.41 | -0.23 | -0.26 | 0.27 | -0.40 | -0.26 | 0.05 | 0.06 | -0.14 |
| Zmagr | 0.46 | 0.81 | 0.54 | -0.46 | -0.40 | 0.40 | 0.88 | 0.37 | -0.37 | -0.33 | 0.45 | 0.73 | 0.46 | -0.42 | -0.41 | 0.40 | 0.79 | 0.51 | -0.43 | -0.42 | 0.49 | 0.62 | 0.33 | -0.43 | -0.29 | 0.09 | 0.01 | -0.08 | 0.19 | 0.19 | 0.04 | 0.53 | 0.28 | 0.03 | -0.04 | 0.33 | 0.06 | 0.02 | 0.03 | -0.10 | -0.13 | 0.00 | -0.13 | -0.28 | -0.16 | -0.12 | -0.08 | 0.05 | 0.00 | 0.00 | 0.00 | 0.00 | 0.00 | 0.00 | 0.00 | 0.00 | 0.55 | 0.93 | 0.66 | -0.70 | -0.59 | 0.48 | 1.00 | 0.52 | -0.50 | -0.49 | 0.51 | 0.86 | 0.59 | -0.65 | -0.57 | 0.50 | 0.92 | 0.67 | -0.67 | -0.57 | 0.54 | 0.71 | 0.37 | -0.58 | -0.50 | 0.10 | 0.02 | -0.09 | 0.22 | 0.24 | 0.06 | 0.60 | 0.40 | 0.04 | -0.06 | 0.51 | 0.09 | 0.04 | 0.05 | -0.26 | -0.22 | 0.00 | -0.17 | -0.41 | -0.22 | -0.14 | -0.26 | 0.05 | 0.20 | -0.69 | -0.36 | -0.13 | 0.12 | -0.08 |
| Zmcns | 0.35 | 0.39 | 0.57 | -0.33 | -0.18 | 0.32 | 0.45 | 0.71 | -0.43 | -0.10 | 0.28 | 0.36 | 0.49 | -0.25 | -0.19 | 0.33 | 0.31 | 0.51 | -0.32 | -0.15 | 0.24 | 0.61 | 0.51 | -0.27 | -0.21 | 0.17 | 0.03 | 0.33 | -0.18 | 0.16 | 0.17 | -0.09 | 0.16 | -0.04 | 0.13 | 0.27 | 0.09 | 0.01 | 0.14 | -0.13 | -0.23 | -0.16 | 0.10 | -0.29 | -0.24 | -0.14 | -0.27 | -0.17 | 0.00 | 0.00 | 0.00 | 0.00 | 0.00 | 0.00 | 0.00 | 0.00 | 0.42 | 0.45 | 0.70 | -0.50 | -0.26 | 0.38 | 0.52 | 1.00 | -0.60 | -0.14 | 0.31 | 0.43 | 0.62 | -0.38 | -0.26 | 0.42 | 0.36 | 0.67 | -0.50 | -0.21 | 0.27 | 0.70 | 0.57 | -0.37 | -0.35 | 0.19 | 0.04 | 0.41 | -0.21 | 0.20 | 0.24 | -0.10 | 0.22 | -0.05 | 0.21 | 0.41 | 0.13 | 0.01 | 0.20 | -0.36 | -0.38 | -0.26 | 0.12 | -0.42 | -0.26 | -0.43 | -0.40 | -0.18 | 0.13 | -0.47 | -0.28 | -0.28 | -0.06 | 0.18 |
| Zmneu | -0.54 | -0.44 | -0.62 | 0.55 | 0.31 | -0.46 | -0.44 | -0.42 | 0.72 | 0.28 | -0.52 | -0.41 | -0.56 | 0.41 | 0.30 | -0.49 | -0.39 | -0.54 | 0.53 | 0.35 | -0.46 | -0.52 | -0.62 | 0.46 | 0.20 | -0.08 | 0.08 | 0.12 | 0.31 | -0.09 | -0.17 | -0.09 | -0.11 | 0.05 | 0.09 | -0.25 | -0.10 | 0.00 | -0.13 | 0.16 | 0.24 | 0.14 | 0.01 | 0.16 | 0.22 | 0.05 | 0.22 | 0.09 | 0.00 | 0.00 | 0.00 | 0.00 | 0.00 | 0.00 | 0.00 | 0.00 | -0.64 | -0.51 | -0.76 | 0.83 | 0.46 | -0.55 | -0.50 | -0.60 | 1.00 | 0.42 | -0.60 | -0.48 | -0.71 | 0.63 | 0.42 | -0.62 | -0.45 | -0.71 | 0.82 | 0.48 | -0.51 | -0.59 | -0.70 | 0.62 | 0.34 | -0.09 | 0.09 | 0.16 | 0.36 | -0.11 | -0.24 | -0.10 | -0.15 | 0.06 | 0.15 | -0.38 | -0.16 | 0.00 | -0.19 | 0.42 | 0.39 | 0.22 | 0.01 | 0.23 | 0.09 | 0.36 | 0.37 | 0.09 | 0.03 | 0.81 | 0.61 | 0.41 | 0.03 | -0.08 |
| Zmopn | -0.54 | -0.48 | -0.47 | 0.26 | 0.57 | -0.52 | -0.42 | -0.10 | 0.30 | 0.68 | -0.58 | -0.42 | -0.28 | 0.18 | 0.51 | -0.51 | -0.49 | -0.42 | 0.24 | 0.62 | -0.44 | -0.21 | -0.29 | 0.23 | 0.35 | -0.12 | 0.11 | 0.20 | 0.12 | 0.13 | -0.20 | -0.47 | -0.22 | 0.00 | 0.16 | -0.25 | 0.06 | -0.12 | -0.17 | 0.03 | 0.05 | -0.11 | 0.31 | 0.23 | 0.07 | -0.03 | 0.08 | 0.10 | 0.00 | 0.00 | 0.00 | 0.00 | 0.00 | 0.00 | 0.00 | 0.00 | -0.65 | -0.55 | -0.57 | 0.40 | 0.83 | -0.62 | -0.49 | -0.14 | 0.42 | 1.00 | -0.66 | -0.49 | -0.35 | 0.28 | 0.70 | -0.65 | -0.57 | -0.55 | 0.38 | 0.85 | -0.50 | -0.24 | -0.32 | 0.31 | 0.61 | -0.13 | 0.14 | 0.25 | 0.14 | 0.16 | -0.29 | -0.54 | -0.31 | 0.00 | 0.26 | -0.38 | 0.09 | -0.28 | -0.24 | 0.09 | 0.08 | -0.17 | 0.40 | 0.33 | -0.06 | 0.13 | 0.12 | 0.11 | -0.24 | 0.44 | 0.32 | 0.09 | -0.03 | 0.23 |
| Zfext | 0.80 | 0.50 | 0.60 | -0.32 | -0.45 | 0.64 | 0.44 | 0.22 | -0.43 | -0.45 | 0.88 | 0.43 | 0.40 | -0.20 | -0.46 | 0.74 | 0.49 | 0.52 | -0.32 | -0.50 | 0.68 | 0.37 | 0.49 | -0.25 | -0.27 | -0.08 | -0.12 | -0.18 | -0.25 | 0.10 | 0.25 | 0.33 | 0.18 | -0.08 | -0.16 | 0.12 | -0.13 | 0.18 | 0.31 | -0.02 | -0.08 | 0.03 | -0.20 | -0.17 | -0.09 | 0.00 | -0.18 | -0.24 | 0.00 | 0.00 | 0.00 | 0.00 | 0.00 | 0.00 | 0.00 | 0.00 | 0.95 | 0.58 | 0.73 | -0.49 | -0.66 | 0.77 | 0.51 | 0.31 | -0.60 | -0.66 | 1.00 | 0.51 | 0.50 | -0.31 | -0.63 | 0.93 | 0.56 | 0.68 | -0.50 | -0.69 | 0.76 | 0.43 | 0.54 | -0.33 | -0.46 | -0.09 | -0.14 | -0.23 | -0.30 | 0.13 | 0.36 | 0.38 | 0.26 | -0.10 | -0.26 | 0.19 | -0.19 | 0.41 | 0.44 | -0.06 | -0.14 | 0.06 | -0.26 | -0.25 | 0.00 | -0.30 | -0.15 | -0.26 | 0.23 | -0.53 | -0.44 | -0.07 | 0.04 | -0.07 |
| Zfagr | 0.44 | 0.85 | 0.58 | -0.50 | -0.47 | 0.34 | 0.75 | 0.30 | -0.35 | -0.33 | 0.45 | 0.85 | 0.58 | -0.49 | -0.50 | 0.38 | 0.83 | 0.56 | -0.45 | -0.51 | 0.47 | 0.65 | 0.32 | -0.51 | -0.30 | -0.01 | -0.42 | -0.30 | 0.32 | 0.32 | 0.03 | 0.56 | 0.35 | 0.12 | -0.13 | 0.32 | 0.07 | -0.01 | 0.02 | -0.11 | -0.09 | 0.06 | -0.21 | -0.28 | -0.15 | 0.00 | -0.04 | 0.11 | 0.00 | 0.00 | 0.00 | 0.00 | 0.00 | 0.00 | 0.00 | 0.00 | 0.52 | 0.98 | 0.72 | -0.76 | -0.69 | 0.41 | 0.86 | 0.43 | -0.48 | -0.49 | 0.51 | 1.00 | 0.73 | -0.76 | -0.70 | 0.47 | 0.96 | 0.74 | -0.70 | -0.70 | 0.52 | 0.74 | 0.36 | -0.69 | -0.51 | -0.02 | -0.50 | -0.37 | 0.37 | 0.41 | 0.04 | 0.64 | 0.48 | 0.15 | -0.21 | 0.49 | 0.11 | -0.02 | 0.03 | -0.29 | -0.14 | 0.10 | -0.27 | -0.41 | 0.00 | -0.07 | -0.24 | 0.12 | 0.12 | -0.74 | -0.46 | -0.14 | 0.21 | -0.16 |
| Zfcns | 0.47 | 0.60 | 0.71 | -0.53 | -0.42 | 0.37 | 0.51 | 0.44 | -0.52 | -0.24 | 0.44 | 0.62 | 0.79 | -0.48 | -0.46 | 0.43 | 0.55 | 0.66 | -0.51 | -0.45 | 0.38 | 0.61 | 0.54 | -0.45 | -0.28 | 0.03 | -0.37 | -0.37 | 0.07 | 0.38 | 0.16 | 0.23 | 0.29 | -0.04 | -0.09 | 0.24 | 0.07 | -0.02 | 0.07 | -0.13 | -0.18 | -0.16 | -0.03 | -0.22 | -0.16 | -0.07 | -0.20 | -0.02 | 0.00 | 0.00 | 0.00 | 0.00 | 0.00 | 0.00 | 0.00 | 0.00 | 0.56 | 0.69 | 0.87 | -0.80 | -0.61 | 0.44 | 0.59 | 0.62 | -0.71 | -0.35 | 0.50 | 0.73 | 1.00 | -0.74 | -0.64 | 0.54 | 0.64 | 0.87 | -0.80 | -0.62 | 0.43 | 0.70 | 0.61 | -0.61 | -0.48 | 0.04 | -0.44 | -0.46 | 0.09 | 0.48 | 0.23 | 0.26 | 0.40 | -0.05 | -0.14 | 0.37 | 0.10 | -0.05 | 0.11 | -0.34 | -0.30 | -0.27 | -0.04 | -0.32 | -0.12 | -0.32 | -0.27 | -0.02 | -0.07 | -0.72 | -0.54 | -0.18 | -0.06 | 0.09 |
| Zfneu | -0.29 | -0.63 | -0.49 | 0.63 | 0.33 | -0.23 | -0.57 | -0.27 | 0.46 | 0.19 | -0.27 | -0.65 | -0.58 | 0.64 | 0.35 | -0.21 | -0.60 | -0.44 | 0.58 | 0.38 | -0.44 | -0.57 | -0.49 | 0.60 | 0.17 | -0.02 | 0.32 | 0.35 | -0.41 | -0.29 | 0.13 | -0.32 | -0.09 | -0.08 | 0.16 | -0.29 | -0.17 | 0.09 | 0.13 | 0.13 | 0.17 | 0.11 | 0.06 | 0.12 | 0.10 | 0.01 | -0.02 | -0.16 | 0.00 | 0.00 | 0.00 | 0.00 | 0.00 | 0.00 | 0.00 | 0.00 | -0.35 | -0.73 | -0.61 | 0.95 | 0.48 | -0.27 | -0.65 | -0.38 | 0.63 | 0.28 | -0.31 | -0.76 | -0.74 | 1.00 | 0.49 | -0.27 | -0.70 | -0.57 | 0.90 | 0.52 | -0.49 | -0.66 | -0.55 | 0.82 | 0.30 | -0.02 | 0.38 | 0.43 | -0.49 | -0.36 | 0.18 | -0.36 | -0.13 | -0.10 | 0.26 | -0.45 | -0.26 | 0.21 | 0.18 | 0.34 | 0.28 | 0.18 | 0.08 | 0.17 | 0.02 | -0.04 | 0.17 | -0.17 | 0.03 | 0.85 | 0.66 | 0.21 | 0.01 | 0.04 |
| Zfopn | -0.54 | -0.60 | -0.54 | 0.36 | 0.67 | -0.46 | -0.50 | -0.18 | 0.31 | 0.48 | -0.55 | -0.59 | -0.50 | 0.31 | 0.72 | -0.50 | -0.60 | -0.53 | 0.34 | 0.69 | -0.48 | -0.43 | -0.27 | 0.32 | 0.49 | -0.05 | 0.33 | 0.36 | -0.10 | -0.46 | -0.16 | -0.43 | -0.34 | -0.01 | 0.04 | -0.35 | -0.03 | -0.03 | -0.17 | 0.16 | 0.19 | 0.04 | 0.21 | 0.35 | 0.12 | 0.07 | 0.12 | 0.16 | 0.00 | 0.00 | 0.00 | 0.00 | 0.00 | 0.00 | 0.00 | 0.00 | -0.65 | -0.69 | -0.66 | 0.54 | 0.98 | -0.55 | -0.57 | -0.26 | 0.42 | 0.70 | -0.63 | -0.70 | -0.64 | 0.49 | 1.00 | -0.63 | -0.69 | -0.69 | 0.52 | 0.94 | -0.54 | -0.50 | -0.30 | 0.44 | 0.85 | -0.05 | 0.39 | 0.44 | -0.11 | -0.59 | -0.23 | -0.49 | -0.48 | -0.01 | 0.06 | -0.54 | -0.05 | -0.06 | -0.24 | 0.41 | 0.32 | 0.06 | 0.27 | 0.51 | 0.13 | 0.20 | 0.20 | 0.17 | 0.00 | 0.52 | 0.37 | 0.25 | 0.04 | 0.10 |
| Ze\_LT30 | 0.82 | 0.47 | 0.62 | -0.31 | -0.45 | 0.76 | 0.44 | 0.30 | -0.45 | -0.44 | 0.82 | 0.40 | 0.42 | -0.17 | -0.45 | 0.79 | 0.44 | 0.55 | -0.31 | -0.48 | 0.58 | 0.41 | 0.50 | -0.21 | -0.30 | 0.20 | -0.06 | -0.12 | -0.32 | 0.11 | 0.40 | 0.25 | 0.20 | -0.13 | -0.09 | 0.14 | -0.13 | 0.19 | 0.30 | -0.02 | -0.11 | 0.00 | -0.18 | -0.20 | -0.15 | -0.08 | -0.29 | -0.28 | 0.00 | 0.00 | 0.00 | 0.00 | 0.00 | 0.00 | 0.00 | 0.00 | 0.98 | 0.54 | 0.77 | -0.47 | -0.66 | 0.91 | 0.50 | 0.42 | -0.62 | -0.65 | 0.93 | 0.47 | 0.54 | -0.27 | -0.63 | 1.00 | 0.51 | 0.72 | -0.49 | -0.66 | 0.65 | 0.47 | 0.56 | -0.28 | -0.52 | 0.22 | -0.07 | -0.15 | -0.37 | 0.14 | 0.57 | 0.28 | 0.28 | -0.15 | -0.15 | 0.22 | -0.20 | 0.42 | 0.43 | -0.06 | -0.18 | -0.01 | -0.23 | -0.29 | -0.15 | -0.47 | -0.24 | -0.30 | 0.23 | -0.47 | -0.35 | -0.05 | 0.00 | -0.01 |
| Za\_LT30 | 0.47 | 0.86 | 0.57 | -0.47 | -0.48 | 0.39 | 0.80 | 0.26 | -0.33 | -0.39 | 0.49 | 0.82 | 0.50 | -0.45 | -0.50 | 0.41 | 0.86 | 0.55 | -0.44 | -0.53 | 0.51 | 0.55 | 0.27 | -0.44 | -0.29 | 0.01 | -0.28 | -0.27 | 0.28 | 0.25 | 0.03 | 0.68 | 0.36 | 0.05 | -0.15 | 0.32 | 0.03 | 0.03 | 0.04 | -0.09 | -0.07 | 0.08 | -0.23 | -0.29 | -0.14 | -0.02 | -0.04 | 0.08 | 0.00 | 0.00 | 0.00 | 0.00 | 0.00 | 0.00 | 0.00 | 0.00 | 0.56 | 0.99 | 0.69 | -0.72 | -0.70 | 0.47 | 0.92 | 0.36 | -0.45 | -0.57 | 0.56 | 0.96 | 0.64 | -0.70 | -0.69 | 0.51 | 1.00 | 0.72 | -0.68 | -0.72 | 0.57 | 0.63 | 0.30 | -0.60 | -0.51 | 0.01 | -0.33 | -0.33 | 0.33 | 0.31 | 0.04 | 0.77 | 0.51 | 0.06 | -0.24 | 0.49 | 0.05 | 0.06 | 0.06 | -0.24 | -0.11 | 0.14 | -0.30 | -0.41 | -0.04 | -0.07 | -0.23 | 0.09 | 0.17 | -0.71 | -0.40 | -0.11 | 0.21 | -0.19 |
| Zc\_LT30 | 0.59 | 0.66 | 0.80 | -0.47 | -0.47 | 0.52 | 0.59 | 0.47 | -0.51 | -0.38 | 0.60 | 0.63 | 0.68 | -0.37 | -0.50 | 0.57 | 0.62 | 0.76 | -0.48 | -0.51 | 0.41 | 0.59 | 0.45 | -0.31 | -0.30 | 0.09 | -0.26 | -0.20 | -0.09 | 0.26 | 0.29 | 0.33 | 0.46 | -0.20 | -0.12 | 0.29 | -0.03 | 0.12 | 0.28 | -0.12 | -0.18 | -0.06 | -0.13 | -0.31 | -0.22 | -0.13 | -0.30 | -0.16 | 0.00 | 0.00 | 0.00 | 0.00 | 0.00 | 0.00 | 0.00 | 0.00 | 0.71 | 0.76 | 0.98 | -0.71 | -0.69 | 0.63 | 0.67 | 0.67 | -0.71 | -0.55 | 0.68 | 0.74 | 0.87 | -0.57 | -0.69 | 0.72 | 0.72 | 1.00 | -0.75 | -0.70 | 0.46 | 0.67 | 0.50 | -0.42 | -0.52 | 0.10 | -0.31 | -0.25 | -0.11 | 0.33 | 0.42 | 0.38 | 0.64 | -0.24 | -0.19 | 0.44 | -0.05 | 0.27 | 0.41 | -0.32 | -0.29 | -0.10 | -0.17 | -0.45 | -0.25 | -0.48 | -0.36 | -0.17 | 0.20 | -0.68 | -0.49 | -0.24 | 0.05 | -0.01 |
| Zn\_LT30 | -0.44 | -0.62 | -0.64 | 0.64 | 0.36 | -0.37 | -0.59 | -0.35 | 0.60 | 0.26 | -0.44 | -0.60 | -0.63 | 0.58 | 0.38 | -0.39 | -0.59 | -0.57 | 0.64 | 0.41 | -0.44 | -0.56 | -0.56 | 0.47 | 0.21 | -0.05 | 0.20 | 0.29 | -0.13 | -0.23 | -0.06 | -0.31 | -0.18 | 0.19 | 0.14 | -0.31 | -0.12 | 0.01 | -0.04 | 0.15 | 0.22 | 0.15 | 0.04 | 0.18 | 0.19 | 0.08 | 0.15 | 0.02 | 0.00 | 0.00 | 0.00 | 0.00 | 0.00 | 0.00 | 0.00 | 0.00 | -0.53 | -0.71 | -0.78 | 0.97 | 0.52 | -0.45 | -0.67 | -0.50 | 0.82 | 0.38 | -0.50 | -0.70 | -0.80 | 0.90 | 0.52 | -0.49 | -0.68 | -0.75 | 1.00 | 0.56 | -0.49 | -0.64 | -0.63 | 0.63 | 0.36 | -0.05 | 0.24 | 0.36 | -0.16 | -0.29 | -0.09 | -0.36 | -0.26 | 0.23 | 0.23 | -0.48 | -0.18 | 0.03 | -0.06 | 0.41 | 0.36 | 0.25 | 0.06 | 0.26 | 0.15 | 0.25 | 0.32 | 0.02 | -0.03 | 0.88 | 0.66 | 0.34 | 0.06 | -0.03 |
| Zo\_LT30 | -0.57 | -0.61 | -0.56 | 0.39 | 0.67 | -0.49 | -0.50 | -0.15 | 0.35 | 0.58 | -0.60 | -0.60 | -0.49 | 0.33 | 0.68 | -0.53 | -0.62 | -0.54 | 0.36 | 0.73 | -0.52 | -0.37 | -0.31 | 0.36 | 0.41 | -0.04 | 0.34 | 0.39 | -0.07 | -0.27 | -0.15 | -0.52 | -0.33 | -0.04 | 0.20 | -0.31 | -0.02 | -0.06 | -0.15 | 0.10 | 0.12 | -0.05 | 0.26 | 0.27 | 0.08 | 0.00 | 0.10 | 0.07 | 0.00 | 0.00 | 0.00 | 0.00 | 0.00 | 0.00 | 0.00 | 0.00 | -0.68 | -0.71 | -0.69 | 0.59 | 0.97 | -0.59 | -0.57 | -0.21 | 0.48 | 0.85 | -0.69 | -0.70 | -0.62 | 0.52 | 0.94 | -0.66 | -0.72 | -0.70 | 0.56 | 1.00 | -0.58 | -0.42 | -0.34 | 0.49 | 0.70 | -0.04 | 0.40 | 0.49 | -0.09 | -0.34 | -0.22 | -0.59 | -0.46 | -0.05 | 0.33 | -0.47 | -0.02 | -0.14 | -0.22 | 0.27 | 0.20 | -0.08 | 0.34 | 0.40 | 0.00 | 0.16 | 0.14 | 0.07 | -0.05 | 0.58 | 0.45 | 0.15 | -0.03 | 0.16 |
| Ze\_GT30 | 0.66 | 0.49 | 0.44 | -0.38 | -0.38 | 0.55 | 0.48 | 0.19 | -0.37 | -0.34 | 0.67 | 0.44 | 0.33 | -0.32 | -0.39 | 0.52 | 0.49 | 0.35 | -0.32 | -0.42 | 0.90 | 0.34 | 0.52 | -0.44 | -0.23 | 0.04 | -0.09 | -0.15 | 0.01 | 0.14 | -0.17 | 0.37 | -0.02 | 0.20 | -0.12 | 0.18 | -0.02 | 0.04 | 0.02 | -0.02 | -0.05 | 0.00 | -0.23 | -0.11 | -0.01 | 0.04 | 0.03 | -0.08 | 0.00 | 0.00 | 0.00 | 0.00 | 0.00 | 0.00 | 0.00 | 0.00 | 0.78 | 0.57 | 0.54 | -0.57 | -0.55 | 0.66 | 0.54 | 0.27 | -0.51 | -0.50 | 0.76 | 0.52 | 0.43 | -0.49 | -0.54 | 0.65 | 0.57 | 0.46 | -0.49 | -0.58 | 1.00 | 0.39 | 0.58 | -0.60 | -0.39 | 0.04 | -0.10 | -0.19 | 0.02 | 0.18 | -0.25 | 0.42 | -0.02 | 0.25 | -0.21 | 0.28 | -0.03 | 0.08 | 0.03 | -0.05 | -0.08 | 0.00 | -0.29 | -0.16 | 0.08 | 0.04 | -0.02 | -0.08 | 0.06 | -0.55 | -0.42 | 0.05 | 0.12 | -0.20 |
| Za\_GT30 | 0.41 | 0.64 | 0.57 | -0.46 | -0.32 | 0.33 | 0.63 | 0.49 | -0.43 | -0.17 | 0.37 | 0.63 | 0.55 | -0.42 | -0.36 | 0.37 | 0.55 | 0.51 | -0.41 | -0.31 | 0.35 | 0.87 | 0.50 | -0.49 | -0.28 | 0.07 | -0.20 | -0.01 | 0.10 | 0.32 | 0.12 | 0.00 | 0.17 | 0.14 | 0.06 | 0.32 | 0.15 | -0.01 | 0.10 | -0.14 | -0.24 | -0.09 | -0.04 | -0.28 | -0.20 | -0.15 | -0.16 | 0.01 | 0.00 | 0.00 | 0.00 | 0.00 | 0.00 | 0.00 | 0.00 | 0.00 | 0.49 | 0.73 | 0.70 | -0.70 | -0.46 | 0.40 | 0.71 | 0.70 | -0.59 | -0.24 | 0.43 | 0.74 | 0.70 | -0.66 | -0.50 | 0.47 | 0.63 | 0.67 | -0.64 | -0.42 | 0.39 | 1.00 | 0.56 | -0.66 | -0.48 | 0.08 | -0.24 | -0.01 | 0.12 | 0.41 | 0.17 | 0.00 | 0.24 | 0.17 | 0.10 | 0.49 | 0.23 | -0.03 | 0.15 | -0.38 | -0.39 | -0.15 | -0.05 | -0.40 | -0.28 | -0.26 | -0.34 | 0.01 | 0.15 | -0.68 | -0.49 | -0.29 | 0.02 | 0.03 |
| Zc\_GT30 | 0.51 | 0.32 | 0.54 | -0.43 | -0.24 | 0.46 | 0.33 | 0.40 | -0.51 | -0.22 | 0.48 | 0.30 | 0.48 | -0.35 | -0.22 | 0.44 | 0.26 | 0.38 | -0.40 | -0.25 | 0.52 | 0.49 | 0.89 | -0.41 | -0.16 | 0.14 | -0.06 | -0.05 | -0.11 | 0.04 | 0.06 | -0.06 | -0.24 | 0.04 | -0.04 | 0.17 | 0.02 | 0.01 | -0.01 | -0.05 | -0.11 | -0.17 | -0.07 | -0.16 | -0.09 | -0.01 | -0.08 | -0.11 | 0.00 | 0.00 | 0.00 | 0.00 | 0.00 | 0.00 | 0.00 | 0.00 | 0.61 | 0.37 | 0.66 | -0.65 | -0.34 | 0.55 | 0.37 | 0.57 | -0.70 | -0.32 | 0.54 | 0.36 | 0.61 | -0.55 | -0.30 | 0.56 | 0.30 | 0.50 | -0.63 | -0.34 | 0.58 | 0.56 | 1.00 | -0.55 | -0.28 | 0.15 | -0.07 | -0.06 | -0.13 | 0.05 | 0.08 | -0.06 | -0.34 | 0.05 | -0.06 | 0.26 | 0.04 | 0.01 | -0.02 | -0.12 | -0.18 | -0.28 | -0.09 | -0.23 | -0.01 | -0.13 | -0.15 | -0.12 | 0.02 | -0.62 | -0.51 | -0.13 | -0.26 | 0.07 |
| Zn\_GT30 | -0.32 | -0.56 | -0.39 | 0.54 | 0.31 | -0.24 | -0.50 | -0.26 | 0.45 | 0.21 | -0.29 | -0.58 | -0.48 | 0.53 | 0.31 | -0.22 | -0.52 | -0.32 | 0.41 | 0.36 | -0.53 | -0.58 | -0.49 | 0.74 | 0.17 | -0.01 | 0.31 | 0.23 | -0.24 | -0.20 | 0.20 | -0.21 | 0.03 | -0.50 | 0.15 | -0.24 | -0.19 | 0.14 | 0.18 | 0.10 | 0.12 | 0.01 | 0.09 | 0.09 | 0.06 | -0.08 | -0.11 | -0.23 | 0.00 | 0.00 | 0.00 | 0.00 | 0.00 | 0.00 | 0.00 | 0.00 | -0.39 | -0.64 | -0.48 | 0.81 | 0.46 | -0.28 | -0.58 | -0.37 | 0.62 | 0.31 | -0.33 | -0.69 | -0.61 | 0.82 | 0.44 | -0.28 | -0.60 | -0.42 | 0.63 | 0.49 | -0.60 | -0.66 | -0.55 | 1.00 | 0.29 | -0.02 | 0.37 | 0.29 | -0.28 | -0.25 | 0.29 | -0.23 | 0.04 | -0.61 | 0.24 | -0.36 | -0.28 | 0.30 | 0.26 | 0.25 | 0.20 | 0.02 | 0.12 | 0.13 | -0.14 | -0.18 | 0.10 | -0.25 | 0.10 | 0.78 | 0.60 | 0.13 | -0.10 | 0.09 |
| Zo\_GT30 | -0.43 | -0.45 | -0.42 | 0.24 | 0.58 | -0.39 | -0.44 | -0.25 | 0.25 | 0.42 | -0.40 | -0.43 | -0.38 | 0.19 | 0.61 | -0.41 | -0.44 | -0.40 | 0.23 | 0.51 | -0.35 | -0.42 | -0.25 | 0.21 | 0.58 | -0.12 | 0.13 | 0.13 | 0.02 | -0.38 | -0.16 | -0.23 | -0.23 | 0.01 | -0.27 | -0.38 | -0.04 | 0.04 | -0.14 | 0.20 | 0.28 | 0.17 | 0.10 | 0.43 | 0.17 | 0.13 | 0.13 | 0.29 | 0.00 | 0.00 | 0.00 | 0.00 | 0.00 | 0.00 | 0.00 | 0.00 | -0.52 | -0.52 | -0.51 | 0.37 | 0.84 | -0.46 | -0.50 | -0.35 | 0.34 | 0.61 | -0.46 | -0.51 | -0.48 | 0.30 | 0.85 | -0.52 | -0.51 | -0.52 | 0.36 | 0.70 | -0.39 | -0.48 | -0.28 | 0.29 | 1.00 | -0.13 | 0.15 | 0.16 | 0.02 | -0.48 | -0.24 | -0.26 | -0.32 | 0.01 | -0.44 | -0.58 | -0.07 | 0.09 | -0.20 | 0.53 | 0.46 | 0.27 | 0.12 | 0.62 | 0.23 | 0.21 | 0.28 | 0.30 | -0.01 | 0.35 | 0.16 | 0.36 | 0.20 | 0.02 |
| ZgenD\_E | 0.16 | 0.03 | 0.11 | -0.03 | -0.05 | 0.48 | 0.09 | 0.14 | -0.06 | -0.09 | -0.07 | -0.01 | 0.03 | -0.01 | -0.04 | 0.18 | 0.01 | 0.08 | -0.03 | -0.03 | 0.04 | 0.07 | 0.14 | -0.01 | -0.08 | 0.91 | 0.17 | 0.14 | -0.06 | -0.06 | 0.17 | -0.04 | -0.02 | -0.03 | 0.07 | 0.09 | -0.06 | 0.05 | -0.09 | 0.06 | 0.04 | -0.02 | -0.16 | -0.06 | -0.10 | -0.18 | -0.16 | -0.07 | 0.00 | 0.00 | 0.00 | 0.00 | 0.00 | 0.00 | 0.00 | 0.00 | 0.19 | 0.03 | 0.13 | -0.05 | -0.07 | 0.57 | 0.10 | 0.19 | -0.09 | -0.13 | -0.09 | -0.02 | 0.04 | -0.02 | -0.05 | 0.22 | 0.01 | 0.10 | -0.05 | -0.04 | 0.04 | 0.08 | 0.15 | -0.02 | -0.13 | 1.00 | 0.20 | 0.17 | -0.07 | -0.07 | 0.24 | -0.05 | -0.02 | -0.03 | 0.12 | 0.13 | -0.09 | 0.12 | -0.13 | 0.16 | 0.06 | -0.04 | -0.21 | -0.08 | -0.34 | -0.25 | -0.17 | -0.07 | 0.13 | 0.05 | 0.16 | 0.16 | 0.05 | -0.13 |
| ZgenD\_A | -0.07 | -0.29 | -0.23 | 0.20 | 0.24 | 0.01 | 0.01 | 0.03 | 0.07 | 0.09 | -0.12 | -0.43 | -0.35 | 0.24 | 0.28 | -0.06 | -0.28 | -0.24 | 0.15 | 0.29 | -0.09 | -0.21 | -0.06 | 0.27 | 0.09 | 0.18 | 0.84 | 0.45 | -0.30 | -0.30 | 0.02 | -0.20 | -0.19 | -0.18 | 0.19 | -0.06 | -0.04 | 0.05 | 0.01 | 0.05 | -0.06 | -0.12 | 0.19 | 0.08 | 0.02 | -0.19 | -0.06 | -0.14 | 0.00 | 0.00 | 0.00 | 0.00 | 0.00 | 0.00 | 0.00 | 0.00 | -0.09 | -0.33 | -0.28 | 0.31 | 0.34 | 0.01 | 0.02 | 0.04 | 0.09 | 0.14 | -0.14 | -0.50 | -0.44 | 0.38 | 0.39 | -0.07 | -0.33 | -0.31 | 0.24 | 0.40 | -0.10 | -0.24 | -0.07 | 0.37 | 0.15 | 0.20 | 1.00 | 0.56 | -0.35 | -0.38 | 0.02 | -0.23 | -0.27 | -0.22 | 0.31 | -0.09 | -0.05 | 0.11 | 0.02 | 0.13 | -0.09 | -0.20 | 0.25 | 0.11 | -0.36 | -0.09 | 0.03 | -0.15 | 0.11 | 0.28 | 0.28 | 0.05 | -0.21 | 0.18 |
| ZgenD\_C | -0.14 | -0.25 | -0.18 | 0.24 | 0.29 | -0.06 | -0.08 | 0.29 | 0.11 | 0.17 | -0.20 | -0.31 | -0.36 | 0.28 | 0.32 | -0.12 | -0.29 | -0.19 | 0.23 | 0.36 | -0.17 | -0.01 | -0.05 | 0.21 | 0.09 | 0.16 | 0.47 | 0.81 | -0.29 | -0.26 | 0.01 | -0.37 | -0.16 | 0.00 | 0.24 | 0.03 | 0.02 | 0.03 | 0.07 | 0.00 | -0.05 | 0.01 | 0.14 | -0.07 | -0.08 | -0.08 | -0.07 | -0.17 | 0.00 | 0.00 | 0.00 | 0.00 | 0.00 | 0.00 | 0.00 | 0.00 | -0.17 | -0.29 | -0.22 | 0.36 | 0.42 | -0.08 | -0.09 | 0.41 | 0.16 | 0.25 | -0.23 | -0.37 | -0.46 | 0.43 | 0.44 | -0.15 | -0.33 | -0.25 | 0.36 | 0.49 | -0.19 | -0.01 | -0.06 | 0.29 | 0.16 | 0.17 | 0.56 | 1.00 | -0.34 | -0.33 | 0.01 | -0.42 | -0.22 | 0.00 | 0.40 | 0.04 | 0.03 | 0.07 | 0.11 | -0.01 | -0.08 | 0.02 | 0.18 | -0.11 | -0.16 | -0.12 | -0.13 | -0.18 | 0.22 | 0.30 | 0.31 | -0.11 | 0.00 | 0.09 |
| ZgenD\_N | -0.25 | 0.26 | -0.10 | -0.14 | -0.04 | -0.24 | 0.19 | -0.15 | 0.26 | 0.09 | -0.26 | 0.32 | 0.07 | -0.32 | -0.08 | -0.30 | 0.28 | -0.08 | -0.10 | -0.06 | 0.02 | 0.11 | -0.11 | -0.21 | 0.01 | -0.07 | -0.30 | -0.28 | 0.85 | 0.25 | -0.34 | 0.28 | 0.00 | 0.16 | -0.08 | 0.08 | 0.09 | -0.11 | -0.30 | 0.02 | 0.07 | 0.02 | -0.07 | 0.04 | 0.13 | 0.04 | 0.27 | 0.30 | 0.00 | 0.00 | 0.00 | 0.00 | 0.00 | 0.00 | 0.00 | 0.00 | -0.30 | 0.30 | -0.13 | -0.21 | -0.06 | -0.29 | 0.22 | -0.21 | 0.36 | 0.14 | -0.30 | 0.37 | 0.09 | -0.49 | -0.11 | -0.37 | 0.33 | -0.11 | -0.16 | -0.09 | 0.02 | 0.12 | -0.13 | -0.28 | 0.02 | -0.07 | -0.35 | -0.34 | 1.00 | 0.31 | -0.49 | 0.32 | -0.01 | 0.19 | -0.14 | 0.12 | 0.13 | -0.25 | -0.43 | 0.06 | 0.11 | 0.04 | -0.09 | 0.05 | 0.08 | 0.45 | 0.21 | 0.31 | -0.01 | -0.11 | -0.11 | 0.21 | 0.03 | -0.14 |
| ZgenD\_O | 0.13 | 0.29 | 0.22 | -0.20 | -0.28 | 0.05 | 0.21 | 0.14 | -0.08 | 0.11 | 0.11 | 0.35 | 0.38 | -0.23 | -0.42 | 0.11 | 0.27 | 0.25 | -0.19 | -0.25 | 0.16 | 0.36 | 0.04 | -0.19 | -0.28 | -0.06 | -0.32 | -0.27 | 0.27 | 0.79 | -0.01 | 0.06 | 0.22 | 0.01 | 0.12 | 0.20 | 0.12 | -0.11 | 0.04 | -0.18 | -0.21 | -0.17 | 0.06 | -0.23 | -0.09 | -0.13 | -0.08 | -0.10 | 0.00 | 0.00 | 0.00 | 0.00 | 0.00 | 0.00 | 0.00 | 0.00 | 0.16 | 0.33 | 0.27 | -0.30 | -0.41 | 0.06 | 0.24 | 0.20 | -0.11 | 0.16 | 0.13 | 0.41 | 0.48 | -0.36 | -0.59 | 0.14 | 0.31 | 0.33 | -0.29 | -0.34 | 0.18 | 0.41 | 0.05 | -0.25 | -0.48 | -0.07 | -0.38 | -0.33 | 0.31 | 1.00 | -0.01 | 0.07 | 0.31 | 0.01 | 0.20 | 0.31 | 0.17 | -0.24 | 0.06 | -0.47 | -0.35 | -0.28 | 0.07 | -0.34 | -0.25 | -0.13 | -0.14 | -0.11 | -0.28 | -0.22 | -0.15 | -0.24 | -0.09 | 0.12 |
| ZageD\_E | 0.34 | 0.07 | 0.32 | 0.02 | -0.16 | 0.38 | 0.05 | 0.17 | -0.17 | -0.19 | 0.32 | 0.03 | 0.18 | 0.12 | -0.16 | 0.45 | 0.03 | 0.32 | -0.06 | -0.16 | -0.23 | 0.15 | 0.07 | 0.22 | -0.14 | 0.22 | 0.02 | 0.01 | -0.42 | -0.01 | 0.70 | -0.08 | 0.27 | -0.38 | 0.02 | -0.02 | -0.15 | 0.20 | 0.35 | -0.01 | -0.09 | 0.00 | 0.01 | -0.14 | -0.17 | -0.15 | -0.39 | -0.28 | 0.00 | 0.00 | 0.00 | 0.00 | 0.00 | 0.00 | 0.00 | 0.00 | 0.40 | 0.08 | 0.40 | 0.03 | -0.24 | 0.45 | 0.06 | 0.24 | -0.24 | -0.29 | 0.36 | 0.04 | 0.23 | 0.18 | -0.23 | 0.57 | 0.04 | 0.42 | -0.09 | -0.22 | -0.25 | 0.17 | 0.08 | 0.29 | -0.24 | 0.24 | 0.02 | 0.01 | -0.49 | -0.01 | 1.00 | -0.09 | 0.39 | -0.46 | 0.03 | -0.03 | -0.22 | 0.45 | 0.51 | -0.03 | -0.15 | -0.01 | 0.02 | -0.21 | -0.28 | -0.64 | -0.28 | -0.30 | 0.23 | 0.00 | 0.00 | -0.13 | -0.12 | 0.20 |
| ZageD\_A | 0.28 | 0.59 | 0.26 | -0.23 | -0.36 | 0.23 | 0.53 | -0.07 | -0.07 | -0.37 | 0.33 | 0.54 | 0.20 | -0.23 | -0.35 | 0.22 | 0.67 | 0.29 | -0.23 | -0.43 | 0.37 | 0.00 | -0.06 | -0.17 | -0.15 | -0.05 | -0.19 | -0.34 | 0.27 | 0.06 | -0.06 | 0.88 | 0.33 | -0.06 | -0.24 | 0.15 | -0.08 | 0.05 | -0.03 | 0.00 | 0.11 | 0.18 | -0.27 | -0.14 | -0.01 | 0.09 | 0.07 | 0.10 | 0.00 | 0.00 | 0.00 | 0.00 | 0.00 | 0.00 | 0.00 | 0.00 | 0.33 | 0.68 | 0.32 | -0.35 | -0.53 | 0.28 | 0.60 | -0.10 | -0.10 | -0.54 | 0.38 | 0.64 | 0.26 | -0.36 | -0.49 | 0.28 | 0.77 | 0.38 | -0.36 | -0.59 | 0.42 | 0.00 | -0.06 | -0.23 | -0.26 | -0.05 | -0.23 | -0.42 | 0.32 | 0.07 | -0.09 | 1.00 | 0.47 | -0.07 | -0.39 | 0.22 | -0.13 | 0.11 | -0.05 | 0.00 | 0.18 | 0.30 | -0.35 | -0.20 | 0.17 | 0.12 | -0.01 | 0.10 | 0.10 | -0.36 | -0.12 | 0.10 | 0.25 | -0.26 |
| ZageD\_C | 0.19 | 0.43 | 0.38 | -0.12 | -0.30 | 0.16 | 0.35 | 0.15 | -0.11 | -0.21 | 0.23 | 0.41 | 0.32 | -0.09 | -0.35 | 0.23 | 0.44 | 0.49 | -0.17 | -0.33 | -0.02 | 0.21 | -0.31 | 0.03 | -0.19 | -0.02 | -0.23 | -0.18 | -0.01 | 0.25 | 0.27 | 0.41 | 0.71 | -0.25 | -0.09 | 0.16 | -0.06 | 0.13 | 0.32 | -0.09 | -0.10 | 0.09 | -0.08 | -0.20 | -0.16 | -0.14 | -0.25 | -0.07 | 0.00 | 0.00 | 0.00 | 0.00 | 0.00 | 0.00 | 0.00 | 0.00 | 0.23 | 0.49 | 0.47 | -0.19 | -0.44 | 0.20 | 0.40 | 0.22 | -0.15 | -0.31 | 0.26 | 0.48 | 0.40 | -0.13 | -0.48 | 0.28 | 0.51 | 0.64 | -0.26 | -0.46 | -0.02 | 0.24 | -0.34 | 0.04 | -0.32 | -0.02 | -0.27 | -0.22 | -0.01 | 0.31 | 0.39 | 0.47 | 1.00 | -0.31 | -0.15 | 0.25 | -0.08 | 0.29 | 0.46 | -0.24 | -0.16 | 0.14 | -0.11 | -0.29 | -0.26 | -0.41 | -0.26 | -0.08 | 0.20 | -0.19 | -0.08 | -0.15 | 0.29 | -0.07 |
| ZageD\_N | -0.05 | 0.06 | -0.16 | -0.02 | -0.03 | -0.08 | 0.03 | -0.03 | 0.05 | 0.00 | -0.08 | 0.12 | -0.04 | -0.06 | -0.01 | -0.12 | 0.05 | -0.19 | 0.15 | -0.03 | 0.22 | 0.15 | 0.05 | -0.45 | 0.01 | -0.03 | -0.19 | 0.00 | 0.16 | 0.01 | -0.32 | -0.06 | -0.22 | 0.82 | -0.04 | -0.02 | 0.11 | -0.16 | -0.28 | 0.04 | 0.08 | 0.14 | -0.07 | 0.07 | 0.12 | 0.18 | 0.30 | 0.31 | 0.00 | 0.00 | 0.00 | 0.00 | 0.00 | 0.00 | 0.00 | 0.00 | -0.06 | 0.07 | -0.20 | -0.03 | -0.04 | -0.10 | 0.04 | -0.05 | 0.06 | 0.00 | -0.10 | 0.15 | -0.05 | -0.10 | -0.01 | -0.15 | 0.06 | -0.24 | 0.23 | -0.05 | 0.25 | 0.17 | 0.05 | -0.61 | 0.01 | -0.03 | -0.22 | 0.00 | 0.19 | 0.01 | -0.46 | -0.07 | -0.31 | 1.00 | -0.07 | -0.04 | 0.17 | -0.35 | -0.40 | 0.10 | 0.13 | 0.23 | -0.09 | 0.10 | 0.33 | 0.48 | 0.21 | 0.33 | -0.16 | -0.08 | -0.08 | 0.19 | 0.18 | -0.14 |
| ZageD\_O | -0.15 | -0.17 | -0.16 | 0.17 | 0.08 | -0.11 | -0.05 | 0.15 | 0.11 | 0.18 | -0.23 | -0.18 | -0.11 | 0.17 | 0.05 | -0.12 | -0.21 | -0.15 | 0.14 | 0.24 | -0.18 | 0.09 | -0.06 | 0.18 | -0.25 | 0.11 | 0.26 | 0.32 | -0.12 | 0.16 | 0.02 | -0.35 | -0.11 | -0.06 | 0.60 | 0.11 | 0.04 | -0.13 | -0.01 | -0.14 | -0.22 | -0.28 | 0.21 | -0.22 | -0.11 | -0.16 | -0.05 | -0.30 | 0.00 | 0.00 | 0.00 | 0.00 | 0.00 | 0.00 | 0.00 | 0.00 | -0.18 | -0.20 | -0.20 | 0.26 | 0.11 | -0.13 | -0.06 | 0.21 | 0.15 | 0.26 | -0.26 | -0.21 | -0.14 | 0.26 | 0.06 | -0.15 | -0.24 | -0.19 | 0.23 | 0.33 | -0.21 | 0.10 | -0.06 | 0.24 | -0.44 | 0.12 | 0.31 | 0.40 | -0.14 | 0.20 | 0.03 | -0.39 | -0.15 | -0.07 | 1.00 | 0.17 | 0.06 | -0.29 | -0.02 | -0.36 | -0.37 | -0.45 | 0.27 | -0.32 | -0.31 | -0.08 | -0.19 | -0.31 | -0.06 | 0.27 | 0.36 | -0.28 | -0.30 | 0.17 |
| Ztfr | 0.20 | 0.44 | 0.35 | -0.32 | -0.36 | 0.20 | 0.45 | 0.29 | -0.27 | -0.26 | 0.17 | 0.42 | 0.29 | -0.29 | -0.39 | 0.17 | 0.42 | 0.33 | -0.31 | -0.35 | 0.25 | 0.43 | 0.23 | -0.27 | -0.34 | 0.12 | -0.08 | 0.03 | 0.10 | 0.24 | -0.02 | 0.20 | 0.17 | -0.03 | 0.10 | 0.65 | 0.16 | -0.08 | 0.13 | -0.28 | -0.40 | -0.13 | 0.05 | -0.48 | -0.12 | -0.21 | -0.11 | -0.14 | 0.00 | 0.00 | 0.00 | 0.00 | 0.00 | 0.00 | 0.00 | 0.00 | 0.24 | 0.51 | 0.42 | -0.48 | -0.53 | 0.24 | 0.51 | 0.41 | -0.38 | -0.38 | 0.19 | 0.49 | 0.37 | -0.45 | -0.54 | 0.22 | 0.49 | 0.44 | -0.48 | -0.47 | 0.28 | 0.49 | 0.26 | -0.36 | -0.58 | 0.13 | -0.09 | 0.04 | 0.12 | 0.31 | -0.03 | 0.22 | 0.25 | -0.04 | 0.17 | 1.00 | 0.25 | -0.18 | 0.19 | -0.74 | -0.66 | -0.21 | 0.07 | -0.70 | -0.39 | -0.18 | -0.20 | -0.15 | -0.02 | -0.49 | -0.29 | -0.62 | -0.11 | 0.15 |
| Zalpha | -0.14 | 0.06 | -0.04 | -0.15 | -0.03 | -0.18 | 0.08 | 0.09 | -0.11 | 0.06 | -0.17 | 0.09 | 0.08 | -0.16 | -0.04 | -0.16 | 0.04 | -0.04 | -0.11 | -0.02 | -0.03 | 0.20 | 0.03 | -0.21 | -0.04 | -0.08 | -0.05 | 0.03 | 0.11 | 0.14 | -0.16 | -0.11 | -0.06 | 0.14 | 0.03 | 0.16 | 0.67 | -0.29 | -0.22 | -0.10 | -0.17 | -0.13 | 0.29 | -0.01 | -0.10 | 0.00 | -0.03 | -0.03 | 0.00 | 0.00 | 0.00 | 0.00 | 0.00 | 0.00 | 0.00 | 0.00 | -0.17 | 0.07 | -0.05 | -0.22 | -0.04 | -0.21 | 0.09 | 0.13 | -0.16 | 0.09 | -0.19 | 0.11 | 0.10 | -0.26 | -0.05 | -0.20 | 0.05 | -0.05 | -0.18 | -0.02 | -0.03 | 0.23 | 0.04 | -0.28 | -0.07 | -0.09 | -0.05 | 0.03 | 0.13 | 0.17 | -0.22 | -0.13 | -0.08 | 0.17 | 0.06 | 0.25 | 1.00 | -0.66 | -0.32 | -0.26 | -0.29 | -0.21 | 0.38 | -0.01 | 0.00 | -0.05 | -0.17 | -0.03 | -0.59 | -0.25 | -0.25 | -0.30 | -0.21 | 0.31 |
| Zpeak | 0.31 | 0.06 | 0.22 | 0.08 | -0.06 | 0.35 | 0.04 | 0.01 | 0.00 | -0.19 | 0.36 | -0.01 | -0.04 | 0.13 | -0.04 | 0.33 | 0.06 | 0.21 | 0.02 | -0.10 | 0.07 | -0.02 | 0.01 | 0.22 | 0.05 | 0.11 | 0.09 | 0.06 | -0.21 | -0.19 | 0.31 | 0.09 | 0.20 | -0.29 | -0.18 | -0.12 | -0.44 | 0.45 | 0.48 | 0.15 | 0.19 | 0.25 | -0.38 | 0.04 | 0.05 | -0.05 | -0.16 | -0.21 | 0.00 | 0.00 | 0.00 | 0.00 | 0.00 | 0.00 | 0.00 | 0.00 | 0.37 | 0.07 | 0.27 | 0.11 | -0.08 | 0.42 | 0.04 | 0.01 | 0.00 | -0.28 | 0.41 | -0.02 | -0.05 | 0.21 | -0.06 | 0.42 | 0.06 | 0.27 | 0.03 | -0.14 | 0.08 | -0.03 | 0.01 | 0.30 | 0.09 | 0.12 | 0.11 | 0.07 | -0.25 | -0.24 | 0.45 | 0.11 | 0.29 | -0.35 | -0.29 | -0.18 | -0.66 | 1.00 | 0.69 | 0.39 | 0.32 | 0.40 | -0.49 | 0.06 | -0.09 | -0.27 | 0.08 | -0.22 | 0.75 | 0.15 | 0.07 | 0.25 | 0.29 | -0.36 |
| Zstop | 0.30 | 0.07 | 0.29 | 0.02 | -0.15 | 0.23 | 0.04 | 0.14 | -0.14 | -0.16 | 0.39 | 0.03 | 0.08 | 0.12 | -0.17 | 0.34 | 0.05 | 0.31 | -0.04 | -0.16 | 0.03 | 0.13 | -0.01 | 0.19 | -0.11 | -0.12 | 0.01 | 0.09 | -0.37 | 0.05 | 0.35 | -0.04 | 0.32 | -0.33 | -0.01 | 0.12 | -0.21 | 0.31 | 0.69 | -0.08 | -0.15 | 0.03 | -0.04 | -0.22 | -0.15 | -0.12 | -0.31 | -0.35 | 0.00 | 0.00 | 0.00 | 0.00 | 0.00 | 0.00 | 0.00 | 0.00 | 0.36 | 0.09 | 0.36 | 0.03 | -0.22 | 0.28 | 0.05 | 0.20 | -0.19 | -0.24 | 0.44 | 0.03 | 0.11 | 0.18 | -0.24 | 0.43 | 0.06 | 0.41 | -0.06 | -0.22 | 0.03 | 0.15 | -0.02 | 0.26 | -0.20 | -0.13 | 0.02 | 0.11 | -0.43 | 0.06 | 0.51 | -0.05 | 0.46 | -0.40 | -0.02 | 0.19 | -0.32 | 0.69 | 1.00 | -0.21 | -0.25 | 0.04 | -0.05 | -0.32 | -0.23 | -0.51 | -0.25 | -0.37 | 0.49 | 0.01 | 0.00 | -0.23 | 0.08 | 0.02 |
| Zafb | -0.05 | -0.23 | -0.22 | 0.26 | 0.25 | 0.04 | -0.23 | -0.25 | 0.30 | 0.06 | -0.06 | -0.25 | -0.27 | 0.22 | 0.30 | -0.05 | -0.21 | -0.24 | 0.27 | 0.20 | -0.04 | -0.33 | -0.11 | 0.19 | 0.31 | 0.14 | 0.11 | -0.01 | 0.05 | -0.37 | -0.02 | 0.00 | -0.17 | 0.09 | -0.22 | -0.49 | -0.18 | 0.17 | -0.14 | 0.37 | 0.45 | 0.20 | -0.26 | 0.40 | 0.24 | 0.11 | 0.10 | 0.14 | 0.00 | 0.00 | 0.00 | 0.00 | 0.00 | 0.00 | 0.00 | 0.00 | -0.06 | -0.27 | -0.27 | 0.40 | 0.36 | 0.05 | -0.26 | -0.36 | 0.42 | 0.09 | -0.06 | -0.29 | -0.34 | 0.34 | 0.41 | -0.06 | -0.24 | -0.32 | 0.41 | 0.27 | -0.05 | -0.38 | -0.12 | 0.25 | 0.53 | 0.16 | 0.13 | -0.01 | 0.06 | -0.47 | -0.03 | 0.00 | -0.24 | 0.10 | -0.36 | -0.74 | -0.26 | 0.39 | -0.21 | 1.00 | 0.74 | 0.33 | -0.34 | 0.57 | 0.20 | 0.16 | 0.39 | 0.14 | 0.23 | 0.42 | 0.24 | 0.84 | 0.15 | -0.31 |
| Zafm | -0.14 | -0.13 | -0.21 | 0.22 | 0.19 | -0.06 | -0.19 | -0.27 | 0.28 | 0.05 | -0.12 | -0.12 | -0.24 | 0.18 | 0.23 | -0.15 | -0.09 | -0.22 | 0.23 | 0.14 | -0.07 | -0.34 | -0.16 | 0.15 | 0.27 | 0.06 | -0.08 | -0.06 | 0.09 | -0.28 | -0.10 | 0.16 | -0.11 | 0.11 | -0.22 | -0.43 | -0.19 | 0.14 | -0.17 | 0.28 | 0.61 | 0.37 | -0.44 | 0.35 | 0.18 | 0.28 | 0.24 | 0.11 | 0.00 | 0.00 | 0.00 | 0.00 | 0.00 | 0.00 | 0.00 | 0.00 | -0.16 | -0.15 | -0.26 | 0.34 | 0.28 | -0.07 | -0.22 | -0.38 | 0.39 | 0.08 | -0.14 | -0.14 | -0.30 | 0.28 | 0.32 | -0.18 | -0.11 | -0.29 | 0.36 | 0.20 | -0.08 | -0.39 | -0.18 | 0.20 | 0.46 | 0.06 | -0.09 | -0.08 | 0.11 | -0.35 | -0.15 | 0.18 | -0.16 | 0.13 | -0.37 | -0.66 | -0.29 | 0.32 | -0.25 | 0.74 | 1.00 | 0.61 | -0.56 | 0.50 | 0.53 | 0.39 | 0.31 | 0.12 | 0.16 | 0.37 | 0.28 | 0.62 | 0.40 | -0.55 |
| Znvm | 0.00 | 0.09 | -0.09 | 0.12 | 0.01 | 0.02 | 0.00 | -0.18 | 0.16 | -0.12 | 0.05 | 0.09 | -0.21 | 0.12 | 0.04 | -0.01 | 0.12 | -0.07 | 0.16 | -0.06 | 0.00 | -0.13 | -0.25 | 0.02 | 0.16 | -0.03 | -0.17 | 0.02 | 0.03 | -0.22 | 0.00 | 0.26 | 0.10 | 0.19 | -0.27 | -0.14 | -0.14 | 0.18 | 0.03 | 0.12 | 0.37 | 0.61 | -0.41 | 0.19 | 0.12 | 0.26 | 0.17 | 0.16 | 0.00 | 0.00 | 0.00 | 0.00 | 0.00 | 0.00 | 0.00 | 0.00 | 0.00 | 0.10 | -0.11 | 0.18 | 0.02 | 0.02 | 0.00 | -0.26 | 0.22 | -0.17 | 0.06 | 0.10 | -0.27 | 0.18 | 0.06 | -0.01 | 0.14 | -0.10 | 0.25 | -0.08 | 0.00 | -0.15 | -0.28 | 0.02 | 0.27 | -0.04 | -0.20 | 0.02 | 0.04 | -0.28 | -0.01 | 0.30 | 0.14 | 0.23 | -0.45 | -0.21 | -0.21 | 0.40 | 0.04 | 0.33 | 0.61 | 1.00 | -0.53 | 0.28 | 0.49 | 0.27 | 0.20 | 0.17 | 0.30 | 0.14 | 0.04 | 0.13 | 0.74 | -0.47 |
| Zdiv | -0.22 | -0.24 | -0.15 | 0.06 | 0.21 | -0.29 | -0.14 | 0.09 | 0.01 | 0.27 | -0.23 | -0.23 | -0.03 | 0.05 | 0.19 | -0.19 | -0.26 | -0.13 | 0.04 | 0.25 | -0.26 | -0.04 | -0.08 | 0.09 | 0.07 | -0.19 | 0.21 | 0.15 | -0.07 | 0.06 | 0.01 | -0.31 | -0.08 | -0.08 | 0.16 | 0.04 | 0.25 | -0.22 | -0.03 | -0.13 | -0.34 | -0.32 | 0.77 | 0.01 | -0.10 | -0.09 | -0.16 | 0.05 | 0.00 | 0.00 | 0.00 | 0.00 | 0.00 | 0.00 | 0.00 | 0.00 | -0.26 | -0.28 | -0.19 | 0.09 | 0.30 | -0.35 | -0.17 | 0.12 | 0.01 | 0.40 | -0.26 | -0.27 | -0.04 | 0.08 | 0.27 | -0.23 | -0.30 | -0.17 | 0.06 | 0.34 | -0.29 | -0.05 | -0.09 | 0.12 | 0.12 | -0.21 | 0.25 | 0.18 | -0.09 | 0.07 | 0.02 | -0.35 | -0.11 | -0.09 | 0.27 | 0.07 | 0.38 | -0.49 | -0.05 | -0.34 | -0.56 | -0.53 | 1.00 | 0.01 | -0.18 | -0.26 | -0.17 | 0.05 | -0.37 | 0.01 | -0.04 | -0.29 | -0.41 | 0.91 |
| Zcoh | -0.22 | -0.37 | -0.35 | 0.16 | 0.33 | -0.21 | -0.36 | -0.30 | 0.17 | 0.22 | -0.22 | -0.35 | -0.25 | 0.11 | 0.37 | -0.23 | -0.36 | -0.35 | 0.17 | 0.29 | -0.14 | -0.35 | -0.21 | 0.09 | 0.36 | -0.08 | 0.09 | -0.09 | 0.05 | -0.26 | -0.14 | -0.18 | -0.20 | 0.09 | -0.20 | -0.46 | -0.01 | 0.03 | -0.22 | 0.21 | 0.31 | 0.17 | 0.01 | 0.69 | 0.24 | 0.20 | 0.13 | 0.31 | 0.00 | 0.00 | 0.00 | 0.00 | 0.00 | 0.00 | 0.00 | 0.00 | -0.27 | -0.43 | -0.43 | 0.24 | 0.49 | -0.26 | -0.41 | -0.42 | 0.23 | 0.33 | -0.25 | -0.41 | -0.32 | 0.17 | 0.51 | -0.29 | -0.41 | -0.45 | 0.26 | 0.40 | -0.16 | -0.40 | -0.23 | 0.13 | 0.62 | -0.08 | 0.11 | -0.11 | 0.05 | -0.34 | -0.21 | -0.20 | -0.29 | 0.10 | -0.32 | -0.70 | -0.01 | 0.06 | -0.32 | 0.57 | 0.50 | 0.28 | 0.01 | 1.00 | 0.38 | 0.21 | 0.40 | 0.32 | -0.09 | 0.31 | 0.07 | 0.42 | 0.23 | -0.07 |
| Znmf | -0.08 | -0.07 | -0.16 | 0.04 | 0.05 | -0.18 | -0.19 | -0.19 | 0.06 | -0.04 | 0.00 | 0.00 | -0.10 | 0.01 | 0.09 | -0.12 | -0.04 | -0.19 | 0.09 | 0.00 | 0.07 | -0.25 | -0.01 | -0.11 | 0.14 | -0.31 | -0.31 | -0.13 | 0.06 | -0.19 | -0.19 | 0.15 | -0.18 | 0.27 | -0.19 | -0.25 | 0.00 | -0.04 | -0.16 | 0.07 | 0.32 | 0.30 | -0.14 | 0.26 | 0.10 | 0.54 | 0.29 | 0.10 | 0.00 | 0.00 | 0.00 | 0.00 | 0.00 | 0.00 | 0.00 | 0.00 | -0.10 | -0.08 | -0.20 | 0.06 | 0.07 | -0.22 | -0.22 | -0.26 | 0.09 | -0.06 | 0.00 | 0.00 | -0.12 | 0.02 | 0.13 | -0.15 | -0.04 | -0.25 | 0.15 | 0.00 | 0.08 | -0.28 | -0.01 | -0.14 | 0.23 | -0.34 | -0.36 | -0.16 | 0.08 | -0.25 | -0.28 | 0.17 | -0.26 | 0.33 | -0.31 | -0.39 | 0.00 | -0.09 | -0.23 | 0.20 | 0.53 | 0.49 | -0.18 | 0.38 | 1.00 | 0.48 | 0.16 | 0.11 | -0.12 | 0.00 | -0.15 | 0.11 | 0.29 | -0.22 |
| Zuni | -0.31 | -0.10 | -0.36 | 0.09 | 0.12 | -0.34 | -0.12 | -0.31 | 0.26 | 0.09 | -0.26 | -0.06 | -0.25 | -0.02 | 0.14 | -0.37 | -0.06 | -0.37 | 0.16 | 0.11 | 0.04 | -0.22 | -0.11 | -0.13 | 0.12 | -0.23 | -0.08 | -0.09 | 0.38 | -0.10 | -0.45 | 0.11 | -0.29 | 0.40 | -0.05 | -0.12 | -0.03 | -0.12 | -0.36 | 0.06 | 0.24 | 0.17 | -0.20 | 0.14 | 0.30 | 0.26 | 0.61 | 0.26 | 0.00 | 0.00 | 0.00 | 0.00 | 0.00 | 0.00 | 0.00 | 0.00 | -0.37 | -0.11 | -0.44 | 0.13 | 0.18 | -0.41 | -0.14 | -0.43 | 0.36 | 0.13 | -0.30 | -0.07 | -0.32 | -0.04 | 0.20 | -0.47 | -0.07 | -0.48 | 0.25 | 0.16 | 0.04 | -0.26 | -0.13 | -0.18 | 0.21 | -0.25 | -0.09 | -0.12 | 0.45 | -0.13 | -0.64 | 0.12 | -0.41 | 0.48 | -0.08 | -0.18 | -0.05 | -0.27 | -0.51 | 0.16 | 0.39 | 0.27 | -0.26 | 0.21 | 0.48 | 1.00 | 0.50 | 0.27 | -0.13 | 0.12 | 0.04 | 0.19 | 0.12 | -0.32 |
| Zabr | -0.17 | -0.23 | -0.27 | 0.19 | 0.13 | -0.20 | -0.23 | -0.28 | 0.27 | 0.08 | -0.13 | -0.21 | -0.21 | 0.11 | 0.14 | -0.19 | -0.20 | -0.28 | 0.21 | 0.10 | -0.02 | -0.30 | -0.13 | 0.08 | 0.16 | -0.16 | 0.03 | -0.11 | 0.18 | -0.11 | -0.20 | -0.01 | -0.19 | 0.17 | -0.12 | -0.13 | -0.11 | 0.04 | -0.17 | 0.15 | 0.19 | 0.12 | -0.14 | 0.28 | 0.60 | 0.09 | 0.31 | 0.11 | 0.00 | 0.00 | 0.00 | 0.00 | 0.00 | 0.00 | 0.00 | 0.00 | -0.20 | -0.26 | -0.34 | 0.28 | 0.19 | -0.23 | -0.26 | -0.40 | 0.37 | 0.12 | -0.15 | -0.24 | -0.27 | 0.17 | 0.20 | -0.24 | -0.23 | -0.36 | 0.32 | 0.14 | -0.02 | -0.34 | -0.15 | 0.10 | 0.28 | -0.17 | 0.03 | -0.13 | 0.21 | -0.14 | -0.28 | -0.01 | -0.26 | 0.21 | -0.19 | -0.20 | -0.17 | 0.08 | -0.25 | 0.39 | 0.31 | 0.20 | -0.17 | 0.40 | 0.16 | 0.50 | 1.00 | 0.12 | -0.07 | 0.36 | 0.09 | 0.29 | 0.00 | -0.10 |
| Zfmp | -0.22 | 0.06 | -0.15 | -0.05 | 0.10 | -0.22 | 0.04 | -0.13 | 0.07 | 0.07 | -0.23 | 0.10 | -0.02 | -0.11 | 0.12 | -0.24 | 0.08 | -0.13 | 0.01 | 0.05 | -0.07 | 0.01 | -0.11 | -0.18 | 0.18 | -0.07 | -0.12 | -0.14 | 0.27 | -0.09 | -0.21 | 0.09 | -0.05 | 0.27 | -0.19 | -0.10 | -0.02 | -0.10 | -0.25 | 0.05 | 0.07 | 0.10 | 0.04 | 0.22 | 0.07 | 0.06 | 0.17 | 0.94 | 0.00 | 0.00 | 0.00 | 0.00 | 0.00 | 0.00 | 0.00 | 0.00 | -0.26 | 0.07 | -0.18 | -0.07 | 0.14 | -0.26 | 0.05 | -0.18 | 0.09 | 0.11 | -0.26 | 0.12 | -0.02 | -0.17 | 0.17 | -0.30 | 0.09 | -0.17 | 0.02 | 0.07 | -0.08 | 0.01 | -0.12 | -0.25 | 0.30 | -0.07 | -0.15 | -0.18 | 0.31 | -0.11 | -0.30 | 0.10 | -0.08 | 0.33 | -0.31 | -0.15 | -0.03 | -0.22 | -0.37 | 0.14 | 0.12 | 0.17 | 0.05 | 0.32 | 0.11 | 0.27 | 0.12 | 1.00 | -0.03 | -0.04 | 0.05 | 0.21 | 0.31 | -0.03 |
| ZSpeak | 0.22 | 0.20 | 0.21 | 0.02 | -0.07 | 0.27 | 0.21 | 0.09 | 0.03 | -0.22 | 0.25 | 0.13 | -0.03 | 0.05 | -0.04 | 0.24 | 0.19 | 0.20 | -0.01 | -0.09 | 0.05 | 0.15 | 0.01 | 0.11 | -0.03 | 0.14 | 0.09 | 0.16 | -0.04 | -0.23 | 0.23 | 0.12 | 0.19 | -0.16 | -0.06 | -0.04 | -0.39 | 0.37 | 0.41 | 0.10 | 0.09 | 0.15 | -0.30 | -0.08 | -0.08 | -0.08 | -0.12 | -0.03 | -0.01 | 0.01 | -0.09 | 0.03 | 0.01 | -0.04 | -0.05 | -0.01 | 0.21 | 0.19 | 0.21 | 0.00 | -0.04 | 0.27 | 0.20 | 0.13 | 0.03 | -0.24 | 0.23 | 0.12 | -0.07 | 0.03 | 0.00 | 0.23 | 0.17 | 0.20 | -0.03 | -0.05 | 0.06 | 0.15 | 0.02 | 0.10 | -0.01 | 0.13 | 0.11 | 0.22 | -0.01 | -0.28 | 0.23 | 0.10 | 0.20 | -0.16 | -0.06 | -0.02 | -0.59 | 0.75 | 0.49 | 0.23 | 0.16 | 0.30 | -0.37 | -0.09 | -0.12 | -0.13 | -0.07 | -0.03 | 1.00 | 0.01 | 0.04 | 0.14 | 0.36 | -0.39 |
| ZSneu | -0.44 | -0.64 | -0.59 | 0.61 | 0.37 | -0.33 | -0.60 | -0.33 | 0.59 | 0.30 | -0.46 | -0.62 | -0.57 | 0.55 | 0.37 | -0.37 | -0.61 | -0.52 | 0.57 | 0.42 | -0.50 | -0.59 | -0.55 | 0.58 | 0.20 | 0.05 | 0.23 | 0.24 | -0.09 | -0.17 | 0.00 | -0.31 | -0.14 | -0.06 | 0.17 | -0.32 | -0.16 | 0.07 | 0.01 | 0.16 | 0.23 | 0.09 | 0.01 | 0.21 | 0.21 | 0.00 | 0.07 | -0.04 | 0.00 | 0.00 | 0.00 | 0.00 | 0.00 | 0.00 | 0.00 | 0.00 | -0.53 | -0.74 | -0.72 | 0.93 | 0.54 | -0.40 | -0.69 | -0.47 | 0.81 | 0.44 | -0.53 | -0.74 | -0.72 | 0.85 | 0.52 | -0.47 | -0.71 | -0.68 | 0.88 | 0.58 | -0.55 | -0.68 | -0.62 | 0.78 | 0.35 | 0.05 | 0.28 | 0.30 | -0.11 | -0.22 | 0.00 | -0.36 | -0.19 | -0.08 | 0.27 | -0.49 | -0.25 | 0.15 | 0.01 | 0.42 | 0.37 | 0.14 | 0.01 | 0.31 | 0.00 | 0.12 | 0.36 | -0.04 | 0.01 | 1.00 | 0.82 | 0.38 | 0.04 | -0.05 |
| ZSfneu | -0.31 | -0.39 | -0.48 | 0.59 | 0.26 | -0.20 | -0.30 | -0.27 | 0.56 | 0.19 | -0.36 | -0.41 | -0.47 | 0.55 | 0.28 | -0.26 | -0.35 | -0.41 | 0.56 | 0.31 | -0.35 | -0.44 | -0.48 | 0.54 | 0.14 | 0.14 | 0.29 | 0.20 | -0.13 | -0.18 | 0.01 | -0.10 | -0.07 | -0.01 | 0.13 | -0.28 | -0.20 | 0.11 | 0.06 | 0.18 | 0.25 | 0.05 | -0.09 | 0.04 | 0.09 | -0.07 | 0.01 | 0.06 | 0.05 | -0.02 | -0.05 | 0.10 | 0.04 | -0.02 | 0.05 | 0.00 | -0.40 | -0.45 | -0.54 | 0.70 | 0.38 | -0.26 | -0.36 | -0.28 | 0.61 | 0.32 | -0.44 | -0.46 | -0.54 | 0.66 | 0.37 | -0.35 | -0.40 | -0.49 | 0.66 | 0.45 | -0.42 | -0.49 | -0.51 | 0.60 | 0.16 | 0.16 | 0.28 | 0.31 | -0.11 | -0.15 | 0.00 | -0.12 | -0.08 | -0.08 | 0.36 | -0.29 | -0.25 | 0.07 | 0.00 | 0.24 | 0.28 | 0.04 | -0.04 | 0.07 | -0.15 | 0.04 | 0.09 | 0.05 | 0.04 | 0.82 | 1.00 | 0.35 | 0.11 | -0.11 |
| ZSafb | 0.02 | -0.08 | -0.18 | 0.31 | 0.11 | 0.08 | -0.06 | -0.25 | 0.40 | -0.01 | -0.01 | -0.09 | -0.14 | 0.24 | 0.16 | 0.01 | -0.05 | -0.17 | 0.33 | 0.05 | 0.05 | -0.22 | -0.13 | 0.17 | 0.22 | 0.14 | 0.07 | -0.15 | 0.14 | -0.25 | -0.04 | 0.12 | -0.08 | 0.20 | -0.26 | -0.53 | -0.25 | 0.21 | -0.08 | 0.41 | 0.47 | 0.11 | -0.28 | 0.28 | 0.20 | 0.09 | 0.13 | 0.20 | 0.03 | 0.03 | -0.12 | 0.13 | 0.04 | -0.04 | 0.09 | -0.06 | -0.03 | -0.14 | -0.22 | 0.30 | 0.22 | 0.05 | -0.13 | -0.28 | 0.41 | 0.09 | -0.07 | -0.14 | -0.18 | 0.21 | 0.25 | -0.05 | -0.11 | -0.24 | 0.34 | 0.15 | 0.05 | -0.29 | -0.13 | 0.13 | 0.36 | 0.16 | 0.05 | -0.11 | 0.21 | -0.24 | -0.13 | 0.10 | -0.15 | 0.19 | -0.28 | -0.62 | -0.30 | 0.25 | -0.23 | 0.84 | 0.62 | 0.13 | -0.29 | 0.42 | 0.11 | 0.19 | 0.29 | 0.21 | 0.14 | 0.38 | 0.35 | 1.00 | 0.11 | -0.30 |
| ZSnvm | -0.02 | 0.14 | -0.04 | -0.04 | 0.09 | 0.01 | 0.07 | -0.12 | 0.00 | 0.01 | 0.00 | 0.14 | -0.09 | -0.04 | 0.10 | -0.05 | 0.15 | -0.01 | 0.00 | 0.02 | 0.07 | -0.01 | -0.25 | -0.12 | 0.21 | 0.03 | -0.16 | -0.05 | 0.07 | -0.14 | -0.12 | 0.20 | 0.17 | 0.16 | -0.27 | -0.05 | -0.18 | 0.21 | 0.03 | 0.15 | 0.26 | 0.46 | -0.38 | 0.28 | 0.02 | 0.03 | -0.02 | 0.26 | 0.10 | -0.14 | 0.04 | -0.09 | 0.10 | 0.05 | 0.08 | -0.05 | 0.04 | 0.20 | 0.00 | 0.00 | 0.04 | 0.06 | 0.12 | -0.06 | 0.03 | -0.03 | 0.04 | 0.21 | -0.06 | 0.01 | 0.04 | 0.00 | 0.21 | 0.05 | 0.06 | -0.03 | 0.12 | 0.02 | -0.26 | -0.10 | 0.20 | 0.05 | -0.21 | 0.00 | 0.03 | -0.09 | -0.12 | 0.25 | 0.29 | 0.18 | -0.30 | -0.11 | -0.21 | 0.29 | 0.08 | 0.15 | 0.40 | 0.74 | -0.41 | 0.23 | 0.29 | 0.12 | 0.00 | 0.31 | 0.36 | 0.04 | 0.11 | 0.11 | 1.00 | -0.46 |
| ZSdiv | -0.06 | -0.18 | -0.03 | 0.01 | 0.14 | -0.12 | -0.09 | 0.12 | -0.04 | 0.22 | -0.07 | -0.18 | 0.06 | 0.03 | 0.12 | -0.03 | -0.20 | -0.03 | -0.01 | 0.17 | -0.14 | -0.02 | 0.06 | 0.06 | 0.06 | -0.11 | 0.20 | 0.09 | -0.09 | 0.11 | 0.09 | -0.24 | -0.08 | -0.10 | 0.10 | 0.10 | 0.18 | -0.16 | -0.04 | -0.11 | -0.31 | -0.25 | 0.70 | -0.04 | -0.01 | -0.12 | -0.18 | -0.03 | 0.01 | -0.02 | 0.11 | 0.01 | 0.01 | 0.08 | 0.01 | 0.01 | -0.06 | -0.16 | 0.00 | 0.01 | 0.12 | -0.14 | -0.08 | 0.18 | -0.08 | 0.23 | -0.07 | -0.16 | 0.09 | 0.04 | 0.10 | -0.01 | -0.19 | -0.01 | -0.03 | 0.16 | -0.20 | 0.03 | 0.07 | 0.09 | 0.02 | -0.13 | 0.18 | 0.09 | -0.14 | 0.12 | 0.20 | -0.26 | -0.07 | -0.14 | 0.17 | 0.15 | 0.31 | -0.36 | 0.02 | -0.31 | -0.55 | -0.47 | 0.91 | -0.07 | -0.22 | -0.32 | -0.10 | -0.03 | -0.39 | -0.05 | -0.11 | -0.30 | -0.46 | 1.00 |

# 6 Results

In this section, we provide results for all analyses included in the manuscript.

## 6.1 Correlations with state-level personality

We estimate the correlation between each personality dimension and the fertility outcomes.

### 6.1.1 Total Fertility Rate

```
cor.test(map.join$Ztfr, map.join$Zext)
```

```
## 
##  Pearson's product-moment correlation
## 
## data:  map.join$Ztfr and map.join$Zext
## t = 1.7247, df = 48, p-value = 0.09101
## alternative hypothesis: true correlation is not equal to 0
## 95 percent confidence interval:
##  -0.03943144  0.48715889
## sample estimates:
##       cor 
## 0.2415676
```

```
cor.test(map.join$Ztfr, map.join$Zagr)
```

```
## 
##  Pearson's product-moment correlation
## 
## data:  map.join$Ztfr and map.join$Zagr
## t = 4.1122, df = 48, p-value = 0.0001525
## alternative hypothesis: true correlation is not equal to 0
## 95 percent confidence interval:
##  0.2704946 0.6906396
## sample estimates:
##       cor 
## 0.5104126
```

```
cor.test(map.join$Ztfr, map.join$Zcns)
```

```
## 
##  Pearson's product-moment correlation
## 
## data:  map.join$Ztfr and map.join$Zcns
## t = 3.2464, df = 48, p-value = 0.002134
## alternative hypothesis: true correlation is not equal to 0
## 95 percent confidence interval:
##  0.1655069 0.6284340
## sample estimates:
##       cor 
## 0.4243074
```

```
cor.test(map.join$Ztfr, map.join$ZSneu)
```

```
## 
##  Pearson's product-moment correlation
## 
## data:  map.join$Ztfr and map.join$ZSneu
## t = -3.9305, df = 48, p-value = 0.0002714
## alternative hypothesis: true correlation is not equal to 0
## 95 percent confidence interval:
##  -0.6785851 -0.2493377
## sample estimates:
##        cor 
## -0.4934395
```

```
cor.test(map.join$Ztfr, map.join$Zopn)
```

```
## 
##  Pearson's product-moment correlation
## 
## data:  map.join$Ztfr and map.join$Zopn
## t = -4.3346, df = 48, p-value = 7.438e-05
## alternative hypothesis: true correlation is not equal to 0
## 95 percent confidence interval:
##  -0.7047008 -0.2956969
## sample estimates:
##        cor 
## -0.5303901
```

### 6.1.2 Initiation

```
cor.test(map.join$Zalpha, map.join$Zext)
```

```
## 
##  Pearson's product-moment correlation
## 
## data:  map.join$Zalpha and map.join$Zext
## t = -1.1679, df = 48, p-value = 0.2486
## alternative hypothesis: true correlation is not equal to 0
## 95 percent confidence interval:
##  -0.4249139  0.1175606
## sample estimates:
##        cor 
## -0.1662265
```

```
cor.test(map.join$Zalpha, map.join$Zagr)
```

```
## 
##  Pearson's product-moment correlation
## 
## data:  map.join$Zalpha and map.join$Zagr
## t = 0.50071, df = 48, p-value = 0.6189
## alternative hypothesis: true correlation is not equal to 0
## 95 percent confidence interval:
##  -0.2104872  0.3435386
## sample estimates:
##        cor 
## 0.07208381
```

```
cor.test(map.join$Zalpha, map.join$Zcns)
```

```
## 
##  Pearson's product-moment correlation
## 
## data:  map.join$Zalpha and map.join$Zcns
## t = -0.3429, df = 48, p-value = 0.7332
## alternative hypothesis: true correlation is not equal to 0
## 95 percent confidence interval:
##  -0.3233320  0.2321082
## sample estimates:
##        cor 
## -0.0494332
```

```
cor.test(map.join$Zalpha, map.join$ZSneu)
```

```
## 
##  Pearson's product-moment correlation
## 
## data:  map.join$Zalpha and map.join$ZSneu
## t = -1.7511, df = 48, p-value = 0.08632
## alternative hypothesis: true correlation is not equal to 0
## 95 percent confidence interval:
##  -0.48996992  0.03574427
## sample estimates:
##        cor 
## -0.2450414
```

```
cor.test(map.join$Zalpha, map.join$Zopn)
```

```
## 
##  Pearson's product-moment correlation
## 
## data:  map.join$Zalpha and map.join$Zopn
## t = -0.30083, df = 48, p-value = 0.7648
## alternative hypothesis: true correlation is not equal to 0
## 95 percent confidence interval:
##  -0.3178894  0.2378394
## sample estimates:
##         cor 
## -0.04338013
```

### 6.1.3 Peak

```
cor.test(map.join$ZSpeak, map.join$Zext)
```

```
## 
##  Pearson's product-moment correlation
## 
## data:  map.join$ZSpeak and map.join$Zext
## t = 1.4573, df = 48, p-value = 0.1515
## alternative hypothesis: true correlation is not equal to 0
## 95 percent confidence interval:
##  -0.07690927  0.45795365
## sample estimates:
##       cor 
## 0.2058451
```

```
cor.test(map.join$ZSpeak, map.join$Zagr)
```

```
## 
##  Pearson's product-moment correlation
## 
## data:  map.join$ZSpeak and map.join$Zagr
## t = 1.3611, df = 48, p-value = 0.1798
## alternative hypothesis: true correlation is not equal to 0
## 95 percent confidence interval:
##  -0.09042637  0.44712909
## sample estimates:
##       cor 
## 0.1927734
```

```
cor.test(map.join$ZSpeak, map.join$Zcns)
```

```
## 
##  Pearson's product-moment correlation
## 
## data:  map.join$ZSpeak and map.join$Zcns
## t = 1.4758, df = 48, p-value = 0.1465
## alternative hypothesis: true correlation is not equal to 0
## 95 percent confidence interval:
##  -0.07432325  0.46000663
## sample estimates:
##       cor 
## 0.2083344
```

```
cor.test(map.join$ZSpeak, map.join$ZSneu)
```

```
## 
##  Pearson's product-moment correlation
## 
## data:  map.join$ZSpeak and map.join$ZSneu
## t = 0.070882, df = 48, p-value = 0.9438
## alternative hypothesis: true correlation is not equal to 0
## 95 percent confidence interval:
##  -0.2688830  0.2877587
## sample estimates:
##        cor 
## 0.01023041
```

```
cor.test(map.join$ZSpeak, map.join$Zopn)
```

```
## 
##  Pearson's product-moment correlation
## 
## data:  map.join$ZSpeak and map.join$Zopn
## t = -0.29447, df = 48, p-value = 0.7697
## alternative hypothesis: true correlation is not equal to 0
## 95 percent confidence interval:
##  -0.3170647  0.2387045
## sample estimates:
##         cor 
## -0.04246466
```

### 6.1.4 Stopping

```
cor.test(map.join$Zstop, map.join$Zext)
```

```
## 
##  Pearson's product-moment correlation
## 
## data:  map.join$Zstop and map.join$Zext
## t = 2.6649, df = 48, p-value = 0.01046
## alternative hypothesis: true correlation is not equal to 0
## 95 percent confidence interval:
##  0.08961594 0.57945198
## sample estimates:
##       cor 
## 0.3590084
```

```
cor.test(map.join$Zstop, map.join$Zagr)
```

```
## 
##  Pearson's product-moment correlation
## 
## data:  map.join$Zstop and map.join$Zagr
## t = 0.59195, df = 48, p-value = 0.5567
## alternative hypothesis: true correlation is not equal to 0
## 95 percent confidence interval:
##  -0.1979069  0.3550645
## sample estimates:
##        cor 
## 0.08513038
```

```
cor.test(map.join$Zstop, map.join$Zcns)
```

```
## 
##  Pearson's product-moment correlation
## 
## data:  map.join$Zstop and map.join$Zcns
## t = 2.6669, df = 48, p-value = 0.0104
## alternative hypothesis: true correlation is not equal to 0
## 95 percent confidence interval:
##  0.0898734 0.5796244
## sample estimates:
##       cor 
## 0.3592345
```

```
cor.test(map.join$Zstop, map.join$ZSneu)
```

```
## 
##  Pearson's product-moment correlation
## 
## data:  map.join$Zstop and map.join$ZSneu
## t = 0.098801, df = 48, p-value = 0.9217
## alternative hypothesis: true correlation is not equal to 0
## 95 percent confidence interval:
##  -0.2651409  0.2914501
## sample estimates:
##        cor 
## 0.01425919
```

```
cor.test(map.join$Zstop, map.join$Zopn)
```

```
## 
##  Pearson's product-moment correlation
## 
## data:  map.join$Zstop and map.join$Zopn
## t = -1.5919, df = 48, p-value = 0.118
## alternative hypothesis: true correlation is not equal to 0
## 95 percent confidence interval:
##  -0.47280930  0.05803268
## sample estimates:
##        cor 
## -0.2239323
```

### 6.1.5 Age at first birth

```
cor.test(map.join$ZSafb, map.join$Zext)
```

```
## 
##  Pearson's product-moment correlation
## 
## data:  map.join$ZSafb and map.join$Zext
## t = -0.19985, df = 48, p-value = 0.8424
## alternative hypothesis: true correlation is not equal to 0
## 95 percent confidence interval:
##  -0.3047354  0.2515330
## sample estimates:
##         cor 
## -0.02883345
```

```
cor.test(map.join$ZSafb, map.join$Zagr)
```

```
## 
##  Pearson's product-moment correlation
## 
## data:  map.join$ZSafb and map.join$Zagr
## t = -1.0041, df = 48, p-value = 0.3204
## alternative hypothesis: true correlation is not equal to 0
## 95 percent confidence interval:
##  -0.4055846  0.1405290
## sample estimates:
##        cor 
## -0.1434291
```

```
cor.test(map.join$ZSafb, map.join$Zcns)
```

```
## 
##  Pearson's product-moment correlation
## 
## data:  map.join$ZSafb and map.join$Zcns
## t = -1.5979, df = 48, p-value = 0.1166
## alternative hypothesis: true correlation is not equal to 0
## 95 percent confidence interval:
##  -0.47346550  0.05719002
## sample estimates:
##        cor 
## -0.2247352
```

```
cor.test(map.join$ZSafb, map.join$ZSneu)
```

```
## 
##  Pearson's product-moment correlation
## 
## data:  map.join$ZSafb and map.join$ZSneu
## t = 2.8801, df = 48, p-value = 0.005924
## alternative hypothesis: true correlation is not equal to 0
## 95 percent confidence interval:
##  0.1181375 0.5982852
## sample estimates:
##       cor 
## 0.3838626
```

```
cor.test(map.join$ZSafb, map.join$Zopn)
```

```
## 
##  Pearson's product-moment correlation
## 
## data:  map.join$ZSafb and map.join$Zopn
## t = 1.5281, df = 48, p-value = 0.1331
## alternative hypothesis: true correlation is not equal to 0
## 95 percent confidence interval:
##  -0.06697955  0.46580564
## sample estimates:
##       cor 
## 0.2153837
```

### 6.1.6 Age at first marriage

```
cor.test(map.join$Zafm, map.join$Zext)
```

```
## 
##  Pearson's product-moment correlation
## 
## data:  map.join$Zafm and map.join$Zext
## t = -1.1402, df = 48, p-value = 0.2599
## alternative hypothesis: true correlation is not equal to 0
## 95 percent confidence interval:
##  -0.4216732  0.1214524
## sample estimates:
##        cor 
## -0.1623849
```

```
cor.test(map.join$Zafm, map.join$Zagr)
```

```
## 
##  Pearson's product-moment correlation
## 
## data:  map.join$Zafm and map.join$Zagr
## t = -1.0812, df = 48, p-value = 0.285
## alternative hypothesis: true correlation is not equal to 0
## 95 percent confidence interval:
##  -0.4147437  0.1297183
## sample estimates:
##        cor 
## -0.1541969
```

```
cor.test(map.join$Zafm, map.join$Zcns)
```

```
## 
##  Pearson's product-moment correlation
## 
## data:  map.join$Zafm and map.join$Zcns
## t = -1.896, df = 48, p-value = 0.06399
## alternative hypothesis: true correlation is not equal to 0
## 95 percent confidence interval:
##  -0.5051908  0.0155276
## sample estimates:
##       cor 
## -0.263961
```

```
cor.test(map.join$Zafm, map.join$ZSneu)
```

```
## 
##  Pearson's product-moment correlation
## 
## data:  map.join$Zafm and map.join$ZSneu
## t = 2.7899, df = 48, p-value = 0.00754
## alternative hypothesis: true correlation is not equal to 0
## 95 percent confidence interval:
##  0.1062380 0.5904917
## sample estimates:
##       cor 
## 0.3735398
```

```
cor.test(map.join$Zafm, map.join$Zopn)
```

```
## 
##  Pearson's product-moment correlation
## 
## data:  map.join$Zafm and map.join$Zopn
## t = 2.013, df = 48, p-value = 0.04975
## alternative hypothesis: true correlation is not equal to 0
## 95 percent confidence interval:
##  0.0007157188 0.5171899782
## sample estimates:
##       cor 
## 0.2790079
```

### 6.1.7 Never married

```
cor.test(map.join$ZSnvm, map.join$Zext)
```

```
## 
##  Pearson's product-moment correlation
## 
## data:  map.join$ZSnvm and map.join$Zext
## t = 0.27885, df = 48, p-value = 0.7816
## alternative hypothesis: true correlation is not equal to 0
## 95 percent confidence interval:
##  -0.2408269  0.3150378
## sample estimates:
##        cor 
## 0.04021667
```

```
cor.test(map.join$ZSnvm, map.join$Zagr)
```

```
## 
##  Pearson's product-moment correlation
## 
## data:  map.join$ZSnvm and map.join$Zagr
## t = 1.3998, df = 48, p-value = 0.168
## alternative hypothesis: true correlation is not equal to 0
## 95 percent confidence interval:
##  -0.0849968  0.4514961
## sample estimates:
##       cor 
## 0.1980362
```

```
cor.test(map.join$ZSnvm, map.join$Zcns)
```

```
## 
##  Pearson's product-moment correlation
## 
## data:  map.join$ZSnvm and map.join$Zcns
## t = 0.021774, df = 48, p-value = 0.9827
## alternative hypothesis: true correlation is not equal to 0
## 95 percent confidence interval:
##  -0.2754459  0.2812445
## sample estimates:
##         cor 
## 0.003142784
```

```
cor.test(map.join$ZSnvm, map.join$ZSneu)
```

```
## 
##  Pearson's product-moment correlation
## 
## data:  map.join$ZSnvm and map.join$ZSneu
## t = 0.28472, df = 48, p-value = 0.7771
## alternative hypothesis: true correlation is not equal to 0
## 95 percent confidence interval:
##  -0.2400303  0.3157992
## sample estimates:
##        cor 
## 0.04106076
```

```
cor.test(map.join$ZSnvm, map.join$Zopn)
```

```
## 
##  Pearson's product-moment correlation
## 
## data:  map.join$ZSnvm and map.join$Zopn
## t = 0.27889, df = 48, p-value = 0.7815
## alternative hypothesis: true correlation is not equal to 0
## 95 percent confidence interval:
##  -0.2408221  0.3150424
## sample estimates:
##        cor 
## 0.04022179
```

### 6.1.8 Divorce

```
cor.test(map.join$ZSdiv, map.join$Zext)
```

```
## 
##  Pearson's product-moment correlation
## 
## data:  map.join$ZSdiv and map.join$Zext
## t = -0.40532, df = 48, p-value = 0.687
## alternative hypothesis: true correlation is not equal to 0
## 95 percent confidence interval:
##  -0.3313643  0.2235790
## sample estimates:
##         cor 
## -0.05840335
```

```
cor.test(map.join$ZSdiv, map.join$Zagr)
```

```
## 
##  Pearson's product-moment correlation
## 
## data:  map.join$ZSdiv and map.join$Zagr
## t = -1.1247, df = 48, p-value = 0.2663
## alternative hypothesis: true correlation is not equal to 0
## 95 percent confidence interval:
##  -0.4198555  0.1236280
## sample estimates:
##        cor 
## -0.1602336
```

```
cor.test(map.join$ZSdiv, map.join$Zcns)
```

```
## 
##  Pearson's product-moment correlation
## 
## data:  map.join$ZSdiv and map.join$Zcns
## t = -0.029899, df = 48, p-value = 0.9763
## alternative hypothesis: true correlation is not equal to 0
## 95 percent confidence interval:
##  -0.2823241  0.2743618
## sample estimates:
##          cor 
## -0.004315504
```

```
cor.test(map.join$ZSdiv, map.join$ZSneu)
```

```
## 
##  Pearson's product-moment correlation
## 
## data:  map.join$ZSdiv and map.join$ZSneu
## t = -0.36722, df = 48, p-value = 0.7151
## alternative hypothesis: true correlation is not equal to 0
## 95 percent confidence interval:
##  -0.3264671  0.2287893
## sample estimates:
##         cor 
## -0.05292915
```

```
cor.test(map.join$ZSdiv, map.join$Zopn)
```

```
## 
##  Pearson's product-moment correlation
## 
## data:  map.join$ZSdiv and map.join$Zopn
## t = 0.86094, df = 48, p-value = 0.3936
## alternative hypothesis: true correlation is not equal to 0
## 95 percent confidence interval:
##  -0.1605407  0.3883356
## sample estimates:
##       cor 
## 0.1233176
```

### 6.1.9 Cohabitation

```
cor.test(map.join$Zcoh, map.join$Zext)
```

```
## 
##  Pearson's product-moment correlation
## 
## data:  map.join$Zcoh and map.join$Zext
## t = -1.9094, df = 48, p-value = 0.06219
## alternative hypothesis: true correlation is not equal to 0
## 95 percent confidence interval:
##  -0.50657883  0.01366263
## sample estimates:
##        cor 
## -0.2656955
```

```
cor.test(map.join$Zcoh, map.join$Zagr)
```

```
## 
##  Pearson's product-moment correlation
## 
## data:  map.join$Zcoh and map.join$Zagr
## t = -3.3012, df = 48, p-value = 0.001822
## alternative hypothesis: true correlation is not equal to 0
## 95 percent confidence interval:
##  -0.6327371 -0.1724467
## sample estimates:
##        cor 
## -0.4301473
```

```
cor.test(map.join$Zcoh, map.join$Zcns)
```

```
## 
##  Pearson's product-moment correlation
## 
## data:  map.join$Zcoh and map.join$Zcns
## t = -3.2994, df = 48, p-value = 0.001831
## alternative hypothesis: true correlation is not equal to 0
## 95 percent confidence interval:
##  -0.6326011 -0.1722267
## sample estimates:
##        cor 
## -0.4299625
```

```
cor.test(map.join$Zcoh, map.join$ZSneu)
```

```
## 
##  Pearson's product-moment correlation
## 
## data:  map.join$Zcoh and map.join$ZSneu
## t = 2.2337, df = 48, p-value = 0.0302
## alternative hypothesis: true correlation is not equal to 0
## 95 percent confidence interval:
##  0.03116146 0.53914668
## sample estimates:
##       cor 
## 0.3068477
```

```
cor.test(map.join$Zcoh, map.join$Zopn)
```

```
## 
##  Pearson's product-moment correlation
## 
## data:  map.join$Zcoh and map.join$Zopn
## t = 3.8582, df = 48, p-value = 0.0003403
## alternative hypothesis: true correlation is not equal to 0
## 95 percent confidence interval:
##  0.2407940 0.6736506
## sample estimates:
##       cor 
## 0.4865321
```

### 6.1.10 Non-marital fertility

```
cor.test(map.join$Znmf, map.join$Zext)
```

```
## 
##  Pearson's product-moment correlation
## 
## data:  map.join$Znmf and map.join$Zext
## t = -0.67939, df = 48, p-value = 0.5002
## alternative hypothesis: true correlation is not equal to 0
## 95 percent confidence interval:
##  -0.3659989  0.1858014
## sample estimates:
##         cor 
## -0.09759356
```

```
cor.test(map.join$Znmf, map.join$Zagr)
```

```
## 
##  Pearson's product-moment correlation
## 
## data:  map.join$Znmf and map.join$Zagr
## t = -0.57907, df = 48, p-value = 0.5652
## alternative hypothesis: true correlation is not equal to 0
## 95 percent confidence interval:
##  -0.3534449  0.1996858
## sample estimates:
##         cor 
## -0.08329139
```

```
cor.test(map.join$Znmf, map.join$Zcns)
```

```
## 
##  Pearson's product-moment correlation
## 
## data:  map.join$Znmf and map.join$Zcns
## t = -1.4089, df = 48, p-value = 0.1653
## alternative hypothesis: true correlation is not equal to 0
## 95 percent confidence interval:
##  -0.45252733  0.08371002
## sample estimates:
##       cor 
## -0.199281
```

```
cor.test(map.join$Znmf, map.join$ZSneu)
```

```
## 
##  Pearson's product-moment correlation
## 
## data:  map.join$Znmf and map.join$ZSneu
## t = -0.01581, df = 48, p-value = 0.9875
## alternative hypothesis: true correlation is not equal to 0
## 95 percent confidence interval:
##  -0.2804515  0.2762412
## sample estimates:
##          cor 
## -0.002281932
```

```
cor.test(map.join$Znmf, map.join$Zopn)
```

```
## 
##  Pearson's product-moment correlation
## 
## data:  map.join$Znmf and map.join$Zopn
## t = 0.47569, df = 48, p-value = 0.6365
## alternative hypothesis: true correlation is not equal to 0
## 95 percent confidence interval:
##  -0.2139280  0.3403569
## sample estimates:
##        cor 
## 0.06849856
```

### 6.1.11 Unintended pregnancy

```
cor.test(map.join$Zuni, map.join$Zext)
```

```
## 
##  Pearson's product-moment correlation
## 
## data:  map.join$Zuni and map.join$Zext
## t = -2.7666, df = 48, p-value = 0.00802
## alternative hypothesis: true correlation is not equal to 0
## 95 percent confidence interval:
##  -0.5884522 -0.1031467
## sample estimates:
##        cor 
## -0.3708471
```

```
cor.test(map.join$Zuni, map.join$Zagr)
```

```
## 
##  Pearson's product-moment correlation
## 
## data:  map.join$Zuni and map.join$Zagr
## t = -0.78504, df = 48, p-value = 0.4363
## alternative hypothesis: true correlation is not equal to 0
## 95 percent confidence interval:
##  -0.3790587  0.1711201
## sample estimates:
##        cor 
## -0.1125904
```

```
cor.test(map.join$Zuni, map.join$Zcns)
```

```
## 
##  Pearson's product-moment correlation
## 
## data:  map.join$Zuni and map.join$Zcns
## t = -3.4153, df = 48, p-value = 0.001305
## alternative hypothesis: true correlation is not equal to 0
## 95 percent confidence interval:
##  -0.6415407 -0.1867885
## sample estimates:
##       cor 
## -0.442148
```

```
cor.test(map.join$Zuni, map.join$ZSneu)
```

```
## 
##  Pearson's product-moment correlation
## 
## data:  map.join$Zuni and map.join$ZSneu
## t = 0.82497, df = 48, p-value = 0.4135
## alternative hypothesis: true correlation is not equal to 0
## 95 percent confidence interval:
##  -0.1655576  0.3839502
## sample estimates:
##       cor 
## 0.1182388
```

```
cor.test(map.join$Zuni, map.join$Zopn)
```

```
## 
##  Pearson's product-moment correlation
## 
## data:  map.join$Zuni and map.join$Zopn
## t = 1.2545, df = 48, p-value = 0.2157
## alternative hypothesis: true correlation is not equal to 0
## 95 percent confidence interval:
##  -0.1053956  0.4349550
## sample estimates:
##       cor 
## 0.1781793
```

### 6.1.12 Abortion rate

```
cor.test(map.join$Zabr, map.join$Zext)
```

```
## 
##  Pearson's product-moment correlation
## 
## data:  map.join$Zabr and map.join$Zext
## t = -1.4471, df = 48, p-value = 0.1544
## alternative hypothesis: true correlation is not equal to 0
## 95 percent confidence interval:
##  -0.45681307  0.07834289
## sample estimates:
##        cor 
## -0.2044635
```

```
cor.test(map.join$Zabr, map.join$Zagr)
```

```
## 
##  Pearson's product-moment correlation
## 
## data:  map.join$Zabr and map.join$Zagr
## t = -1.881, df = 48, p-value = 0.06604
## alternative hypothesis: true correlation is not equal to 0
## 95 percent confidence interval:
##  -0.50363328  0.01761612
## sample estimates:
##        cor 
## -0.2620164
```

```
cor.test(map.join$Zabr, map.join$Zcns)
```

```
## 
##  Pearson's product-moment correlation
## 
## data:  map.join$Zabr and map.join$Zcns
## t = -2.4852, df = 48, p-value = 0.01648
## alternative hypothesis: true correlation is not equal to 0
## 95 percent confidence interval:
##  -0.56307590 -0.06545173
## sample estimates:
##        cor 
## -0.3376481
```

```
cor.test(map.join$Zabr, map.join$ZSneu)
```

```
## 
##  Pearson's product-moment correlation
## 
## data:  map.join$Zabr and map.join$ZSneu
## t = 2.6446, df = 48, p-value = 0.01102
## alternative hypothesis: true correlation is not equal to 0
## 95 percent confidence interval:
##  0.08689259 0.57762593
## sample estimates:
##       cor 
## 0.3566151
```

```
cor.test(map.join$Zabr, map.join$Zopn)
```

```
## 
##  Pearson's product-moment correlation
## 
## data:  map.join$Zabr and map.join$Zopn
## t = 1.345, df = 48, p-value = 0.1849
## alternative hypothesis: true correlation is not equal to 0
## 95 percent confidence interval:
##  -0.09268559  0.44530441
## sample estimates:
##       cor 
## 0.1905788
```

### 6.1.13 Family planning expenditures

```
cor.test(map.join$Zfmp, map.join$Zext)
```

```
## 
##  Pearson's product-moment correlation
## 
## data:  map.join$Zfmp and map.join$Zext
## t = -1.8925, df = 48, p-value = 0.06446
## alternative hypothesis: true correlation is not equal to 0
## 95 percent confidence interval:
##  -0.50482774  0.01601489
## sample estimates:
##        cor 
## -0.2635075
```

```
cor.test(map.join$Zfmp, map.join$Zagr)
```

```
## 
##  Pearson's product-moment correlation
## 
## data:  map.join$Zfmp and map.join$Zagr
## t = 0.50532, df = 48, p-value = 0.6156
## alternative hypothesis: true correlation is not equal to 0
## 95 percent confidence interval:
##  -0.2098536  0.3441231
## sample estimates:
##        cor 
## 0.07274318
```

```
cor.test(map.join$Zfmp, map.join$Zcns)
```

```
## 
##  Pearson's product-moment correlation
## 
## data:  map.join$Zfmp and map.join$Zcns
## t = -1.2671, df = 48, p-value = 0.2112
## alternative hypothesis: true correlation is not equal to 0
## 95 percent confidence interval:
##  -0.4364044  0.1036262
## sample estimates:
##        cor 
## -0.1799109
```

```
cor.test(map.join$Zfmp, map.join$ZSneu)
```

```
## 
##  Pearson's product-moment correlation
## 
## data:  map.join$Zfmp and map.join$ZSneu
## t = -0.30967, df = 48, p-value = 0.7582
## alternative hypothesis: true correlation is not equal to 0
## 95 percent confidence interval:
##  -0.3190351  0.2366362
## sample estimates:
##         cor 
## -0.04465265
```

```
cor.test(map.join$Zfmp, map.join$Zopn)
```

```
## 
##  Pearson's product-moment correlation
## 
## data:  map.join$Zfmp and map.join$Zopn
## t = 1.0011, df = 48, p-value = 0.3218
## alternative hypothesis: true correlation is not equal to 0
## 95 percent confidence interval:
##  -0.1409501  0.4052255
## sample estimates:
##       cor 
## 0.1430082
```

### 6.1.14 Total Fertility Rate with Other Fertility Outcomes

```
cor.test(map.join$Ztfr, map.join$Zalpha)
```

```
## 
##  Pearson's product-moment correlation
## 
## data:  map.join$Ztfr and map.join$Zalpha
## t = 1.7528, df = 48, p-value = 0.08601
## alternative hypothesis: true correlation is not equal to 0
## 95 percent confidence interval:
##  -0.0354983  0.4901571
## sample estimates:
##       cor 
## 0.2452729
```

```
cor.test(map.join$Ztfr, map.join$ZSpeak)
```

```
## 
##  Pearson's product-moment correlation
## 
## data:  map.join$Ztfr and map.join$ZSpeak
## t = -0.16184, df = 48, p-value = 0.8721
## alternative hypothesis: true correlation is not equal to 0
## 95 percent confidence interval:
##  -0.2997525  0.2566628
## sample estimates:
##        cor 
## -0.0233533
```

```
cor.test(map.join$Ztfr, map.join$Zstop)
```

```
## 
##  Pearson's product-moment correlation
## 
## data:  map.join$Ztfr and map.join$Zstop
## t = 1.3261, df = 48, p-value = 0.1911
## alternative hypothesis: true correlation is not equal to 0
## 95 percent confidence interval:
##  -0.09534451  0.44315116
## sample estimates:
##       cor 
## 0.1879923
```

```
cor.test(map.join$Ztfr, map.join$ZSafb)
```

```
## 
##  Pearson's product-moment correlation
## 
## data:  map.join$Ztfr and map.join$ZSafb
## t = -5.5112, df = 48, p-value = 1.386e-06
## alternative hypothesis: true correlation is not equal to 0
## 95 percent confidence interval:
##  -0.7678280 -0.4163215
## sample estimates:
##        cor 
## -0.6225292
```

```
cor.test(map.join$Ztfr, map.join$Zafm)
```

```
## 
##  Pearson's product-moment correlation
## 
## data:  map.join$Ztfr and map.join$Zafm
## t = -6.0562, df = 48, p-value = 2.057e-07
## alternative hypothesis: true correlation is not equal to 0
## 95 percent confidence interval:
##  -0.7914912 -0.4649679
## sample estimates:
##        cor 
## -0.6581377
```

```
cor.test(map.join$Ztfr, map.join$ZSnvm)
```

```
## 
##  Pearson's product-moment correlation
## 
## data:  map.join$Ztfr and map.join$ZSnvm
## t = -0.76712, df = 48, p-value = 0.4468
## alternative hypothesis: true correlation is not equal to 0
## 95 percent confidence interval:
##  -0.3768549  0.1736148
## sample estimates:
##        cor 
## -0.1100512
```

```
cor.test(map.join$Ztfr, map.join$ZSdiv)
```

```
## 
##  Pearson's product-moment correlation
## 
## data:  map.join$Ztfr and map.join$ZSdiv
## t = 1.0174, df = 48, p-value = 0.3141
## alternative hypothesis: true correlation is not equal to 0
## 95 percent confidence interval:
##  -0.1386710  0.4071665
## sample estimates:
##       cor 
## 0.1452845
```

```
cor.test(map.join$Ztfr, map.join$Zcoh)
```

```
## 
##  Pearson's product-moment correlation
## 
## data:  map.join$Ztfr and map.join$Zcoh
## t = -6.7154, df = 48, p-value = 2.009e-08
## alternative hypothesis: true correlation is not equal to 0
## 95 percent confidence interval:
##  -0.8162185 -0.5179996
## sample estimates:
##        cor 
## -0.6959958
```

```
cor.test(map.join$Ztfr, map.join$Znmf)
```

```
## 
##  Pearson's product-moment correlation
## 
## data:  map.join$Ztfr and map.join$Znmf
## t = -2.9284, df = 48, p-value = 0.005197
## alternative hypothesis: true correlation is not equal to 0
## 95 percent confidence interval:
##  -0.6023971 -0.1244720
## sample estimates:
##        cor 
## -0.3893308
```

```
cor.test(map.join$Ztfr, map.join$Zuni)
```

```
## 
##  Pearson's product-moment correlation
## 
## data:  map.join$Ztfr and map.join$Zuni
## t = -1.2646, df = 48, p-value = 0.2121
## alternative hypothesis: true correlation is not equal to 0
## 95 percent confidence interval:
##  -0.4361163  0.1039783
## sample estimates:
##        cor 
## -0.1795665
```

```
cor.test(map.join$Ztfr, map.join$Zabr)
```

```
## 
##  Pearson's product-moment correlation
## 
## data:  map.join$Ztfr and map.join$Zabr
## t = -1.4105, df = 48, p-value = 0.1648
## alternative hypothesis: true correlation is not equal to 0
## 95 percent confidence interval:
##  -0.45270863  0.08348361
## sample estimates:
##     cor 
## -0.1995
```

```
cor.test(map.join$Ztfr, map.join$Zfmp)
```

```
## 
##  Pearson's product-moment correlation
## 
## data:  map.join$Ztfr and map.join$Zfmp
## t = -1.046, df = 48, p-value = 0.3008
## alternative hypothesis: true correlation is not equal to 0
## 95 percent confidence interval:
##  -0.4105754  0.1346544
## sample estimates:
##        cor 
## -0.1492888
```

## 6.2 Mediation models for key correlates

A reviewer suggested conducting mediation analyses to test whether any of the other fertility variables could help understand the association between personality and total fertility. These models assume a causal model in which personality influences some sort of intermediary behavior (e.g., age at first birth) which ultimately has an impact on total fertility. While this model seems plausible, we do not have evidence to support strong mediational interpretations. The most likely mediators are those which have a strong association with total fertility, such as age at first birth or marriage and cohabitation. Given the sample size, it is doubtful that we would have statistical power to detect an indirect effect. We evaluated whether the personality-TFR association differed from the previous value to provide a descriptive framing for these analyses. We use bootstrap standard errors with 500 draws due to the indirect effect.

For the personality dimensions that significantly correlated with the total fertility rate (i.e., A, C, N, and O), we modeled as a mediator any other fertility outcome that correlated with personality > |.3|. Thus, we estimated ten models: Agreebleness-cohabitation; Conscientiousness-stopping; Conscientiousness-cohabitation; Conscientiousness-unintended pregnancy; Conscientiousness-abortion; Neuroticism-age at first bith; Neuroticism-age at first marriage; Neuroticism-cohabitation; Neuroticism-abortion; Openness-cohabitation.

### 6.2.1 Agreeableness-cohabitation

The previous correlation was .51.

```
agr.coh<-"
Ztfr ~ c*Zagr + b*Zcoh
Zcoh ~ a*Zagr

ind:=a*b
red:=.51-c
percentred:=(red/.51)*100
"
agr.coh.fit<-sem(agr.coh, as.data.frame(map.join), bootstrap = 500)
summary(agr.coh.fit)
```

```
## lavaan 0.6-6 ended normally after 14 iterations
## 
##   Estimator                                         ML
##   Optimization method                           NLMINB
##   Number of free parameters                          5
##                                                       
##   Number of observations                            50
##                                                       
## Model Test User Model:
##                                                       
##   Test statistic                                 0.000
##   Degrees of freedom                                 0
## 
## Parameter Estimates:
## 
##   Standard errors                             Standard
##   Information                                 Expected
##   Information saturated (h1) model          Structured
## 
## Regressions:
##                    Estimate  Std.Err  z-value  P(>|z|)
##   Ztfr ~                                              
##     Zagr       (c)    0.259    0.106    2.435    0.015
##     Zcoh       (b)   -0.585    0.106   -5.497    0.000
##   Zcoh ~                                              
##     Zagr       (a)   -0.430    0.128   -3.369    0.001
## 
## Variances:
##                    Estimate  Std.Err  z-value  P(>|z|)
##    .Ztfr              0.452    0.090    5.000    0.000
##    .Zcoh              0.799    0.160    5.000    0.000
## 
## Defined Parameters:
##                    Estimate  Std.Err  z-value  P(>|z|)
##     ind               0.251    0.088    2.873    0.004
##     red               0.251    0.106    2.361    0.018
##     percentred       49.227   20.854    2.361    0.018
```

Results indicate that a significant indirect effect (ind in the Defined Parameters section), implying that some of the association between agreeableness and total fertility rate could be mediated by cohabitation. Further, the raw association of .51 was reduced by .251 correlation units (red in the Defined Parameters section), or approximately 49.23% of the association.

### 6.2.2 Conscientiousness-stopping

The previous correlation was .42.

```
cns.stop<-"
Ztfr ~ c*Zcns + b*Zstop
Zstop ~ a*Zcns

ind:=a*b
red:=.42-c
percentred:=(red/.42)*100
"
cns.stop.fit<-sem(cns.stop, as.data.frame(map.join), bootstrap = 500)
summary(cns.stop.fit)
```

```
## lavaan 0.6-6 ended normally after 11 iterations
## 
##   Estimator                                         ML
##   Optimization method                           NLMINB
##   Number of free parameters                          5
##                                                       
##   Number of observations                            50
##                                                       
## Model Test User Model:
##                                                       
##   Test statistic                                 0.000
##   Degrees of freedom                                 0
## 
## Parameter Estimates:
## 
##   Standard errors                             Standard
##   Information                                 Expected
##   Information saturated (h1) model          Structured
## 
## Regressions:
##                    Estimate  Std.Err  z-value  P(>|z|)
##   Ztfr ~                                              
##     Zcns       (c)    0.410    0.137    2.988    0.003
##     Zstop      (b)    0.041    0.137    0.298    0.766
##   Zstop ~                                             
##     Zcns       (a)    0.359    0.132    2.722    0.006
## 
## Variances:
##                    Estimate  Std.Err  z-value  P(>|z|)
##    .Ztfr              0.802    0.160    5.000    0.000
##    .Zstop             0.854    0.171    5.000    0.000
## 
## Defined Parameters:
##                    Estimate  Std.Err  z-value  P(>|z|)
##     ind               0.015    0.050    0.296    0.767
##     red               0.010    0.137    0.076    0.940
##     percentred        2.467   32.642    0.076    0.940
```

Results indicate a non-significant indirect effect. Further, the raw association of .42 was reduced by .01 correlation units, or approximately 2.47% of the association.

### 6.2.3 Conscientiousness-cohabitation

The previous correlation was .42.

```
cns.coh<-"
Ztfr ~ c*Zcns + b*Zcoh
Zcoh ~ a*Zcns

ind:=a*b
red:=.42-c
percentred:=(red/.42)*100
"
cns.coh.fit<-sem(cns.coh, as.data.frame(map.join), bootstrap = 500)
summary(cns.coh.fit)
```

```
## lavaan 0.6-6 ended normally after 13 iterations
## 
##   Estimator                                         ML
##   Optimization method                           NLMINB
##   Number of free parameters                          5
##                                                       
##   Number of observations                            50
##                                                       
## Model Test User Model:
##                                                       
##   Test statistic                                 0.000
##   Degrees of freedom                                 0
## 
## Parameter Estimates:
## 
##   Standard errors                             Standard
##   Information                                 Expected
##   Information saturated (h1) model          Structured
## 
## Regressions:
##                    Estimate  Std.Err  z-value  P(>|z|)
##   Ztfr ~                                              
##     Zcns       (c)    0.153    0.110    1.390    0.164
##     Zcoh       (b)   -0.630    0.110   -5.709    0.000
##   Zcoh ~                                              
##     Zcns       (a)   -0.430    0.128   -3.367    0.001
## 
## Variances:
##                    Estimate  Std.Err  z-value  P(>|z|)
##    .Ztfr              0.486    0.097    5.000    0.000
##    .Zcoh              0.799    0.160    5.000    0.000
## 
## Defined Parameters:
##                    Estimate  Std.Err  z-value  P(>|z|)
##     ind               0.271    0.093    2.900    0.004
##     red               0.267    0.110    2.416    0.016
##     percentred       63.472   26.277    2.416    0.016
```

Results indicate a significant indirect effect. Further, the raw association of .42 was reduced by .27 correlation units, or approximately 63.47% of the association.

### 6.2.4 Conscientiousness-unintended pregnancy

The previous correlation was .42.

```
cns.uni<-"
Ztfr ~ c*Zcns + b*Zuni
Zuni ~ a*Zcns

ind:=a*b
red:=.42-c
percentred:=(red/.42)*100
"
cns.uni.fit<-sem(cns.uni, as.data.frame(map.join), bootstrap = 500)
summary(cns.uni.fit)
```

```
## lavaan 0.6-6 ended normally after 11 iterations
## 
##   Estimator                                         ML
##   Optimization method                           NLMINB
##   Number of free parameters                          5
##                                                       
##   Number of observations                            50
##                                                       
## Model Test User Model:
##                                                       
##   Test statistic                                 0.000
##   Degrees of freedom                                 0
## 
## Parameter Estimates:
## 
##   Standard errors                             Standard
##   Information                                 Expected
##   Information saturated (h1) model          Structured
## 
## Regressions:
##                    Estimate  Std.Err  z-value  P(>|z|)
##   Ztfr ~                                              
##     Zcns       (c)    0.429    0.143    3.003    0.003
##     Zuni       (b)    0.010    0.143    0.070    0.944
##   Zuni ~                                              
##     Zcns       (a)   -0.442    0.127   -3.486    0.000
## 
## Variances:
##                    Estimate  Std.Err  z-value  P(>|z|)
##    .Ztfr              0.803    0.161    5.000    0.000
##    .Zuni              0.788    0.158    5.000    0.000
## 
## Defined Parameters:
##                    Estimate  Std.Err  z-value  P(>|z|)
##     ind              -0.004    0.063   -0.070    0.944
##     red              -0.009    0.143   -0.061    0.951
##     percentred       -2.078   33.992   -0.061    0.951
```

Results indicate a non-significant indirect effect. Further, the raw association of .42 was not reduced.

### 6.2.5 Conscientiousness-abortion

The previous correlation was .42.

```
cns.abr<-"
Ztfr ~ c*Zcns + b*Zabr
Zabr ~ a*Zcns

ind:=a*b
red:=.42-c
percentred:=(red/.42)*100
"
cns.abr.fit<-sem(cns.abr, as.data.frame(map.join), bootstrap = 500)
summary(cns.abr.fit)
```

```
## lavaan 0.6-6 ended normally after 10 iterations
## 
##   Estimator                                         ML
##   Optimization method                           NLMINB
##   Number of free parameters                          5
##                                                       
##   Number of observations                            50
##                                                       
## Model Test User Model:
##                                                       
##   Test statistic                                 0.000
##   Degrees of freedom                                 0
## 
## Parameter Estimates:
## 
##   Standard errors                             Standard
##   Information                                 Expected
##   Information saturated (h1) model          Structured
## 
## Regressions:
##                    Estimate  Std.Err  z-value  P(>|z|)
##   Ztfr ~                                              
##     Zcns       (c)    0.403    0.136    2.968    0.003
##     Zabr       (b)   -0.063    0.136   -0.468    0.640
##   Zabr ~                                              
##     Zcns       (a)   -0.338    0.133   -2.536    0.011
## 
## Variances:
##                    Estimate  Std.Err  z-value  P(>|z|)
##    .Ztfr              0.800    0.160    5.000    0.000
##    .Zabr              0.868    0.174    5.000    0.000
## 
## Defined Parameters:
##                    Estimate  Std.Err  z-value  P(>|z|)
##     ind               0.021    0.047    0.460    0.646
##     red               0.017    0.136    0.126    0.900
##     percentred        4.077   32.322    0.126    0.900
```

Results indicate a non-significant indirect effect. Further, the raw association of .42 was reduced by .02 correlation units, or approximately 4.08% of the association.

### 6.2.6 Neuroticism-age at first birth

The previous correlation was -.49.

```
neu.afb<-"
Ztfr ~ c*ZSneu + b*ZSafb
ZSafb ~ a*ZSneu

ind:=a*b
red:=-.49-c
percentred:=(red/-.49)*100
"
neu.afb.fit<-sem(neu.afb, as.data.frame(map.join), bootstrap = 500)
summary(neu.afb.fit)
```

```
## lavaan 0.6-6 ended normally after 13 iterations
## 
##   Estimator                                         ML
##   Optimization method                           NLMINB
##   Number of free parameters                          5
##                                                       
##   Number of observations                            50
##                                                       
## Model Test User Model:
##                                                       
##   Test statistic                                 0.000
##   Degrees of freedom                                 0
## 
## Parameter Estimates:
## 
##   Standard errors                             Standard
##   Information                                 Expected
##   Information saturated (h1) model          Structured
## 
## Regressions:
##                    Estimate  Std.Err  z-value  P(>|z|)
##   Ztfr ~                                              
##     ZSneu      (c)   -0.298    0.112   -2.660    0.008
##     ZSafb      (b)   -0.508    0.112   -4.528    0.000
##   ZSafb ~                                             
##     ZSneu      (a)    0.384    0.131    2.940    0.003
## 
## Variances:
##                    Estimate  Std.Err  z-value  P(>|z|)
##    .Ztfr              0.526    0.105    5.000    0.000
##    .ZSafb             0.836    0.167    5.000    0.000
## 
## Defined Parameters:
##                    Estimate  Std.Err  z-value  P(>|z|)
##     ind              -0.195    0.079   -2.466    0.014
##     red              -0.192    0.112   -1.708    0.088
##     percentred       39.092   22.894    1.708    0.088
```

Results indicate a significant indirect effect. Further, the raw association of -.49 was reduced by .19 correlation units, or approximately 39.09% of the association.

### 6.2.7 Neuroticism-age at first marriage

The previous correlation was -.49.

```
neu.afm<-"
Ztfr ~ c*ZSneu + b*Zafm
Zafm ~ a*ZSneu

ind:=a*b
red:=-.49-c
percentred:=(red/-.49)*100
"
neu.afm.fit<-sem(neu.afm, as.data.frame(map.join), bootstrap = 500)
summary(neu.afm.fit)
```

```
## lavaan 0.6-6 ended normally after 12 iterations
## 
##   Estimator                                         ML
##   Optimization method                           NLMINB
##   Number of free parameters                          5
##                                                       
##   Number of observations                            50
##                                                       
## Model Test User Model:
##                                                       
##   Test statistic                                 0.000
##   Degrees of freedom                                 0
## 
## Parameter Estimates:
## 
##   Standard errors                             Standard
##   Information                                 Expected
##   Information saturated (h1) model          Structured
## 
## Regressions:
##                    Estimate  Std.Err  z-value  P(>|z|)
##   Ztfr ~                                              
##     ZSneu      (c)   -0.288    0.107   -2.681    0.007
##     Zafm       (b)   -0.551    0.107   -5.131    0.000
##   Zafm ~                                              
##     ZSneu      (a)    0.374    0.131    2.847    0.004
## 
## Variances:
##                    Estimate  Std.Err  z-value  P(>|z|)
##    .Ztfr              0.486    0.097    5.000    0.000
##    .Zafm              0.843    0.169    5.000    0.000
## 
## Defined Parameters:
##                    Estimate  Std.Err  z-value  P(>|z|)
##     ind              -0.206    0.083   -2.490    0.013
##     red              -0.202    0.107   -1.884    0.060
##     percentred       41.276   21.904    1.884    0.060
```

Results indicate a significant indirect effect. Further, the raw association of -.49 was reduced by .2 correlation units, or approximately 41.28% of the association.

### 6.2.8 Neuroticism-cohabitation

The previous correlation was -.49.

```
neu.coh<-"
Ztfr ~ c*ZSneu + b*Zcoh
Zcoh ~ a*ZSneu

ind:=a*b
red:=-.49-c
percentred:=(red/-.49)*100
"
neu.coh.fit<-sem(neu.coh, as.data.frame(map.join), bootstrap = 500)
summary(neu.coh.fit)
```

```
## lavaan 0.6-6 ended normally after 13 iterations
## 
##   Estimator                                         ML
##   Optimization method                           NLMINB
##   Number of free parameters                          5
##                                                       
##   Number of observations                            50
##                                                       
## Model Test User Model:
##                                                       
##   Test statistic                                 0.000
##   Degrees of freedom                                 0
## 
## Parameter Estimates:
## 
##   Standard errors                             Standard
##   Information                                 Expected
##   Information saturated (h1) model          Structured
## 
## Regressions:
##                    Estimate  Std.Err  z-value  P(>|z|)
##   Ztfr ~                                              
##     ZSneu      (c)   -0.309    0.097   -3.174    0.002
##     Zcoh       (b)   -0.601    0.097   -6.176    0.000
##   Zcoh ~                                              
##     ZSneu      (a)    0.307    0.135    2.280    0.023
## 
## Variances:
##                    Estimate  Std.Err  z-value  P(>|z|)
##    .Ztfr              0.421    0.084    5.000    0.000
##    .Zcoh              0.888    0.178    5.000    0.000
## 
## Defined Parameters:
##                    Estimate  Std.Err  z-value  P(>|z|)
##     ind              -0.184    0.086   -2.139    0.032
##     red              -0.181    0.097   -1.860    0.063
##     percentred       36.946   19.865    1.860    0.063
```

Results indicate a significant indirect effect. Further, the raw association of -.49 was reduced by .18 correlation units, or approximately 36.95% of the association.

### 6.2.9 Neuroticism-abortion

The previous correlation was -.49.

```
neu.abr<-"
Ztfr ~ c*ZSneu + b*Zabr
Zabr ~ a*ZSneu

ind:=a*b
red:=-.49-c
percentred:=(red/-.49)*100
"
neu.abr.fit<-sem(neu.abr, as.data.frame(map.join), bootstrap = 500)
summary(neu.abr.fit)
```

```
## lavaan 0.6-6 ended normally after 10 iterations
## 
##   Estimator                                         ML
##   Optimization method                           NLMINB
##   Number of free parameters                          5
##                                                       
##   Number of observations                            50
##                                                       
## Model Test User Model:
##                                                       
##   Test statistic                                 0.000
##   Degrees of freedom                                 0
## 
## Parameter Estimates:
## 
##   Standard errors                             Standard
##   Information                                 Expected
##   Information saturated (h1) model          Structured
## 
## Regressions:
##                    Estimate  Std.Err  z-value  P(>|z|)
##   Ztfr ~                                              
##     ZSneu      (c)   -0.484    0.132   -3.676    0.000
##     Zabr       (b)   -0.027    0.132   -0.205    0.838
##   Zabr ~                                              
##     ZSneu      (a)    0.357    0.132    2.699    0.007
## 
## Variances:
##                    Estimate  Std.Err  z-value  P(>|z|)
##    .Ztfr              0.741    0.148    5.000    0.000
##    .Zabr              0.855    0.171    5.000    0.000
## 
## Defined Parameters:
##                    Estimate  Std.Err  z-value  P(>|z|)
##     ind              -0.010    0.047   -0.204    0.838
##     red              -0.006    0.132   -0.047    0.963
##     percentred        1.260   26.859    0.047    0.963
```

Results indicate a non-significant indirect effect. Further, the raw association of -.49 was reduced by .01 correlation units, or approximately 1.26% of the association.

### 6.2.10 Openness-cohabitation

The previous correlation was -.53.

```
opn.coh<-"
Ztfr ~ c*Zopn + b*Zcoh
Zcoh ~ a*Zopn

ind:=a*b
red:=-.53-c
percentred:=(red/-.53)*100
"
opn.coh.fit<-sem(opn.coh, as.data.frame(map.join), bootstrap = 500)
summary(opn.coh.fit)
```

```
## lavaan 0.6-6 ended normally after 13 iterations
## 
##   Estimator                                         ML
##   Optimization method                           NLMINB
##   Number of free parameters                          5
##                                                       
##   Number of observations                            50
##                                                       
## Model Test User Model:
##                                                       
##   Test statistic                                 0.000
##   Degrees of freedom                                 0
## 
## Parameter Estimates:
## 
##   Standard errors                             Standard
##   Information                                 Expected
##   Information saturated (h1) model          Structured
## 
## Regressions:
##                    Estimate  Std.Err  z-value  P(>|z|)
##   Ztfr ~                                              
##     Zopn       (c)   -0.251    0.111   -2.270    0.023
##     Zcoh       (b)   -0.574    0.111   -5.185    0.000
##   Zcoh ~                                              
##     Zopn       (a)    0.487    0.124    3.938    0.000
## 
## Variances:
##                    Estimate  Std.Err  z-value  P(>|z|)
##    .Ztfr              0.458    0.092    5.000    0.000
##    .Zcoh              0.748    0.150    5.000    0.000
## 
## Defined Parameters:
##                    Estimate  Std.Err  z-value  P(>|z|)
##     ind              -0.279    0.089   -3.136    0.002
##     red              -0.279    0.111   -2.519    0.012
##     percentred       52.597   20.881    2.519    0.012
```

Results indicate a significant indirect effect. Further, the raw association of -.53 was reduced by .28 correlation units, or approximately 52.60% of the association.

## 6.3 Male-Female personality

In this section, we report a variety of models intended to test whether male or female state-level personality is more strongly or differentially associated with fertility outcomes. As described in the methods, male/female personality aggregates tended to be strongly correlated.

```
(cor(map.join$Zmext,map.join$Zfext)+
cor(map.join$Zmagr,map.join$Zfagr)+
cor(map.join$Zmcns,map.join$Zfcns)+
cor(map.join$Zmneu,map.join$ZSfneu)+
cor(map.join$Zmopn,map.join$Zfopn))/5
```

```
##           [,1]
## [1,] 0.7100771
```

### 6.3.1 Extraversion

```
reg.tfr.gender.Ext<-lm(Ztfr~Zmext+Zfext, data=map.join@data)
summary(reg.tfr.gender.Ext)
```

```
## 
## Call:
## lm(formula = Ztfr ~ Zmext + Zfext, data = map.join@data)
## 
## Residuals:
##      Min       1Q   Median       3Q      Max 
## -2.34772 -0.62490 -0.05085  0.47537  2.42185 
## 
## Coefficients:
##              Estimate Std. Error t value Pr(>|t|)
## (Intercept) 4.047e-18  1.401e-01   0.000    1.000
## Zmext       2.351e-01  2.213e-01   1.062    0.293
## Zfext       9.743e-03  2.213e-01   0.044    0.965
## 
## Residual standard error: 0.9905 on 47 degrees of freedom
## Multiple R-squared:  0.05889,    Adjusted R-squared:  0.01884 
## F-statistic: 1.471 on 2 and 47 DF,  p-value: 0.2402
```

```
tfr.gender.Ext<-commonalityCoefficients(map.join@data,"Ztfr", list("Zmext", "Zfext"))
tfr.gender.Ext
```

```
## $CC
##                            Coefficient     % Total
## Unique to Zmext                 0.0226       38.38
## Unique to Zfext                 0.0000        0.07
## Common to Zmext, and Zfext      0.0363       61.56
## Total                           0.0589      100.00
## 
## $CCTotalbyVar
##       Unique Common  Total
## Zmext 0.0226 0.0363 0.0589
## Zfext 0.0000 0.0363 0.0363
```

```
reg.alp.gender.Ext<-lm(Zalpha~Zmext+Zfext, data=map.join@data)
summary(reg.alp.gender.Ext)
```

```
## 
## Call:
## lm(formula = Zalpha ~ Zmext + Zfext, data = map.join@data)
## 
## Residuals:
##      Min       1Q   Median       3Q      Max 
## -2.67818 -0.32191  0.08391  0.46245  2.17561 
## 
## Coefficients:
##               Estimate Std. Error t value Pr(>|t|)
## (Intercept) -2.877e-17  1.410e-01   0.000    1.000
## Zmext       -1.601e-01  2.227e-01  -0.719    0.476
## Zfext       -6.708e-02  2.227e-01  -0.301    0.765
## 
## Residual standard error: 0.997 on 47 degrees of freedom
## Multiple R-squared:  0.04664,    Adjusted R-squared:  0.006076 
## F-statistic:  1.15 on 2 and 47 DF,  p-value: 0.3255
```

```
alp.gender.Ext<-commonalityCoefficients(map.join@data,"Zalpha", list("Zmext", "Zfext"))
alp.gender.Ext
```

```
## $CC
##                            Coefficient     % Total
## Unique to Zmext                 0.0105       22.47
## Unique to Zfext                 0.0018        3.94
## Common to Zmext, and Zfext      0.0343       73.59
## Total                           0.0466      100.00
## 
## $CCTotalbyVar
##       Unique Common  Total
## Zmext 0.0105 0.0343 0.0448
## Zfext 0.0018 0.0344 0.0362
```

```
reg.pea.gender.Ext<-lm(ZSpeak~Zmext+Zfext, data=map.join@data)
summary(reg.pea.gender.Ext)
```

```
## 
## Call:
## lm(formula = ZSpeak ~ Zmext + Zfext, data = map.join@data)
## 
## Residuals:
##      Min       1Q   Median       3Q      Max 
## -2.04295 -0.54233 -0.04307  0.67126  2.59169 
## 
## Coefficients:
##              Estimate Std. Error t value Pr(>|t|)
## (Intercept) 6.587e-17  1.389e-01   0.000    1.000
## Zmext       2.280e-01  2.195e-01   1.039    0.304
## Zfext       5.463e-02  2.195e-01   0.249    0.805
## 
## Residual standard error: 0.9825 on 47 degrees of freedom
## Multiple R-squared:  0.07415,    Adjusted R-squared:  0.03475 
## F-statistic: 1.882 on 2 and 47 DF,  p-value: 0.1636
```

```
pea.gender.Ext<-commonalityCoefficients(map.join@data,"ZSpeak", list("Zmext", "Zfext"))
pea.gender.Ext
```

```
## $CC
##                            Coefficient     % Total
## Unique to Zmext                 0.0213       28.68
## Unique to Zfext                 0.0012        1.65
## Common to Zmext, and Zfext      0.0517       69.67
## Total                           0.0741      100.00
## 
## $CCTotalbyVar
##       Unique Common  Total
## Zmext 0.0213 0.0516 0.0729
## Zfext 0.0012 0.0517 0.0529
```

```
reg.stp.gender.Ext<-lm(Zstop~Zmext+Zfext, data=map.join@data)
summary(reg.stp.gender.Ext)
```

```
## 
## Call:
## lm(formula = Zstop ~ Zmext + Zfext, data = map.join@data)
## 
## Residuals:
##     Min      1Q  Median      3Q     Max 
## -3.5131 -0.5580  0.1574  0.5284  1.8078 
## 
## Coefficients:
##               Estimate Std. Error t value Pr(>|t|)   
## (Intercept)  2.809e-17  1.288e-01   0.000  1.00000   
## Zmext       -1.488e-01  2.035e-01  -0.731  0.46822   
## Zfext        5.561e-01  2.035e-01   2.732  0.00883 **
## ---
## Signif. codes:  0 '***' 0.001 '**' 0.01 '*' 0.05 '.' 0.1 ' ' 1
## 
## Residual standard error: 0.9109 on 47 degrees of freedom
## Multiple R-squared:  0.2041, Adjusted R-squared:  0.1702 
## F-statistic: 6.026 on 2 and 47 DF,  p-value: 0.004679
```

```
stp.gender.Ext<-commonalityCoefficients(map.join@data,"Zstop", list("Zmext", "Zfext"))
stp.gender.Ext
```

```
## $CC
##                            Coefficient     % Total
## Unique to Zmext                 0.0091        4.44
## Unique to Zfext                 0.1264       61.95
## Common to Zmext, and Zfext      0.0686       33.62
## Total                           0.2041      100.00
## 
## $CCTotalbyVar
##       Unique Common  Total
## Zmext 0.0091 0.0686 0.0777
## Zfext 0.1264 0.0686 0.1950
```

```
reg.afb.gender.Ext<-lm(ZSafb~Zmext+Zfext, data=map.join@data)
summary(reg.afb.gender.Ext)
```

```
## 
## Call:
## lm(formula = ZSafb ~ Zmext + Zfext, data = map.join@data)
## 
## Residuals:
##     Min      1Q  Median      3Q     Max 
## -2.7819 -0.6015  0.0494  0.6977  2.3038 
## 
## Coefficients:
##               Estimate Std. Error t value Pr(>|t|)
## (Intercept) -2.335e-17  1.422e-01   0.000    1.000
## Zmext        2.495e-01  2.247e-01   1.111    0.272
## Zfext       -2.595e-01  2.247e-01  -1.155    0.254
## 
## Residual standard error: 1.006 on 47 degrees of freedom
## Multiple R-squared:  0.03004,    Adjusted R-squared:  -0.01124 
## F-statistic: 0.7278 on 2 and 47 DF,  p-value: 0.4883
```

```
afb.gender.Ext<-commonalityCoefficients(map.join@data,"ZSafb", list("Zmext", "Zfext"))
afb.gender.Ext
```

```
## $CC
##                            Coefficient     % Total
## Unique to Zmext                 0.0255       84.73
## Unique to Zfext                 0.0275       91.70
## Common to Zmext, and Zfext     -0.0230      -76.42
## Total                           0.0300      100.00
## 
## $CCTotalbyVar
##       Unique  Common  Total
## Zmext 0.0255 -0.0230 0.0025
## Zfext 0.0275 -0.0229 0.0046
```

```
reg.afm.gender.Ext<-lm(Zafm~Zmext+Zfext, data=map.join@data)
summary(reg.afm.gender.Ext)
```

```
## 
## Call:
## lm(formula = Zafm ~ Zmext + Zfext, data = map.join@data)
## 
## Residuals:
##      Min       1Q   Median       3Q      Max 
## -2.98901 -0.55673  0.09092  0.53706  1.93824 
## 
## Coefficients:
##               Estimate Std. Error t value Pr(>|t|)
## (Intercept)  1.617e-17  1.428e-01   0.000    1.000
## Zmext        8.192e-02  2.256e-01   0.363    0.718
## Zfext       -2.013e-01  2.256e-01  -0.892    0.377
## 
## Residual standard error: 1.01 on 47 degrees of freedom
## Multiple R-squared:  0.02187,    Adjusted R-squared:  -0.01975 
## F-statistic: 0.5254 on 2 and 47 DF,  p-value: 0.5947
```

```
afm.gender.Ext<-commonalityCoefficients(map.join@data,"Zafm", list("Zmext", "Zfext"))
afm.gender.Ext
```

```
## $CC
##                            Coefficient     % Total
## Unique to Zmext                 0.0027       12.55
## Unique to Zfext                 0.0166       75.75
## Common to Zmext, and Zfext      0.0026       11.70
## Total                           0.0219      100.00
## 
## $CCTotalbyVar
##       Unique Common  Total
## Zmext 0.0027 0.0026 0.0053
## Zfext 0.0166 0.0025 0.0191
```

```
reg.nvm.gender.Ext<-lm(ZSnvm~Zmext+Zfext, data=map.join@data)
summary(reg.nvm.gender.Ext)
```

```
## 
## Call:
## lm(formula = ZSnvm ~ Zmext + Zfext, data = map.join@data)
## 
## Residuals:
##     Min      1Q  Median      3Q     Max 
## -2.2831 -0.5447 -0.2199  0.6032  2.8428 
## 
## Coefficients:
##               Estimate Std. Error t value Pr(>|t|)
## (Intercept) -2.312e-17  1.441e-01   0.000    1.000
## Zmext        7.701e-02  2.276e-01   0.338    0.737
## Zfext       -1.636e-02  2.276e-01  -0.072    0.943
## 
## Residual standard error: 1.019 on 47 degrees of freedom
## Multiple R-squared:  0.004261,   Adjusted R-squared:  -0.03811 
## F-statistic: 0.1006 on 2 and 47 DF,  p-value: 0.9045
```

```
nvm.gender.Ext<-commonalityCoefficients(map.join@data,"ZSnvm", list("Zmext", "Zfext"))
nvm.gender.Ext
```

```
## $CC
##                            Coefficient     % Total
## Unique to Zmext                 0.0024       56.91
## Unique to Zfext                 0.0001        2.57
## Common to Zmext, and Zfext      0.0017       40.52
## Total                           0.0043      100.00
## 
## $CCTotalbyVar
##       Unique Common  Total
## Zmext 0.0024 0.0018 0.0042
## Zfext 0.0001 0.0017 0.0018
```

```
reg.div.gender.Ext<-lm(ZSdiv~Zmext+Zfext, data=map.join@data)
summary(reg.div.gender.Ext)
```

```
## 
## Call:
## lm(formula = ZSdiv ~ Zmext + Zfext, data = map.join@data)
## 
## Residuals:
##     Min      1Q  Median      3Q     Max 
## -1.6750 -0.7767 -0.1279  0.6570  1.8856 
## 
## Coefficients:
##               Estimate Std. Error t value Pr(>|t|)
## (Intercept) -2.856e-17  1.427e-01   0.000    1.000
## Zmext       -2.130e-01  2.255e-01  -0.945    0.350
## Zfext        9.601e-02  2.255e-01   0.426    0.672
## 
## Residual standard error: 1.009 on 47 degrees of freedom
## Multiple R-squared:  0.02313,    Adjusted R-squared:  -0.01844 
## F-statistic: 0.5564 on 2 and 47 DF,  p-value: 0.577
```

```
div.gender.Ext<-commonalityCoefficients(map.join@data,"ZSdiv", list("Zmext", "Zfext"))
div.gender.Ext
```

```
## $CC
##                            Coefficient     % Total
## Unique to Zmext                 0.0185       80.17
## Unique to Zfext                 0.0038       16.30
## Common to Zmext, and Zfext      0.0008        3.53
## Total                           0.0231      100.00
## 
## $CCTotalbyVar
##       Unique Common  Total
## Zmext 0.0185  9e-04 0.0194
## Zfext 0.0038  8e-04 0.0046
```

```
reg.coh.gender.Ext<-lm(Zcoh~Zmext+Zfext, data=map.join@data)
summary(reg.coh.gender.Ext)
```

```
## 
## Call:
## lm(formula = Zcoh ~ Zmext + Zfext, data = map.join@data)
## 
## Residuals:
##      Min       1Q   Median       3Q      Max 
## -1.92794 -0.60793 -0.09338  0.50677  2.69678 
## 
## Coefficients:
##               Estimate Std. Error t value Pr(>|t|)
## (Intercept)  7.885e-19  1.392e-01   0.000    1.000
## Zmext       -1.625e-01  2.199e-01  -0.739    0.463
## Zfext       -1.206e-01  2.199e-01  -0.548    0.586
## 
## Residual standard error: 0.9841 on 47 degrees of freedom
## Multiple R-squared:  0.07109,    Adjusted R-squared:  0.03156 
## F-statistic: 1.799 on 2 and 47 DF,  p-value: 0.1767
```

```
coh.gender.Ext<-commonalityCoefficients(map.join@data,"Zcoh", list("Zmext", "Zfext"))
coh.gender.Ext
```

```
## $CC
##                            Coefficient     % Total
## Unique to Zmext                 0.0108       15.20
## Unique to Zfext                 0.0059        8.36
## Common to Zmext, and Zfext      0.0543       76.44
## Total                           0.0711      100.00
## 
## $CCTotalbyVar
##       Unique Common  Total
## Zmext 0.0108 0.0543 0.0651
## Zfext 0.0059 0.0544 0.0603
```

```
reg.nmf.gender.Ext<-lm(Znmf~Zmext+Zfext, data=map.join@data)
summary(reg.nmf.gender.Ext)
```

```
## 
## Call:
## lm(formula = Znmf ~ Zmext + Zfext, data = map.join@data)
## 
## Residuals:
##      Min       1Q   Median       3Q      Max 
## -3.06413 -0.49069  0.00482  0.55096  2.07081 
## 
## Coefficients:
##               Estimate Std. Error t value Pr(>|t|)  
## (Intercept)  3.674e-18  1.358e-01   0.000   1.0000  
## Zmext       -5.314e-01  2.145e-01  -2.477   0.0169 *
## Zfext        4.080e-01  2.145e-01   1.902   0.0633 .
## ---
## Signif. codes:  0 '***' 0.001 '**' 0.01 '*' 0.05 '.' 0.1 ' ' 1
## 
## Residual standard error: 0.9603 on 47 degrees of freedom
## Multiple R-squared:  0.1155, Adjusted R-squared:  0.07781 
## F-statistic: 3.067 on 2 and 47 DF,  p-value: 0.05597
```

```
nmf.gender.Ext<-commonalityCoefficients(map.join@data,"Znmf", list("Zmext", "Zfext"))
nmf.gender.Ext
```

```
## $CC
##                            Coefficient     % Total
## Unique to Zmext                 0.1155      100.00
## Unique to Zfext                 0.0681       58.95
## Common to Zmext, and Zfext     -0.0681      -58.95
## Total                           0.1155      100.00
## 
## $CCTotalbyVar
##       Unique  Common  Total
## Zmext 0.1155 -0.0681 0.0474
## Zfext 0.0681 -0.0681 0.0000
```

```
reg.uni.gender.Ext<-lm(Zuni~Zmext+Zfext, data=map.join@data)
summary(reg.uni.gender.Ext)
```

```
## 
## Call:
## lm(formula = Zuni ~ Zmext + Zfext, data = map.join@data)
## 
## Residuals:
##      Min       1Q   Median       3Q      Max 
## -2.61810 -0.35107  0.09655  0.58428  2.57533 
## 
## Coefficients:
##               Estimate Std. Error t value Pr(>|t|)  
## (Intercept) -8.176e-18  1.318e-01   0.000   1.0000  
## Zmext       -4.388e-01  2.082e-01  -2.108   0.0404 *
## Zfext        3.993e-02  2.082e-01   0.192   0.8487  
## ---
## Signif. codes:  0 '***' 0.001 '**' 0.01 '*' 0.05 '.' 0.1 ' ' 1
## 
## Residual standard error: 0.9318 on 47 degrees of freedom
## Multiple R-squared:  0.1672, Adjusted R-squared:  0.1318 
## F-statistic: 4.718 on 2 and 47 DF,  p-value: 0.01357
```

```
uni.gender.Ext<-commonalityCoefficients(map.join@data,"Zuni", list("Zmext", "Zfext"))
uni.gender.Ext
```

```
## $CC
##                            Coefficient     % Total
## Unique to Zmext                 0.0787       47.09
## Unique to Zfext                 0.0007        0.39
## Common to Zmext, and Zfext      0.0878       52.52
## Total                           0.1672      100.00
## 
## $CCTotalbyVar
##       Unique Common  Total
## Zmext 0.0787 0.0879 0.1666
## Zfext 0.0007 0.0878 0.0885
```

```
reg.abr.gender.Ext<-lm(Zabr~Zmext+Zfext, data=map.join@data)
summary(reg.abr.gender.Ext)
```

```
## 
## Call:
## lm(formula = Zabr ~ Zmext + Zfext, data = map.join@data)
## 
## Residuals:
##      Min       1Q   Median       3Q      Max 
## -1.79018 -0.49148 -0.09971  0.39939  2.98939 
## 
## Coefficients:
##               Estimate Std. Error t value Pr(>|t|)
## (Intercept) -6.304e-18  1.402e-01   0.000    1.000
## Zmext       -2.921e-01  2.215e-01  -1.319    0.194
## Zfext        7.532e-02  2.215e-01   0.340    0.735
## 
## Residual standard error: 0.9914 on 47 degrees of freedom
## Multiple R-squared:  0.05715,    Adjusted R-squared:  0.01703 
## F-statistic: 1.424 on 2 and 47 DF,  p-value: 0.2508
```

```
abr.gender.Ext<-commonalityCoefficients(map.join@data,"Zabr", list("Zmext", "Zfext"))
abr.gender.Ext
```

```
## $CC
##                            Coefficient     % Total
## Unique to Zmext                 0.0349       61.03
## Unique to Zfext                 0.0023        4.06
## Common to Zmext, and Zfext      0.0200       34.91
## Total                           0.0571      100.00
## 
## $CCTotalbyVar
##       Unique Common  Total
## Zmext 0.0349 0.0199 0.0548
## Zfext 0.0023 0.0200 0.0223
```

```
reg.fmp.gender.Ext<-lm(Zfmp~Zmext+Zfext, data=map.join@data)
summary(reg.fmp.gender.Ext)
```

```
## 
## Call:
## lm(formula = Zfmp ~ Zmext + Zfext, data = map.join@data)
## 
## Residuals:
##     Min      1Q  Median      3Q     Max 
## -1.6950 -0.6901 -0.2080  0.5481  3.0390 
## 
## Coefficients:
##               Estimate Std. Error t value Pr(>|t|)
## (Intercept)  5.992e-18  1.388e-01   0.000    1.000
## Zmext       -1.487e-01  2.193e-01  -0.678    0.501
## Zfext       -1.437e-01  2.193e-01  -0.655    0.516
## 
## Residual standard error: 0.9817 on 47 degrees of freedom
## Multiple R-squared:  0.07562,    Adjusted R-squared:  0.03628 
## F-statistic: 1.922 on 2 and 47 DF,  p-value: 0.1576
```

```
fmp.gender.Ext<-commonalityCoefficients(map.join@data,"Zfmp", list("Zmext", "Zfext"))
fmp.gender.Ext
```

```
## $CC
##                            Coefficient     % Total
## Unique to Zmext                 0.0090       11.96
## Unique to Zfext                 0.0084       11.16
## Common to Zmext, and Zfext      0.0581       76.88
## Total                           0.0756      100.00
## 
## $CCTotalbyVar
##       Unique Common  Total
## Zmext 0.0090 0.0582 0.0672
## Zfext 0.0084 0.0582 0.0666
```

### 6.3.2 Agreeableness

```
reg.tfr.gender.Agr<-lm(Ztfr~Zmagr+Zfagr, data=map.join@data)
summary(reg.tfr.gender.Agr)
```

```
## 
## Call:
## lm(formula = Ztfr ~ Zmagr + Zfagr, data = map.join@data)
## 
## Residuals:
##     Min      1Q  Median      3Q     Max 
## -1.8618 -0.5515 -0.1017  0.5251  2.0813 
## 
## Coefficients:
##              Estimate Std. Error t value Pr(>|t|)
## (Intercept) 2.585e-17  1.232e-01   0.000    1.000
## Zmagr       3.424e-01  2.403e-01   1.425    0.161
## Zfagr       1.978e-01  2.403e-01   0.823    0.415
## 
## Residual standard error: 0.871 on 47 degrees of freedom
## Multiple R-squared:  0.2723, Adjusted R-squared:  0.2413 
## F-statistic: 8.792 on 2 and 47 DF,  p-value: 0.0005707
```

```
tfr.gender.Agr<-commonalityCoefficients(map.join@data,"Ztfr", list("Zmagr", "Zfagr"))
tfr.gender.Agr
```

```
## $CC
##                            Coefficient     % Total
## Unique to Zmagr                 0.0314       11.54
## Unique to Zfagr                 0.0105        3.85
## Common to Zmagr, and Zfagr      0.2303       84.60
## Total                           0.2723      100.00
## 
## $CCTotalbyVar
##       Unique Common  Total
## Zmagr 0.0314 0.2304 0.2618
## Zfagr 0.0105 0.2303 0.2408
```

```
reg.alp.gender.Agr<-lm(Zalpha~Zmagr+Zfagr, data=map.join@data)
summary(reg.alp.gender.Agr)
```

```
## 
## Call:
## lm(formula = Zalpha ~ Zmagr + Zfagr, data = map.join@data)
## 
## Residuals:
##     Min      1Q  Median      3Q     Max 
## -2.9709 -0.5109  0.1497  0.4859  2.5788 
## 
## Coefficients:
##               Estimate Std. Error t value Pr(>|t|)
## (Intercept) -1.905e-17  1.435e-01   0.000    1.000
## Zmagr        3.647e-03  2.800e-01   0.013    0.990
## Zfagr        1.067e-01  2.800e-01   0.381    0.705
## 
## Residual standard error: 1.015 on 47 degrees of freedom
## Multiple R-squared:  0.01207,    Adjusted R-squared:  -0.02997 
## F-statistic: 0.2872 on 2 and 47 DF,  p-value: 0.7517
```

```
alp.gender.Agr<-commonalityCoefficients(map.join@data,"Zalpha", list("Zmagr", "Zfagr"))
alp.gender.Agr
```

```
## $CC
##                            Coefficient     % Total
## Unique to Zmagr                 0.0000        0.03
## Unique to Zfagr                 0.0031       25.30
## Common to Zmagr, and Zfagr      0.0090       74.67
## Total                           0.0121      100.00
## 
## $CCTotalbyVar
##       Unique Common  Total
## Zmagr 0.0000  0.009 0.0090
## Zfagr 0.0031  0.009 0.0121
```

```
reg.pea.gender.Agr<-lm(ZSpeak~Zmagr+Zfagr, data=map.join@data)
summary(reg.pea.gender.Agr)
```

```
## 
## Call:
## lm(formula = ZSpeak ~ Zmagr + Zfagr, data = map.join@data)
## 
## Residuals:
##      Min       1Q   Median       3Q      Max 
## -1.92229 -0.66847 -0.04201  0.62531  2.35183 
## 
## Coefficients:
##               Estimate Std. Error t value Pr(>|t|)
## (Intercept)  6.572e-17  1.405e-01   0.000    1.000
## Zmagr        3.862e-01  2.741e-01   1.409    0.165
## Zfagr       -2.134e-01  2.741e-01  -0.779    0.440
## 
## Residual standard error: 0.9933 on 47 degrees of freedom
## Multiple R-squared:  0.05367,    Adjusted R-squared:  0.0134 
## F-statistic: 1.333 on 2 and 47 DF,  p-value: 0.2735
```

```
pea.gender.Agr<-commonalityCoefficients(map.join@data,"ZSpeak", list("Zmagr", "Zfagr"))
pea.gender.Agr
```

```
## $CC
##                            Coefficient     % Total
## Unique to Zmagr                 0.0400       74.50
## Unique to Zfagr                 0.0122       22.75
## Common to Zmagr, and Zfagr      0.0015        2.75
## Total                           0.0537      100.00
## 
## $CCTotalbyVar
##       Unique Common  Total
## Zmagr 0.0400 0.0015 0.0415
## Zfagr 0.0122 0.0015 0.0137
```

```
reg.stp.gender.Agr<-lm(Zstop~Zmagr+Zfagr, data=map.join@data)
summary(reg.stp.gender.Agr)
```

```
## 
## Call:
## lm(formula = Zstop ~ Zmagr + Zfagr, data = map.join@data)
## 
## Residuals:
##     Min      1Q  Median      3Q     Max 
## -3.7107 -0.4958  0.1382  0.5751  1.8329 
## 
## Coefficients:
##               Estimate Std. Error t value Pr(>|t|)
## (Intercept)  1.747e-17  1.442e-01   0.000    1.000
## Zmagr        7.097e-02  2.814e-01   0.252    0.802
## Zfagr       -3.022e-02  2.814e-01  -0.107    0.915
## 
## Residual standard error: 1.02 on 47 degrees of freedom
## Multiple R-squared:  0.00228,    Adjusted R-squared:  -0.04018 
## F-statistic: 0.0537 on 2 and 47 DF,  p-value: 0.9478
```

```
stp.gender.Agr<-commonalityCoefficients(map.join@data,"Zstop", list("Zmagr", "Zfagr"))
stp.gender.Agr
```

```
## $CC
##                            Coefficient     % Total
## Unique to Zmagr                 0.0014       59.21
## Unique to Zfagr                 0.0002       10.74
## Common to Zmagr, and Zfagr      0.0007       30.05
## Total                           0.0023      100.00
## 
## $CCTotalbyVar
##       Unique Common Total
## Zmagr 0.0014  6e-04 2e-03
## Zfagr 0.0002  7e-04 9e-04
```

```
reg.afb.gender.Agr<-lm(ZSafb~Zmagr+Zfagr, data=map.join@data)
summary(reg.afb.gender.Agr)
```

```
## 
## Call:
## lm(formula = ZSafb ~ Zmagr + Zfagr, data = map.join@data)
## 
## Residuals:
##     Min      1Q  Median      3Q     Max 
## -2.6513 -0.4773  0.1590  0.6001  1.9618 
## 
## Coefficients:
##               Estimate Std. Error t value Pr(>|t|)
## (Intercept) -2.771e-17  1.429e-01   0.000    1.000
## Zmagr       -3.876e-02  2.788e-01  -0.139    0.890
## Zfagr       -1.093e-01  2.788e-01  -0.392    0.697
## 
## Residual standard error: 1.01 on 47 degrees of freedom
## Multiple R-squared:  0.02069,    Adjusted R-squared:  -0.02098 
## F-statistic: 0.4966 on 2 and 47 DF,  p-value: 0.6118
```

```
afb.gender.Agr<-commonalityCoefficients(map.join@data,"ZSafb", list("Zmagr", "Zfagr"))
afb.gender.Agr
```

```
## $CC
##                            Coefficient     % Total
## Unique to Zmagr                 0.0004        1.95
## Unique to Zfagr                 0.0032       15.47
## Common to Zmagr, and Zfagr      0.0171       82.58
## Total                           0.0207      100.00
## 
## $CCTotalbyVar
##       Unique Common  Total
## Zmagr 0.0004 0.0171 0.0175
## Zfagr 0.0032 0.0171 0.0203
```

```
reg.afm.gender.Agr<-lm(Zafm~Zmagr+Zfagr, data=map.join@data)
summary(reg.afm.gender.Agr)
```

```
## 
## Call:
## lm(formula = Zafm ~ Zmagr + Zfagr, data = map.join@data)
## 
## Residuals:
##      Min       1Q   Median       3Q      Max 
## -2.78546 -0.63013  0.02972  0.66642  2.38402 
## 
## Coefficients:
##               Estimate Std. Error t value Pr(>|t|)
## (Intercept)  1.459e-17  1.403e-01   0.000    1.000
## Zmagr       -3.668e-01  2.737e-01  -1.340    0.187
## Zfagr        1.729e-01  2.737e-01   0.632    0.531
## 
## Residual standard error: 0.9921 on 47 degrees of freedom
## Multiple R-squared:  0.05592,    Adjusted R-squared:  0.01575 
## F-statistic: 1.392 on 2 and 47 DF,  p-value: 0.2586
```

```
afm.gender.Agr<-commonalityCoefficients(map.join@data,"Zafm", list("Zmagr", "Zfagr"))
afm.gender.Agr
```

```
## $CC
##                            Coefficient     % Total
## Unique to Zmagr                 0.0361       64.50
## Unique to Zfagr                 0.0080       14.34
## Common to Zmagr, and Zfagr      0.0118       21.16
## Total                           0.0559      100.00
## 
## $CCTotalbyVar
##       Unique Common  Total
## Zmagr 0.0361 0.0118 0.0479
## Zfagr 0.0080 0.0119 0.0199
```

```
reg.nvm.gender.Agr<-lm(ZSnvm~Zmagr+Zfagr, data=map.join@data)
summary(reg.nvm.gender.Agr)
```

```
## 
## Call:
## lm(formula = ZSnvm ~ Zmagr + Zfagr, data = map.join@data)
## 
## Residuals:
##     Min      1Q  Median      3Q     Max 
## -2.0284 -0.6429 -0.2543  0.5925  2.8122 
## 
## Coefficients:
##               Estimate Std. Error t value Pr(>|t|)
## (Intercept) -1.063e-17  1.401e-01   0.000    1.000
## Zmagr       -2.269e-01  2.734e-01  -0.830    0.411
## Zfagr        4.050e-01  2.734e-01   1.481    0.145
## 
## Residual standard error: 0.9909 on 47 degrees of freedom
## Multiple R-squared:  0.05828,    Adjusted R-squared:  0.0182 
## F-statistic: 1.454 on 2 and 47 DF,  p-value: 0.2439
```

```
nvm.gender.Agr<-commonalityCoefficients(map.join@data,"ZSnvm", list("Zmagr", "Zfagr"))
nvm.gender.Agr
```

```
## $CC
##                            Coefficient     % Total
## Unique to Zmagr                 0.0138       23.68
## Unique to Zfagr                 0.0440       75.45
## Common to Zmagr, and Zfagr      0.0005        0.87
## Total                           0.0583      100.00
## 
## $CCTotalbyVar
##       Unique Common  Total
## Zmagr 0.0138  5e-04 0.0143
## Zfagr 0.0440  5e-04 0.0445
```

```
reg.div.gender.Agr<-lm(ZSdiv~Zmagr+Zfagr, data=map.join@data)
summary(reg.div.gender.Agr)
```

```
## 
## Call:
## lm(formula = ZSdiv ~ Zmagr + Zfagr, data = map.join@data)
## 
## Residuals:
##     Min      1Q  Median      3Q     Max 
## -1.6212 -0.7385 -0.1705  0.7880  1.7720 
## 
## Coefficients:
##               Estimate Std. Error t value Pr(>|t|)
## (Intercept) -3.859e-17  1.415e-01   0.000    1.000
## Zmagr        2.300e-01  2.760e-01   0.833    0.409
## Zfagr       -3.582e-01  2.760e-01  -1.298    0.201
## 
## Residual standard error: 1 on 47 degrees of freedom
## Multiple R-squared:  0.04024,    Adjusted R-squared:  -0.0006057 
## F-statistic: 0.9852 on 2 and 47 DF,  p-value: 0.381
```

```
div.gender.Agr<-commonalityCoefficients(map.join@data,"ZSdiv", list("Zmagr", "Zfagr"))
div.gender.Agr
```

```
## $CC
##                            Coefficient     % Total
## Unique to Zmagr                 0.0142       35.24
## Unique to Zfagr                 0.0344       85.47
## Common to Zmagr, and Zfagr     -0.0083      -20.71
## Total                           0.0402      100.00
## 
## $CCTotalbyVar
##       Unique  Common  Total
## Zmagr 0.0142 -0.0084 0.0058
## Zfagr 0.0344 -0.0083 0.0261
```

```
reg.coh.gender.Agr<-lm(Zcoh~Zmagr+Zfagr, data=map.join@data)
summary(reg.coh.gender.Agr)
```

```
## 
## Call:
## lm(formula = Zcoh ~ Zmagr + Zfagr, data = map.join@data)
## 
## Residuals:
##      Min       1Q   Median       3Q      Max 
## -2.00427 -0.57268  0.03631  0.52397  2.02943 
## 
## Coefficients:
##               Estimate Std. Error t value Pr(>|t|)
## (Intercept) -1.551e-17  1.307e-01   0.000    1.000
## Zmagr       -2.150e-01  2.551e-01  -0.843    0.404
## Zfagr       -2.260e-01  2.551e-01  -0.886    0.380
## 
## Residual standard error: 0.9244 on 47 degrees of freedom
## Multiple R-squared:  0.1804, Adjusted R-squared:  0.1455 
## F-statistic: 5.172 on 2 and 47 DF,  p-value: 0.009326
```

```
coh.gender.Agr<-commonalityCoefficients(map.join@data,"Zcoh", list("Zmagr", "Zfagr"))
coh.gender.Agr
```

```
## $CC
##                            Coefficient     % Total
## Unique to Zmagr                 0.0124        6.87
## Unique to Zfagr                 0.0137        7.59
## Common to Zmagr, and Zfagr      0.1543       85.54
## Total                           0.1804      100.00
## 
## $CCTotalbyVar
##       Unique Common  Total
## Zmagr 0.0124 0.1543 0.1667
## Zfagr 0.0137 0.1543 0.1680
```

```
reg.nmf.gender.Agr<-lm(Znmf~Zmagr+Zfagr, data=map.join@data)
summary(reg.nmf.gender.Agr)
```

```
## 
## Call:
## lm(formula = Znmf ~ Zmagr + Zfagr, data = map.join@data)
## 
## Residuals:
##      Min       1Q   Median       3Q      Max 
## -2.57122 -0.62122  0.05582  0.60668  1.95710 
## 
## Coefficients:
##               Estimate Std. Error t value Pr(>|t|)   
## (Intercept)  5.186e-18  1.310e-01   0.000  1.00000   
## Zmagr       -8.139e-01  2.555e-01  -3.186  0.00257 **
## Zfagr        6.939e-01  2.555e-01   2.716  0.00922 **
## ---
## Signif. codes:  0 '***' 0.001 '**' 0.01 '*' 0.05 '.' 0.1 ' ' 1
## 
## Residual standard error: 0.926 on 47 degrees of freedom
## Multiple R-squared:  0.1776, Adjusted R-squared:  0.1426 
## F-statistic: 5.074 on 2 and 47 DF,  p-value: 0.01011
```

```
nmf.gender.Agr<-commonalityCoefficients(map.join@data,"Znmf", list("Zmagr", "Zfagr"))
nmf.gender.Agr
```

```
## $CC
##                            Coefficient     % Total
## Unique to Zmagr                 0.1776      100.00
## Unique to Zfagr                 0.1291       72.68
## Common to Zmagr, and Zfagr     -0.1291      -72.68
## Total                           0.1776      100.00
## 
## $CCTotalbyVar
##       Unique  Common  Total
## Zmagr 0.1776 -0.1291 0.0485
## Zfagr 0.1291 -0.1291 0.0000
```

```
reg.uni.gender.Agr<-lm(Zuni~Zmagr+Zfagr, data=map.join@data)
summary(reg.uni.gender.Agr)
```

```
## 
## Call:
## lm(formula = Zuni ~ Zmagr + Zfagr, data = map.join@data)
## 
## Residuals:
##      Min       1Q   Median       3Q      Max 
## -2.67614 -0.60610 -0.02227  0.56270  2.79829 
## 
## Coefficients:
##               Estimate Std. Error t value Pr(>|t|)
## (Intercept) -5.643e-18  1.425e-01   0.000    1.000
## Zmagr       -2.858e-01  2.780e-01  -1.028    0.309
## Zfagr        1.761e-01  2.780e-01   0.634    0.529
## 
## Residual standard error: 1.007 on 47 degrees of freedom
## Multiple R-squared:  0.02658,    Adjusted R-squared:  -0.01484 
## F-statistic: 0.6417 on 2 and 47 DF,  p-value: 0.531
```

```
uni.gender.Agr<-commonalityCoefficients(map.join@data,"Zuni", list("Zmagr", "Zfagr"))
uni.gender.Agr
```

```
## $CC
##                            Coefficient     % Total
## Unique to Zmagr                 0.0219       82.38
## Unique to Zfagr                 0.0083       31.27
## Common to Zmagr, and Zfagr     -0.0036      -13.66
## Total                           0.0266      100.00
## 
## $CCTotalbyVar
##       Unique  Common  Total
## Zmagr 0.0219 -0.0036 0.0183
## Zfagr 0.0083 -0.0036 0.0047
```

```
reg.abr.gender.Agr<-lm(Zabr~Zmagr+Zfagr, data=map.join@data)
summary(reg.abr.gender.Agr)
```

```
## 
## Call:
## lm(formula = Zabr ~ Zmagr + Zfagr, data = map.join@data)
## 
## Residuals:
##      Min       1Q   Median       3Q      Max 
## -1.66998 -0.60425 -0.08732  0.51248  2.86426 
## 
## Coefficients:
##               Estimate Std. Error t value Pr(>|t|)
## (Intercept) -1.674e-17  1.392e-01   0.000    1.000
## Zmagr       -2.048e-01  2.715e-01  -0.754    0.454
## Zfagr       -6.950e-02  2.715e-01  -0.256    0.799
## 
## Residual standard error: 0.9841 on 47 degrees of freedom
## Multiple R-squared:  0.07113,    Adjusted R-squared:  0.03161 
## F-statistic:   1.8 on 2 and 47 DF,  p-value: 0.1766
```

```
abr.gender.Agr<-commonalityCoefficients(map.join@data,"Zabr", list("Zmagr", "Zfagr"))
abr.gender.Agr
```

```
## $CC
##                            Coefficient     % Total
## Unique to Zmagr                 0.0112       15.81
## Unique to Zfagr                 0.0013        1.82
## Common to Zmagr, and Zfagr      0.0586       82.37
## Total                           0.0711      100.00
## 
## $CCTotalbyVar
##       Unique Common  Total
## Zmagr 0.0112 0.0586 0.0698
## Zfagr 0.0013 0.0586 0.0599
```

```
reg.fmp.gender.Agr<-lm(Zfmp~Zmagr+Zfagr, data=map.join@data)
summary(reg.fmp.gender.Agr)
```

```
## 
## Call:
## lm(formula = Zfmp ~ Zmagr + Zfagr, data = map.join@data)
## 
## Residuals:
##     Min      1Q  Median      3Q     Max 
## -1.2702 -0.8641 -0.1346  0.6378  3.0920 
## 
## Coefficients:
##               Estimate Std. Error t value Pr(>|t|)
## (Intercept)  1.926e-17  1.426e-01   0.000    1.000
## Zmagr       -1.924e-01  2.783e-01  -0.692    0.493
## Zfagr        2.849e-01  2.783e-01   1.024    0.311
## 
## Residual standard error: 1.009 on 47 degrees of freedom
## Multiple R-squared:  0.0244, Adjusted R-squared:  -0.01712 
## F-statistic: 0.5877 on 2 and 47 DF,  p-value: 0.5596
```

```
fmp.gender.Agr<-commonalityCoefficients(map.join@data,"Zfmp", list("Zmagr", "Zfagr"))
fmp.gender.Agr
```

```
## $CC
##                            Coefficient     % Total
## Unique to Zmagr                 0.0099       40.68
## Unique to Zfagr                 0.0218       89.20
## Common to Zmagr, and Zfagr     -0.0073      -29.88
## Total                           0.0244      100.00
## 
## $CCTotalbyVar
##       Unique  Common  Total
## Zmagr 0.0099 -0.0073 0.0026
## Zfagr 0.0218 -0.0073 0.0145
```

### 6.3.3 Conscientiousness

```
reg.tfr.gender.Cns<-lm(Ztfr~Zmcns+Zfcns, data=map.join@data)
summary(reg.tfr.gender.Cns)
```

```
## 
## Call:
## lm(formula = Ztfr ~ Zmcns + Zfcns, data = map.join@data)
## 
## Residuals:
##     Min      1Q  Median      3Q     Max 
## -2.1939 -0.5352 -0.2518  0.5797  2.1170 
## 
## Coefficients:
##              Estimate Std. Error t value Pr(>|t|)  
## (Intercept) 2.419e-17  1.299e-01   0.000   1.0000  
## Zmcns       3.011e-01  1.670e-01   1.803   0.0777 .
## Zfcns       1.810e-01  1.670e-01   1.084   0.2839  
## ---
## Signif. codes:  0 '***' 0.001 '**' 0.01 '*' 0.05 '.' 0.1 ' ' 1
## 
## Residual standard error: 0.9185 on 47 degrees of freedom
## Multiple R-squared:  0.1909, Adjusted R-squared:  0.1564 
## F-statistic: 5.543 on 2 and 47 DF,  p-value: 0.006896
```

```
tfr.gender.Cns<-commonalityCoefficients(map.join@data,"Ztfr", list("Zmcns", "Zfcns"))
tfr.gender.Cns
```

```
## $CC
##                            Coefficient     % Total
## Unique to Zmcns                 0.0560       29.34
## Unique to Zfcns                 0.0202       10.60
## Common to Zmcns, and Zfcns      0.1146       60.06
## Total                           0.1909      100.00
## 
## $CCTotalbyVar
##       Unique Common  Total
## Zmcns 0.0560 0.1146 0.1706
## Zfcns 0.0202 0.1147 0.1349
```

```
reg.alp.gender.Cns<-lm(Zalpha~Zmcns+Zfcns, data=map.join@data)
summary(reg.alp.gender.Cns)
```

```
## 
## Call:
## lm(formula = Zalpha ~ Zmcns + Zfcns, data = map.join@data)
## 
## Residuals:
##     Min      1Q  Median      3Q     Max 
## -2.9034 -0.4978  0.1291  0.4893  2.5645 
## 
## Coefficients:
##               Estimate Std. Error t value Pr(>|t|)
## (Intercept) -1.714e-17  1.431e-01   0.000    1.000
## Zmcns        1.138e-01  1.839e-01   0.619    0.539
## Zfcns        2.968e-02  1.839e-01   0.161    0.873
## 
## Residual standard error: 1.012 on 47 degrees of freedom
## Multiple R-squared:  0.01801,    Adjusted R-squared:  -0.02378 
## F-statistic: 0.431 on 2 and 47 DF,  p-value: 0.6524
```

```
alp.gender.Cns<-commonalityCoefficients(map.join@data,"Zalpha", list("Zmcns", "Zfcns"))
alp.gender.Cns
```

```
## $CC
##                            Coefficient     % Total
## Unique to Zmcns                 0.0080       44.41
## Unique to Zfcns                 0.0005        3.02
## Common to Zmcns, and Zfcns      0.0095       52.57
## Total                           0.0180      100.00
## 
## $CCTotalbyVar
##       Unique Common  Total
## Zmcns  8e-03 0.0095 0.0175
## Zfcns  5e-04 0.0095 0.0100
```

```
reg.pea.gender.Cns<-lm(ZSpeak~Zmcns+Zfcns, data=map.join@data)
summary(reg.pea.gender.Cns)
```

```
## 
## Call:
## lm(formula = ZSpeak ~ Zmcns + Zfcns, data = map.join@data)
## 
## Residuals:
##     Min      1Q  Median      3Q     Max 
## -1.7786 -0.7063 -0.1437  0.7175  2.5964 
## 
## Coefficients:
##               Estimate Std. Error t value Pr(>|t|)
## (Intercept)  7.389e-17  1.406e-01   0.000    1.000
## Zmcns        2.746e-01  1.808e-01   1.519    0.135
## Zfcns       -2.391e-01  1.808e-01  -1.323    0.192
## 
## Residual standard error: 0.9945 on 47 degrees of freedom
## Multiple R-squared:  0.05137,    Adjusted R-squared:  0.01101 
## F-statistic: 1.273 on 2 and 47 DF,  p-value: 0.2896
```

```
pea.gender.Cns<-commonalityCoefficients(map.join@data,"ZSpeak", list("Zmcns", "Zfcns"))
pea.gender.Cns
```

```
## $CC
##                            Coefficient     % Total
## Unique to Zmcns                 0.0466       90.66
## Unique to Zfcns                 0.0353       68.73
## Common to Zmcns, and Zfcns     -0.0305      -59.39
## Total                           0.0514      100.00
## 
## $CCTotalbyVar
##       Unique  Common  Total
## Zmcns 0.0466 -0.0305 0.0161
## Zfcns 0.0353 -0.0305 0.0048
```

```
reg.stp.gender.Cns<-lm(Zstop~Zmcns+Zfcns, data=map.join@data)
summary(reg.stp.gender.Cns)
```

```
## 
## Call:
## lm(formula = Zstop ~ Zmcns + Zfcns, data = map.join@data)
## 
## Residuals:
##     Min      1Q  Median      3Q     Max 
## -3.4189 -0.6024  0.1406  0.5561  1.8235 
## 
## Coefficients:
##               Estimate Std. Error t value Pr(>|t|)
## (Intercept)  2.977e-17  1.414e-01   0.000    1.000
## Zmcns        2.226e-01  1.817e-01   1.225    0.227
## Zfcns       -3.199e-02  1.817e-01  -0.176    0.861
## 
## Residual standard error: 0.9995 on 47 degrees of freedom
## Multiple R-squared:  0.04177,    Adjusted R-squared:  0.000998 
## F-statistic: 1.024 on 2 and 47 DF,  p-value: 0.3669
```

```
stp.gender.Cns<-commonalityCoefficients(map.join@data,"Zstop", list("Zmcns", "Zfcns"))
stp.gender.Cns
```

```
## $CC
##                            Coefficient     % Total
## Unique to Zmcns                 0.0306       73.26
## Unique to Zfcns                 0.0006        1.51
## Common to Zmcns, and Zfcns      0.0105       25.22
## Total                           0.0418      100.00
## 
## $CCTotalbyVar
##       Unique Common  Total
## Zmcns 0.0306 0.0105 0.0411
## Zfcns 0.0006 0.0106 0.0112
```

```
reg.afb.gender.Cns<-lm(ZSafb~Zmcns+Zfcns, data=map.join@data)
summary(reg.afb.gender.Cns)
```

```
## 
## Call:
## lm(formula = ZSafb ~ Zmcns + Zfcns, data = map.join@data)
## 
## Residuals:
##     Min      1Q  Median      3Q     Max 
## -2.2435 -0.6256  0.1342  0.6530  2.1136 
## 
## Coefficients:
##               Estimate Std. Error t value Pr(>|t|)
## (Intercept) -3.790e-17  1.386e-01   0.000    1.000
## Zmcns       -2.742e-01  1.781e-01  -1.539    0.130
## Zfcns       -1.096e-02  1.781e-01  -0.062    0.951
## 
## Residual standard error: 0.9799 on 47 degrees of freedom
## Multiple R-squared:  0.07903,    Adjusted R-squared:  0.03984 
## F-statistic: 2.017 on 2 and 47 DF,  p-value: 0.1445
```

```
afb.gender.Cns<-commonalityCoefficients(map.join@data,"ZSafb", list("Zmcns", "Zfcns"))
afb.gender.Cns
```

```
## $CC
##                            Coefficient     % Total
## Unique to Zmcns                 0.0464       58.75
## Unique to Zfcns                 0.0001        0.09
## Common to Zmcns, and Zfcns      0.0325       41.15
## Total                           0.0790      100.00
## 
## $CCTotalbyVar
##       Unique Common  Total
## Zmcns 0.0464 0.0326 0.0790
## Zfcns 0.0001 0.0325 0.0326
```

```
reg.afm.gender.Cns<-lm(Zafm~Zmcns+Zfcns, data=map.join@data)
summary(reg.afm.gender.Cns)
```

```
## 
## Call:
## lm(formula = Zafm ~ Zmcns + Zfcns, data = map.join@data)
## 
## Residuals:
##     Min      1Q  Median      3Q     Max 
## -2.7705 -0.4440  0.1202  0.6163  1.9916 
## 
## Coefficients:
##               Estimate Std. Error t value Pr(>|t|)  
## (Intercept) -2.262e-18  1.331e-01   0.000   1.0000  
## Zmcns       -3.118e-01  1.712e-01  -1.822   0.0749 .
## Zfcns       -1.067e-01  1.712e-01  -0.624   0.5359  
## ---
## Signif. codes:  0 '***' 0.001 '**' 0.01 '*' 0.05 '.' 0.1 ' ' 1
## 
## Residual standard error: 0.9415 on 47 degrees of freedom
## Multiple R-squared:  0.1498, Adjusted R-squared:  0.1136 
## F-statistic: 4.139 on 2 and 47 DF,  p-value: 0.02209
```

```
afm.gender.Cns<-commonalityCoefficients(map.join@data,"Zafm", list("Zmcns", "Zfcns"))
afm.gender.Cns
```

```
## $CC
[truncated: 227,963 more chars]
