# Supplementary material for: Geographic Variation in Personality is Associated With Fertility Across the United States
Source: Personal Sci. Author manuscript; Available in PMC 2024 Aug 30. (PMC11364352; doi:10.5964/ps.7275)
Supplement: Personality and Fertility Supplement [file NIHMS1912217-supplement-Personality_and_Fertility_Supplement.docx]

**Supplemental Materials**

**Geographic Variation in Personality is Associated with Fertility across the United States**

<https://osf.io/f7c89/?view_only=5f573ef84a3b475d84ad7e5bef1496a4>

In the online supplement, we provide more in-depth description of our data preparation and empirical results.

**Data Preparation: Ensuring Spatial Autocorrelation Does Not Bias Results**

When analyzing spatial data, it is important to recognize that the observed regions may not be randomly drawn, a critical assumption of most statistical tests. When observations are not independent, it is likely that standard errors are biased downwards. Because we were not interested in modeling spatial processes (e.g., spillover), our goal was to use the simplest technique for which we do not violate assumptions. If the observations displayed spatial autocorrelation, then we would need to use spatial regression techniques to avoid bias. However, if the observations displayed no spatial autocorrelation, then standard correlation techniques would be appropriate because we do not violate any assumptions.

We used Moran’s I (Moran, 1950) to assess spatial autocorrelation. Positive Moran’s I indicates that high values tend to cluster near high values and low values tend to cluster near low values. Negative Moran’s I indicates that observations are dispersed, with high values near low values and vice versa. Nonsignificant Moran’s I indicates that observations are distributed randomly across geographic space. To calculate Moran’s I, we used a queen’s case contiguity weights matrix and TIGER shapefiles from the U.S. Census Bureau^^[[1]](#footnote-1)^^. We used the spdep package in R to estimate Moran’s I (Bivand, 2014). We allowed for geographic islands (e.g., Alaska and Hawaii). Generally, we observed positive spatial autocorrelation for nearly all study variables.

We were primarily interested in the association between state-level personality and fertility, holding known correlates constant. Therefore, we computed residuals from linear models in which each of the primary study variables (i.e., personality and fertility) were regressed on the established correlates. The residual variance from these models is reported in Table S1. The established correlates accounted for approximately half of the variance of each personality and fertility variable, on average. This indicates that sociodemographic characteristics and values are important explanatory variables for both state-level personality and fertility, but substantial unexplained variation remains. Because we were not interested in the regression parameters from these models (which may have too small of standard errors due to spatial autocorrelation), we used ordinary least squares regression.

We then tested for spatial autocorrelation among the residuals. For most variables, controlling for sociodemographic characteristics and values reduced spatial autocorrelation to nonsignificance. Remaining spatial autocorrelation was observed for neuroticism, female neuroticism, peak fertility, age at first birth, percent never married, and percent divorce. To avoid spatial autocorrelation obscuring associations between personality and fertility, we used spatial regression models, which take into account the spatial structure of the data (LeSage & Pace, 2009). These models include spatial lag models, where the spatial lag of the dependent measure is included as a predictor, spatial error models, where errors are allowed to correlate based on geographical relations, and models that combine both (i.e., SARAR). Each model is designed to control for the spatial structure of the data and provide unbiased parameter estimates. Spatially oriented regression diagnostics can detect sources of geographic error and guide the selection of the appropriate model (Anselin et al., 1996). Based on these diagnostic tools, we used a spatial error model to regress female neuroticism, peak fertility, age at first birth, percent never married, and percent divorce on the sociodemographic characteristics and value controls, and we used a spatial lag model to regress neuroticism on the controls. Residuals from these models did not show spatial autocorrelation and were saved for analysis.

**Subgroup Personality and Fertility**

Table S2 reports the results of a gender-based analysis and age-based analysis for extraversion, agreeableness, conscientiousness, neuroticism, and openness to experience.

*Extraversion.* The general-level association with peak fertility was largely driven by common variance shared by male and female extraversion. On the other hand, the general-level association with stopping behavior was driven, in part, by common variance, but the unique effect of female extraversion accounted for a larger proportion of variance. For the general association with unintended pregnancy, this pattern was reversed. Roughly equivalent proportions of variance were accounted for by the unique effect of male extraversion and the common effect. Other minor associations were primarily driven by common variance, but there was some evidence of a male effect for divorce. An additional suppression effect emerged in the prediction of the non-marital fertility rate. Higher male extraversion was associated with lower non-marital fertility, and higher female extraversion was associated with higher non-marital fertility. The negative commonality effect can be interpreted as the amount the unique effects are increased by including the suppressor variable (see Nimon, 2010, p. 714-16). Significant predictive power was gained by including both male and female indicators of extraversion.

Further, young extraversion (< 30 years) was primarily associated with fertility. The general-level associations with peak fertility, stopping behavior, and percent unintended pregnancy had similar signs for age < 30 years extraversion, and the effect was primarily driven by unique effects of this age category. For each outcome, some suppression was observed, but particularly so for unintended pregnancy. Additional, small suppression effects were observed for abortion rate, family planning expenditures, and non-marital fertility with younger extraversion predicting lower levels of these variables. On the whole, common effects were rather small, and unique effects (plus suppression) tended to explain the majority of the variance in fertility outcomes.

*Agreeableness.* The general-level associations with the total fertility rate and prevalence of cohabitation were primarily driven by common effects, but the association with divorce differed by gender. Male agreeableness was associated with higher rates of divorce, and female agreeableness was associated with lower rates of divorce. The majority of variance explained was due to unique effects, but neither regression parameter was significant due to inflated standard errors. Similar to extraversion, a suppression effect emerged such that male agreeableness tended to be associated with lower non-marital fertility, and female agreeableness tended to be associated with higher non-marital fertility.

Sizeable common effects across age were observed for total fertility rate, age at first birth, and percent cohabit. The remaining effects were primarily due to unique effects of age ≥ 30 population with small evidence of suppression. Higher levels of age ≥ 30 agreeableness were associated with a higher total fertility rate and lower age at first birth, age at first marriage, percent never married, abortion rate, non-marital fertility rate, and unintended pregnancy. The association between agreeableness and conventional patterns of fertility was primarily due to levels of age ≥ 30 agreeableness.

*Conscientiousness*. Total fertility rate, which displayed a general-level association, was primarily associated with common variance, but there were also small unique effects for both male and female conscientiousness. Interestingly, it appears that the majority of the general-level associations with the fertility context (i.e., cohabitation, abortion, and unintended pregnancy) were driven by sizable common effects with additional unique male effects. This contrasts with near zero standardized regression coefficients and no unique effects for female conscientiousness.

Age < 30 conscientiousness tended to reflect the general-level associations to a stronger degree than the age ≥ 30 conscientiousness, which had few associations with fertility. Higher age < 30 conscientiousness was associated with conventional fertility practices such as a higher total fertility rate and lower age at first birth, percent cohabit, abortion rate, non-marital fertility rate, and unintended pregnancy. Age < 30 conscientiousness was also associated with later peak fertility and more stopping behavior. Age ≥ 30 conscientiousness predicted less stopping behavior and a smaller never married population. The majority of the effects were unique with only one modest suppression effect for stopping behavior. Common variance across aged conscientiousness explained variance in the total fertility rate and percent cohabitation.

*Neuroticism.* The general-level association with the total fertility rate was primarily driven by common effects with a sizeable unique female effect as well. General-level associations with age at first birth and marriage were primarily associated with common effects, but these outcomes were additionally associated with sizeable unique male effects. The general-level association with the abortion rate was primarily associated with male neuroticism with essentially no common effect. Three suppression effects were observed for stopping behavior, family planning expenditures, and unintended pregnancy. Higher male neuroticism was associated with less stopping behavior and more family planning expenditures and unintended pregnancies. Female neuroticism displayed the opposite pattern. Female neuroticism was also significantly predictive of later peak fertility.

The general-level associations with total fertility, age at first birth and marriage, and abortion were primarily associated with age < 30 neuroticism. Age < 30 neuroticism also predicted less stopping behavior and higher levels of never married individuals, non-marital fertility, and unintended pregnancies. The effects for stopping behavior, non-marital fertility, and unintended pregnancy were driven by moderate suppression effects. Age ≥ 30 neuroticism tended to only predict fertility outcomes in the presence of a suppression effect. Sizable common effects were found for total fertility rate, age at first birth, and age at first marriage.

*Openness.* The general-level associations with the total fertility rate and cohabitation rate were driven relatively equally by common effects and unique female openness effects. The general-level divorce association displayed the opposite pattern with a relatively equal distribution of common effects and male openness effects. General-level associations with age at first birth and marriage displayed suppression effects in which male openness was associated with younger ages, and female openness was associated with later ages. Both were primarily driven by unique female openness effects. Suppression effects also led to novel male driven associations with peak fertility and percent never married. In both cases, higher male openness was associated with lower levels of these variables.

The general-level associations with total fertility, age at first birth and marriage, and cohabitation were primarily driven by common effects or unique effects of age ≥ 30 openness. Age ≥ 30 openness also predicted later peak fertility, greater never married population, more family planning expenditures, and high non-marital fertility in the context of suppression effects. Age < 30 openness predicted higher rates of divorce primarily due to a unique effect.

**References**

Anselin, L., Bera, A. K., Floras, R., & Yoon, M. J. (1996). Simple diagnostic tests for spatial dependence. *Regional Science and Urban Economics, 26*, 77-104.

Bivand, R. (2014). *Spdep: Spatial Dependence: Weighting Schemes, Statistics and Models. R package version 0.5-77*. Available at http://cran.r-project.org/web/packages/spdep/spdep.pdf.

LeSage, J. & Pace, R. K. (2009). *Introduction to Spatial Econometrics*. Taylor & Francis.

Moran, P. A. P. (1950). Notes on continuous stochastic phenomena. *Biometrika, 37*, 17-23 (1950).

Nimon, K., Henson, R. K., & Gates, M. S. (2010). Revisiting interpretation of canonical correlation analysis: A tutorial and demonstration of canonical commonality analysis. *Multivariate Behavioral Research, 45*(4), 702-724.

| **Table S1 \| Descriptive statistics of study variables** | | | | | | |
| --- | --- | --- | --- | --- | --- | --- |
| Variable | Mean (SD) | Range | Year | Moran’s I | Residual Variance | Residual Moran’s I |
| *Personality* |  |  |  |  |  |  |
| Extraversion | 0.00 (0.04) | -0.13-0.08 | 1999-2005 | .22 * | .71 | .14 |
| Agreeableness | 0.00 (0.06) | -0.15-0.11 | 1999-2005 | .25 ** | .75 | .12 |
| Conscientiousness | 0.00 (0.05) | -0.11-0.09 | 1999-2005 | .34 *** | .66 | .14 |
| Neuroticism | 0.00 (0.05) | -0.08-0.14 | 1999-2005 | .52 *** | .43 | .19 * |
| Openness to experience | -0.02 (0.06) | -0.17-0.09 | 1999-2005 | .35 *** | .47 | .15 |
| *Male Personality* |  |  |  |  |  |  |
| Extraversion | 0.09 (0.03) | 0.00- 0.17 | 1999-2005 | .24 ** | .69 | .16 |
| Agreeableness | 0.47 (0.03) | 0.39-  0.52 | 1999-2005 | .09 | .77 | .03 |
| Conscientiousness | 0.32 (0.03) | 0.26-  0.38 | 1999-2005 | .40 *** | .50 | .00 |
| Neuroticism | -0.29 (0.03) | -0.34-  -0.22 | 1999-2005 | .50 *** | .53 | .15 |
| Openness to experience | 0.43 (0.03) | 0.37- 0.51 | 1999-2005 | .29 ** | .47 | .00 |
| *Female Personality* |  |  |  |  |  |  |
| Extraversion | 0.21 (0.02) | 0.14- 0.26 | 1999-2005 | .20 * | .77 | .10 |
| Agreeableness | 0.58 (0.03) | 0.50- 0.64 | 1999-2005 | .31 *** | .72 | .14 |
| Conscientiousness | 0.41 (0.03) | 0.34- 0.46 | 1999-2005 | .31 *** | .62 | .13 |
| Neuroticism | 0.02 (0.03) | -0.04- 0.12 | 1999-2005 | .51 *** | .41 | .18 * |
| Openness to experience | 0.36 (0.04) | 0.27- 0.42 | 1999-2005 | .36 *** | .52 | .16 |
| *Less than 30 Personality* | |  |  |  |  |  |
| Extraversion | -0.01 (0.04) | -0.14- 0.09 | 1999-2005 | .23 ** | .63 | .12 |
| Agreeableness | 0.01 (0.07) | -0.16- 0.15 | 1999-2005 | .24 ** | .75 | .14 |
| Conscientiousness | 0.02 (0.06) | -0.14- 0.13 | 1999-2005 | .28 ** | .58 | .09 |
| Neuroticism | 0.00 (0.05) | -0.09- 0.12 | 1999-2005 | .59 *** | .41 | .17 |
| Openness to experience | -0.02 (0.06) | -0.17- 0.11 | 1999-2005 | .30 ** | .54 | .14 |
| *Greater than 30 Personality* | |  |  |  |  |  |
| Extraversion | 0.01 (0.03) | -0.09- 0.07 | 1999-2005 | .10 | .80 | .10 |
| Agreeableness | -0.03 (0.04) | -0.14- 0.06 | 1999-2005 | .11 | .76 | -.06 |
| Conscientiousness | -0.05 (0.04) | -0.14- 0.02 | 1999-2005 | .21 * | .80 | .15 |
| Neuroticism | 0.02 (0.06) | -0.12- 0.17 | 1999-2005 | .22 * | .55 | .10 |
| Openness to experience | -0.03 (0.08) | -0.18- 0.13 | 1999-2005 | .51 *** | .34 | .15 |
| *Gender Difference Personality* | |  |  |  |  |  |
| Extraversion | -0.12 (0.02) | -0.17-  -0.09 | 1999-2005 | -.02 | .83 | -.08 |
| Agreeableness | -0.11 (0.02) | -0.14-  -0.07 | 1999-2005 | .07 | .71 | -.08 |
| Conscientiousness | -0.08 (0.02) | -0.14-  -0.03 | 1999-2005 | .12 | .65 | -.01 |
| Neuroticism | -0.31 (0.02) | -0.35-  -0.23 | 1999-2005 | .15 | .72 | .07 |
| Openness to experience | 0.08 (0.02) | 0.01- 0.14 | 1999-2005 | .34 *** | .62 | -.06 |
| *Age Difference Personality* | |  |  |  |  |  |
| Extraversion | -0.02 (0.04) | -0.10- 0.05 | 1999-2005 | .18 * | .48 | -.05 |
| Agreeableness | 0.04 (0.05) | -0.09- 0.17 | 1999-2005 | .15 | .77 | .10 |
| Conscientiousness | 0.07 (0.06) | -0.11- 0.21 | 1999-2005 | .05 | .51 | -.05 |
| Neuroticism | -0.02 (0.04) | -0.11- 0.09 | 1999-2005 | .15 | .68 | -.08 |
| Openness to experience | 0.01 (0.06) | -0.11- 0.14 | 1999-2005 | .57 *** | .37 | .11 |
| *Fertility Schedule* |  |  |  |  |  |  |
| Total fertility rate | 1.95 (0.17) | 1.63-2.45 | 2010 | .51 *** | .43 | .02 |
| Initiation | 10.51 (1.68) | 6.06-13.34 | 2010 | .19 * | .44 | .13 |
| Peak | 26.83 (2.49) | 22.72-32.16 | 2010 | .60 *** | .20 | .23 ** |
| Stopping | 3.87 (0.82) | 1.28-5.16 | 2010 | .28 ** | .48 | .10 |
| *Markers of Fertility Norms* | |  |  |  |  |  |
| Age at first birth | 24.84  (1.19) | 22.60-27.70 | 2006 | .64 *** | .14 | .21 * |
| Age at first marriage | 27.31 (1.13) | 24.45-29.75 | 2010 | .48 *** | .37 | .08 |
| Percent never married | 30.70  (2.82) | 24.80-37.53 | 2010 | .34 *** | .38 | .17 * |
| Percent divorce | 1.46 (0.22) | 1.00-2.92 | 2010 | .45 *** | .60 | .31 *** |
| Percent cohabit | 6.88  (1.09) | 4.6-9.3 | 2010 | .50 *** | .48 | .07 |
| Non-marital fertility | 35.55 (6.22) | 15.80-51.20 | 2010 | .38 *** | .29 | .01 |
| Percent unintended pregnancy | 51.56 (5.44) | 38-65 | 2006 | .48 *** | .38 | -.01 |
| Abortion rate | 15.62 (8.35) | 0.90-40.0 | 2008 | .36 *** | .36 | -.07 |
| Family planning expenditures | 107.46 (43.31) | 31-245 | 2010 | .12 | .89 | .05 |
| *Sociodemographic Characteristics and Value Controls* | | | |  |  |  |
| Median income (in $1,000) | 49.76 (8.06) | 36.85-68.85 | 2010 | .28 ** | - | - |
| Percent African American | 10.34 (9.55) | 0.4-37.0 | 2010 | .64 *** | - | - |
| Percent Hispanic | 10.61 (9.88) | 1.2-46.3 | 2010 | .42 *** | - | - |
| Percent female | 50.66 (0.75) | 47.9-51.7 | 2010 | .53 *** | - | - |
| Percent with a B.A. | 27.16 (4.76) | 17.3-38.3 | 2010 | .35 *** | - | - |
| Percent urban | 73.58 (14.56) | 38.66-94.95 | 2010 | .30 *** | - | - |
| Vote for Obama | 50.51 (9.49) | 32.54-71.85 | 2008 | .18 * | - | - |
| Percent very religious | 39.62 (8.62) | 23.80-56.60 | 2010 | .11 | - | - |
| Note. SD = Standard Deviation. B.A. = Bachelor’s degree. Personality variables derived from data described in Rentfrow et al.^23^. Fertility variables were derived from data described in Martin et al.^38^. Sociodemographic characteristics were derived from the 2010 Census^45^. Vote for Obama was derived from the Federal Election Commission report^46^. Percent very religious was derived from a Gallup poll^47^. Moran’s I is a measure of spatial autocorrelation and was calculated based on a queen contiguity matrix. Residual variance refers to the proportion of variance in the main study variables remaining after sociodemographic characteristics and value variables were controlled. Residual Moran’s I refers to the spatial autocorrelation of the residuals.  * *p* < .05; ** *p* < .01; *** *p* <.001. | | | | | | |

| **Table S2 \| Personality-fertility associations by trait, subgroup, relative trait, and model** | | | | | | | | | | |
| --- | --- | --- | --- | --- | --- | --- | --- | --- | --- | --- |
|  | Subgroup Personality  (multiple regression) | | | | | | Relative Personality  (correlation) | | Trait Covariation  (multiple regression) | |
|  | β’s | | % *R*^2^ Common | β’s | | % *R*^2^ Common | Male - Female | < 30 -  ≥ 30 | β’s | % *R*^2^ Common |
|  | Male | Female |  | < 30 | ≥ 30 |  |  |  |  |  |
| **Panel A: Extraversion** |  |  |  |  |  |  |  |  |  |  |
| Total fertility rate | .24 | .01 | 61.56% | .06 | .24 | 55.96% | .13 | -.03 | -.38 | 20.84% |
| Initiation | -.16 | -.07 | 73.59% | -.31 | .17 | -29.15% | -.09 | -.22 | -.35 | -16.06% |
| Peak | .23 | .05 | 69.67% | .33 | -.15 | -14.58% | .13 | .23 | .19 | -8.15% |
| Stopping | -.15 | **.56** | 33.62% | **.70** | **-.42** | -35.54% | -.13 | **.51** | .17 | -4.25% |
| Age at first birth | .25 | -.26 | -76.42% | -.15 | .15 | -65.03% | .16 | -.13 | .42 | -2.66% |
| Age at first marriage | .08 | -.20 | 11.70% | -.23 | .07 | 7.99% | .06 | -.15 | .17 | 9.51% |
| Percent never married | .08 | -.02 | 40.52% | -.13 | .20 | -41.19% | .05 | -.12 | .17 | -7.27% |
| Percent divorce | -.21 | .10 | 3.53% | .20 | -.33 | -.37.27% | -.13 | .20 | -.07 | -5.60% |
| Percent cohabit | -.16 | -.12 | 76.44% | -.33 | .06 | 24.90% | -.08 | -.21 | .33 | 19.19% |
| Non-marital fertility | **-.53** | .41 | -58.95% | -.35 | .30 | -62.07% | **-.34** | -.28 | .13 | -3.56% |
| Percent unintended pregnancy | **-.44** | .04 | 52.52% | **-.86** | **.60** | -48.46% | -.25 | **-.64** | -.12 | 3.74% |
| Abortion rate | -.29 | .08 | 34.91% | **-.40** | .23 | -33.68% | -.17 | **-.28** | .10 | 22.08% |
| Family planning expenditures | -.15 | -.14 | 76.88% | **-.43** | .20 | -14.41% | -.07 | **-.30** | -.26 | -7.60% |
| **Panel B: Agreeableness** |  |  |  |  |  |  |  |  |  |  |
| Total fertility rate | .34 | .20 | 84.60% | .29 | .31 | 63.28% | -.09 | .22 | .12 | - |
| Initiation | .00 | .11 | 74.67% | -.16 | .33 | -19.91% | -.05 | -.13 | -.11 | - |
| Peak | .39 | -.21 | 2.75% | .13 | .07 | 60.83% | .11 | .10 | .45 | **-** |
| Stopping | .07 | -.03 | 30.05% | -.06 | .18 | 4.38% | .02 | -.05 | -.21 | **-** |
| Age at first birth | -.04 | -.11 | 82.58% | .13 | **-.37** | 1.30% | .05 | .10 | .46 | - |
| Age at first marriage | -.37 | .17 | 21.16% | .23 | **-.54** | -10.60% | -.09 | .18 | **.50** | - |
| Percent never married | -.23 | .41 | .87% | .33 | -.18 | -30.31% | -.21 | .25 | **.76** | **-** |
| Percent divorce | .23 | -.36 | -20.71% | -.34 | .24 | -48.71% | .18 | -.26 | -.47 | - |
| Percent cohabit | -.21 | -.23 | 85.54% | -.26 | -.24 | 63.23% | .11 | -.20 | -.14 | **-** |
| Non-marital fertility | **-.81** | **.69** | -72.68% | .22 | **-.42** | -25.46% | **-.36** | .17 | -.04 | - |
| Percent unintended pregnancy | -.29 | .18 | -13.66% | .15 | -.35 | -11.76% | -.09 | .12 | .32 | **-** |
| Abortion rate | -.20 | -.07 | 82.37% | -.02 | -.33 | 43.59% | .03 | -.01 | .08 | - |
| Family planning expenditures | -.19 | .28 | -.29.88% | .13 | -.07 | -27.21% | -.15 | .10 | .46 | - |
| **Panel C: Conscientiousness** | |  |  |  |  |  |  |  |  |  |
| Total fertility rate | .30 | .18 | 60.06% | **.41** | .05 | 33.56% | .04 | .25 | .10 | - |
| Initiation | .11 | .03 | 52.57% | -.09 | .08 | -49.39% | .03 | -.08 | -.28 | - |
| Peak | .27 | -.24 | -.59.39% | .25 | -.11 | -17.61% | .22 | .20 | .32 | **-** |
| Stopping | .22 | -.03 | 25.22% | **.55** | -.29 | -27.75% | .11 | **.46** | **.71** | **-** |
| Age at first birth | -.27 | -.01 | 41.15% | -.23 | -.01 | 27.60% | -.11 | -.15 | -.22 | - |
| Age at first marriage | -.31 | -.11 | 55.22% | -.27 | -.04 | 34.78% | -.08 | -.16 | -.12 | - |
| Percent never married | -.04 | -.04 | 61.77% | .24 | **-.38** | -36.88% | .00 | **.29** | -.18 | **-** |
| Percent divorce | .20 | -.03 | 24.98% | -.06 | .09 | -34.02% | .09 | -.07 | .21 | - |
| Percent cohabit | **-.36** | -.09 | 52.54% | **-.45** | -.01 | 26.12% | -.11 | **-.29** | -.39 | **-** |
| Non-marital fertility | -.31 | .07 | 16.57% | -.32 | .15 | -20.75% | -.16 | -.26 | -.55 | - |
| Percent unintended pregnancy | **-.38** | -.09 | 51.46% | **-.56** | .15 | -.80% | -.12 | **-.41** | **-.86** | **-** |
| Abortion rate | **-.37** | -.04 | 45.59% | **-.39** | .05 | 15.12% | -.13 | -.26 | -.30 | - |
| Family planning expenditures | -.27 | .14 | -26.60% | -.15 | -.05 | 42.15% | -.18 | -.08 | -.35 | - |
| **Panel D: Neuroticism** |  |  |  |  |  |  |  |  |  |  |
| Total fertility rate | -.31 | -.10 | 53.56% | **-.42** | -.10 | 53.38% | .12 | -.04 | -.27 | - |
| Initiation | -.01 | -.24 | 40.53% | .00 | -.28 | 40.16% | .13 | .17 | **-.59** | - |
| Peak | .01 | .04 | 48.61% | -.16 | .20 | -54.71% | -.01 | -.16 | **.48** | - |
| Stopping | -.30 | .18 | -37.85% | **-.39** | **.51** | -53.80% | **-.43** | **-.40** | **.48** | **-** |
| Age at first birth | .31 | .17 | 58.07% | **.43** | -.14 | 3.78% | .21 | .19 | **.60** | - |
| Age at first marriage | **.35** | .07 | 48.57% | **.40** | -.06 | 27.02% | .11 | .13 | **.55** | - |
| Percent never married | -.05 | .15 | -4.70% | .20 | -.23 | -61.58% | .03 | .18 | .37 | - |
| Percent divorce | -.02 | -.10 | 49.96% | -.14 | .17 | -55.96% | -.14 | -.14 | -.32 | - |
| Percent cohabit | .30 | -.11 | -4.94% | .30 | -.06 | 20.58% | .05 | .10 | -.13 | - |
| Non-marital fertility | .28 | -.32 | -58.37% | **.39** | **-.39** | -63.39% | .08 | **.33** | -.32 | **-** |
| Percent unintended pregnancy | **.53** | -.28 | -27.29% | **.61** | **-.56** | -62.54% | **.45** | **.48** | -.27 | **-** |
| Abortion rate | **.50** | -.21 | -11.27% | .43 | -.17 | -6.08% | .21 | .21 | .27 | - |
| Family planning expenditures | .10 | -.01 | 30.63% | .29 | **-.43** | -44.97% | **.31** | **.33** | -.17 | **-** |
| **Panel E: Openness** |  |  |  |  |  |  |  |  |  |  |
| Total fertility rate | -.01 | **-.53** | 50.54% | -.13 | **-.49** | 62.41% | **.31** | .17 | **-.49** | - |
| Initiation | .25 | -.23 | -68.42% | .04 | -.10 | -7.96% | .17 | .06 | -.23 | - |
| Peak | **-.48** | .34 | -49.44% | -.10 | .07 | -41.54% | -.28 | -.06 | .35 | - |
| Stopping | -.14 | -.14 | 70.12% | -.16 | -.08 | 68.06% | .06 | -.02 | -.04 | - |
| Age at first birth | -.16 | .36 | -6.82% | -.20 | **.50** | 2.55% | -.24 | **-.28** | .35 | **-** |
| Age at first marriage | -.29 | **.52** | -24.72% | -.26 | **.65** | 2.34% | **-.35** | **-.37** | .36 | **-** |
| Percent never married | -.12 | .12 | -69.64% | -.33 | **.43** | -57.04% | -.09 | **-.30** | .36 | **-** |
| Percent divorce | .31 | -.12 | 3.10% | .28 | -.18 | -39.21% | .12 | .17 | .06 | - |
| Percent cohabit | -.06 | **.55** | 40.05% | -.08 | **.68** | 39.67% | **-.34** | **-.32** | **.41** | **-** |
| Non-marital fertility | -.29 | .34 | -66.24% | -.32 | **.46** | -48.91% | -.25 | **-.31** | -.07 | **-** |
| Percent unintended pregnancy | -.02 | .21 | 40.96% | .01 | .20 | 54.13% | -.13 | -.08 | -.12 | - |
| Abortion rate | -.04 | .23 | 31.33% | -.11 | .35 | 16.37% | -.14 | -.19 | -.04 | - |
| Family planning expenditures | -.02 | .18 | 40.96% | -.28 | **.50** | -26.73% | -.11 | **-.31** | .14 | **-** |
| Note. The second and third columns report standardized regression coefficients (β) from a multiple regression using male and female personality. The fourth column reports the percent of the total effect due to common variance of male and female personality. The fifth through seventh column report similar multiple regression results for personality age groups. The eighth and ninth column report correlations between relative personality (i.e., male - female personality and young - old personality) and fertility. The tenth column reports multiple regression results from models that included each personality trait to control for trait covariation. A total of 13 models were fit, and parameters are listed separately for ease of presentation. The final column reports the percent of the total effect explained by variance common to all personality traits. Parameters printed in bold are significant at *p* < .05. Negative % Common values indicate suppressor effects. All variables adjusted for sociodemographic characteristics and value controls. | | | | | | | | | | |

1. Retrieved from https://www.census.gov/geo/maps-data/data/tiger.html. [↑](#footnote-ref-1)
